# Expanding the Paradigm of Structure-Based Drug Design: Molecular Dynamics Simulations Support the Development of New Pyridine-Based Protein Kinase C-Targeted Agonists

**DOI:** 10.1021/acs.jmedchem.2c01448

**Published:** 2023-04-03

**Authors:** Saara Lautala, Riccardo Provenzani, Ilari Tarvainen, Katia Sirna, S. Tuuli Karhu, Evgeni Grazhdankin, Antti K. Lehtinen, Hanan Sa’d, Artturi Koivuniemi, Henri Xhaard, Raimo K. Tuominen, Virpi Talman, Alex Bunker, Jari Yli-Kauhaluoma

**Affiliations:** † Drug Research Program, Division of Pharmaceutical Biosciences, University of Helsinki, P.O. Box 56 (Viikinkaari 5 E), FI-00014 Helsinki, Finland; ‡ Drug Research Program, Division of Pharmaceutical Chemistry and Technology, University of Helsinki, P.O. Box 56 (Viikinkaari 5 E), FI-00014 Helsinki, Finland; ¶ Drug Research Program, Division of Pharmacology and Pharmacotherapy, University of Helsinki, P.O. Box 56 (Viikinkaari 5 E), FI-00014 Helsinki, Finland; § School of Pharmacy, The University of Jordan, Queen Rania Street, 11942 Amman, Jordan

## Abstract

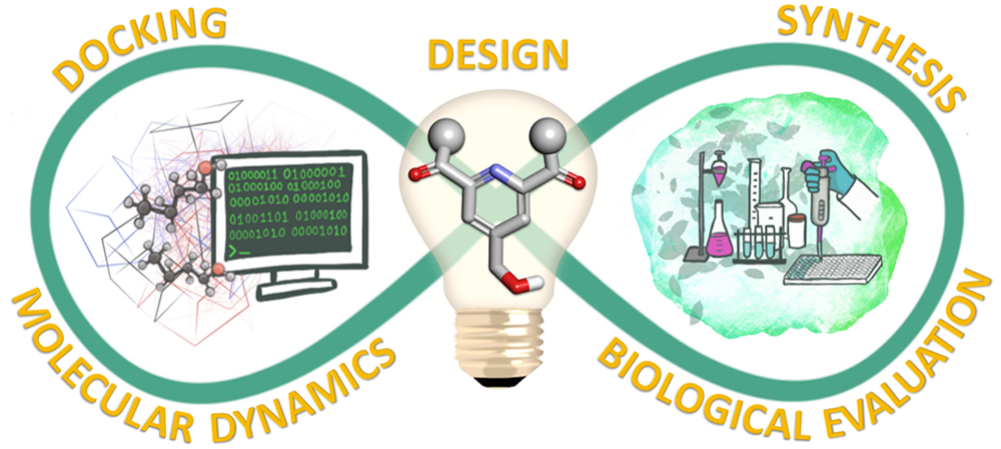

Protein kinase C (PKC) modulators hold therapeutic potential for various diseases, including cancer, heart failure, and Alzheimer’s disease. Targeting the C1 domain of PKC represents a promising strategy; the available protein structures warrant the design of PKC-targeted ligands via a structure-based approach. However, the PKC C1 domain penetrates the lipid membrane during binding, complicating the design of drug candidates. The standard docking–scoring approach for PKC lacks information regarding the dynamics and the membrane environment. Molecular dynamics (MD) simulations with PKC, ligands, and membranes have been used to address these shortcomings. Previously, we observed that less computationally intensive simulations of just ligand–membrane interactions may help elucidate C1 domain-binding prospects. Here, we present the design, synthesis, and biological evaluation of new pyridine-based PKC agonists implementing an enhanced workflow with ligand–membrane MD simulations. This workflow holds promise to expand the approach in drug design for ligands targeted to weakly membrane-associated proteins.

## Introduction

Abnormalities in protein kinase C (PKC) activity correlate with multiple pathologies.^1,2^ Considering the number of cellular signaling cascades it contributes to, this is expected. The PKC isozyme family is a group of ten proteins that, while inactive, reside in the cytoplasm and, upon activation, relocate to the cytosolic leaflets of cellular membranes to phosphorylate specific substrates.^3^ The family is composed of three subfamilies: classical PKCs (cPKCs), novel PKCs (nPKCs), and atypical PKCs (aPKCs), which differ in both structure of their regulatory domain and mode of activation.^2,3^ The isozymes in the cPKC subfamily are activated in the presence of Ca^2+^ and 1,2-diacyl-*sn*-glycerol (DAG); nPKCs only need DAG, while aPKCs are activated via phospholipids and protein–protein interactions.^3^ Targeting this family of enzymes can potentially treat heart, lung, and kidney disease, diabetes, cancer, and even neurodegenerative conditions including Alzheimer’s disease.^1,3−5^

The major challenge in targeting PKC for therapeutic purposes is to achieve a reversible activation of the enzyme. The natural agonist DAG can induce both transient and sustained reversible activation of PKC isozymes; these types of stimulations correspond to the physiological activation of the isozymes and thus correlate with beneficial biological effects. Instead, activation by ultrapotent agonists (e.g., phorbol esters) is irreversible and induces downregulation of the enzyme that can lead to tumor-promoting effects.^4^

For this reason, mimicking the DAG-triggered reversible activation by binding to the C1 domain of PKC has been attempted earlier with a variety of nature-derived, semisynthetic, and synthetic compounds, including bryostatins, aplysiatoxin, and alotaketal analogs,^6,7^ ingenols,^8^ indolactams and benzolactams,^9^ DAG-lactones,^10,11^ 2-aryl-3-hydroxypropyl esters,^12^ isophthalates (HMIs),^13^ and pyrimidines (PYRs).^14^ However, finding compounds with sufficient specificity, potency, and a practical synthesis pathway has proved challenging. Small alterations of the molecular structure of the ligand can lead to unexpected and drastic changes in its behavior.^14^ Even the fine details of DAG-induced PKC modulation (e.g., isozyme selectivity of certain DAG species and analogs) are not yet fully understood in spite of many examples of both experimental^15−18^ and computational studies^19−21^ having been carried out.

The current lack of knowledge concerning the precise behavior and detailed activation mechanism of PKC at the membrane interface likely compromised numerous attempts to target the enzyme via its C1 domain. As standard docking and biological experiments alone seemingly cannot provide a sufficient level of information, studying the behavior of the protein and both endogenous/exogenous C1 domain-targeted ligands through molecular dynamics (MD) simulations have been used to elucidate this phenomenon.^7,12,19,22−26^ Most of these studies incorporated the PKCδ C1B domain (PDB ID: 1PTR(27)), the ligand of interest, and the relevant membrane. Unfortunately, these types of simulations can be extremely laborious due to their complex nature. Our goal is to develop a less computationally intensive protocol that allows us to screen a larger number of compounds.

Traditionally, these studies highlighted the key hydrogen bonds (H-bonds) between the hydroxy and a carbonyl group of the ligands with the C1 domain backbone of the enzyme,^13,14,19^ but interestingly, evidence of relevant ligand–membrane interactions also exists. In our previous work,^14^ we observed that modifying the structure of active lead compounds (e.g., HMI-1a3) by switching the phenyl core with a pyrimidine (e.g., PYR-1gP) to improve the hydrophilicity of the scaffold leads to a precipitous decrease in binding affinity. We discovered a possible explanation by carrying out a set of MD simulations of the ligands in the relevant bilayer: an intramolecular H-bond between the hydroxy group and one nitrogen atom of the pyrimidine core, enhanced by the lipophilic membrane environment, changes the orientation of the compounds in the membrane.^23^ Further, Ryckbosch et al. have determined that even the H-bonding pattern of the ligand at the lipid surface can differentiate ligands with tumor-promoting/suppressing behaviors.^22^

We thus hypothesized that the protein-independent positioning and orientation of a ligand candidate in the membrane environment may predict binding prospects. Hence, ligand–membrane MD simulations could provide an attractive, additional, and possibly alternative way of screening for potentially active ligand candidates targeted to membrane-associated proteins with more rigor than only docking calculations but using less computational resources than extensive MD simulation. This is supported by previous studies focused on the effects of membrane orientation for various drug molecules,^28−32^ and regarding ligands targeted to the membrane-bound catechol-*O*-methyltransferase, MD simulations changed the drug design paradigm.^33^

In this work, we aimed at developing new PKC activators by building on this assumption and our previous efforts with PKC modulators.^13,14,23^ Instead of merely using the ligand–membrane MD simulations to determine the cause of failure of a previously synthesized and tested compound (i.e., a retrospective analysis method), we incorporate them as an additional integral component of the structure-based design phase to develop new PKC activators. Thus, the augmented drug design protocol we propose includes five steps: (1) design of a virtual library of compounds containing a pyridine-based scaffold, (2) preliminary virtual screening by “traditional” molecular docking (fit to the binding site and analysis of ligand–protein interactions), (3) MD simulations (*in silico* behavior; dynamics and orientation in the relevant membrane environment) of the candidates selected by the docking, (4) synthesis of the selected candidates that showed appropriate behavior in MD simulations, and finally, (5) biological assays (*in vitro* behavior) and validation of the predictions. As a result, we present two new PKC agonists and discuss the potential of our augmented approach as a convenient strategy to decrease false docking-based predictions for ligands targeting PKC and other membrane-associated proteins.

## Results

### Design and Molecular Docking

**Figure 1:**
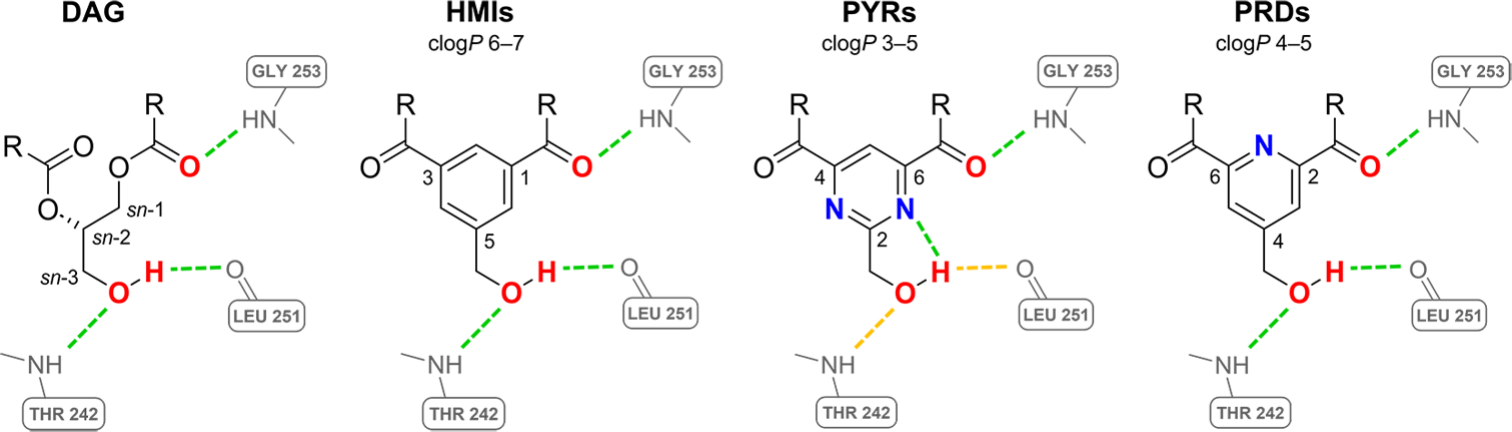
Comparison between the scaffolds of 1,2-diacyl-*sn*-glycerol (DAG), isophthalates (HMIs), pyrimidines (PYRs), and the newly designed pyridines (PRDs). Possible hydrogen interactions with key amino acids (gray) of the PKCδ C1B domain (PDB ID: 1PTR) backbone are highlighted in green (strong) and orange (weak) dashed lines. Hydroxy groups and carbonyl oxygens needed for the ligand–protein interactions are highlighted in red, and nitrogen atoms are highlighted in blue.

We based the design of the new scaffold on the HMIs^13^ (Figure 1), amphipathic ligands that exhibit PKC-mediated biological effects by mimicking the endogenous ligand DAG, targeted to the C1 domain of the enzyme^34−38^ and the information gained from our previous MD study.^23^ As the most active HMIs possess poor water solubility due to their clog*P* values ranging from 6 to 7, in previous work, we designed and synthesized HMI analogs based on the more hydrophilic pyrimidine core (i.e., PYRs, Figure 1).^14^ However, an intramolecular H-bond between the hydroxy group and a nitrogen atom of the pyrimidine core possibly caused the loss of affinity of the PYRs for the target.^23^

**Figure 2:**
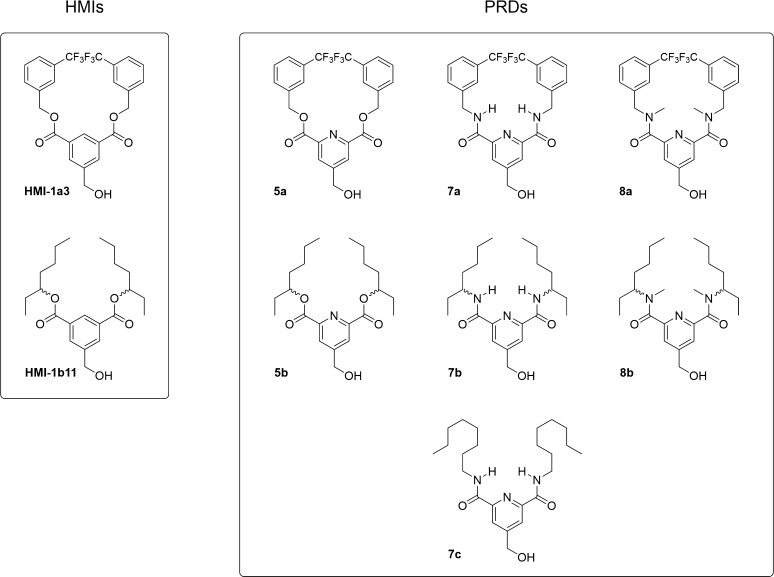
Structures of the isophthalate reference compounds (HMIs, left) and the set of designed 2,4,6-trisubstituted pyridines (PRDs, right).

Again, by scaffold hopping, we designed a set of new compounds (PRDs, Figure 1) introducing a pyridine core, another nitrogen-containing heterocycle. The trisubstituted pyridine bearing the hydroxymethyl group in position 4 would still reduce the overall clog*P* of the scaffold in comparison to the phenyl of the HMIs and would also avoid the undesired intramolecular H-bond between the hydroxy group and the nitrogen of the heterocyclic core of the PYRs.^23^ We maintained the hydroxy and carbonyl groups, needed for the ligand–protein interactions, in the same relative positions. To obtain a comprehensive understanding of the ligand–protein interactions, we designed both ester and amide derivatives. We tailored the design and molecular docking study to a set of seven trisubstituted PRDs (Figure 2) bearing (1) the hydroxymethyl group in position 4 and (2) ester (**5a** and **5b**), secondary (**7a**–**c**), or tertiary (**8a** and **8b**) amide groups in positions 2 and 6. For both ester/amide moieties, we chose the hydrophobic substituents 3-(trifluoromethyl)benzyl and 3-heptyl to evaluate the designed compounds as the corresponding analogs of the active isophthalates HMI-1a3 and HMI-1b11, respectively.^13,35−37^ We also included in the study **7c**, bearing octyl substituents, since during the syntheses we used the readily available octylamine to test the reaction conditions before obtaining compounds **7a** and **7b**.

**Figure 3:**
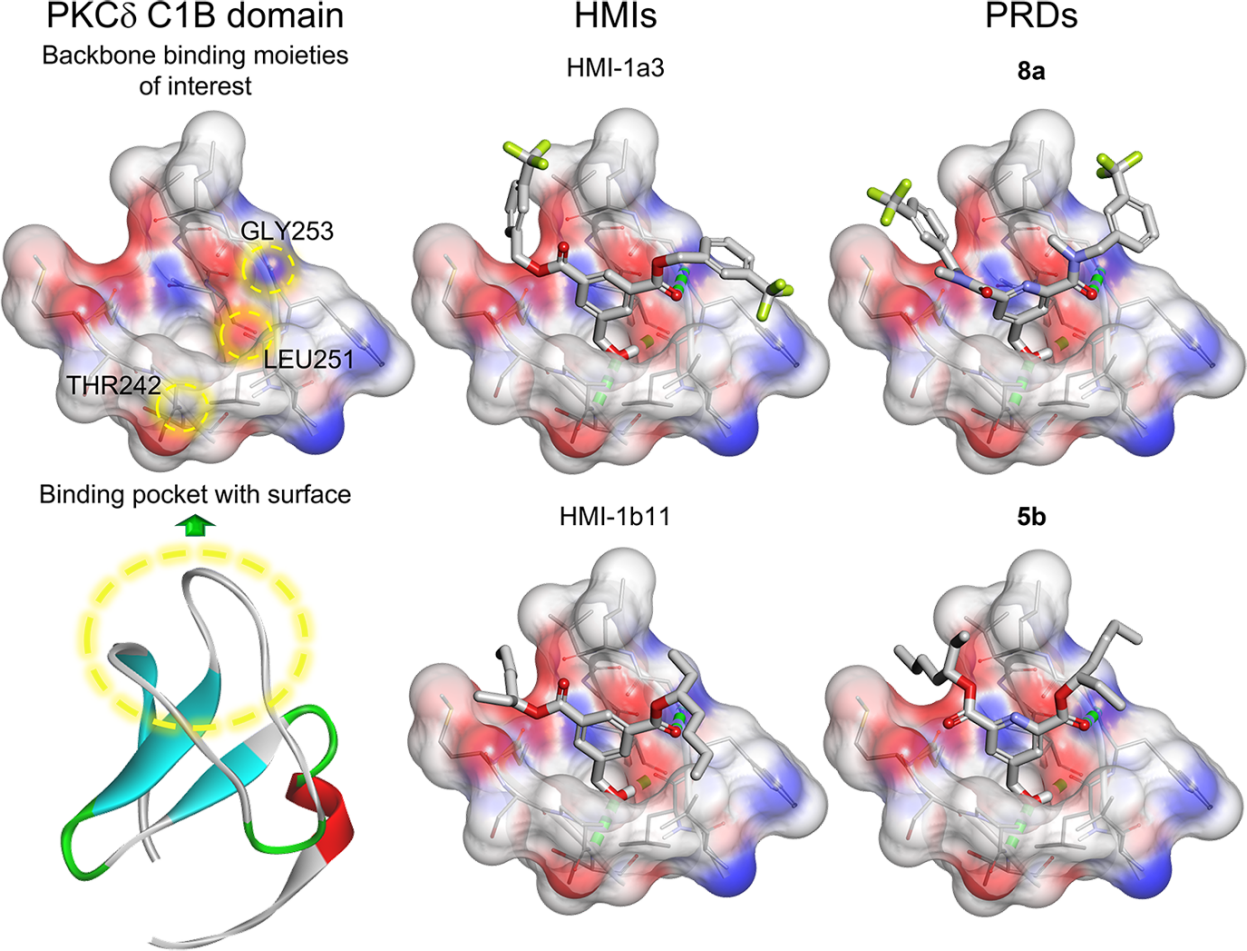
Graphical representation of the molecular docking. The figure shows the crystal structure of the PKCδ C1B (PDB ID: 1PTR) (left) and the comparison of the docking poses of HMI-1a3 and HMI-1b11 (middle) with **8a** and **5b** (right), respectively. In the 3D ribbon protein structure, α-helix, β-sheets, and β-turns are colored red, cyan, and green, respectively. Binding pocket and backbone binding moieties of interest are highlighted with yellow dashed circles. Atom color code: carbon, light gray; oxygen, red; nitrogen, blue; fluorine, lime; hydrogen, white. Hydrogen bonds are shown as green dashed lines.

We docked the seven PRDs into the crystal structure of the PKCδ C1B domain (PDB ID: 1PTR(27)) together with HMI-1a3 and HMI-1b11 as the positive controls. With this docking study, we assessed only that the new candidates maintain the desired H-bonds with the C1 domain backbone: (1) the hydroxy group engages as both H-bond acceptor/donor with the amide of Thr242 and carbonyl of Leu251, respectively, and (2) one of the carbonyl groups acts as the H-bond acceptor toward the amide of Gly253 (Figure 3). All seven PRDs docked correctly and returned comparable scores to the control compounds (the full list is available in the Supporting Information, SI, Docking scores and SMILES of all tested compounds).

However, as PKC is a weakly membrane-associated protein and the docking setting lacks the membrane environment, the docking scores do not fully correlate to the binding affinity. We previously demonstrated that the behavior of the ligands in the membrane plays a crucial role for the successful binding to the target.^23^ We thus performed MD simulations of the ligands in a phospholipid bilayer environment to mimic the binding assay conditions to obtain a more comprehensive prediction of their possible activity.

### Molecular Dynamics

**Figure 4:**
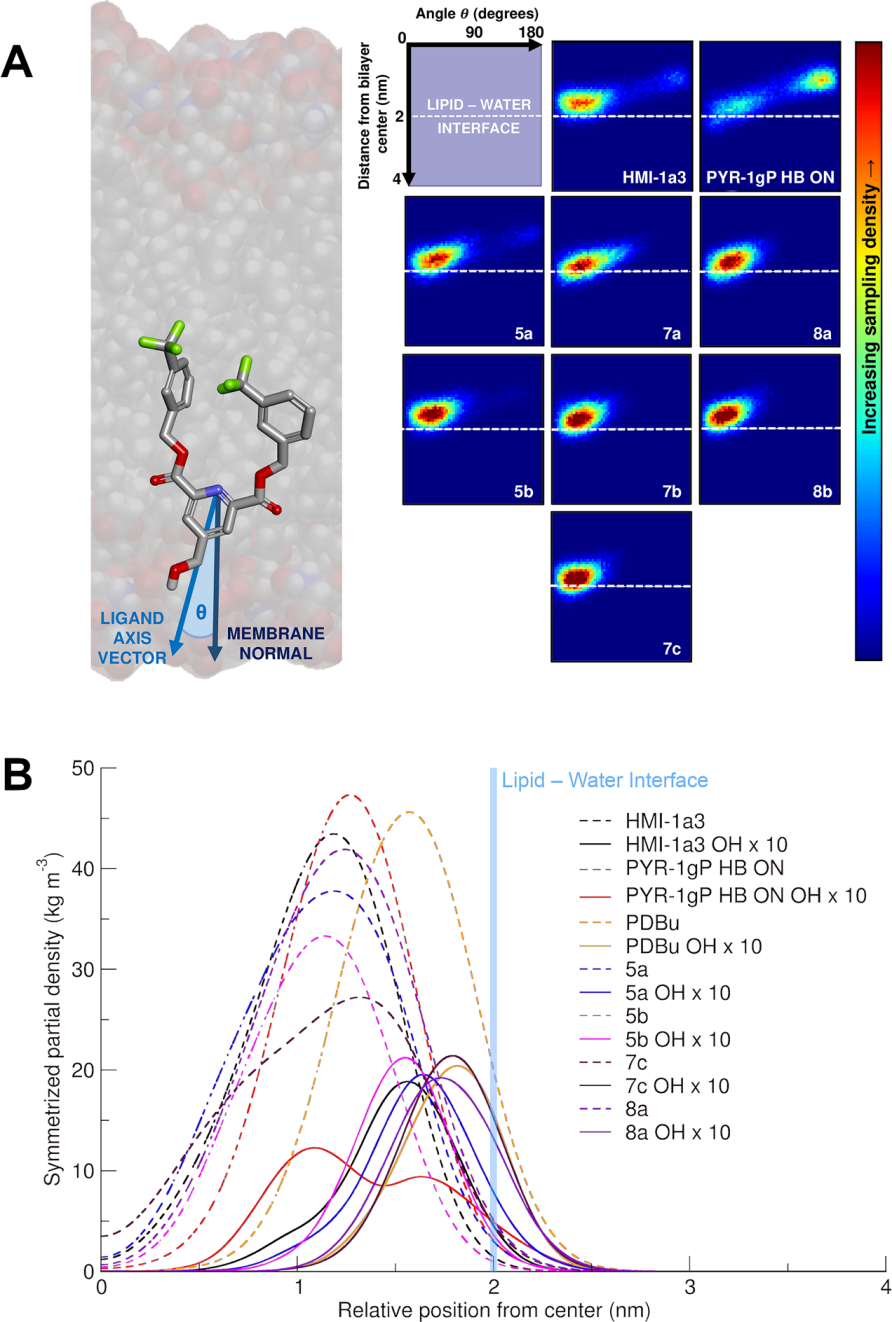
(A) Orientation–distance analysis presented in 2D heat maps (right) accompanied by a graphical representation of the ligand axis vector in relation to the membrane normal (left). The *x*-axis of the heat maps represents the angle θ, while the *y*-axis displays the distance of the hydroxy groups from the bilayer center along the membrane normal. (B) Partial density profiles of full HMI-1a3, PYR-1gP_HB–ON_, PDBu, **5a**, **5b**, **7c**, and **8a** (dashed lines) and their hydroxy groups (solid lines). The lipid–water interface is displayed as a blue vertical line.

Visual analysis of the simulation trajectories found good behavior for all tested candidates; all new compounds exhibited HMI-like behavior, with their hydroxy groups orienting toward the lipid–water interface. We observed attractive interactions between the ligand candidates that lead to the transient formation of ligand clusters during the simulations. The clusters formed and disassembled spontaneously during the simulations and did not significantly affect the results. However, our simulations did not provide sufficient data to determine the prevalence of this phenomenon. We quantified the behavior of the ligand candidates using population heat maps, in which we plotted the orientation of the candidates and the position of their hydroxy groups (Figure 4A). We defined the orientation of the candidates by the angle θ between the membrane normal and the ligand central axis vector (Figure 4A, left). We determined the position of the hydroxy groups of the candidates by measuring the vertical distance between the center of mass (COM) of the membrane and the COM of each hydroxy group. Along with the new candidates, we included the population heat maps of HMI-1a3 and PYR-1gP with engaged intramolecular H-bond (PYR-1gP_HB–ON_) as positive and negative controls, respectively (Figure 4A, top-right).

The main population of HMI-1a3 resides at ∼1.6 nm from the center of the membrane core and with an angle θ ≲ 50° from the membrane normal axis vector. Instead, the main population of PYR-1gP_HB–ON_ locates at ∼1 nm with an angle θ ≳ 130°, considerably closer to the bilayer center than HMI-1a3. These populations represent our references for the “correct” (i.e., HMI-1a3) and “incorrect” (i.e., PYR-1gP_HB–ON_) orientation in the membrane and correspond to previous results obtained using the OPLS-AA force field.^23^ Small populations of the opposing orientations exist in both simulations; HMI-1a3 may orient incorrectly due to clustering during the simulation, while PYR-1gP_HB–ON_ can occasionally orient correctly despite the intramolecular H-bond.^23^

All new candidates clearly present the desired behavior (Figure 4A). The distance from the bilayer center varies between ∼1.6 and ∼1.8 nm, and the angle remains mostly within the range of 15° ≲θ≲ 50°. This indicates both desired location (near the phosphate groups of the lipids) and orientation (constrained to a relatively small angle).

Similarly to our previous study,^23^ we calculated (1) the positioning via partial density analysis (Figure 4B), (2) the number of H-bonds per molecule with both water and lipid bilayer (Table 1), and (3) the solvent-accessible surface area (SASA) of the hydroxy groups (Table 2) for compounds **5a**, **5b**, **7c**, and **8a**. We included the reference compounds HMI-1a3 and PYR-1gP_HB–ON_ as the positive and negative controls, respectively. Additionally, in the partial density analysis, we included the potent PKC activator and tumor promoter phorbol-12,13-dibutyrate (PDBu).

**Table 1:** Number of Hydrogen Bonds per Molecule between the Hydroxy Group (OH) and Ester/Amide Groups (OO/NO) of the Ligand Candidates with Both Water and 1-Stearoyl-2-docosahexaenoyl-*sn*-glycero-3-phospho-l-serine (SDPS)^a^

	OH		OO/NO	
compound	water	SDPS	water	SDPS
HMI-1a3	0.83 ± 0.06	0.56 ± 0.06	0.79 ± 0.07	0.041 ± 0.013
PYR-1gP	0.46 ± 0.12	0.11 ± 0.05	0.98 ± 0.07	0.11 ± 0.04
**5a**	0.77 ± 0.04	0.54 ± 0.05	0.85 ± 0.06	0.08 ± 0.05
**5b**	0.88 ± 0.05	0.60 ± 0.04	0.84 ± 0.03	0.022 ± 0.013
**7c**	0.79 ± 0.04	0.62 ± 0.04	1.26 ± 0.02	0.06 ± 0.02
**8a**	0.86 ± 0.03	0.62 ± 0.04	1.30 ± 0.07	0.07 ± 0.03

a Values are time-block averages (5 blocks, 300 ns each) for all four ligand candidates in each trajectory presented with standard deviation as the error.

**Table 2:** Solvent Accessible Surface Area (SASA) in nm^2^ per Molecule for Hydroxy (OH) and Ester/Amide Groups (OO/NO) of the Ligand Candidates^a^

compound	OH	OO/NO
HMI-1a3	0.059 ± 0.005	0.064 ± 0.009
PYR-1gP	0.06 ± 0.02	0.110 ± 0.007
**5a**	0.057 ± 0.007	0.067 ± 0.006
**5b**	0.059 ± 0.007	0.051 ± 0.004
**7c**	0.072 ± 0.006	0.063 ± 0.002
**8a**	0.09 ± 0.01	0.072 ± 0.005

a Values are time-block averages (5 blocks, 300 ns each) for all four ligand candidates in each trajectory presented with standard deviation as error.

As expected, the density profiles tell a similar story as the orientation–distance heat maps; all analyzed PRDs show comparable profiles with those of the positive control HMI-1a3. Only minor variations appear in the positioning of the density peak of the different structures. The hydroxy group of **5b** clearly overlaps with that of HMI-1a3, while the rest are predominantly located closer to the lipid–water interface (Figure 4).

All analyzed PRDs show a comparable behavior to HMI-1a3 also in both H-bond and SASA analyses. Minor variations appear among them within error estimates here as well, and no distinguishing trend appears for the analyzed candidates.

Overall, the MD results presented marginal differences in behavior among the ligand candidates. As all PRDs represented potential successful hits, we decided to synthesize and biologically evaluate all seven compounds.

### Synthesis

**Scheme 1:**
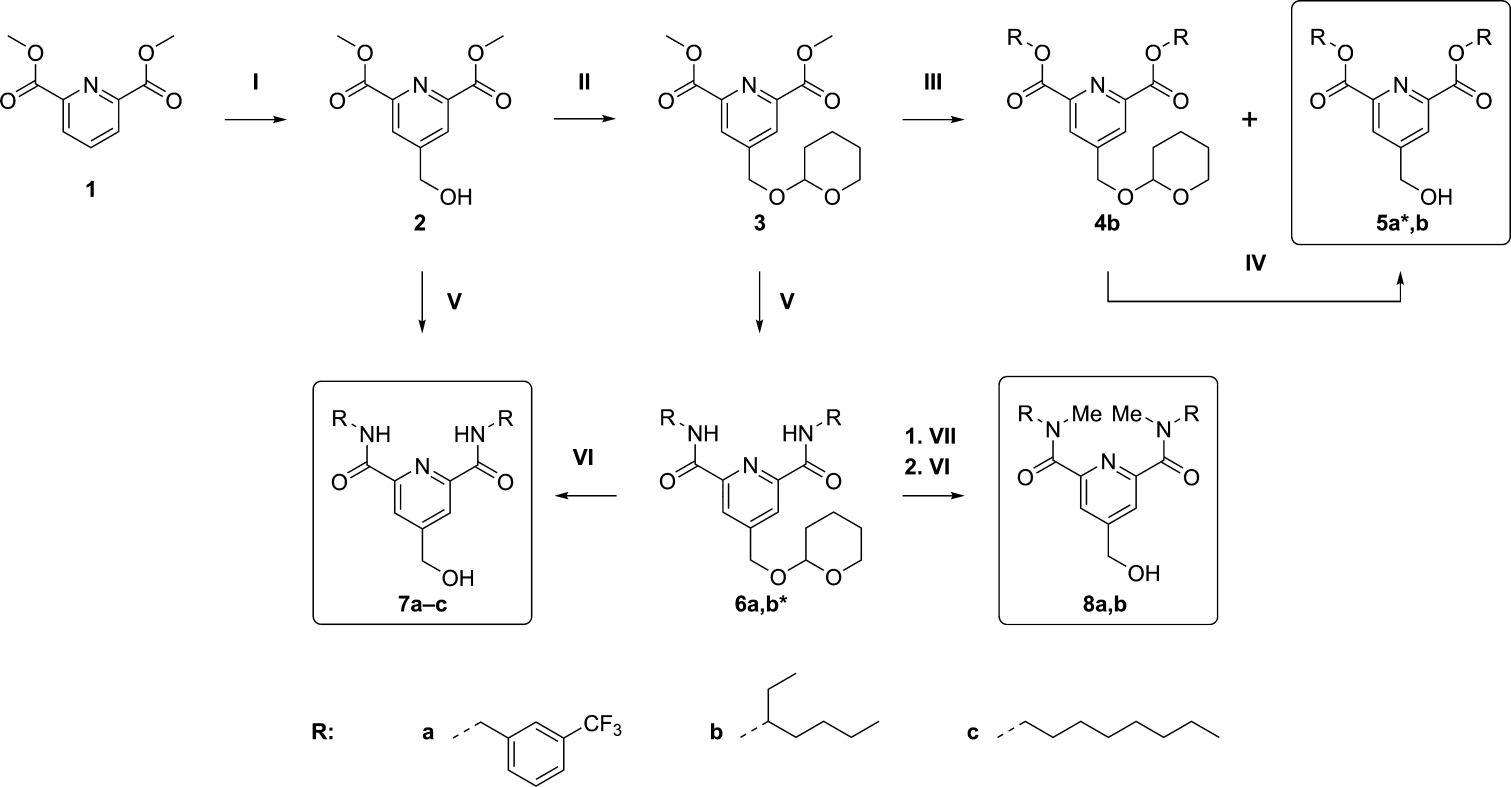
Synthesis of the Pyridine Derivatives Conditions: (I) Fe(ClO_4_)_2_, HClO_4_, H_2_O_2_, MeOH/H_2_O, 0–10 °C, 1 h → rt, 3 h, 50%; (II) DHP, PPTS, DCE, rt, 6 h, 83%; (III) Zn_4_(OCOCF_3_)_6_O, R–OH, *i*-Pr_2_O, 100 °C, 72 h, 41% (**4b**) + 16–30% (*5a: also in solvent-free conditions, MW 80 °C, 6 h + MW 100 °C, 15 h, 25%); (IV) Dowex 50WX8, MeOH, 40 °C, 23 h, 25%; (V) R–NH_2_, MeOH, MW 120 °C, 6–12 h, 36–80% (*6b: NaH, Me–THF, rt, 1.5 h + MW 40 °C, 2.5 h, 34%); (VI) Montmorillonite K10, MeOH, MW 55 °C, 8 h, 73–84%; (VII) NaH, MeI, THF, rt, 16 h (crude product).

We obtained the desired PRDs in two to five steps (Scheme 1). We performed a Fenton’s oxidation-type reaction (I, Scheme 1) by treating the commercially available dimethyl 2,6-pyridinedicarboxylate (**1**) with H_2_O_2_ in a solution of Fe(ClO_4_)_2_ in methanol and water in the presence of HClO_4_ to introduce the hydroxymethyl group in position 4 of the pyridine core.^39^ Hence, we protected the hydroxy group of the intermediate **2** as a tetrahydropyranyl (THP) ether^13^ to perform the subsequent transesterification and amidation reactions.

The bis-methyl ester **3** underwent Zn-catalyzed transesterifications (III, Scheme 1) by reacting in the presence of the μ-oxo-tetranuclear zinc cluster Zn_4_(OCOCF_3_)_6_O and 3-(trifluoromethyl)benzyl or 3-heptyl alcohols in *i*-Pr_2_O. We prepared Zn_4_(OCOCF_3_)_6_O and performed the reactions by adapting the procedures reported by Iwasaki et al. on similar compounds.^40^ These transesterifications directly afforded the THP-free bis(3-trifluoromethyl)benzyl ester (**5a**) in low yield (16%) while giving both THP-protected (**4b**) and THP-free (**5b**) bis-3-heptyl esters in a 3:2 molar ratio. From a green chemistry perspective, since *i*-Pr_2_O appears in the list of undesirable solvents, we attempted a greener approach by performing this reaction in neat conditions. We microwave irradiated the bis-methyl ester **3** in the presence of the Zn cluster, with 3-(trifluoromethyl)benzyl alcohol as the solvent, and we obtained the bis-3-(trifluoromethyl)benzyl ester **5a** with an improved yield (25%). Since we isolated the bis-3-heptyl ester **5b** from both transesterification and THP-deprotection reactions in a sufficient amount for the biological testing, we did not attempt the transesterification of the bis-methyl ester **3** with 3-heptanol in neat conditions. For the THP deprotection of the bis-3-heptyl ester **4b**, we followed the method previously applied for the deprotection of the HMIs^13^ using the acid resin Dowex 50WX8 in MeOH (IV, Scheme 1) and obtained the THP-free bis-3-heptyl ester **5b** in moderate yield (25%).

We obtained the amide **6a** (62% yield) by microwave irradiating the bis-methyl ester **3** with the commercially available 3-(trifluoromethyl)benzylamine in MeOH, adapting a method reported by Zubenko et al. on related compounds.^41^ For the 3-heptyl derivative, we synthesized 3-heptanamine (**SI2**, Scheme S1) via reductive amination of 3-heptanone (**SI1**, Scheme S1) as described by Kapoor et al.^42^ The synthesis of the bis-3-heptyl derivative **6b** required a stronger basic environment as the same conditions applied for the bis-3-(trifluoromethyl)benzyl derivative **6a** generated only the monosubstituted product in low yield. We first pretreated 3-heptanamine (**SI2**) with NaH and then reacted it with the bis-methyl ester **3** in THF under microwave irradiation to obtain the bis-3-heptyl amide **6b** in 36% yield. We also substituted THF with the greener Me-THF (V, Scheme 1), and it resulted in a slightly longer reaction time but a comparable yield (34%).

Since the THP deprotection of the bis-3-heptyl ester **4b** using Dowex 50WX8 produced an inferior yield in comparison with the HMIs, to deprotect the bis-3-heptyl amide **6b**, we adapted a method described by Li et al.^43^ using the environmentally friendlier acid clay Montmorillonite K10 in MeOH (VI, Scheme 1). The reaction gave the product **7b** in good yield (84%). We obtained the final products **7a** and **7c** in fair–good yields (>60%) by applying conditions V (Scheme 1) in the presence of 3-(trifluoromethyl)benzylamine and 3-octylamine, respectively, directly on the intermediate **2**. Finally, we alkylated intermediates **6a** and **6b** using MeI in the presence of NaH in THF (VII, Scheme 1) and subsequently cleaved the THP protective groups with Montmorillonite K10 to obtain the final products **8a** and **8b**, respectively, in good yields (>70%).

Regarding the stereochemistry of the PRDs, the 3-heptyl-containing products **5b**, **7b**, and **8b** were isolated as diastereomeric mixtures. The different stereoisomers (i.e., enantiomers *R*|*R* and *S*|*S* and *meso* compounds) formed since we utilized 3-heptanol and 3-heptanamine, both containing the stereogenic center C3, as racemic compounds in the transesterification (III) and amidation (V) steps, respectively (Scheme 1). While the ^1^H and ^13^C NMR spectra did not clearly display the stereoisomerism of the 3-heptyl-containing PRDs, possibly due to the high degree of freedom of rotation around the stereogenic carbons, the ^1^H and ^13^C NMR spectra of PRDs **8a** and **8b** showed that both compounds present mixtures of conformers due to the *cis*–*trans* isomerism that can take place upon amide bond formation and further alkylation (steps V and VI, Scheme 1). To further confirm that we obtained a mixture of conformers and exclude the presence of impurities, we conducted a variable temperature NMR experiment study (all NMR spectra and their comparisons are available in the Supporting NMR Appendix, SI).

### Biological Evaluation and Structure–Activity Relationship Analysis

**Figure 5:**
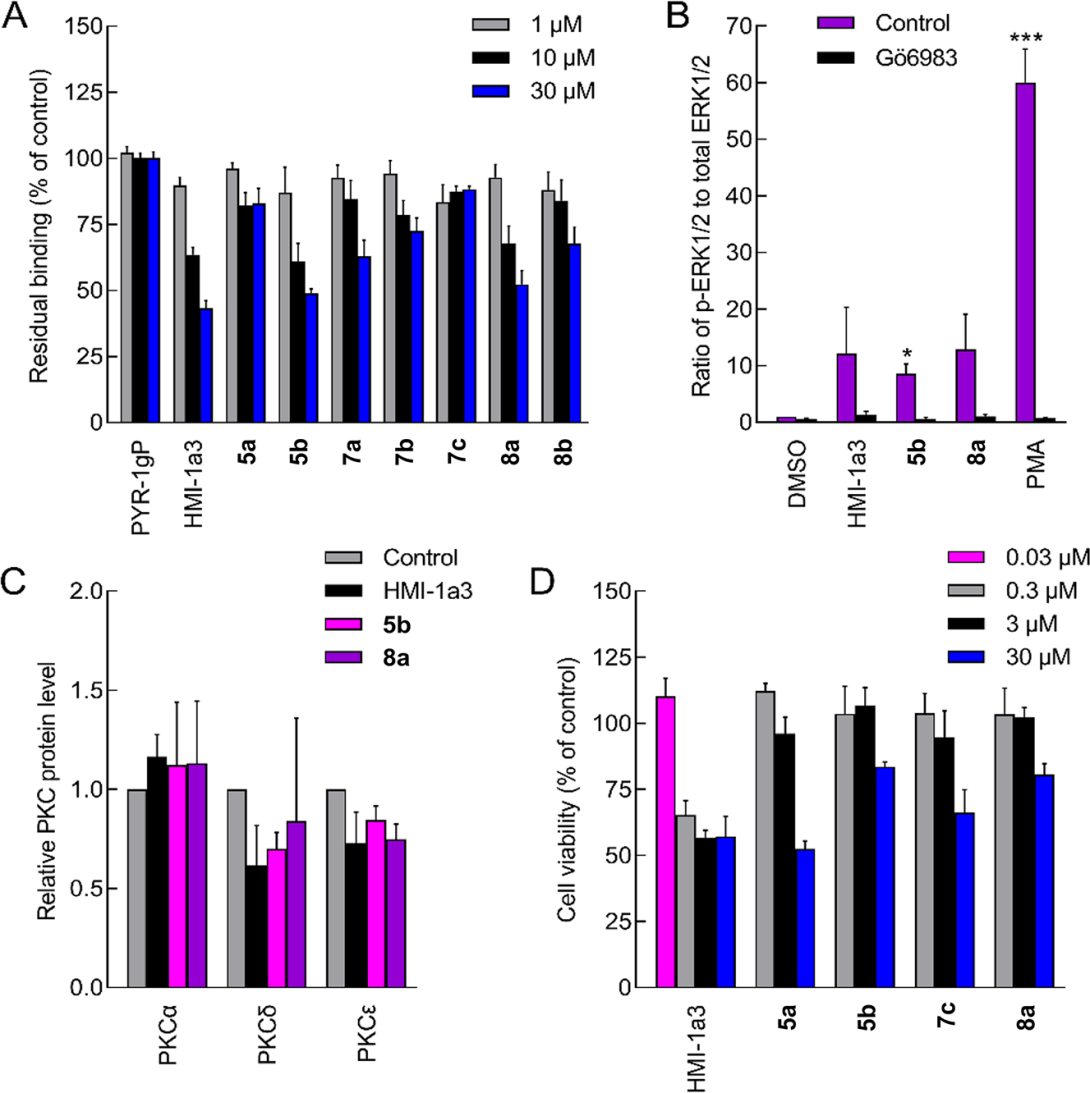
Biological evaluation of the pyridine derivatives (PRDs). (A) The binding affinity of PRDs in comparison with PYR-1gP and HMI-1a3 as negative and positive controls, respectively. The binding of [^3^H]PDBu (10 nM) to purified PKCα was measured in the presence of increasing concentrations of the compounds. The results are expressed as mean + SEM (*N* = 3) of residual [^3^H]PDBu binding (% of control). (B) The effect of pyridines on PKC-mediated ERK1/2 phosphorylation in neonatal mouse cardiac fibroblasts. The cells were exposed to the compounds (at 10 μM concentration, except for PMA at 10 nM concentration) with or without PKC inhibitor (1 μM Gö6983) for 30 min. The amount of total ERK1/2 and p-ERK1/2 was quantified from cellular lysates using Alpha technology. The results are normalized to DMSO-control and expressed as mean + SEM (*N* = 3). *P < 0.05 vs DMSO-control; ***P < 0.001 vs DMSO-control (randomized block design ANOVA followed by Dunnett’s post hoc test). (C) The effect of PRDs on PKC protein levels in neonatal mouse cardiac fibroblasts. The cells were exposed to the compounds (at 10 μM concentration) for 24 h and lysed, and total proteins were extracted and analyzed with Western blotting. The results are normalized to the control and expressed as mean + SEM (*N* = 3). Representative images of Western blots are shown in Figure S3. (D) DU145 human prostate cancer cells were exposed to the compounds for 24 h, and cell viability was quantified using the MTT assay. Results are presented as percentage of control (0.1% DMSO) and expressed as mean + SEM (*N* = 3–4).

We evaluated the PRDs for binding to the C1 domains of recombinant full-length human PKCα as described earlier.^13,14,44^ We selected the PKCα isoform to allow maximum comparison with all the previous compounds in the HMI/PYR families, which were tested using PKCα. The compounds were used at a concentration range of 0.2–30 μM (raw data is available in the SI). The results demonstrate that the ester **5b** and the tertiary amide **8a** successfully displaced [20-^3^H]phorbol-12,13-dibutyrate ([^3^H]PDBu) from PKCα in a concentration-dependent manner, with comparable affinity to the reference compound HMI-1a3 (Figure 5A). Although we tested the ester **5b** as a diastereomeric mixture, we expect a negligible difference in the affinity of the stereoisomers as, in our previous work,^13^ enantiomerically pure isophthalates and the corresponding diastereomeric mixture obtained comparable affinity values. Regarding the conformer mixture of **8a**, with the current data, it is not possible to elucidate any specific conformer–affinity relationship. It is interesting to note the difference in the functional group–hydrophobic substituent of the two binding scaffolds: the ester-containing **5b** features the 3-heptyl substituent, while the secondary-amide-containing **8a** features the benzyl substituent. However, our current computational methods cannot elucidate the reason for this functional group–hydrophobic substituent binding preference. Despite exhibiting a behavior similar to compounds **5b** and **8a** in the orientation–distance analysis (Figure 4A), compounds **7a**, **7b**, and **8b** exhibited lower potency for binding to PKC. Compounds **5a** and **7c**, instead, could not displace [^3^H]PDBu in the concentration range tested.

Based on these results, we selected the PRDs **5b** and **8a** for the ERK1/2 (also known as mitogen-activated protein kinases MAPK3/1) phosphorylation and PKC downregulation assays to assess their PKC-mediated activity and properties as PKC activators, respectively.

To investigate whether **5b** and **8a** also modulate PKC in the cellular environment, we performed ERK1/2 phosphorylation assays in living cells. We chose this approach as several PKC isoforms induce ERK1/2 phosphorylation via the Raf-MEK pathway (mitogen-activated protein kinase cascade) to regulate gene expression and modulate apoptosis signaling. We treated neonatal mouse cardiac fibroblasts with compounds HMI-1a3, **5b**, and **8a** at 10 μM for 30 min. The novel PRDs demonstrated PKC-dependent ERK1/2 phosphorylation similar to the reference compound HMI-1a3 (Figure 5B). Pretreatment with the PKC inhibitor Gö6983 blocked the phosphorylation, demonstrating that the effect was mediated by PKC.

Potent C1 domain-binding PKC activators, such as phorbol esters, induce overstimulation and consequent downregulation of the enzyme.^45^ Antal et al.^46^ suggested that the phorbol ester-induced downregulation and consequent loss of function of PKC correlate to the tumor-promoting effects of the compound. We thus investigated the effects of **5b** and **8a** on the expression of PKCα, -δ, and -ϵ, which are the most abundantly expressed PKC isoforms in mouse cardiac fibroblasts.^37^ The compounds had no effect on the expression levels of the isoforms studied (Figure 5C).

To assess the anticancer activity of the compounds, we also evaluated the effect of the PRDs on the viability of DU145 prostate cancer cells using the 3-(4,5-dimethylthiazol-2-yl)-2,5-diphenyltetrazolium bromide (MTT) assay.^47,48^ We tested the two most active (**5b** and **8a**) and two nonactive (**5a** and **7c**) compounds at three different concentrations (0.3, 3.0, and 30 μM), and we compared the results to HMI-1a3, for which the concentration of 0.03 μM was also included. All PRDs were less potent than HMI-1a3 against prostate cancer metabolic activity. The effect on cell viability did not correlate with binding to PKC, which is in line with our previous work showing that the anticancer activity of the HMIs is most often mediated through a non-PKC-dependent route.^35,49,50^ Altogether, PRDs **5b** and **8a** show very similar biological characteristics to the previously established HMI-1a3 in the studies described here.

## Discussion

Targeting PKC for therapeutic purposes is of interest due to the role PKC plays in multiple pathologies, such as cancer, heart disease, diabetes, and Alzheimer’s disease.^2−5^ During the past decades, multiple strategies to mimic the C1 domain-targeted natural agonist DAG for achieving therapeutic modulation of PKC have emerged, with varying results. One cause of failure in targeting could be the lack of mechanistic understanding of the ligand–protein interaction at the lipid–cytosol interface. Such behavior is notoriously hard to probe with traditional computer-aided screening methods, e.g., molecular docking–scoring.

With the development of contemporary *in silico* methods, like MD simulations, this has become feasible. Large-scale MD simulations have gained prominence in drug design processes,^30^ especially in the context of C1 domain-targeting compounds.^7,12,23,25,26^ However, the disadvantage of extensive MD simulation is the need for large computational resources and an elaborate setup. Additional methods, less computationally intensive than protein-containing MD simulations but more accurate than docking–scoring alone, are needed.

An interesting array of interactions worth considering when designing C1 domain-targeted ligands seems to emerge already between the membrane lipids and the ligand candidates alone.^22^ Based on our previous study,^23^ we hypothesized that simulations of behavior and orientation of ligand candidates in the respective membranes could serve as a complementary, or even substitutive, option to predict C1 domain-binding propensity in comparison with the more computationally intensive membrane–ligand–protein binding energy calculations. In this research, our 2-fold goal was to discover a new class of PKC agonists while introducing a refined structure-based drug design workflow by implementing ligand–membrane MD simulations in the design phase.

For this purpose, in addition to the computational docking, we generated force field parameters for the new ligand candidates based on the AMBER GAFF force field and studied their behavior and orientation in SDPS lipid bilayers by modeling the binding assay environment prior to synthesis. Both docking and MD simulations predicted the desired behavior for all seven considered compounds. The positioning of the candidates in the heat maps (Figure 4A) mostly corresponds to that of the control compound HMI-1a3 with minor variations among them. Interestingly, following their synthesis and subsequent biological evaluation, some differences emerged. PRDs **5b** and **8a** exhibited similar binding affinity as HMI-1a3, while PRDs **5a** and **7c** displayed nearly no binding affinity, despite the similar computational behavior. In line with the HMIs, the PRDs demonstrate lower binding affinity to PKC than phorbol esters; this was expected as our main focus was to develop new PKC-targeted ligands with lower lipophilicity than the HMIs with retained, rather than improved, binding affinity.

We thus investigated the simulation trajectories of PRDs **5a**, **5b**, **7c**, and **8a** more rigorously to look for common depicting qualities. In the partial density profiles (Figure 4B), **5b** shows the best alignment with HMI-1a3 for both peaks relative to the whole compound and the hydroxy group. The peak relative to the hydroxy group of **7c** seems to align with the corresponding peak of the potent binder PDBu; however, the partial density peak of the whole molecule displays a rather different profile compared to that of PDBu. The hydroxy group peaks of PRDs **5a** and **8a**, instead, fall in between those of HMI-1a3 and PDBu.

The H-bonding and SASA analyses show the same trend, or lack thereof, as found in the other analyses. All compounds exhibit HMI-1a3-like behavior in both analyses, and variance lies within error estimates. The hydroxy group of **5b** seems marginally more prone to engage H-bonds with water, and the hydroxy group of **8a** possesses slightly higher SASA than the other compounds. Overall, all analyzed candidates returned comparable results with HMI-1a3, and none of them follow the behavior of PYR-1gP_HB–ON_.

In our recent study,^21^ we proposed that a positioning closer to the lipid–water interface and increased SASA of the hydroxy groups can give DAGs an advantage in activating PKC. However, the effect is unlikely to be the sole factor involved. Further, considering that the ultrapotent activator PDBu resides close to the interface (Figure 4B), it also suggests that compounds positioning closer to the interface would possess high affinity. In contrast, all but PRD **5b** position closer to the aqueous interface than HMI-1a3 and, except **8a**, possess lower affinity.

This suggests that our approach cannot serve as a fully substitutive method to more extensive MD simulations. The orientation–distance and density analysis carried out in parallel demonstrate the desired behavior for all candidates, some of which did not bind in our biological assays. Probably, the lack of binding affinity of **5a** and **7c** relates to a more complex regulation system that our model, lacking the C1 domain, could not highlight. While in terms of understanding the binding mode and possible downstream effects on the induced PKC activity, our approach remains incomplete as that cannot be deduced based on the ligand behavior alone. However, the benefit of adding the ligand–membrane simulations into the pipeline is indisputable. The difference to the negative controls PYR-1gP_HB–ON_, HMI-1a3–0, and PYR-1gP-0 are evident in the orientation–distance heat maps and in the SASA and H-bond analyses. Our simulations thus enable the exclusion of initial orientation and positioning of the ligands in the membrane as reasons for the failure of **5a** and **7c**. Thus, for the purpose of predicting which of the designed ligands possess binding prospects and whether or not they should undergo synthesis and biological evaluation, our approach is appropriate.

The regulation system that alters the binding behavior of **5a** and **7c** may still indirectly relate to the ligand orientation, as the presence of a full ligand–protein complex undoubtedly affects the overall positioning/orientation of the ligands. Evidence of such a regulation system was observed previously by Ryckbosch et al.^22^ In their study, the C1B domain–PDBu complex sits deep within the membrane and with a small angle relative to the membrane normal. Instead, C1B domain–bryostatin/prostratin complexes could also occupy a shallower position and with a greater angle. They propose that depth and orientation of the penetration of PKC C1B and other domains can influence the availability of the PKC complex for the substrate proteins and thus control the activation patterns. In light of these observations, it would be especially interesting to study the location and orientation of the PKCδ C1B domain complexed with our new PRDs. However, studying the binding modes of PKC with each ligand represents a separate research avenue requiring significantly more computational resources than our approach. Here, we focused on finding new functioning PKC activators rather than rigorously investigating the differences between binding and nonbinding candidates within a set of promising compounds.

## Conclusions

In this study, we employed a refined five-step approach to a drug design process for finding PKC-targeted agonists. The five steps can be described as follows: (1) we designed a new scaffold to mimic the natural agonist DAG and based this design on the active but highly lipophilic HMIs, (2) we docked a selection of seven PRDs into the PKCδ C1B domain (PDB ID: 1PTR(27)) to assess their correct poses and ligand–protein interactions, (3) using MD simulations, we investigated the orientation and positioning of these PRDs in SDPS bilayers and observed that all seven showed desired HMI-like behavior, and (4) we prepared the ligands with state-of-the-art synthesis methods and (5) characterized their biological activities on PKC in terms of binding affinity, enzyme activation, and enzyme downregulation. Compounds **5b** and **8a** successfully bound PKCα with a comparable affinity to the reference compound HMI-1a3; they both induced PKC-mediated ERK1/2 phosphorylation, and neither of them induced PKC downregulation. At the end of this pipeline, we discovered two new PRDs as promising C1 domain-targeted DAG mimetics that possess lower lipophilicity than the HMIs while retaining HMI-like affinity. Our five-step approach provides a working example of a feasible multistage procedure for drug development, especially in the case of weakly membrane-associated proteins such as PKC.

## Experimental Section

### Design

The structures, clog*P*, and SMILES of the new designed compounds were generated using ChemDraw Professional 22, and the full list is available in the SI.

### Molecular Docking

The compounds were docked using the Schrödinger Maestro molecular modeling environment with the default settings as described below.^51^ The crystal structure of the PKCδ C1B domain (PDB ID: 1PTR(27)) was obtained from the Protein Data Bank^52^ and preprocessed using Protein Preparation Wizard to assign bond orders, add hydrogens, create zero-order bonds to metals, and generate probable ionization and tautomeric states with Epik at pH 7.0 ± 2.0. Missing side chains of Lys234, Arg273, and Glu274 were reconstructed using Prime. The receptor grid was generated with an enclosing box centered on the centroid of the phorbol 13-acetate ligand (exact grid coordinates can be found in the SI).

The ligand candidates were prepared with LigPrep to generate low-energy 3D structures. The default OPLS3e force field was used.^53^ Ionization and tautomeric states were predicted with Epik at pH 7.0 ± 2.0. Up to 32 stereoisomers were generated. The prepared compounds were docked using the Glide XP protocol^54^ and scored according to the proprietary empirical scoring function GlideScore (based on Chemscore^55^). Compound conformations were generated during the docking process with default settings. Epik state penalty correction was included in the docking score to penalize high-energy states. The reward intramolecular hydrogen bonds option was selected to add a reward term to GlideScore for each ligand intramolecular hydrogen bond and to favor the selection of poses with such hydrogen bonds. Up to ten poses per compound were generated. Postdocking minimization was performed to optimize ligand geometries within the default threshold of 0.5 kcal/mol with respect to the original docked pose. As a control, the complexed phorbol 13-acetate was redocked (RMSD < 0.5 Å to X-ray structure) together with the other compounds.

### Molecular Dynamics

For the MD portion of this study, seven systems were constructed, one for each studied new ligand candidate. In the case of candidates containing stereogenic centers (i.e., **5b**, **7b**, and **8b**), the simulations were conducted using the *meso* isomers. The amide analogs **8a** and **8b** were initialized in the *cis*|*cis* conformation of the amide bonds; this conformation is held during the simulations. As in our previous work,^23^ the systems that were simulated were selected to match the conditions of the *in vitro* binding assays.^13,14^ These simulations were composed of ligand candidates embedded in bilayers containing 128 molecules of 1-stearoyl-2-docosahexaenoyl-*sn*-glycero-3-phospho-l-serine (SDPS) solvated in 6400 water molecules with 128 K^+^ counterions. The systems were parametrized using AMBER-compatible force fields; i.e., the lipids were modeled using Lipid17,^56−58^ the ligand molecules, with GAFF,^59^ water, with the TIP3P model,^60^ and ions, with potentials created by Dang et al.^61^ The initial coordinates of the lipid bilayers were constructed using the CHARMM-GUI interface,^62−64^ after which four units of the studied ligand candidate were placed manually into the membrane core with lateral separation using VMD and Gromacs.^65−68^ Partial charges and other parameters for the ligand molecules were derived according to the standard GAFF procedures, using Gaussian16^69^ and Ambertools package Antechamber18.^70,71^ Further details of the parametrization can be found in the SI.

In addition to these simulations, some supplementary systems were also created for control purposes. These models included HMI-1a3 (positive control) and PYR-1gP with the internal hydrogen bond engaged with bias (PYR-1gP_HB–ON_, negative control). We also performed simulations of negative control compounds with no hydroxymethyl group for HMI, PYR, and PRDs (i.e., HMI-1a3-0, PYR-1gP-0, PRD-5a-0, and PRD-5b-0). We also repeated our approach for PDBu to relate the position of our candidates to that of an ultrapotent agonist (Figure S2). These simulations established that, for HMI and PYR, the hydroxymethyl group and its availability is crucial for obtaining the correct orientation in the membrane, while the new compounds can orient correctly even without the hydroxymethyl group. This demonstrates the robustness of the new design choices when compared to previous scaffolds. Further information on the conducted simulations and the structures of the control compounds are available in the SI.

After initialization, all systems were carefully equilibrated and finally simulated freely for 1.6 or 1.7 μs using Gromacs 2020.3.^72^ Before analysis, the first 100 or 200 ns were cut from the trajectory to ensure proper equilibration, so in total, 1.5 μs of each trajectory was used for analysis. For more detailed information on the simulation parameters, see the SI. All analysis was conducted using Gromacs 2020.3/2020.4^72,73^ simulation packages, accompanied by in-house postprocessing algorithms.

### Synthesis Procedures

#### General Information

All reagents, acquired from Fluka, Fluorochem, and Merck/Sigma-Aldrich were used without further purification. The progress of the chemical reactions was monitored by thin-layer chromatography on Silica Gel 60 F254 aluminum sheets or amino-functionalized KP-NH TLC glass plates, visualized under UV light (λ: 254/366 nm) and, when necessary, stained with phosphomolybdic acid (10% w/v in EtOH). Microwave reactions were performed with a Biotage Initiator+ SP Wave Microwave Synthesizer. Flash SiO_2_ (when specified, amino-functionalized NH_2_–SiO_2_) column chromatography was performed with a Biotage Isolera Spektra Systems equipped with prepacked columns. The volume of the eluents is expressed in column volume (CV). ^1^H, ^13^C, and ^19^F NMR spectra, available with assignments in the SI (Supporting NMR Appendix) including ^1^H–^13^C HSQC, ^1^H–^13^C HMBC, and ^1^H–^15^N HMBC 2D NMR spectra, were acquired on a Bruker Ascend 400 MHz - Avance III HD NMR spectrometer and processed with MestReNova 14.2.1 software. Chemical shifts (δ) are reported as parts per million (ppm) relative to the chemical shifts of the residual nondeuterated solvent: CDCl_3_, 7.26 and 77.16 ppm; DMSO-*d*_6_, 2.50 and 39.52, for ^1^H and ^13^C NMR, respectively. For the ^19^F and ^15^N NMR measurements, no F-/N-containing reference compound was utilized; the reference frequencies were applied by default through the solvent lock (^2^H) signal according to IUPAC recommended method and the manufacturer’s protocols. Multiplicities of peaks are represented by s (singlet), d (doublet), t (triplet), q (quartet), quint (quintet), and m (multiplet). In the case of a mixture of conformers (**8a** and **8b**), multiplicities of peaks are represented by ms (multiple singlets referring to the same nucleus/nuclei) and mm (multiple multiplets referring to the same nucleus/nuclei). Visual features of peaks including broad (br) or apparent (app) are also indicated. In ^13^C NMR data, peaks referring to two symmetrical carbons or two different carbons with overlapping signals (2C) are also indicated. Low-resolution mass (MS–APCI) analyses were performed on a MS Advion expression CMS spectrometer equipped with an APCI ion source and an atmospheric solids analysis probe (ASAP). The exact mass and purity (>95%) of all tested compounds were confirmed by LC–MS (HRMS–ESI) analyses with a Waters Acquity UPLC system equipped with an Acquity UPLC BEH C18 column (1.7 μm, 50 mm × 2.1 mm), an Acquity PDA detector, and a Waters Synapt G2 HDMS mass spectrometer via an ESI ion source in positive mode. Mass data are reported for the molecular ions [M + H]^+^. Total ion chromatogram and photodiode array signals, mass spectra, and single mass analyses of all tested compounds are available in the SI (Supporting LC–MS Appendix).

#### Dimethyl 4-(Hydroxymethyl)pyridine-2,6-dicarboxylate (**2**)

A solution of Fe(ClO_4_)_2_·H_2_O (4.65 g, 12.8 mmol) in H_2_O (4.7 mL) and a 30% solution of H_2_O_2_ in H_2_O (8.00 mL, 77.6 mmol) were added dropwise at 0 °C (not exceeding 10 °C) over 1 h to a mixture of dimethylpyridine-2,6-dicarboxylate (2.50 g, 12.8 mmol), MeOH (7.5 mL, 261 mmol, 20.4 equiv), and a 70% solution of HClO_4_ in H_2_O (5.60 mL, 64.9 mmol, 5.1 equiv). The reaction mixture was allowed to warm up slowly to rt, and it was stirred for 3 h. The volatile components were evaporated under reduced pressure, and the pH of the residue was adjusted to 8 with a saturated solution of K_2_CO_3_ in H_2_O. Brown iron(III) salts precipitated and were filtered on Celite. The filtrate was extracted with EtOAc, and the combined organic layers were evaporated under reduced pressure. The residue was purified by flash column chromatography; eluents: *n*-hexane (A), EtOAc/EtOH 3:1 (B); gradient: 12% B, 1 CV; 12–100% B, 10 CV; 100% B, 4 CV. Compound **2** was isolated as a white solid (1.43 g, 6.34 mmol, 49.5% yield). TLC (*n*-heptane/EtOAc/EtOH 4:3:1 v/v): *R*_*f*_ = 0.3. ^1^H NMR (400 MHz, CDCl_3_) δ 8.27 (app t, *J* = 0.9 Hz, 2H), 4.88 (d, *J* = 4.4 Hz, 2H), 3.99 (s, 6H), 2.85 (br s, 1H). ^13^C NMR (101 MHz, CDCl_3_) δ 165.3 (2C), 153.8, 148.3 (2C), 125.4 (2C), 62.9, 53.3 (2C). MS-APCI [M + H]^+^ calcd. for C_10_H_12_NO_5_, 226.1; found, 226.1.

#### Dimethyl 4-[[(Tetrahydro-2*H*-pyran-2-yl)oxy]methyl]pyridine-2,6-dicarboxylate (**3**)

A mixture of **2** (0.700 g, 3.11 mmol), 3,4-dihydro-2*H*-pyran (851 μL, 9.36 mmol, 3 equiv), and pyridinium *p*-toluenesulfonate (39.9 mg, 0.155 mmol, 0.05 equiv) in 1,2-dichloroethane (6.9 mL) was stirred at rt for 6 h. The reaction was quenched by the addition of cold water, extracted with DCM, and washed with a saturated solution of NaHCO_3_ in H_2_O and then with brine. The combined organic layers were evaporated under reduced pressure, and the residue was purified by flash column chromatography; eluents: *n*-heptane (A), EtOAc/EtOH 3:1 (B); gradient: 12% B, 1 CV; 12–75% B, 8 CV. Compound **3** was isolated as a white solid (795 mg, 2.57 mmol, 82.7% yield). TLC (*n*-heptane/EtOAc/EtOH 4:3:1 v/v): *R*_*f*_ = 0.48. ^1^H NMR (400 MHz, CDCl_3_) δ 8.28 (app t, *J* = 0.8 Hz, 2H), 4.92 (dt, *J* = 14.3, 0.9 Hz, 1H), 4.75 (t, *J* = 3.5 Hz, 1H), 4.62 (dt, *J* = 14.3, 0.9 Hz, 1H), 4.01 (s, 6H), 3.89–3.79 (m, 1H), 3.60–3.50 (m, 1H), 1.96–1.81 (m, 1H), 1.85–1.67 (m, 2H), 1.70–1.49 (m, 3H). ^13^C NMR (101 MHz, CDCl_3_) δ 165.3 (2C), 151.3, 148.4 (2C), 126.1 (2C), 98.7, 66.7, 62.4, 53.3 (2C), 30.4, 25.4, 19.2. MS-APCI [M + H]^+^ calcd. for C_15_H_20_NO_5_, 310.1; found, 310.1.

#### Preparation of the Tetranuclear Zinc Cluster for Zn-Catalyzed Transesterifications of Compound **3**

The μ-oxo-tetranuclear zinc cluster Zn_4_(OCOCF_3_)_6_O was prepared by sublimation from zinc trifluoroacetate hydrate following the procedure reported by Iwasaki and co-workers.^40^

#### Di(heptan-3-yl) 4-[[(Tetrahydro-2*H*-pyran-2-yl)oxy]methyl]pyridine-2,6-dicarboxylate (**4b**)

A mixture of **3** (0.100 g, 0.323 mmol), 3-heptanol (111 μL, 0.776 mmol, 2.4 equiv), and Zn_4_(OCOCF_3_)_6_O (4.6 mg, 4.8 μmol, 0.015 equiv) in *i*-Pr_2_O (1 mL) was refluxed for 4 d. The solvent was evaporated under reduced pressure; the excess alcohol was removed by vacuum distillation, and the residue was purified by flash column chromatography; eluents: *n*-heptane (A), EtOAc/acetone 3:1 (B); gradient: 6% B, 1 CV; 6–50% B, 10 CV. Compound **4b** was isolated as a colorless oil (62.8 mg, 0.131 mmol, 40.7% yield). TLC (*n*-heptane/EtOAc/acetone 4:3:1 v/v): *R*_*f*_ = 0.50. ^1^H NMR (400 MHz, CDCl_3_) δ 8.18 (t, *J* = 0.8 Hz, 2H), 5.18–5.06 (m, 2H), 4.91 (dt, *J* = 14.2, 0.9 Hz, 1H), 4.75 (t, *J* = 3.5 Hz, 1H), 4.64 (dt, *J* = 14.2, 0.8 Hz, 1H), 3.99–3.74 (m, 1H), 3.69–3.43 (m, 1H), 1.95–1.85 (m, 1H), 1.84–1.65 (m, 10H), 1.65–1.50 (m, 3H), 1.45–1.27 (m, 8H), 0.96 (t, *J* = 7.4 Hz, 6H), 0.88 (app t, *J* = 7.1 Hz, 6H). ^13^C NMR (101 MHz, CDCl_3_) δ 164.6 (2C), 150.6, 149.4 (2C), 125.5 (2C), 98.6, 77.9 (2C), 66.8, 62.4, 33.3 (2C), 30.5, 27.6 (2C), 27.0 (2C), 25.4, 22.7 (2C), 19.3, 14.1 (2C), 9.8 (2C). MS-APCI [M + H]^+^ calcd. for C_27_H_44_NO_6_, 478.3; found, 478.4.

#### Bis[3-(trifluoromethyl)benzyl] 4-[[(Tetrahydro-2*H*-pyran-2-yl)oxy]methyl]pyridine-2,6-dicarboxylate (**5a**)

##### Method A

A mixture of **3** (0.100 g, 0.323 mmol), 3-(trifluoromethyl)benzyl alcohol (264 μL, 1.88 mmol, 5.8 equiv), and Zn_4_(OCOCF_3_)_6_O (5.6 mg, 6.8 μmol, 0.02 equiv) in *i*-Pr_2_O (1 mL) was refluxed for 72 h. The solvent was evaporated under reduced pressure, and the residue was purified by flash column chromatography; eluents: *n*-heptane (A), EtOAc/acetone 3:1 (B); gradient: 15% B, 1 CV; 17–70% B, 15 CV. Compound **5a** was isolated as a white solid (26 mg, 0.047 mmol, 16% yield).

##### Method B (Neat)

A mixture of **3** (0.030 g, 0.10 mmol) and Zn_4_(OCOCF_3_)_6_O (1.4 mg, 1.5 μmol, 0.015 equiv) in 3-(trifluoromethyl)benzyl alcohol (207 μL, 1.45 mmol, 15 equiv) was microwave irradiated at 80 °C for 6 h under argon atmosphere. Additional Zn_4_(OCOCF_3_)_6_O (1.4 mg, 3.4 μmol, 0.015 equiv) was added, and the mixture was microwave irradiated at 100 °C for 15 h under argon atmosphere. The excess alcohol was removed by vacuum distillation, and the residue was purified by flash column chromatography as in Method A. Compound **5a** was isolated as a white solid (12 mg, 0.024 mmol, 25% yield).

TLC (*n*-heptane/EtOAc/acetone 4:3:1 v/v): *R*_*f*_ = 0.37. ^1^H NMR (400 MHz, CDCl_3_) δ 8.27 (t, *J* = 0.9 Hz, 2H), 7.74 (s, 2H), 7.67 (d, *J* = 7.6 Hz, 2H), 7.59 (d, *J* = 8.0 Hz, 2H), 7.48 (t, *J* = 7.7 Hz, 2H), 5.48 (s, 4H), 4.87 (s, 2H), 2.43 (br s, 1H). ^13^C NMR (101 MHz, CDCl_3_) δ 164.5 (2C), 153.6, 148.4 (2C), 136.5 (2C), 132.0 (app q, *J* = 1.4 Hz, 2C), 131.2 (q, *J* = 32.6 Hz, 2C), 129.3 (2C), 125.6 (2C), 125.6–125.3 (m, sym, 4C), 124.1 (q, *J* = 272.4 Hz, 2C), 67.0 (2C), 62.9. HRMS–ESI (*m*/*z*): [M + H]^+^ calcd. for C_24_H_18_F_6_NO_5_, 514.1089; found, 514.1090.

#### Di(heptan-3-yl) 4-(Hydroxymethyl)pyridine-2,6-dicarboxylate (**5b**)

##### Method A

In the same procedure that was used to obtain **4b**, compound **5b** was isolated as a colorless oil (38 mg, 0.10 mmol, 30% yield).

##### Method B

Dry Dowex 50WX8 (180 mg) was added to a solution of **4b** (69 mg, 0.14 mmol) in MeOH (1 mL). The mixture was stirred at 40 °C for 23 h. The resin was filtered; the solvent was evaporated under reduced pressure, and the residue was purified by flash column chromatography; eluents: *n*-heptane (A), EtOAc/acetone 3:1 (B); gradient: 10% B, 1 CV; 10–41% B, 8 CV. Compound **5b** was isolated as a colorless oil (12 mg, 0.024 mmol, 25% yield).

TLC (*n*-heptane/EtOAc/acetone 12:3:1 v/v): *R*_*f*_ = 0.25. ^1^H NMR (400 MHz, CDCl_3_) δ 8.18 (app t, *J* = 0.9 Hz, 2H), 5.20–5.07 (m, 2H), 4.89 (s, 2H), 2.40 (br s, 1H), 1.81–1.60 (m, 8H), 1.46–1.25 (m, 8H), 0.96 (t, *J* = 7.4 Hz, 6H), 0.88 (app t, *J* = 7.2 Hz, 6H). ^13^C NMR (101 MHz, CDCl_3_) δ 164.6 (2C), 152.9, 149.4 (2C), 124.8 (2C), 78.0 (2C), 63.2, 33.4 (2C), 27.7 (2C), 27.1 (2C), 22.7 (2C), 14.1 (2C), 9.8 (2C). HRMS–ESI (*m*/*z*): [M + H]^+^ calcd. for C_22_H_36_NO_5_, 394.2593; found, 394.2595.

#### 4-[[(Tetrahydro-2*H*-pyran-2-yl)oxy]methyl]-*N*^2^,*N*^6^-bis[3-(trifluoromethyl)benzyl]pyridine-2,6-dicarboxamide (**6a**)

3-(Trifluoromethyl)benzylamine (264 μL, 1.84 mmol, 5.7 equiv) was added to a solution of **3** (0.100 g, 0.323 mmol) in anhydrous MeOH (3 mL) under argon atmosphere. The mixture was microwave irradiated at 120 °C for 15 h and 160 °C for 30 min. The reaction mixture was diluted with EtOAc and shaken with water. The pH of the aqueous layer was adjusted to 3–4 by adding a 1 M solution of KHSO_4_ in H_2_O, and the layers were separated. The aqueous layer was extracted twice with EtOAc; the combined organic layers were washed with brine and dried under reduced pressure, and the residue was purified by flash column chromatography; eluents: *n*-heptane (A), EtOAc (B); gradient: 12% B, 1 CV; 12–100% B, 15 CV. Compound **6a** was isolated as a white solid (0.120 g, 0.202 mmol, 62.4% yield). TLC (*n*-heptane/EtOAc 1:1 v/v): *R*_*f*_ = 0.25. ^1^H NMR (400 MHz, CDCl_3_) δ 8.36 (app t, *J* = 0.9 Hz, 2H), 8.31 (t, *J* = 6.4 Hz, 2H), 7.48 (s, 4H), 7.49–7.44 (m, 2H), 7.41–7.33 (m, 2H), 4.89 (dt, *J* = 14.4, 0.9 Hz, 1H), 4.74 (t, *J* = 3.4 Hz, 1H), 4.63 (d, *J* = 6.4 Hz, 4H), 4.59 (dt, *J* = 14.4, 0.8 Hz, 1H), 3.89–3.79 (m, 1H), 3.60–3.50 (m, 1H), 1.93–1.79 (m, 1H), 1.82–1.64 (m, 2H), 1.67–1.48 (m, 3H). ^13^C NMR (101 MHz, CDCl_3_) δ 163.9 (2C), 152.3, 148.8 (2C), 139.2 (2C), 131.2 (2C), 131.1 (q, *J* = 32.2 Hz, 2C), 129.3 (2C), 124.5 (q, *J* = 3.8 Hz, 2C), 124.4 (q, *J* = 3.8 Hz, 2C), 124.0 (q, *J* = 271.8 Hz, 2C), 123.6 (2C), 98.7, 66.9, 62.3, 43.1 (2C), 30.4, 25.4, 19.2. MS-APCI [M + H]^+^ calcd. for C_29_H_28_F_6_N_3_O_4_, 596.2; found, 596.3.

#### *N*^2^,*N*^6^-Di(heptan-3-yl)-4-[[(tetrahydro-2*H*-pyran-2-yl)oxy]methyl]pyridine-2,6-dicarboxamide (**6b**)

A 60% suspension of NaH in mineral oil (41.5 mg, 1.04 mmol, 3 equiv) was added to an ice-cooled solution of 3-heptanamine (158 μL, 1.07 mmol, 3.1 equiv) in anhydrous THF (1 mL) under argon atmosphere, and the mixture was stirred for 15 min at 0 °C. The resulting suspension was added to a solution of **3** (107 mg, 0.346 mmol) in anhydrous THF (0.5 mL) under argon atmosphere at 0 °C, and the color turned yellow. The mixture was stirred at rt for 1 h, and the color turned to bright orange; then, it was microwave irradiated at 40 °C for 1 h and its color darkened. The reaction was quenched with ice water (***Caution!****Quenching with ice water is not advised for bigger-scale reactions due to the exothermic evolution of H_2_*) and transferred to a separatory funnel. The pH was adjusted to 3–4 by adding a 1 M solution of KHSO_4_ in H_2_O, and the aqueous layer was extracted with EtOAc. The combined organic layers were washed with brine and dried under reduced pressure, and the residue was purified by flash column chromatography; eluents: *n*-heptane (A), EtOAc (B); gradient: 12% B, 1 CV; 12–100% B, 15 CV. Compound **6b** was isolated as a white powder (58 mg, 0.12 mmol, 36% yield). TLC (*n*-heptane/EtOAc 1:1 v/v): *R*_*f*_ = 0.4. ^1^H NMR (400 MHz, CDCl_3_) δ 8.33 (app t, *J* = 0.9 Hz, 2H), 7.41 (d, *J* = 8.9 Hz, 2H), 4.90 (dt, *J* = 14.2, 0.9 Hz, 1H), 4.75 (t, *J* = 3.4 Hz, 1H), 4.61 (dt, *J* = 14.3, 0.8 Hz, 1H), 4.14–3.98 (m, 2H), 3.92–3.78 (m, 1H), 3.63–3.47 (m, 1H), 1.96–1.83 (m, 1H), 1.83–1.58 (m, 7H), 1.62–1.45 (m, 6H), 1.44–1.27 (m, 8H), 0.97 (td, *J* = 7.4, 1.2 Hz, 6H), 0.94–0.80 (m, 6H). ^13^C NMR (101 MHz, CDCl_3_) δ 163.3 (2C), 152.0, 149.3 (2C), 123.0 (2C), 98.6, 67.0, 62.1, 51.0 (2C), 34.4 (2C), 30.4, 28.2 (2C), 28.1 (d, *J* = 1.1 Hz, 2C), 25.5, 22.8 (d, *J* = 1.5 Hz, 2C), 19.1, 14.2 (2C), 10.3 (2C). MS-APCI [M + H]^+^ calcd. for C_27_H_46_N_3_O_4_, 476.3; found, 476.2.

#### 4-(Hydroxymethyl)-*N*^2^,*N*^6^-bis[3-(trifluoromethyl)benzyl]pyridine-2,6-dicarboxamide (**7a**)

3-(Trifluoromethyl)benzylamine (0.420 mL, 2.93 mmol, 6.6 equiv) was added to a solution of **2** (0.100 g, 0.444 mmol) in anhydrous MeOH (4 mL) under argon atmosphere. The mixture was microwave irradiated at 120 °C for 12 h. The reaction mixture was diluted with EtOAc and shaken with water. The pH of the aqueous layer was adjusted to 3–4 by adding a 1 M solution of KHSO_4_ in H_2_O, and the layers were separated. The aqueous layer was extracted twice with EtOAc; the combined organic layers were washed with brine and dried under reduced pressure, and the residue was purified by flash column chromatography; eluents: *n*-heptane (A), EtOAc (B); gradient: 12% B, 1 CV; 12–100% B, 15 CV. Compound **7a** was isolated as a white solid (147 mg, 0.287 mmol, 64.7% yield). TLC (*n*-heptane/EtOAc/EtOH 4:3:1 v/v): *R*_*f*_ = 0.45. ^1^H NMR (400 MHz, CDCl_3_) δ 8.42 (t, *J* = 6.4 Hz, 2H), 8.32 (app t, *J* = 0.9 Hz, 2H), 7.47–7.39 (m, 6H), 7.33 (t, *J* = 7.8 Hz, 2H), 4.75 (d, *J* = 5.5 Hz, 2H), 4.58 (d, *J* = 6.3 Hz, 4H), 3.57 (t, *J* = 5.9 Hz, 1H). ^1^H NMR (400 MHz, DMSO-*d*_6_) δ 9.93 (t, *J* = 6.4 Hz, 2H), 8.22 (app t, *J* = 0.9 Hz, 2H), 7.71–7.66 (m, 2H), 7.66–7.63 (m, 2H), 7.63–7.61 (m, 2H), 7.60–7.55 (m, 2H), 5.66 (t, *J* = 5.8 Hz, 1H), 4.71 (t, *J* = 5.5 Hz, 6H). ^13^C NMR (101 MHz, CDCl_3_) δ 164.2 (2C), 154.9, 148.6 (2C), 139.1 (2C), 131.1 (2C), 131.0 (q, *J* = 32.1 Hz, 2C), 129.3 (2C), 124.5 (q, *J* = 3.8 Hz, 2C), 124.3 (q, *J* = 3.8 Hz, 2C), 124.0 (q, *J* = 272.3 Hz, 2C), 122.9 (2C), 63.0, 43.1 (2C). HRMS–ESI (*m*/*z*): [M + H]^+^ calcd. for C_24_H_20_F_6_N_3_O_3_, 512.1409; found, 512.1411.

#### *N*^2^,*N*^6^-Di(heptan-3-yl)-4-(hydroxymethyl)pyridine-2,6-dicarboxamide (**7b**)

Montmorillonite K10 (30 mg) was added to a solution of **6b** (18 mg, 0.037 mmol) in anhydrous MeOH (1 mL), and the mixture was microwave irradiated at 55 °C for 2 h. The clay was removed by vacuum filtration on a sintered funnel; the solvent was removed under reduced pressure, and the residue was purified by flash column chromatography; eluents: *n*-heptane (A), EtOAc/EtOH 3:1 (B); gradient: 10% B, 1 CV; 10–62% B, 7 CV. Compound **7b** was isolated as a white solid (13 mg, 0.031 mmol, 84% yield). TLC (*n*-heptane/EtOAc/EtOH 4:3:1 v/v): *R*_*f*_ = 0.5. ^1^H NMR (400 MHz, CDCl_3_) δ 8.38 (app t, *J* = 0.8 Hz, 2H), 7.45 (d, *J* = 9.3 Hz, 2H), 4.85 (s, 2H), 4.11–3.98 (m, 2H), 3.86 (s, 1H), 1.76–1.58 (m, 4H), 1.61–1.43 (m, 4H), 1.43–1.26 (m, 8H), 0.95 (t, *J* = 7.4 Hz, 6H), 0.88 (app t, *J* = 6.5 Hz, 6H). ^13^C NMR (101 MHz, CDCl_3_) δ ^13^C NMR (101 MHz, CDCl_3_) δ 163.5 (2C), 155.0, 149.2 (2C), 122.6 (2C), 63.3, 51.0 (2C), 34.4 (2C), 28.2 (2C), 28.0 (d, *J* = 1.5 Hz, 2C), 22.8 (d, *J* = 1.6 Hz, 2C), 14.1 (2C), 10.2 (2C). HRMS–ESI (*m*/*z*): [M + H]^+^ calcd. for C_22_H_38_N_3_O_3_, 392.2913; found, 392.2913.

#### 4-(Hydroxymethyl)-*N*^2^,*N*^6^-dioctylpyridine-2,6-dicarboxamide (**7c**)

1-Octylamine (243 μL, 1.465 mmol, 6.6 equiv) was added to a solution of **2** (0.050 g, 0.222 mmol) in anhydrous MeOH (2.3 mL) under argon atmosphere. The mixture was microwave irradiated at 120 °C for 6 h. The reaction mixture was diluted with EtOAc and shaken with water. The pH of the aqueous layer was adjusted to 3–4 by adding a 1 M solution of KHSO_4_ in H_2_O, and the layers were separated. The aqueous layer was extracted twice with EtOAc; the combined organic layers were washed with brine and dried under reduced pressure, and the residue was purified by flash column chromatography; eluents: *n*-heptane (A), EtOAc/EtOH 3:1 (B); gradient: 12% B, 1 CV; 12–47% B, 9 CV. Compound **7c** was isolated as a white solid (74 mg, 0.18 mmol, 80% yield). TLC (*n*-heptane/EtOAc/EtOH 4:3:1 v/v): *R*_*f*_ = 0.42. ^1^H NMR (400 MHz, CDCl_3_) δ 8.33 (app t, *J* = 0.9 Hz, 2H), 7.82 (t, *J* = 6.1 Hz, 2H), 4.82 (app d, *J* = 4.4 Hz, 2H), 3.91 (br s, 1H), 3.55–3.32 (m, 4H), 1.72–1.55 (m, 4H), 1.43–1.20 (m, 20H), 0.86 (app t, *J* = 7.1, 6.7 Hz, 6H). ^13^C NMR (101 MHz, CDCl_3_) δ 163.9 (2C), 154.9, 149.1 (2C), 122.5 (2C), 63.3, 39.9 (2C), 31.9 (2C), 29.8 (2C), 29.43 (2C), 29.37 (2C), 27.2 (2C), 22.8 (2C), 14.2 (2C). HRMS–ESI (*m*/*z*): [M + H]^+^ calcd. for C_24_H_42_N_3_O_3_, 420.3226; found, 420.3227.

#### 4-(Hydroxymethyl)-*N*^2^,*N*^6^-dimethyl-*N*^2^,*N*^6^-bis[3-(trifluoromethyl)benzyl]pyridine-2,6-dicarboxamide (**8a**)

A 60% suspension of NaH in mineral oil (30.2 mg, 0.756 mmol, 5 equiv) was added to a solution of **6a** (0.90 g, 0.15 mmol) in anhydrous THF (2 mL) at 0 °C. The mixture was stirred at rt for 1 h under argon atmosphere. A white precipitate formed. Iodomethane (188 μL, 3.02 mmol, 20 equiv) was added in one portion, and the mixture was stirred overnight. The reaction mixture was diluted with EtOAc and shaken with water made alkaline with a saturated solution of K_2_CO_3_ in H_2_O. The aqueous layer was extracted twice with EtOAc; the organic layers were combined, and the solvent was removed under reduced pressure. The residue was dissolved in anhydrous MeOH (1 mL). Montmorillonite K10 (25 mg) was added, and the mixture was microwave irradiated for 8 h at 55 °C. The clay was removed by vacuum filtration on a sintered funnel; the solvent was removed under reduced pressure, and the residue was purified by flash column chromatography; eluents: *n*-heptane (A), EtOAc/EtOH 3:1 (B); gradient: 13% B, 1 CV; 13–100% B, 15 CV; 100% B, 5 CV. Compound **8a** was isolated as an opalescent viscous liquid (59 mg, 0.11 mmol, 73% yield). TLC (*n*-heptane/EtOAc/acetone 4:3:1 v/v): *R*_*f*_ = 0.45. Mixture of conformers: ^1^H NMR (400 MHz, CDCl_3_) δ 7.82–7.67 (mm, 2H), 7.62–7.28 (mm, 8H), 4.79–4.68 (mm, 2H), 4.84–4.47 (ms, 4H), 3.43–3.25 (mm, 1H), 3.08–2.79 (ms, 6H). ^1^H NMR (400 MHz, DMSO-*d*_6_) δ 7.70–7.59 (mm, 2H), 7.70–7.55 (mm, 4H), 7.70–7.44 (mm, 4H), 5.65–5.56 (mm, 1H), 4.83–4.43 (ms, 4H), 4.69–4.57 (mm, 2H), 2.95–2.63 (ms, 6H). ^1^H NMR (400 MHz, DMSO-*d*_6_, 100 °C) δ 7.72–7.48 (m, 10H), 5.29 (br s, 1H), 4.73 (br s, 4H), 4.64 (s, 2H), 2.92 (br s, 6H). Mixture of conformers: ^13^C NMR (101 MHz, CDCl_3_) δ 168.9–168.6 (ms, 2C), 154.4–153.9 (ms), 152.8–152.5 (ms, 2C), 138.0–137.6 (ms, 2C), 131.8–130.7 (mm, 2C), 129.4 (2C), 129.4–129.1 (mm, 2C), 125.1–124.5 (mm, 2C), 124.7–124.1 (mm, 2C), 128.4–119.9 (mm, 2C), 122.5–121.9 (ms, 2C), 63.0–62.8 (ms), 54.9–50.8 (ms, 2C), 37.3–33.4 (ms, 2C). ^13^C NMR (101 MHz, DMSO-*d*_6_) δ 168.2–167.9 (ms, 2C), 155.2–154.8 (ms), 152.7–152.5 (ms, 2C), 138.8–138.3 (ms, 2C), 131.7–131.2 (ms, 2C), 129.8–129.3 (ms, 2C), 129.7–128.7 (mm, 2C), 128.4–119.7 (mm, 2C), 124.6–123.8 (mm, 4C), 121.2–120.8 (ms, 2C), 61.4–61.1 (ms), 53.3–49.5 (ms, 2C), 36.8–32.6 (ms, 2C). ^13^C NMR (101 MHz, DMSO-*d*_6_, 100 °C) δ 167.5 (2C), 154.2, 152.4 (2C), 138.2 (2C), 130.9 (2C), 129.1 (q, *J* = 31.7 Hz, 2C), 128.9 (2C), 123.6 (q, *J* = 272.4 Hz, 2C), 124.1–122.9 (m, 4C), 120.5 (2C), 61.1, 53.5–48.8 (ms, 2C), 36.6-31.8 (ms, 2C). HRMS–ESI (*m*/*z*): [M + H]^+^ calcd. for C_26_H_24_F_6_N_3_O_3_, 540.1722; found, 540.1722.

#### *N*^2^,*N*^6^-Di(heptan-3-yl)-4-(hydroxymethyl)-*N*^2^,*N*^6^-dimethylpyridine-2,6-dicarboxamide (**8b**)

A 60% suspension of NaH in mineral oil (0.010 g, 0.26 mmol, 5 equiv) was added to a solution of **6b** (25 mg, 0.052 mmol) in anhydrous THF (0.75 mL) at 0 °C. The mixture was stirred at rt for 1 h under argon atmosphere. A yellow precipitate formed. Iodomethane (65 μL, 1.0 mmol, 20 equiv) was added in one portion, and the mixture was stirred overnight. The reaction mixture was diluted with EtOAc and shaken with water made alkaline with a saturated solution of K_2_CO_3_ in H_2_O. The aqueous layer was extracted two additional times with EtOAc; the organic layers were combined, and the solvent was removed under reduced pressure. The residue was dissolved in anhydrous MeOH (1 mL). Montmorillonite K10 (25 mg) was added, and the mixture was microwave irradiated for 10 h at 55 °C. The clay was removed by vacuum filtration on a sintered funnel; the solvent was removed under reduced pressure, and the residue was purified by flash column chromatography; eluents: *n*-heptane (A), EtOAc/EtOH 3:1 (B); gradient: 10% B, 1 CV; 10–65% B, 9 CV. Compound **8b** was isolated as an opalescent viscous liquid (17 mg, 0.39 mmol, 76% yield). TLC (*n*-heptane/EtOAc/EtOH 4:3:1 v/v): *R*_*f*_ = 0.42. Mixture of conformers: ^1^H NMR (400 MHz, CDCl_3_) δ 7.60–7.45 (mm, 2H), 4.75–4.66 (mm, 2H), 4.66–3.26 (mm, 3H), 2.93–2.63 (ms, 6H), 1.62–1.37 (mm, 4H), 1.61–1.25 (mm, 4H), 1.40–1.06 (mm, 8H), 0.97–0.71 (mm, 12H). ^1^H NMR (400 MHz, DMSO-*d*_6_) δ 7.52–7.30 (mm, 2H), 5.59 (br s, 1H), 4.68–4.54 (mm, 2H), 4.54–3.28 (mm, 2H), 2.79–2.58 (ms, 6H), 1.58–1.41 (mm, 4H), 1.59–1.30 (mm, 4H), 1.32–1.03 (mm, 8H), 0.90–0.67 (mm, 12H). ^1^H NMR (400 MHz, DMSO-*d*_6_, 100 °C) δ 7.55–7.32 (m, 2H), 5.25 (br s, 1H), 4.62 (s, 2H), 4.55–3.38 (mm, 2H), 2.85–2.64 (mm, 6H), 1.61–1.46 (m, 4H), 1.61–1.30 (m, 4H), 1.44–1.08 (m, 8H), 0.96–0.69 (m, 12H). Mixture of conformers: ^13^C NMR (101 MHz, CDCl_3_) δ 170.3–169.5 (ms, 2C), 154.4–153.3 (ms, 3C), 121.2–120.6 (ms, 2C), 63.1–62.8 (ms), 60.0–54.6 (ms, 2C), 32.8–31.6 (ms, 2C), 28.6–28.2 (ms, 2C), 30.4–25.8 (ms, 2C), 25.4–25.0 (ms, 2C), 22.8–22.6 (ms, 2C), 14.3–13.9 (ms, 2C), 11.0–10.6 (ms, 2C). ^13^C NMR (101 MHz, DMSO-*d*_6_) δ 169.2–168.8 (ms, 2C), 154.7–153.8 (ms, 3C), 120.5–119.2 (ms, 2C), 61.5–61.2 (ms), 59.0–53.4 (ms, 2C), 32.0–30.7 (ms, 2C), 28.1–27.7 (ms, 2C), 29.8–25.0 (ms, 2C), 24.6–24.3 (ms, 2C), 22.2–21.9 (ms, 2C), 14.1–13.8 (ms, 2C), 10.8–10.5 (ms, 2C). ^13^C NMR (101 MHz, DMSO-*d*_6_, 100 °C) δ 168.4 (2C), 153.7 (3C), 120.2–118.8 (ms, 2C), 61.1, 58.6–53.4 (ms, 2C), 31.6–30.2 (ms, 2C), 27.4 (2C), 29.7–24.5 (ms, 2C), 24.0 (2C), 21.3 (2C), 13.0 (2C), 9.9 (2C). HRMS–ESI (*m*/*z*): [M + H]^+^ calcd. for C_24_H_42_N_3_O_3_, 420.3226; found, 420.3228.

### Biological Evaluation

The biological activity of the synthesized compounds was assessed by a radioligand displacement assay using [^3^H]PDBu to evaluate the ability of the candidates to bind the C1 domain of PKCα. In addition, pERK1/2 phosphorylation assays were performed in neonatal mouse cardiac fibroblasts to investigate the effect of the compounds on PKC activation. The best candidates were checked for PKC downregulation on PKCα, -δ, and -ϵ to ensure therapeutic potential. Finally, the effect of the compounds on DU145 prostate cancer cell viability was analyzed using the MTT assay. In these assays, control refers to exposure to the vehicle (0.1% DMSO), and *N* refers to the number of independent experiments. Each experiment was repeated at least three times with two or more parallel samples that were averaged to produce *N* = 1. More detailed descriptions of these methods can be found in the SI. Statistical analyses for the results of these experiments were conducted with GraphPad Prism version 7.0 for Windows^74^ or IBM SPSS Statistics 28 software. Differences at the level of *P* < 0.05 were considered statistically significant.

## Supporting Information Available

Supporting results: MD simulations of control compounds and Western blotting; supporting experimental section: molecular docking, molecular dynamics, synthesis, and biological evaluation and structure–activity relationship analysis; supporting NMR appendix: 1D (^1^H, ^13^C, and ^19^F) and 2D (^1^H–^13^C HSQC, ^1^H–^13^C HMBC, and ^1^H–^15^N HMBC) spectra of all synthesized compounds; supporting LC–MS appendix: total ion chromatogram and photodiode array signals, mass spectra, and single mass analyses of all tested compounds (PDF)
Docking scores and SMILES of all tested compounds (CSV)
Atomic coordinates (*.gro) and required topology files (*.itp) for molecules used in the simulations (ZIP)
Biological assays (raw data) (XLSX)

## Abbreviations Used

APCI: atmospheric pressure chemical ionization
aPKC: atypical protein kinase C
app: apparent
ASAP: atmospheric solids analysis probe
br: broad
COM: center of mass
cPKC: classical protein kinase C
CV: column volume
DAG: 1,2-diacyl-*sn*-glycerol
HMI: (5-hydroxymethyl) isophthalate
mm: multiple multiplets
ms: multiple singlets
MTT: 3-(4,5-dimethylthiazol-2-yl)-2,5-diphenyltetrazolium bromide
nPKC: novel protein kinase C
OH: hydroxy group
OO/NO: ester/amide groups
PDBu: phorbol-12,13-dibutyrate
PRD: pyridine
PYR: pyrimidine
SASA: solvent accessible surface area
SDPS: 1-stearoyl-2-docosahexaenoyl-*sn*-glycero-3-phospho-l-serine
