# Supplementary material for: Expanding the Paradigm of Structure-Based Drug Design: Molecular Dynamics Simulations Support the Development of New Pyridine-Based Protein Kinase C-Targeted Agonists
Source: J Med Chem. 2023 Apr 3;66(7):4588–602. doi: 10.1021/acs.jmedchem.2c01448 (PMC10108360; doi:10.1021/acs.jmedchem.2c01448)

Supporting Information
Expanding the Paradigm of Structure-Based Drug Design: Molecular Dynamics Simulations Support the Development of New Pyridine-Based Protein Kinase C–Targeted Agonists

Saara Lautala,[†,‖] Riccardo Provenzani,[*,‡,‖] Ilari Tarvainen,[¶] Katia Sirna,[‡] S. Tuuli Karhu,[¶] Evgeni Grazhdankin,[‡] Antti K. Lehtinen,[‡] Hanan Sa'd,[†,§] Artturi Koivuniemi,[†] Henri Xhaard,[‡] Raimo K. Tuominen,[¶] Virpi Talman,[¶] Alex Bunker,[†] and Jari Yli-Kauhaluoma[‡]

†*Drug Research Program, Division of Pharmaceutical Biosciences, P.O. Box 56 (Viikinkaari 5 E), FI-00014, University of Helsinki, Finland*

‡*Drug Research Program, Division of Pharmaceutical Chemistry and Technology, P.O. Box 56 (Viikinkaari 5 E), FI-00014, University of Helsinki, Finland*

¶*Drug Research Program, Division of Pharmacology and Pharmacotherapy, P.O. Box 56 (Viikinkaari 5 E), FI-00014, University of Helsinki, Finland*

§*School of Pharmacy, The University of Jordan, Queen Rania St., 11942, Amman, Jordan*

‖*Contributed equally to this work*

E-mail: riccardo.provenzani@helsinki.fi

Contents

Supporting Results **S3**

Molecular Dynamics: Control Compounds S3

Western Blotting S6

Supporting Experimental Section **S6**

Molecular Docking S6

Molecular Dynamics S7

Ligand Parameterization S7

Dihedral Bias Potential on PYR-1gP S8

Simulation Parameters S9

Control Compounds S10

Synthesis Procedure S10

3-Heptanamine (S2) S10

Biological Evaluation and Structure–Activity Relationship Analysis S11

Cell Culture S11

Displacement Assay S11

Western Blotting S12

ERK1/2 phosphorylation assay S13

Cell Viability Assay S14

Ethics S14

References **S15**

Supporting NMR Appendix **S18**

Supporting LC–MS Appendix **S134**

Supporting Results

Molecular Dynamics: Control Compounds

To examine the effect of the key hydroxymethyl group, and thus the effect of the intramolecular hydrogen bond proposed to form in PYR-1gP,[S1] simulations inspecting the orientation in the membrane of four negative-control DAG mimetics PRD-5a-0, PRD-5b-0, HMI-1a3-0, and PYR-1gP-0 (Figure S1) were also performed. These compounds correspond to PRDs **5a** and **5b**, HMI-1a3 and PYR-1gP, respectively; however, they lack the hydroxymethyl group needed for binding the target. As a positive control, the ultrapotent agonist PDBu was also simulated.

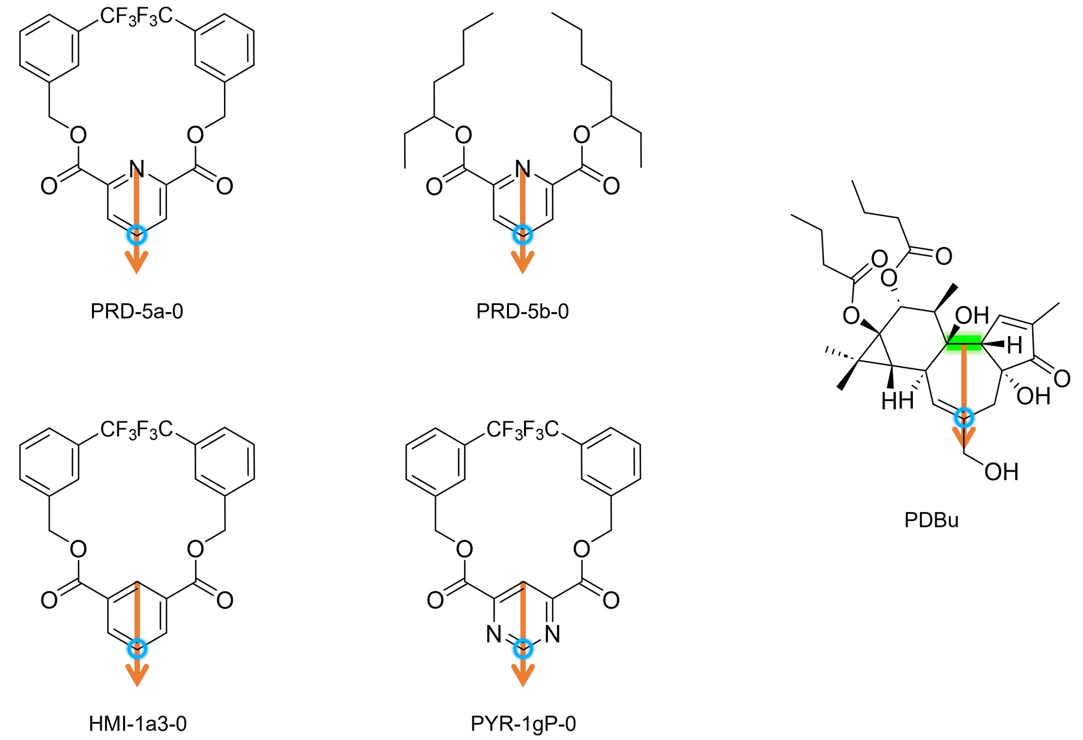

Figure S1: Molecular structures of the four proposed negative-control DAG mimetics PRD-5a-0, PRD-5b-0, HMI-1a3-0, PYR-1gP-0, and the ultrapotent agonist PDBu. The aromatic carbon that would normally bear the hydroxymethyl group of PRDs, HMI, and PYR, and the corresponding carbon of PDBu are highlighted with a blue circle. The axis vectors for each compound are shown in orange. The cyclic core carbons of PDBu, with the center of mass from where the axis vector originates, are highlighted in green.

Orientation–distance matrices were constructed for all the aforementioned compounds (Figure S2). Compounds HMI-1a3-0 and PYR-1gP-0 both occupied the PYR-1gP-like incorrect orientation, further confirming the importance of the hydroxymethyl group to enable the correct orientation of these ligands. Results on PRD-5a-0 and PRD-5b-0 show that the new steps in design for the newest scaffold themselves already guide the orientation of the ligand candidates to the correct orientation, even without the influence of the hydroxymethyl group. The orientation–distance analysis of PDBu was slightly ambiguous in comparison to that of the other compounds due to the different central cyclic cores. The axis vector of PDBu was defined as a vector through the center of mass of the cyclic core carbons highlighted in green and the carbon bound to the hydroxymethyl group highlighted in blue (Figure S2). As visible from the analysis, the cyclic core of PDBu sits almost perpendicular to the membrane normal (parallel to the membrane plane), thus information about its orientation is not directly comparable to our compounds. Information about positioning through partial density analysis could however be compared and is applied in Figure 4B in the main text.

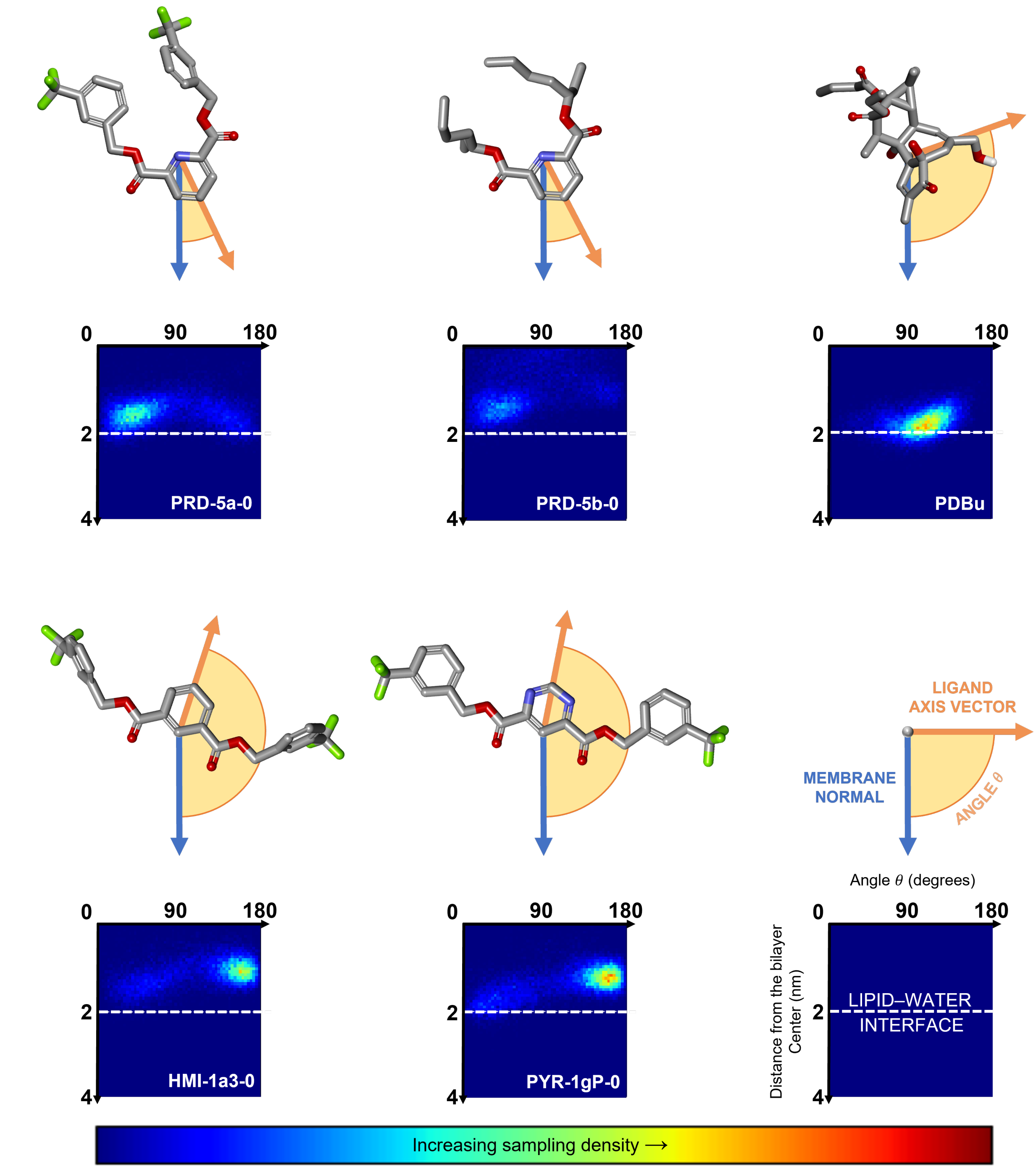

Figure S2: Orientation–distance population heat maps for PRD-5a-0, PRD-5b-0, HMI-1a3-0, PYR-1gP-0, and PDBu with a visual state from their simulation trajectories. Atom color code: carbon is colored light gray, oxygen red, nitrogen blue, fluorine lime, and hydrogen white.

[Back ↑]

Wester Blotting

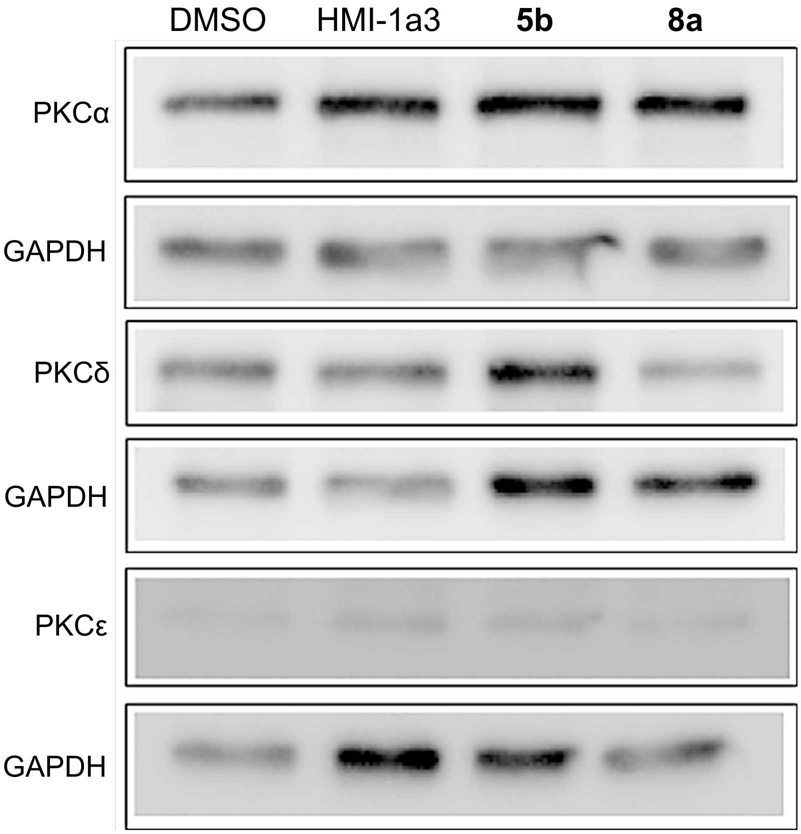

Figure S3: Representative Western blot images for PKCα, -δ, and -ϵ. Glyceraldehyde 3-phosphate dehydrogenase (GAPDH) was used as the loading control and it is shown for each membrane below the corresponding PKC blot.

Supporting Experimental Section

Molecular Docking

The molecular docking was performed in Schrödinger Maestro (Release 2019-4) molecular modeling environment as described in the main text. Input parameters are listed in Table S1.

Table S1: The parameters used for docking in Schrödinger software.

Stage	Parameter	Value
LigPrep	FORCE_FIELD	16
	EPIK	Yes
	DETERMINE_CHIRALITIES	No
	IGNORE_CHIRALITIES	No
	NUM_STEREOISOMERS	32
Receptor Grid Generation	GRID_CENTER	10.644409749235544, 26.62356016485155, 24.24789054093784
	INNERBOX	10, 10, 10
	OUTERBOX	23.76735948185505, 23.76735948185505, 23.76735948185505
Glide Docking	POSES_PER_LIG	10
	POSTDOCK_XP_DELE	0.5
	PRECISION	XP
	REWARD_INTRA_HBONDS	True

Molecular Dynamics

Ligand Parameterization

Initial structures of the ligand candidates were created with Avogadro[S2] in two initial geometries, linear and arms-up as presented in Figure S4. Ligand parameterization was performed according to the GAFF-force field procedure[S3] using Gaussian16[S4] together with the Ambertools package[S5] in Amber18.[S6] Both of these geometries were optimized at HF/6-31G* level,[S7] and the geometry that reached the lowest final energy was selected for charge derivation (data not shown, available upon request). The partial charges were calculated for the respective geometries at the HF/6-31G* level, with the self-consistent field set to default option Tight (SCF=Tight). The electrostatic potential around the ligand candidates was evaluated using the Merz-Singh-Kollman method,[S8] with 10 layers and 17 gridpoints per unit area. Antechamber18 was utilized to assign the partial charges according to the RESP

procedure[S9] and atomtypes according to the GAFF force field.[S3]

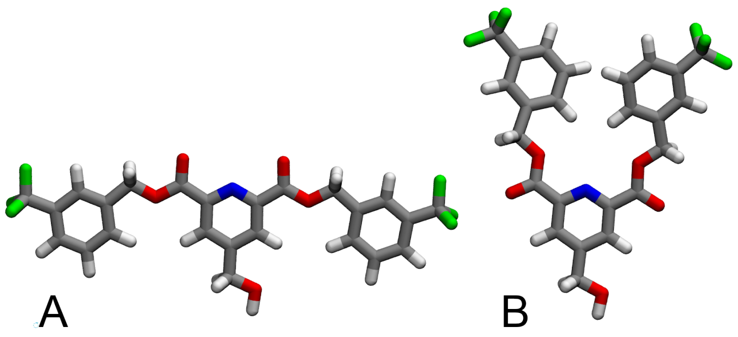

Figure S4: Ligand candidate **5a** presented in initial geometries for parameterization. A) Linear geometry of **5a**. B) Arms-up initial geometry of **5a**. Atom color code: carbon is colored gray, oxygen red, nitrogen blue, fluorine lime, and hydrogen white.

Dihedral Bias Potential on PYR-1gP

Simulations of PYR-1gP with the initial parameters assigned to PYR-1gP in GAFF by antechamber showed different behavior than those conducted with OPLS-AA (Figure S5 A).[S1] Upon inspection, it became clear that the GAFF parameters do not replicate the internal hydrogen bond formation. For this reason, we examined the behavior and energies of PYR-1gP dihedrals N-C-C-O and C-C-O-H with the current quantum theory (QM) level as carried out in our previous study.[S1] The energy profiles derived from HF/6-31G* QM calculations for both dihedrals were similar to those previously derived at b3lyp/aug-cc-pVTZ level (data not shown, available upon request).[S1] This presents a discrepancy in the parameters Ambertools produces and the preferred conformation indicated by the QM calculations. As reparameterization is known to be laborious, and such structure is not present in the new scaffold, we decided to manually fix the hydrogen bond in the ON position using a bias potential in PYR-1gP to see if the behavior observed in OPLS-AA is reproduced when the bond is ON. This was the case, further establishing that when the internal hydrogen bond forms, the orientation of PYR-1gP changes.

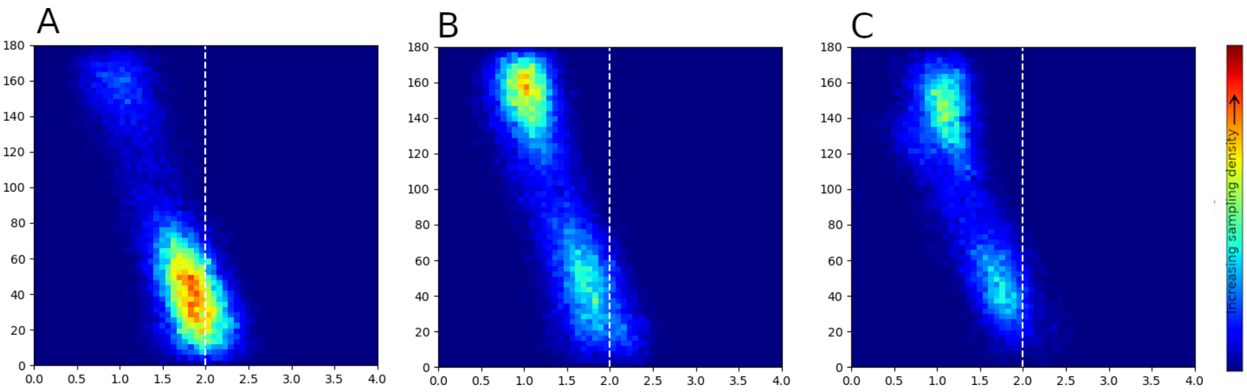

Figure S5: Orientation–distance matrix for different parameterizations of PYR-1gP. In A) the behavior resulting in GAFF default parameters is shown, B) shows the behavior with the intramolecular hydrogen bond biased ON and C) shows the behavior of PYR-1gP in previous OPLS-AA simulation.[S1] Panel C reprinted with permission from S. Lautala et al., J. Chem. Inf. Model. 2020, 60, 11, 5624–5633, Copyright 2020 American Chemical Society.

Simulation Parameters

Simulations were performed with GROMACS simulation engine version 2020.3.[S10–S12] The integrator for equations of motion was the leapfrog algorithm with 2 fs timestep. Periodic boundary conditions were implemented in all directions. Lengths of bonds to hydrogen atoms were constrained using the LINCS algorithm.[S13] Non-bonded interactions were handled with the Verlet cut-off scheme,[S14] with the cut-off radius for Lennard-Jones interactions at 1.0 nm. Particle mesh Ewald method (PME)[S15] was used for electrostatic interactions with a short-range component cut-off of 1.0 nm, interpolation order of 6 and grid spacing of 0.1 nm. The simulation temperature was set to physiological temperature (310 K) using the Nosé-Hoover thermostat[S16,S17] using a coupling constant of 0.4 ps. Lipids and ligand molecules were coupled to the thermostat together, separate from water and ions. Parrinello-Rahman barostat[S18,S19] with semi-isotropic (xy and z) coupling was used to set the pressure to atmospheric pressure (1 bar) with a time constant of 10 ps and compressibility of 4.5×10^{-5} bar^{-1}.

[Back ↑]

Control Compounds

Synthesis Procedure

Scheme

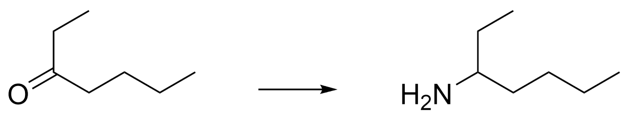

S1 S2

Scheme S1: Synthesis of 3-heptanamine. Conditions: $CH_3CO_2NH_4$, MeOH, rt, 90 min + $NaBH_3CN$, rt, 60 h, (78%).

3-Heptanamine (S2)

3-Heptanamine was prepared from 3-heptanone following the procedure reported by Kapoor and coworkers.[S20] Ammonium acetate (14 g, 18 mmol, 10 equiv) was added to a solution of 3-heptanone (2.4 mL, 18 mmol) in anhydrous methanol (40.5 mL) and the reaction mixture was stirred at rt for 90 min under argon atmosphere. $NaBH_3CN$ (1.1 g, 18 mmol) was added in portions and the mixture was stirred for at rt for 60 h. The mixture was diluted with a 1 M NaOH solution in H_2O (40 mL), stirred for 30 min, and extracted with Et_2O. The combined organic layers were extracted with a 1 M solution of HCl in H_2O. The combined aqueous layers were treated with aqueous NH_4OH solution until basic, followed by extraction with CH_2Cl_2. The combined organic layers were dried over Na_2SO_4 and evaporated under reduced pressure at 20 °C (warning: the boiling point of the product ranges between 100 °C and 110 °C at 1 atm). The crude product was isolated as a pale-yellow oil (1.6 g, ~14 mmol, ~78% yield) and was used without further purification. TLC (*n*-heptane:EtOAc 1:1 v/v + 1% TEA): $R_f = 0.2$. ^{1}H NMR (400 MHz, $CDCl_3$) δ 2.62–2.51 (m, 1H), 1.71 (br s, 2H), 1.52–1.38 (m, 3H), 1.42–1.21 (m, 5H), 0.91 (t, $J = 7.4$ Hz, 3H), 0.91–0.87 (m, 3H). ^{13}C NMR (101 MHz, $CDCl_3$) δ 52.7, 37.1, 30.5, 28.4, 22.9, 14.1, 10.4. HRMS–ESI (m/z): $[M+H]^+$ calcd. for $C_{24}H_{42}N_3O_3$, 420.3226; found, 420.3228.

Biological Evaluation and Structure–Activity Relationship Analysis

Cell Culture

Neonatal mouse cardiac fibroblasts were isolated from 1–3 day-old NMRI mice. The protocol utilized MACS, (magnetic-activated cell sorting) technology by Miltenyi Biotec.[S21] The animals were sacrificed by decapitation. Ventricles were dissected and dissociated into single-cell suspensions using a Neonatal Heart Dissociation Kit (#130-098-373) and a gentle MACS Dissociator (#130-093-235) according to the manufacturer's protocol. Fibroblasts were separated with a Neonatal Cardiac Fibroblast Isolation Kit (#130-101-372) according to the manufacturer's protocol using MS columns (#130-042-201). The cells were cultured on gelatin-coated cell culture plates in Dulbecco's modified Eagle medium/Nutrient mixture F-12 (DMEM/F-12) supplemented with 10% fetal bovine serum (FBS), 100 U/mL penicillin, and 100 µg/mL streptomycin overnight prior to compound exposures.

Displacement Assay

Materials: [20-^{3}H]Phorbol-12,13-dibutyrate ([^{3}H]PDBu) (20 Ci/mmol) was acquired from American Radiolabeled Chemicals Inc. (Saint Louis, MO). Phorbol 12-myristate-13-acetate (PMA) and 1,2-diacyl-*sn*-glycero-3-phospho-L-serine (PS; product number: P6641) and bovine immunoglobulin G (IgG) were purchased from Sigma-Aldrich (Steinheim, Germany). The protease inhibitor (Complete Protease Inhibitor Cocktail Tablets) was from Roche (Mannheim, Germany) and the Optiphase SuperMix liquid scintillant was from PerkinElmer (Groningen, Netherlands).

Method: PKCα protein was produced in recombinant baculovirus-infected Sf9 cells as described previously.[S22] The cells were harvested two days after infection, washed with PBS, and the resultant cell pellets were frozen. Subsequently, the cells were suspended in a buffer containing 25 mM Tris-HCl (pH 7.5), 0.5 mM EGTA, 0.1% Triton X-100, and protease inhibitors to prepare a crude cell lysate. After a 30-min incubation on ice, the lysate was

centrifuged at 16000 g for 15 min at 4 °C and the supernatant representing the soluble (cytosolic) fraction was collected. The protein content of the supernatant was determined using the Bradford assay. The ability of the compounds to compete in binding to the regulatory domain of PKCα with radioactively labeled phorbol ester [^{3}H]PDBu was determined as described previously.[S23] First, 20 µg of protein/well from the supernatant was incubated with the test compounds and [^{3}H]PDBu for 10 min at rt in a 96-well Durapore filter plate (Millipore, cat. no. MSHVN4B50, Carrigtwohill, Ireland) in a total volume of 125 µL. The final concentrations in the assay were as follows: 20 mM Tris-HCl (pH 7.5), 40 µM $CaCl_2$, 10 mM $MgCl_2$, 400 µg/mL bovine IgG, 25 nM [^{3}H]PDBu, and 0.1 mg/mL PS. Proteins were then precipitated by the addition of cold 20% poly(ethylene glycol) 6000, and after 15 min of incubation on a plate shaker at rt the filters were washed six times using a vacuum manifold with buffer containing 20 mM Tris-HCl (pH 7.5), 100 µM $CaCl_2$, and 5 mM $MgCl_2$. The plates were dried and Optiphase SuperMix liquid scintillant was added to each well. After an equilibration period of 3 h, the radioactivity was measured using a Wallac Microbeta Trilux microplate liquid scintillation counter (PerkinElmer, Waltham, MA, USA). All tested compounds were diluted in DMSO to give the same final DMSO concentration (4%) in the binding assay in each well. PMA (1 µM) was used as a positive control in all assays and as the nonspecific binding was around 5%, only the total binding was measured. The results were calculated as a percentage of control (DMSO) from the same plate. The graphs were created using Graph-Pad Prism version 5.02 for Windows (GraphPad Software, La Jolla, CA, www.graphpad.com).

Western Blotting

The cells were grown on 6-well plates and exposed to the compounds for 24 h after which they were washed with ice-cold PBS and lysed on ice with 1% SDS in 50 mM Tris-HCl (pH 7.5), and genomic DNA was sheared with a 25 G needle. Protein concentrations were determined with bicinchoninic acid protein assay kit. From each sample 10 µg of total pro-

tein was resolved on a 12% Mini-protean TGX stain-free gel and transferred by Trans-Blot Turbo transfer system to polyvinylidene difluoride membranes. The membranes were blocked with 5% non-fat milk in 0.1% Tween 20 in Tris-buffered saline (TBST) for 1 h at rt, after which they were incubated overnight at 4 °C in a shaker with primary antibodies: anti-PKCα (ab32376; Abcam, 1:1000 dilution), anti-PKCδ (ab182126; Abcam, 1:1000 dilution), anti-PKCϵ (ab124806; Abcam, 1:1000 dilution), and Anti-Glyceraldehyde-3-Phosphate Dehydrogenase Antibody, clone 6C5 (GAPDH) (MAB374; Sigma-Aldrich, 1:10000 dilution). On the following day, the membranes were washed with TBST and incubated with blocking buffer containing HRP-linked secondary antibody (goat anti-rabbit, #170-6515; Bio-Rad, CA, USA or antimouse IgG #7076S; Cell Signaling Technology) for 1 h at rt. Secondary antibodies were detected with chemiluminescent substrate (SuperSignal West Pico, #34080; Thermo Fisher) utilizing LAS 3000 Imaging System (Fujifilm, Tokyo, Japan). Quantification was carried out by measuring the optical densities of the immunoreactive bands using IMAGEJ software (https://imagej.net/Downloads). The optical densities were first normalized to the optical densities of glyceraldehyde 3-phosphate dehydrogenase (GAPDH) immunoreactive bands, which served as the loading controls, and then to the corresponding control (cells treated with the vehicle only) on the same membrane.

ERK1/2 Phosphorylation Assay

The MEK–ERK pathway activation was studied by measuring ERK1/2 phosphorylation in neonatal mouse cardiac fibroblasts exposed to HMIs and pyridine analogs. The cells were exposed to the compounds with or without PKC inhibitor Gö6983 for 30 minutes after which they were lysed and the amount of phospho-ERK1/2 (p-ERK1/2) and total ERK1/2 were detected using AlphaLISA *SureFire Ultra* p-ERK 1/2 (Thr202/Tyr204) and AlphaLISA *SureFire Ultra* Total ERK1/2 assay kits (PerkinElmer) according to the manufacturer's protocol. The cells were lysed in 80 µL lysis buffer, and 30 µL of the lysate was transferred from each well to 96-well 1/2 Area OptiPlates (PerkinElmer). The lysates were then incu-

bated with the Acceptor bead mix for 1 h at rt, followed by the addition of the Donor bead mix and incubation overnight at rt. The Alpha signal was measured using an EnSpire Alpha plate reader (PerkinElmer) with standard AlphaLISA settings.

Cell Viability Assay

The DU145 human prostate cancer cells were cultured at 37 °C in a humidified atmosphere of 5% carbon dioxide. The cells were grown in RPMI1640 medium, supplemented with 10% FBS and 1% of penicillin-streptomycin as described previously,[S24] and passaged every seventh day at a 1:12 ratio. Upon reaching 80–90% confluence, the cells were seeded on a sterile 96-well plate at a density of 1×10^4 cells/well, and grown for 24 h before compound exposure. The compounds were diluted to the normal growth medium, added to the cells, and after 24 h, the MTT solution (5 mg/mL in HBSS) was added to each well at a final concentration of 0.5 mg/mL. The plate was then incubated for 2 h in a cell culture incubator, whereafter the culture medium was removed and 200 µL of DMSO was added to each well to dissolve the purple formazan crystals generated from the enzymatic activity of viable cells. The absorbance was read at 550 nm and the absorbance at 655 nm was subtracted as the background. The results of each experiment were calculated as average from three technical replicates to represent N=1, and the experiments were repeated three times (N=3).

Ethics

The animals were housed and terminated in accordance with the 3R principles of the European Union directive 2010/63/EU governing the care and use of experimental animals, and following local laws and regulations [Finnish Act on the Protection of Animals Used for Scientific or Educational Purposes (497/2013), Government Decree on the Protection of Animals Used for Scientific or Educational Purposes (564/2013)]. The use of animals for collecting tissues was reviewed and approved by Laboratory Animal Center, Helsinki Institute of Life Sciences, University of Helsinki (internal license KEK17-012).

References

(S1) Lautala, S.; Provenzani, R.; Koivuniemi, A.; Kulig, W.; Talman, V.; Róg, T.; Tuominen, R. K.; Yli-Kauhaluoma, J.; Bunker, A. Rigorous Computational Study Reveals What Docking Overlooks: Double Trouble from Membrane Association in Protein Kinase C Modulators. *J. Chem. Inf. Model.* **2020**, *60*, 5624–5633.

(S2) Hanwell, M. D.; Curtis, D. E.; Lonie, D. C.; Vandermeerschd, T.; Zurek, E.; Hutchison, G. R. Avogadro: An advanced semantic chemical editor, visualization, and analysis platform. *J. Cheminf.* **2012**, *4*.

(S3) Wang, J.; Wolf, R. M.; Caldwell, J. W.; Kollman, P. A.; Case, D. A. Development and testing of a general Amber force field. *J. Comput. Chem.* **2004**, *25*, 1157–1174.

(S4) M.J. Frisch, G. T.; H.B. Schlegel, G. S.; M.A. Robb, J. C.; G. Scalmani, V.; Barone, G. P.; H. Nakatsuji, X. L.; M. Caricato, A. M.; J. Bloino, B. J.; R., G.; B. Mennucci, H. H.; J.V. Ortiz, A. I.; J.L. Sonnenberg, W.; F. Ding, F.; Lipparini, F. E.; J. Goings, B. P.; A. Petrone, T. H.; D. Ranasinghe, V. Z.; J. Gao, N.; Rega, G. Z.; W. Liang, M. H.; M. Ehara, K. T.; R. Fukuda, J. H.; M. Ishida, T. N.; Y., H.; O. Kitao, H. N.; T. Vreven, K. T.; J.A. Montgomery Jr., J. P.; F. Ogliaro, M.; Bearpark, J. H.; E.N. Brothers, K. K.; V.N. Staroverov, T. K.; R. Kobayashi, J. N.; K., R.; A.P. Rendell, J. B.; S.S. Iyengar, J. T.; M. Cossi, J. M.; M. Klene, C. A.; R. Cammi, J. O.; R.L. Martin, K. M.; O. Farkas, J. F. Gaussian 16 Rev. B.01.

(S5) Salomon-Ferrer, R.; Case, D. A.; Walker, R. C. An overview of the Amber biomolecular simulation package. *Wiley Interdiscip. Rev.: Comput. Mol. Sci.* **2013**, *3*, 198–210.

(S6) D.A. Case, I. B.-S.; S.R. Brozell, D. C.; T.E. Cheatham, I.; V.W.D. Cruzeiro, T. D.; R.E. Duke, D. G.; M.K. Gilson, H. G.; A.W. Goetz, D. G.; R Harris, N. H.; Y. Huang, S. I.; A. Kovalenko, T. K.; T.S. Lee, S. L.; P. Li, C. L.; J. Liu, T. L.; R. Luo, D.; Mermelstein, K. M.; Y. Miao, G. M.; C. Nguyen, H. N.; I. Omelyan, A. O.; F. Pan, R.; Qi, D. R.; A. Roitberg, C. S.; S. Schott-Verdugo, J. S.; C.L. Simmerling, J. S.; R. SalomonFerrer, J. S.; R.C. Walker, J. W.; H. Wei, R. W.; X. Wu, L. X.; D.M. York, P. K. Amber 2018. 2018.

(S7) Ditchfield, R.; Hehre, W. J.; Pople, J. A. Self-Consistent Molecular-Orbital Methods. IX. An Extended Gaussian-Type Basis for Molecular-Orbital Studies of Organic Molecules. *J. Chem. Phys.* **1971**, *54*, 724–728.

(S8) Singh, U. C.; Kollman, P. A. An approach to computing electrostatic charges for molecules. *J. Comput. Chem.* **1984**, *5*, 129–145.

(S9) Bayly, C. I.; Cieplak, P.; Cornell, W.; Kollman, P. A. A well-behaved electrostatic potential based method using charge restraints for deriving atomic charges: the RESP model. *J. Phys. Chem.* **1993**, *97*, 10269–10280.

(S10) Berendsen, H. J. C.; van der Spoel, D.; van Drunen, R. GROMACS: A message-passing parallel molecular dynamics implementation. *Comput. Phys. Commun.* **1995**, *91*, 43–56.

(S11) Spoel, D. V. D.; Lindahl, E.; Hess, B.; Groenhof, G.; Mark, A. E.; Berendsen, H. J. C. GROMACS: Fast, flexible, and free. *J. Comput. Chem.* **2005**, *26*, 1701–1718.

(S12) development team, T. G. GROMACS Documentation release 2020.3. 2021.

(S13) Hess, B.; Bekker, H.; Berendsen, H. J. C.; Fraaije, J. G. E. M. LINCS: A linear constraint solver for molecular simulations. *J. Comput. Chem.* **1997**, *18*, 1463–1472.

(S14) Abraham, M.; van der Spoel, D.; Lindahl, E.; Hess, B.; the development team, G. GROMACS User Manual version 5.1.5. 2017.

(S15) Essmann, U.; Perera, L.; Berkowitz, M. L.; Darden, T.; Lee, H.; Pedersen, L. G. A smooth particle mesh Ewald method. *J. Chem. Phys.* **1995**, *103*, 8577–8593.

(S16) Nosé, S. A molecular dynamics method for simulations in the canonical ensemble. *Mol. Phys.* **1984**, *52*, 255–268.

(S17) Hoover, W. G. Canonical dynamics: Equilibrium phase-space distributions. *Phys. Rev. A* **1985**, *31*, 1695–1697.

(S18) Parrinello, M.; Rahman, A. Polymorphic transitions in single crystals: A new molecular dynamics method. *J. Appl. Phys.* **1981**, *52*, 7182–7190.

(S19) Nosé, S.; Klein, M. L. Constant pressure molecular dynamics for molecular systems. *Mol. Phys.* **1983**, *50*, 1055–1076.

(S20) Kapoor, M.; Liu, D.; Young, M. C. Carbon Dioxide-Mediated C(sp^3)–H Arylation of Amine Substrates. *J. Am. Chem. Soc.* **2018**, *140*, 6818–6822.

(S21) Miltenyi, S.; Müller, W.; Weichel, W.; Radbruch, A. High gradient magnetic cell separation with MACS. *Cytometry* **1990**, *11*, 231–238.

(S22) Tammela, P.; Ekokoski, E.; García-Horsman, A.; Talman, V.; Finel, M.; Tuominen, R.; Vuorela, P. Screening of natural compounds and their derivatives as potential protein kinase C inhibitors. *Drug Dev. Res.* **2004**, *63*, 76–87.

(S23) Boije af Gennäs, G.; Talman, V.; Aitio, O.; Ekokoski, E.; Finel, M.; Tuominen, R. K.; Yli-Kauhaluoma, J. Design, Synthesis, and Biological Activity of Isophthalic Acid Derivatives Targeted to the C1 Domain of Protein Kinase C. *J. Med. Chem.* **2009**, *52*, 3969–3981.

(S24) Jäntti, M. H.; Talman, V.; Räsänen, K.; Tarvainen, I.; Koistinen, H.; Tuominen, R. K. Anticancer activity of the protein kinase C modulator HMI-1a3 in 2D and 3D cell culture models of androgen-responsive and androgen-unresponsive prostate cancer. *FEBS Open Bio* **2018**, *8*, 817–828.

Supporting NMR Appendix

Peak reports and multiplet assignments are provided for all compounds. Compound peaks are highlighted in **green** and residual solvent peaks in **yellow**. All 2D NMR spectra are edited to highlight the correlation peaks. ^{1}H–^{13}C HSQC spectra include DEPT-135 information with –CH– and $–CH_3$ correlation peaks in positive phase (**red**) while $–CH_2–$ correlation peaks in negative phase (**blue**). For most of the compounds, ^{1}H–^{15}N HMBC spectrum and corresponding ^{15}N 1D projection are also reported.

Contents

Compounds	Page
Compound **2**	S20
Compound **3**	S26
Compound **4b**	S32
Compound **5a**	S38
Compound **5b**	S46
Compound **6a**	S52
Compound **6b**	S59
Compound **7a**	S65
Compound **7b**	S75
Compound **7c**	S81

Compound **8a** S87

Compound **8b** S110

Compound **S2** S130

^{1}H NMR (400 MHz, $CDCl_3$) δ 8.27 (app t, *J* = 0.9 Hz, 2H), 4.88 (d, *J* = 4.4 Hz, 2H), 3.99 (s, 6H), 2.85 (br s, 1H).

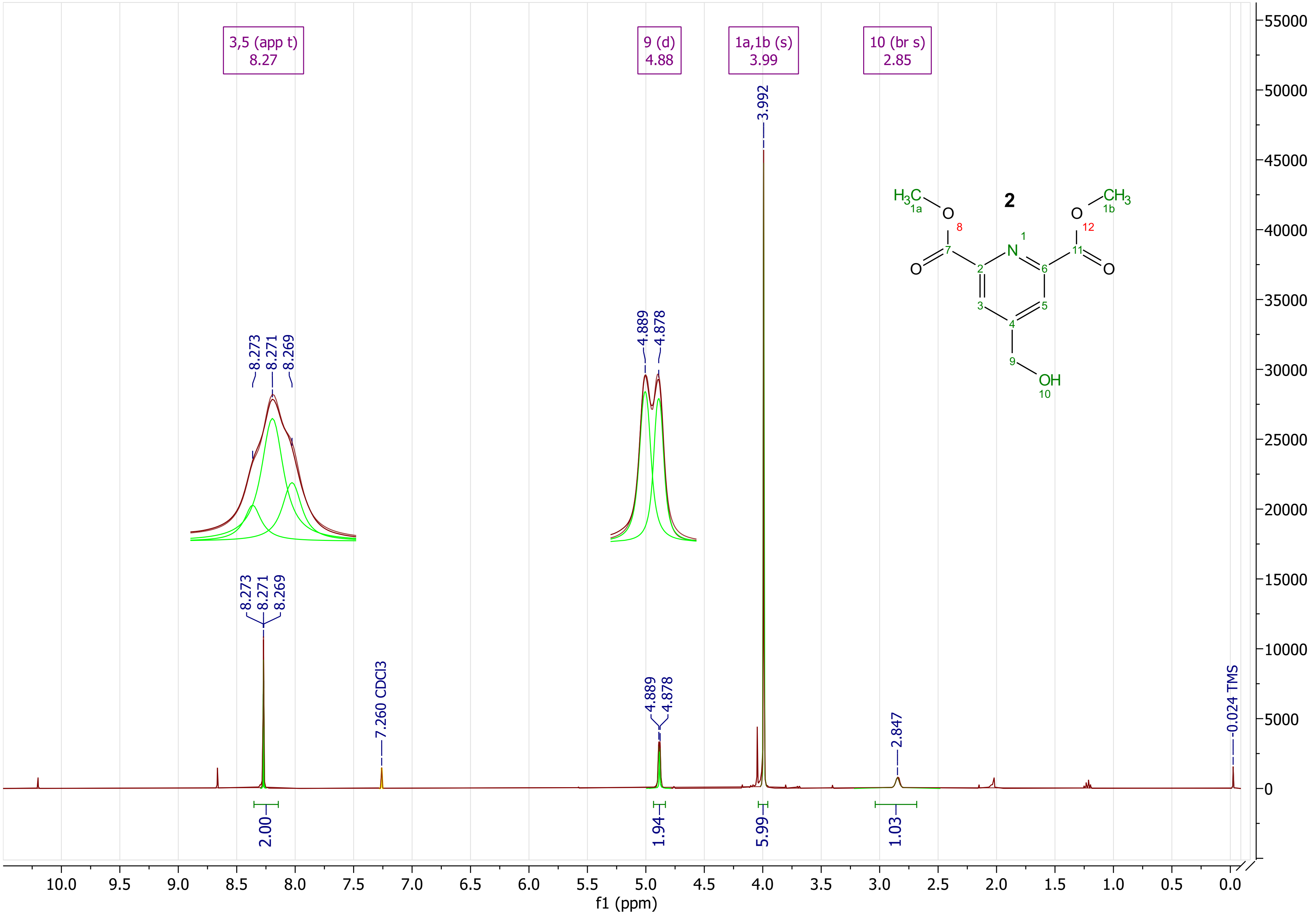

^{13}C NMR (101 MHz, $CDCl_3$) δ 165.3 (2C), 153.8, 148.3 (2C), 125.4 (2C), 62.9, 53.3 (2C).

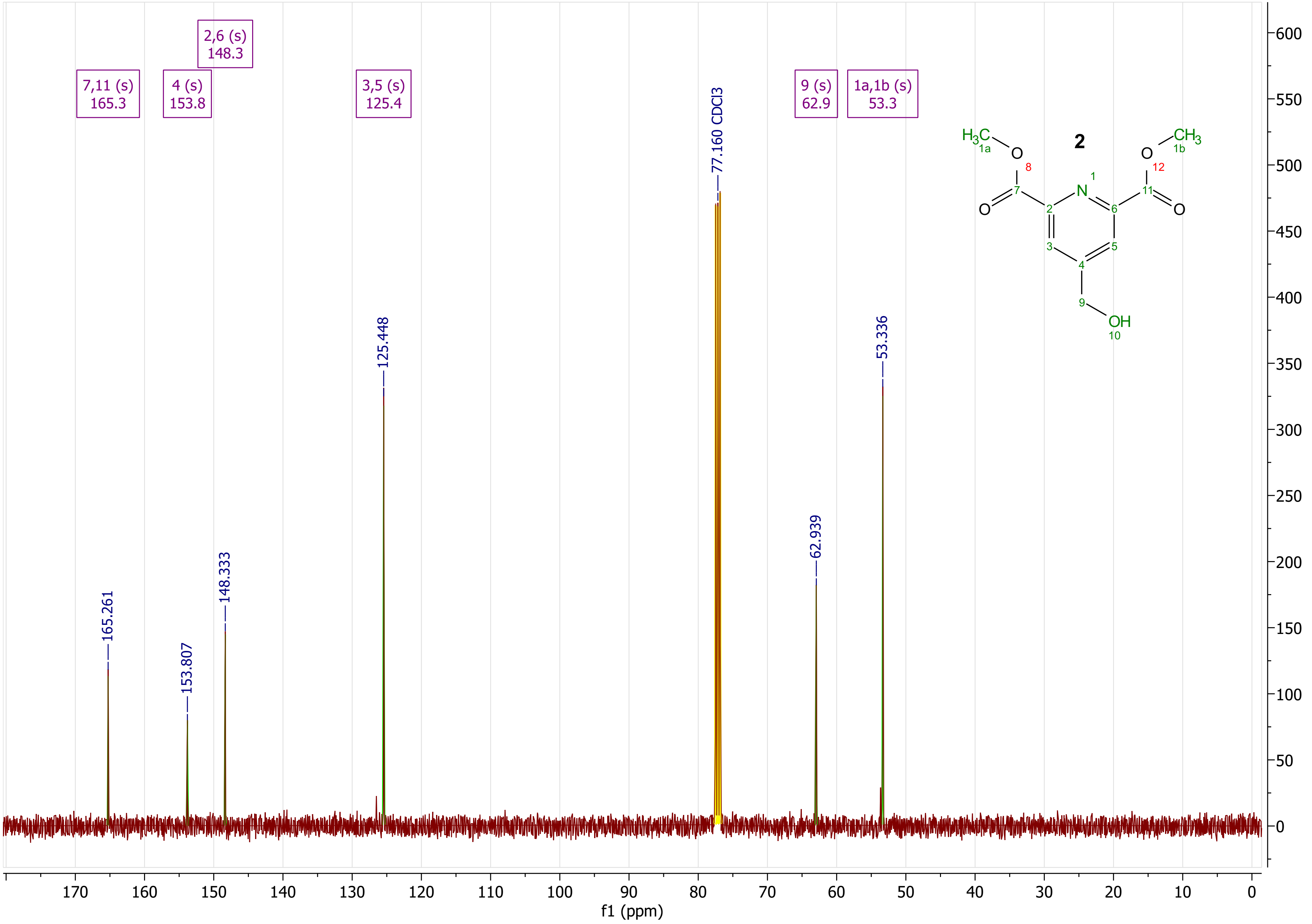

[Back ↑]

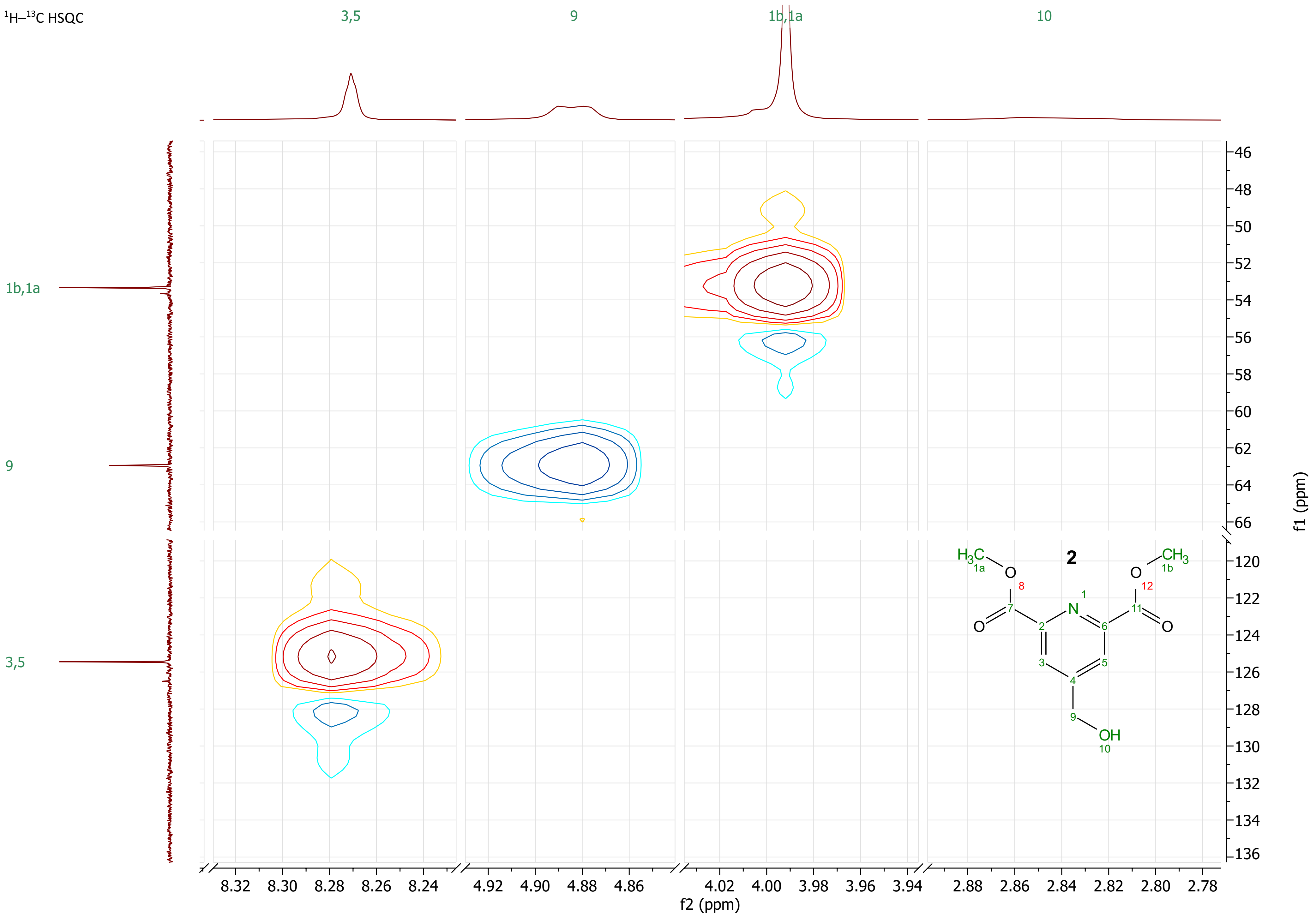

[Back ↑]

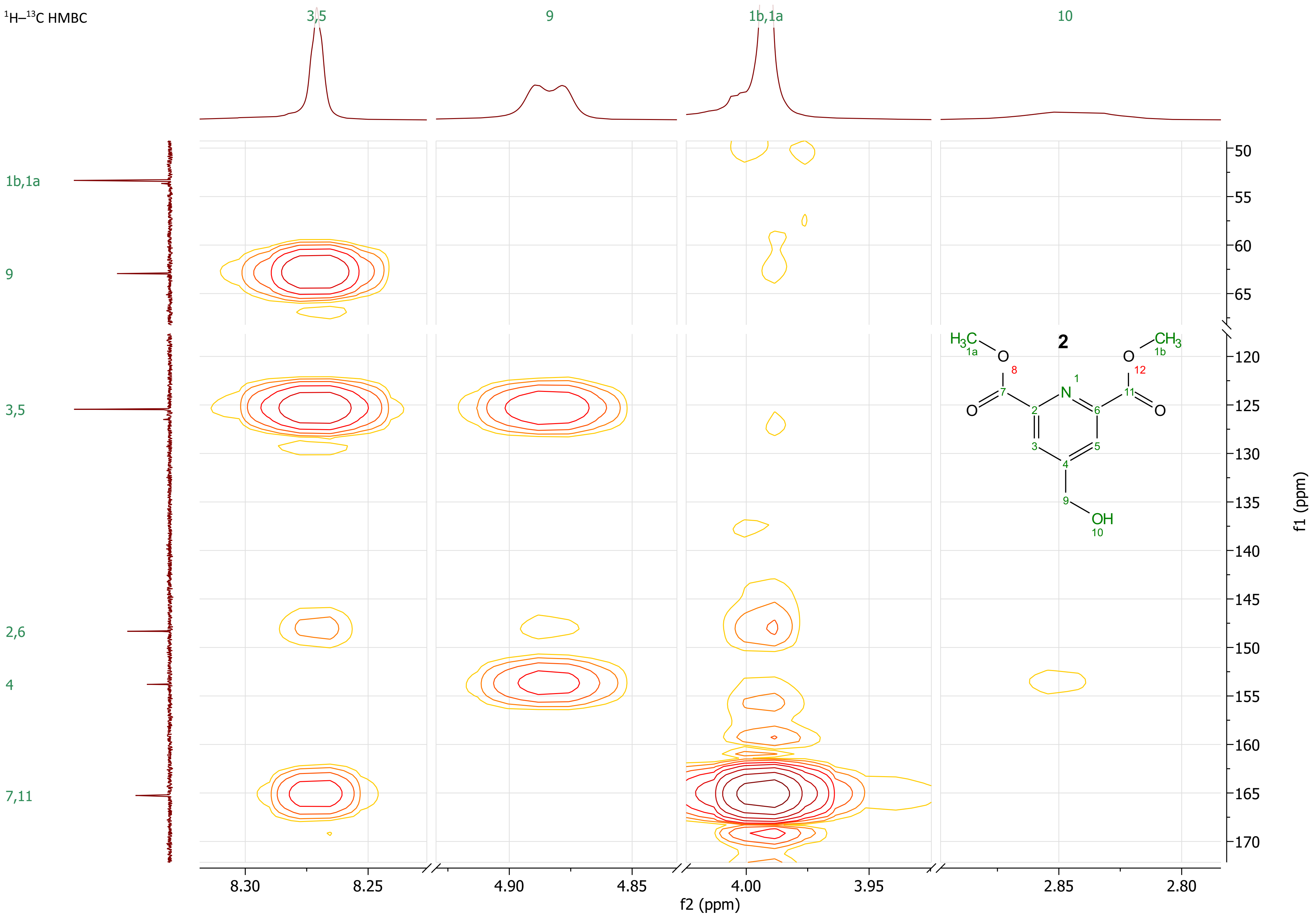

^{1}H–^{13}C HMBC
3,5
9
1b,1a
10
1b,1a
9
3,5
2,6
4
7,11
2
H3C
1a
O
8
7
O
N
1
2
3
4
5
6
11
O
12
O
CH3
1b
9
OH
10
f2 (ppm)
f1 (ppm)

[Back ↑]

^{1}H–^{15}N HMBC

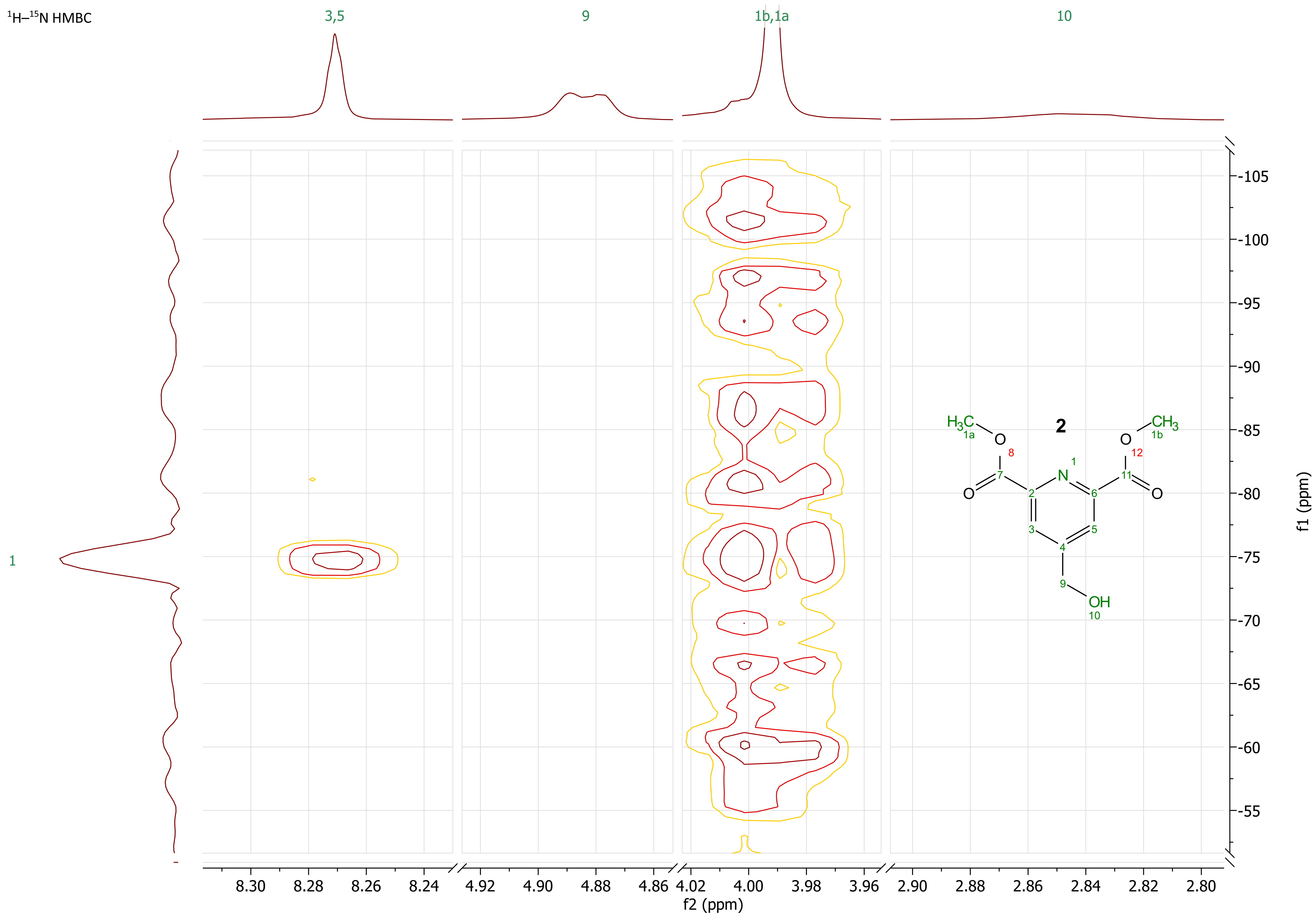

[Back ↑]

^{15}N NMR (41 MHz, $CDCl_3$) δ -74.72. – Projection f1

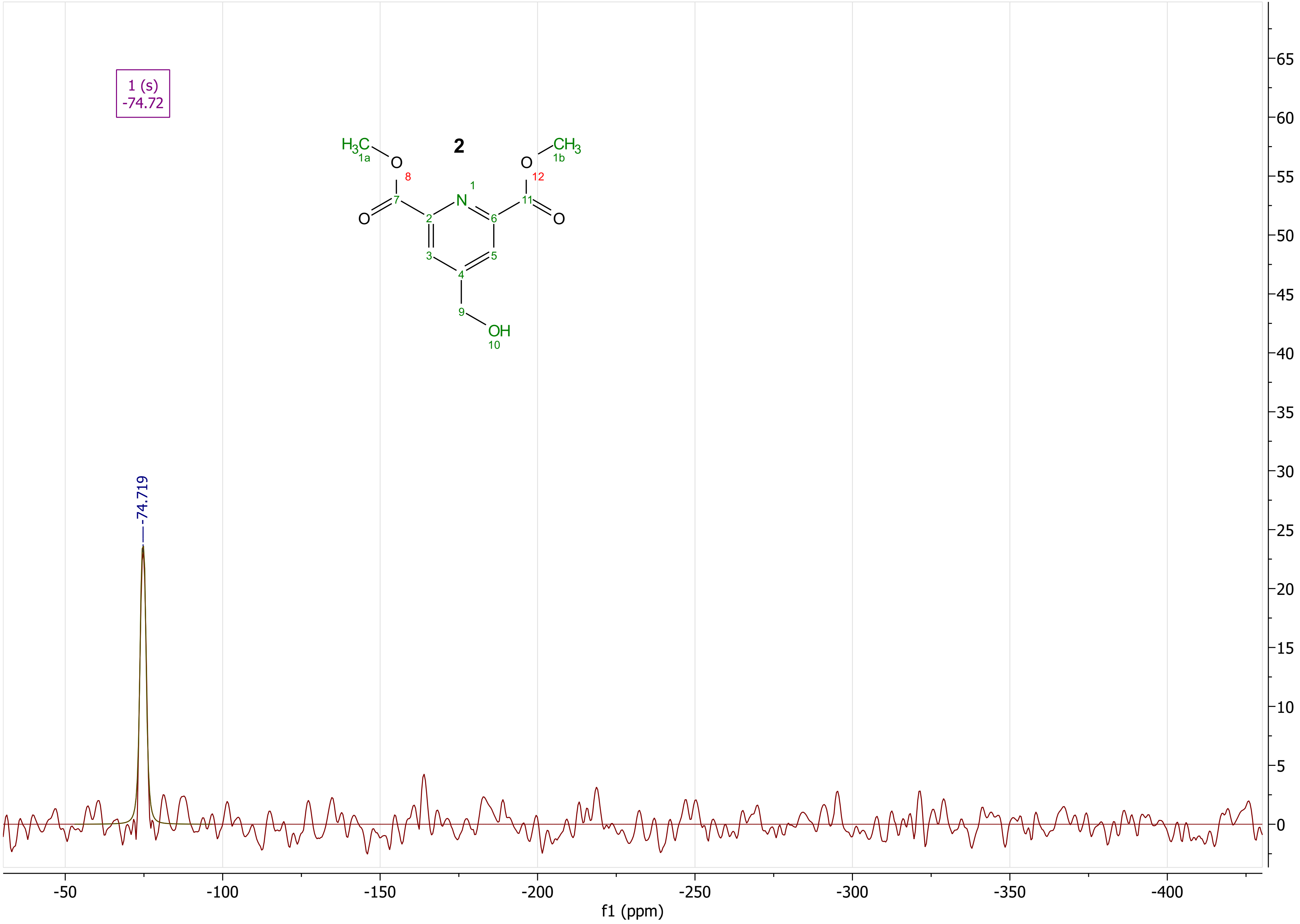

^{1}H NMR (400 MHz, $CDCl_3$) δ 8.28 (t, *J* = 0.8 Hz, 2H), 4.92 (dt, *J* = 14.3, 0.9 Hz, 1H), 4.75 (t, *J* = 3.5 Hz, 1H), 4.62 (dt, *J* = 14.3, 0.9 Hz, 1H), 4.01 (s, 6H), 3.89 – 3.79 (m, 1H), 3.60 – 3.50 (m, 1H), 1.96 – 1.81 (m, 1H), 1.85 – 1.67 (m, 2H), 1.70 – 1.49 (m, 3H).

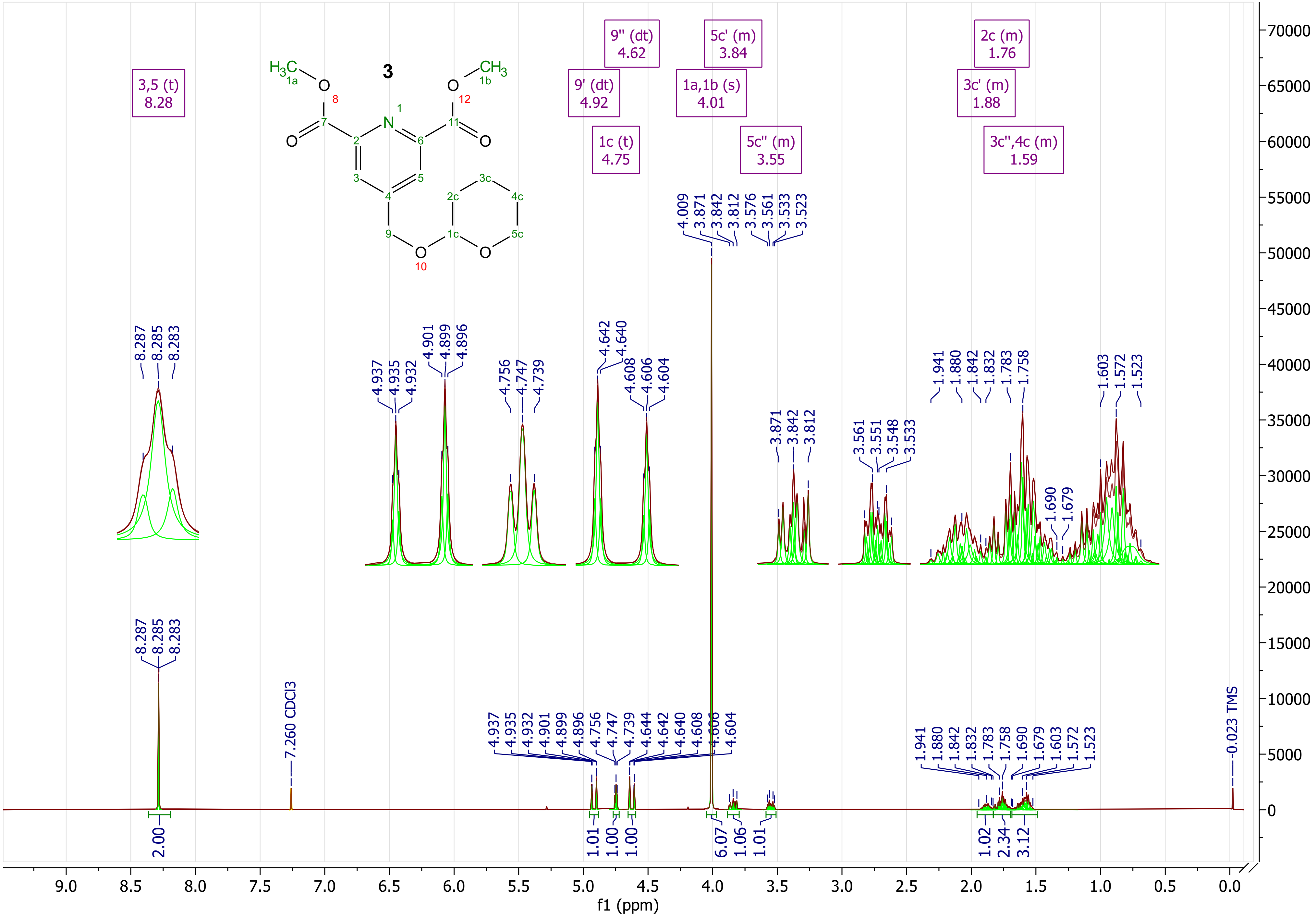

^{13}C NMR (101 MHz, $CDCl_3$) δ 165.3 (2C), 151.3, 148.4 (2C), 126.1 (2C), 98.7, 66.7, 62.4, 53.3 (2C), 30.4, 25.4, 19.2.

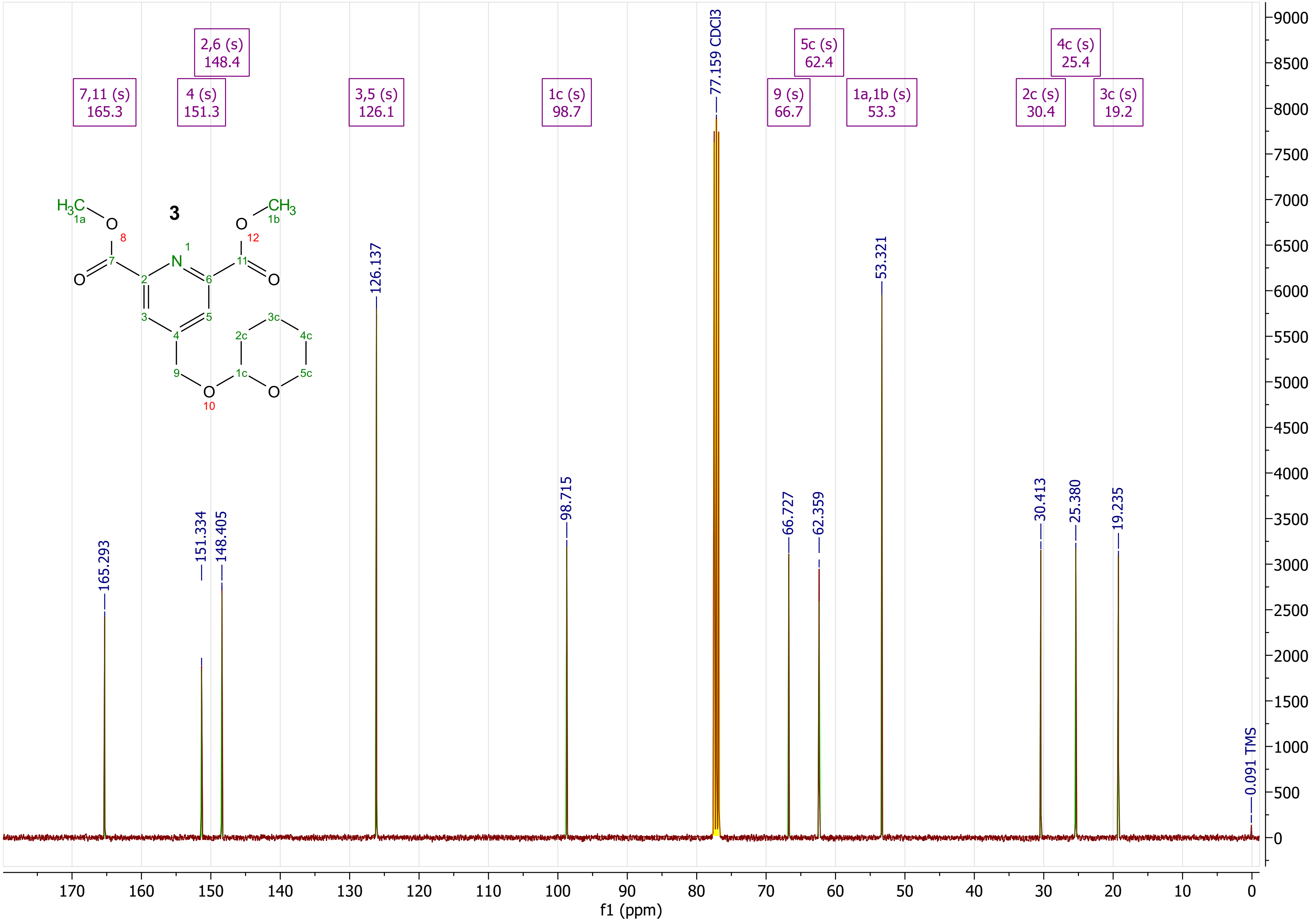

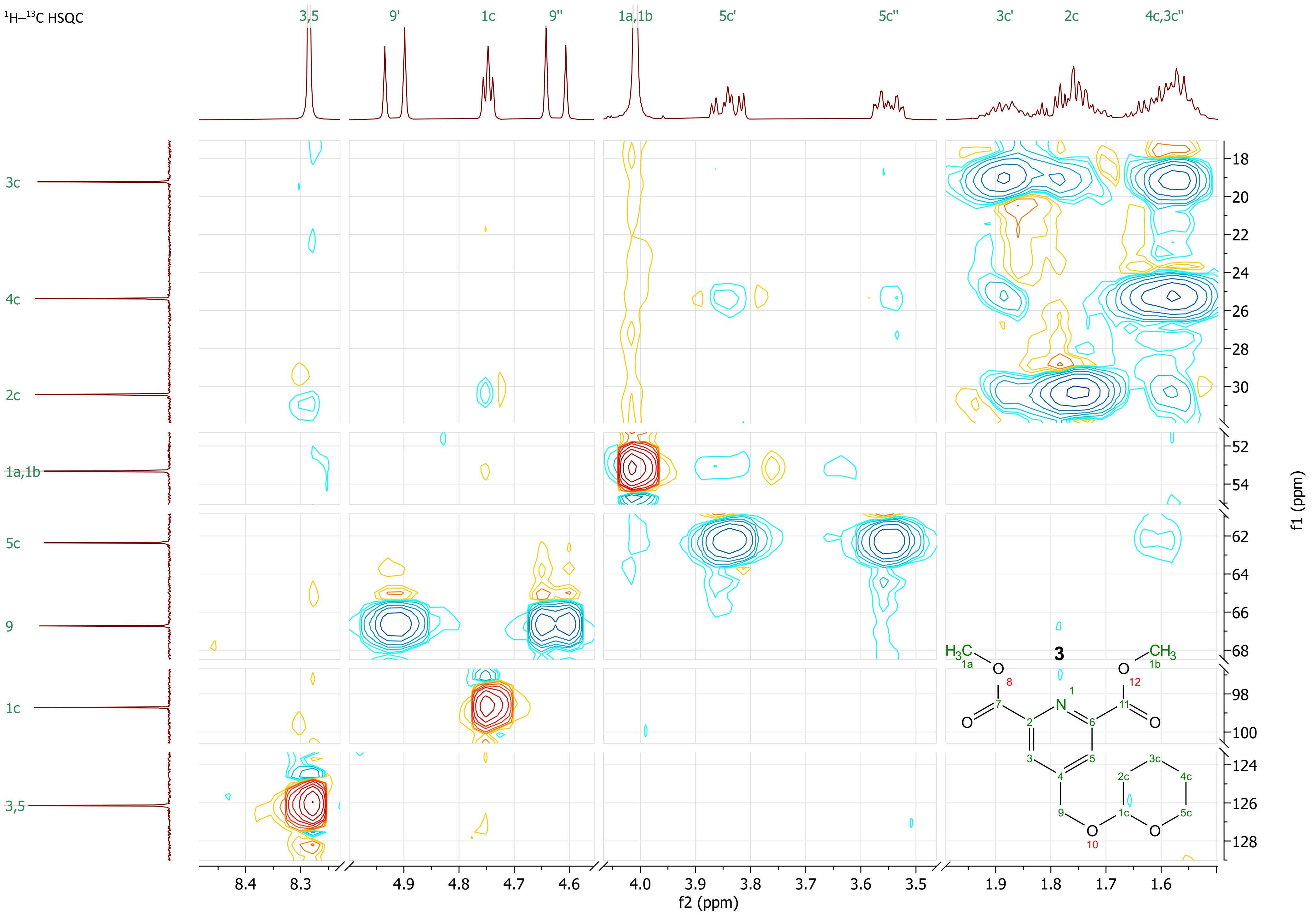

¹H–¹³C HSQC
3,5
9'
1c
9''
1a,1b
5c'
5c''
3c'
2c
4c,3c''
3c
4c
2c
1a,1b
5c
9
1c
3,5
f2 (ppm)
f1 (ppm)
3

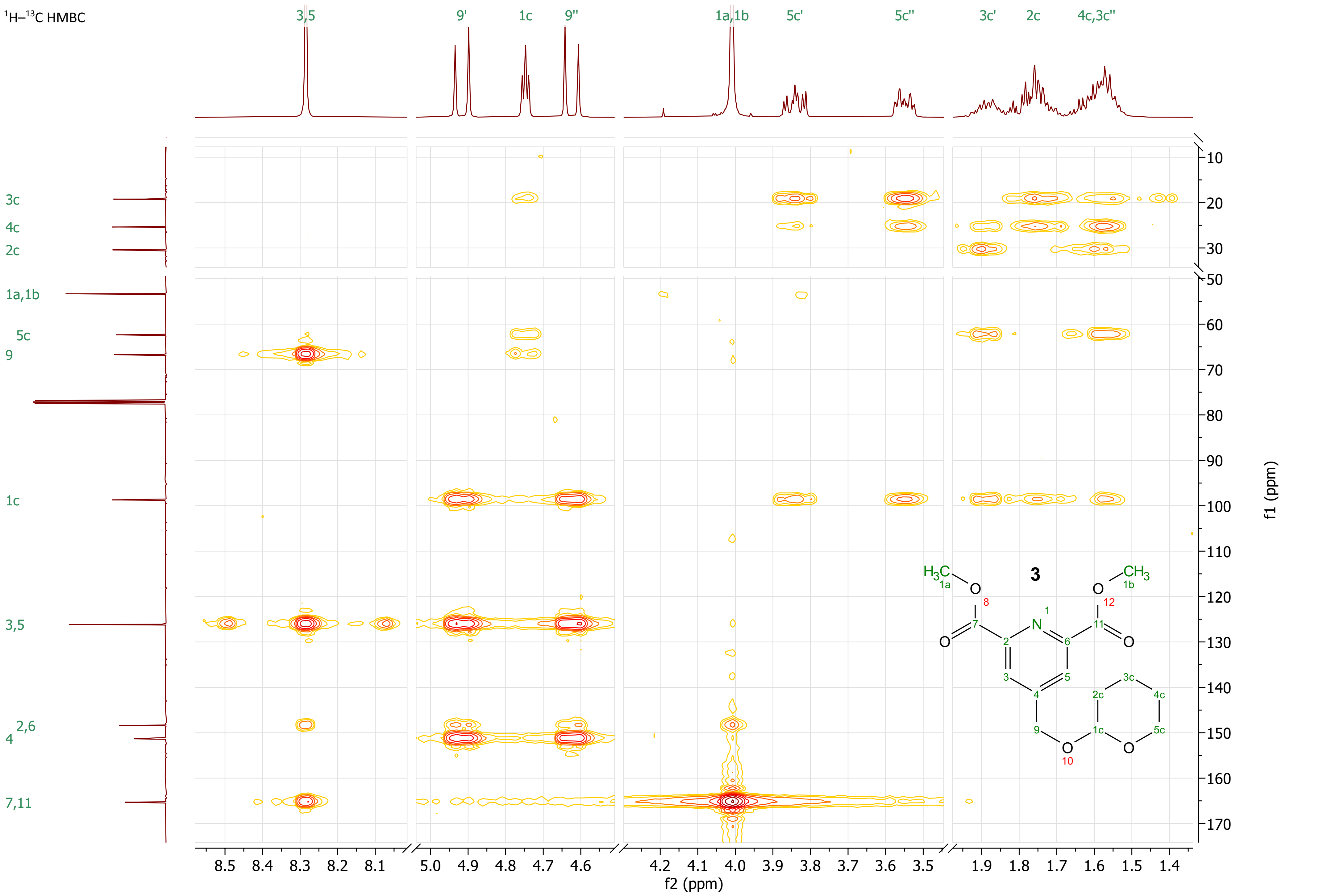

¹H–¹³C HMBC
3,5
9'
1c
9''
1a,1b
5c'
5c''
3c'
2c
4c,3c''
3c
4c
2c
1a,1b
5c
9
1c
3,5
2,6
4
7,11
f2 (ppm)
f1 (ppm)
3
H3C
1a
CH3
1b
O
N

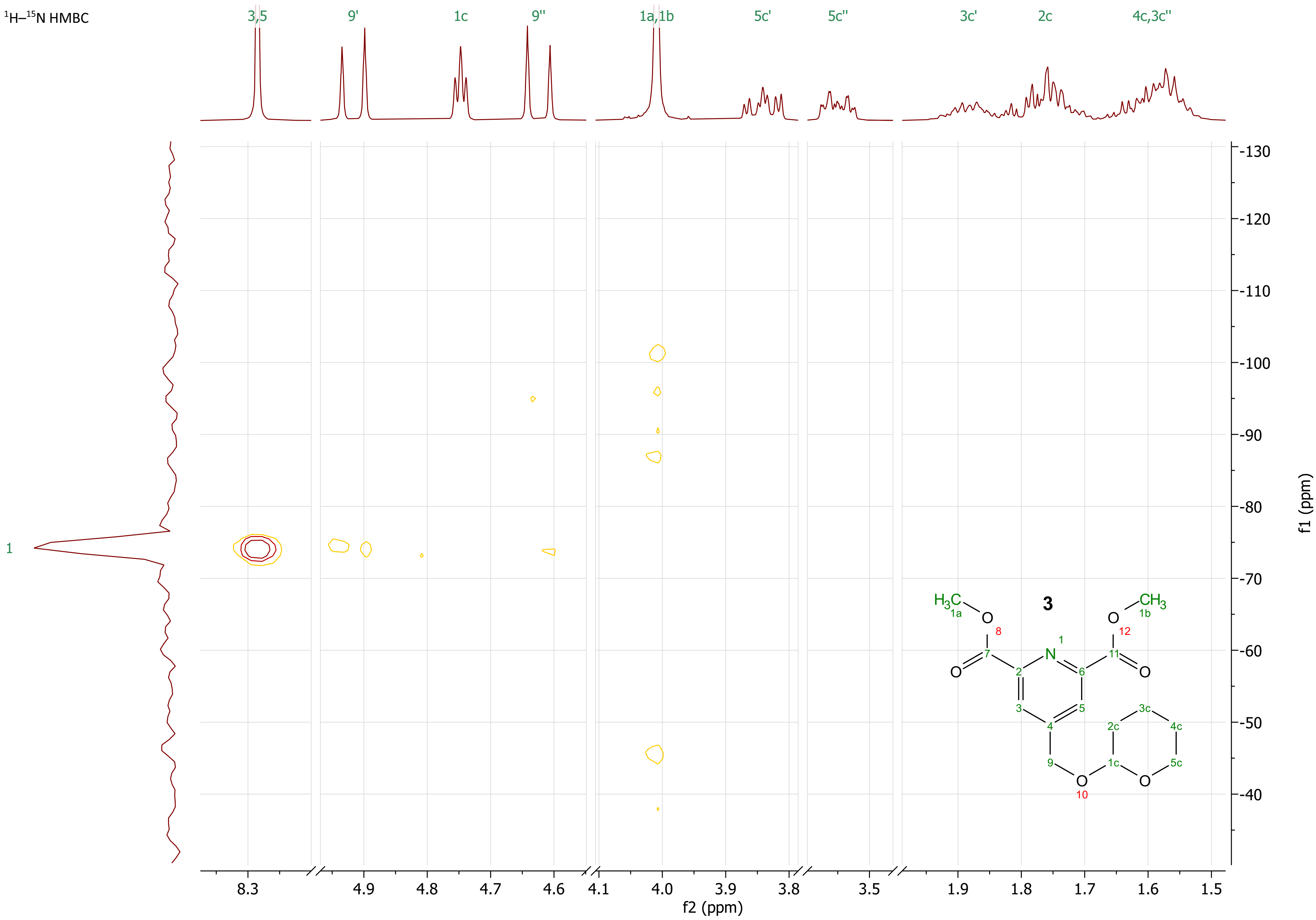

¹H–¹⁵N HMBC
3,5
9'
1c
9''
1a,1b
5c'
5c''
3c'
2c
4c,3c''
1
f1 (ppm)
f2 (ppm)
3

[Back ↑]

^{15}N NMR (41 MHz, $CDCl_3$) δ -74.29. – Projection f1

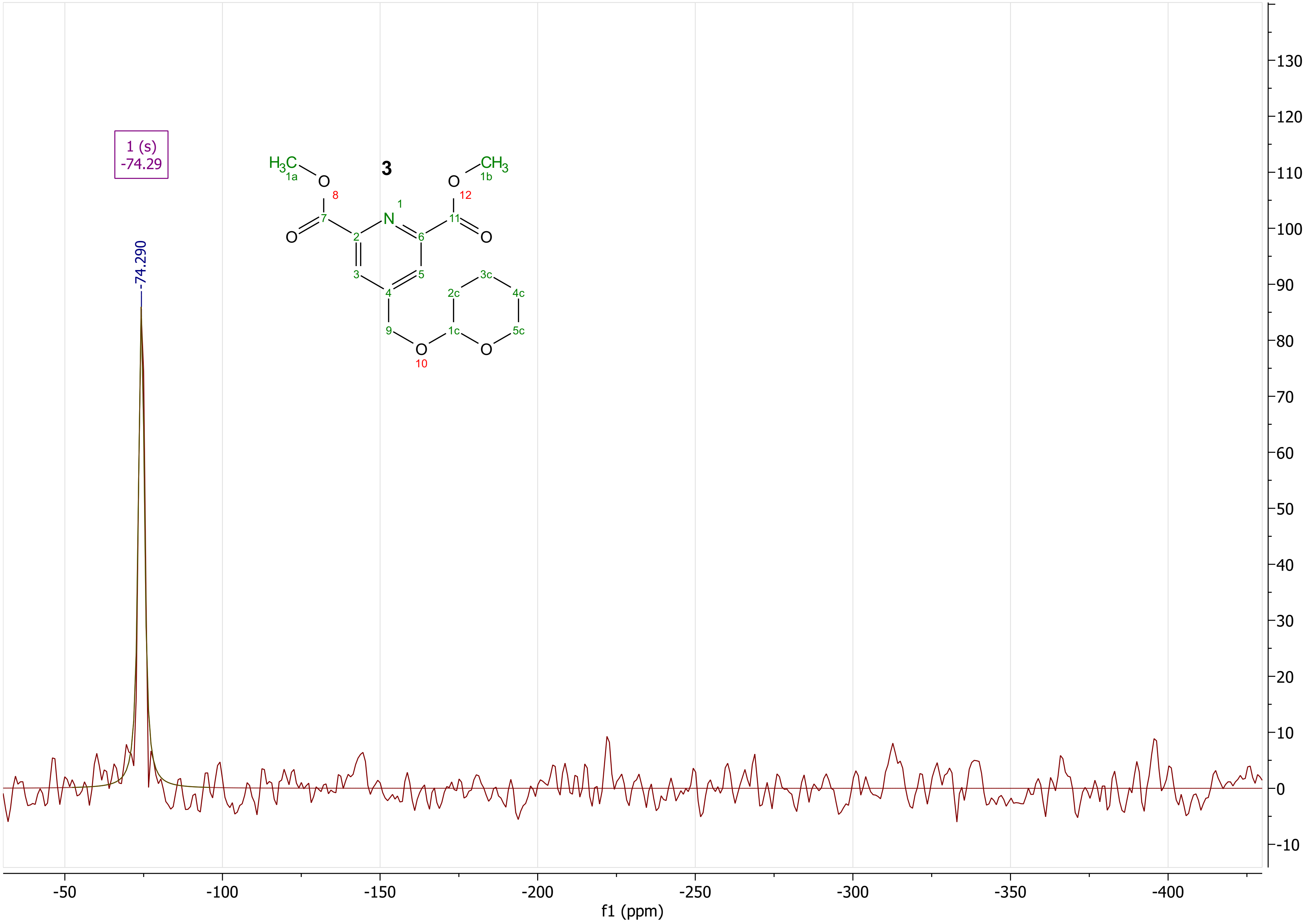

[Back ↑]

^{1}H NMR (400 MHz, $CDCl_3$) δ 8.18 (t, *J* = 0.8 Hz, 2H), 5.18 – 5.06 (m, 2H), 4.91 (dt, *J* = 14.2, 0.9 Hz, 1H), 4.75 (t, *J* = 3.5 Hz, 1H), 4.64 (dt, *J* = 14.2, 0.8 Hz, 1H), 3.99 – 3.74 (m, 1H), 3.69 – 3.43 (m, 1H), 1.95 – 1.85 (m, 1H), 1.84 – 1.65 (m, 10H), 1.65 – 1.50 (m, 3H), 1.45 – 1.27 (m, 8H), 0.96 (t, *J* = 7.4 Hz, 6H), 0.88 (app t, *J* = 7.1 Hz, 6H).

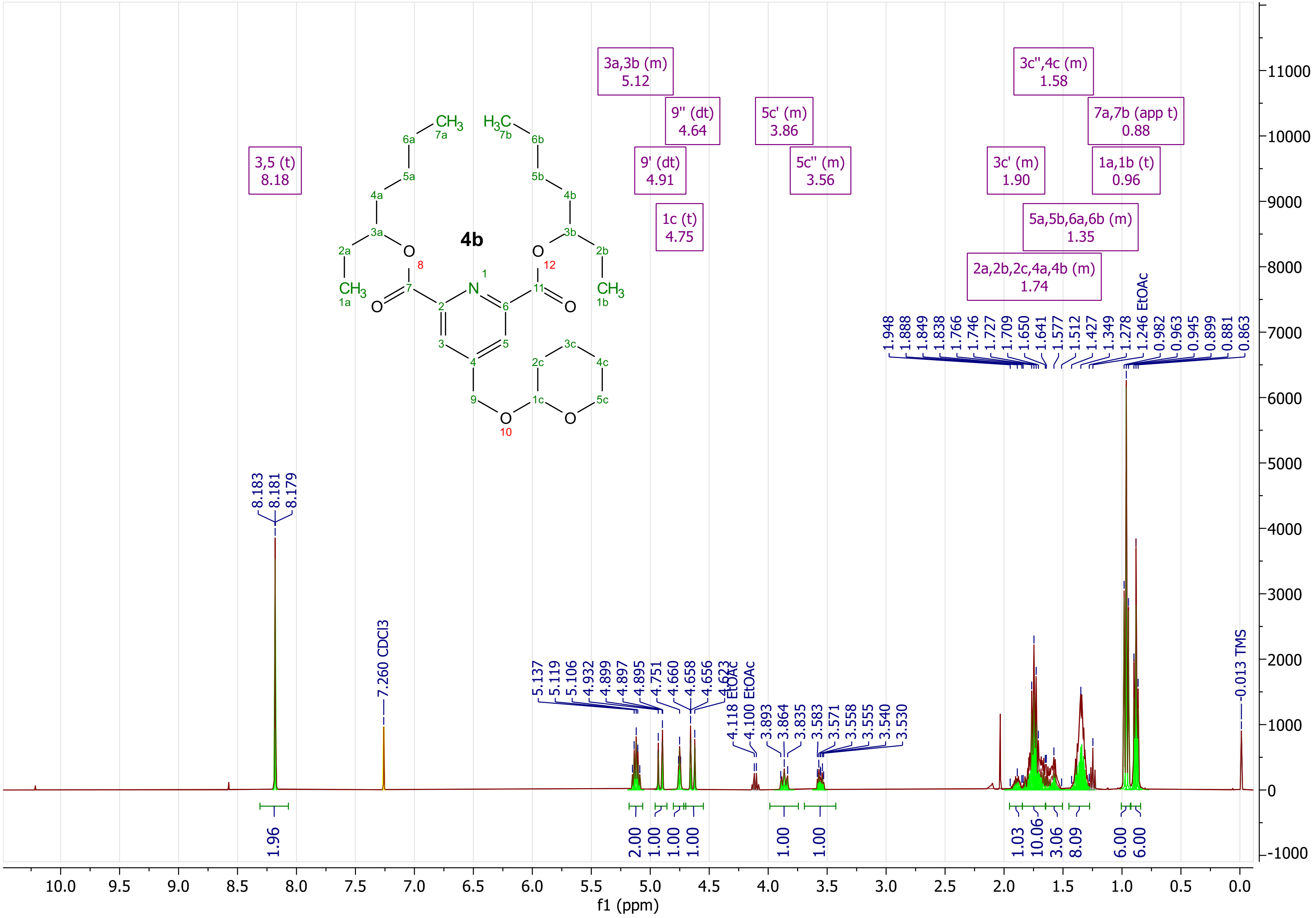

^{13}C NMR (101 MHz, $CDCl_3$) δ 164.6 (2C), 150.6, 149.4 (2C), 125.5 (2C), 98.6, 77.9 (2C), 66.8, 62.4, 33.3 (2C), 30.5, 27.6 (2C), 27.0 (2C), 25.4, 22.7 (2C), 19.3, 14.1 (2C), 9.8 (2C).

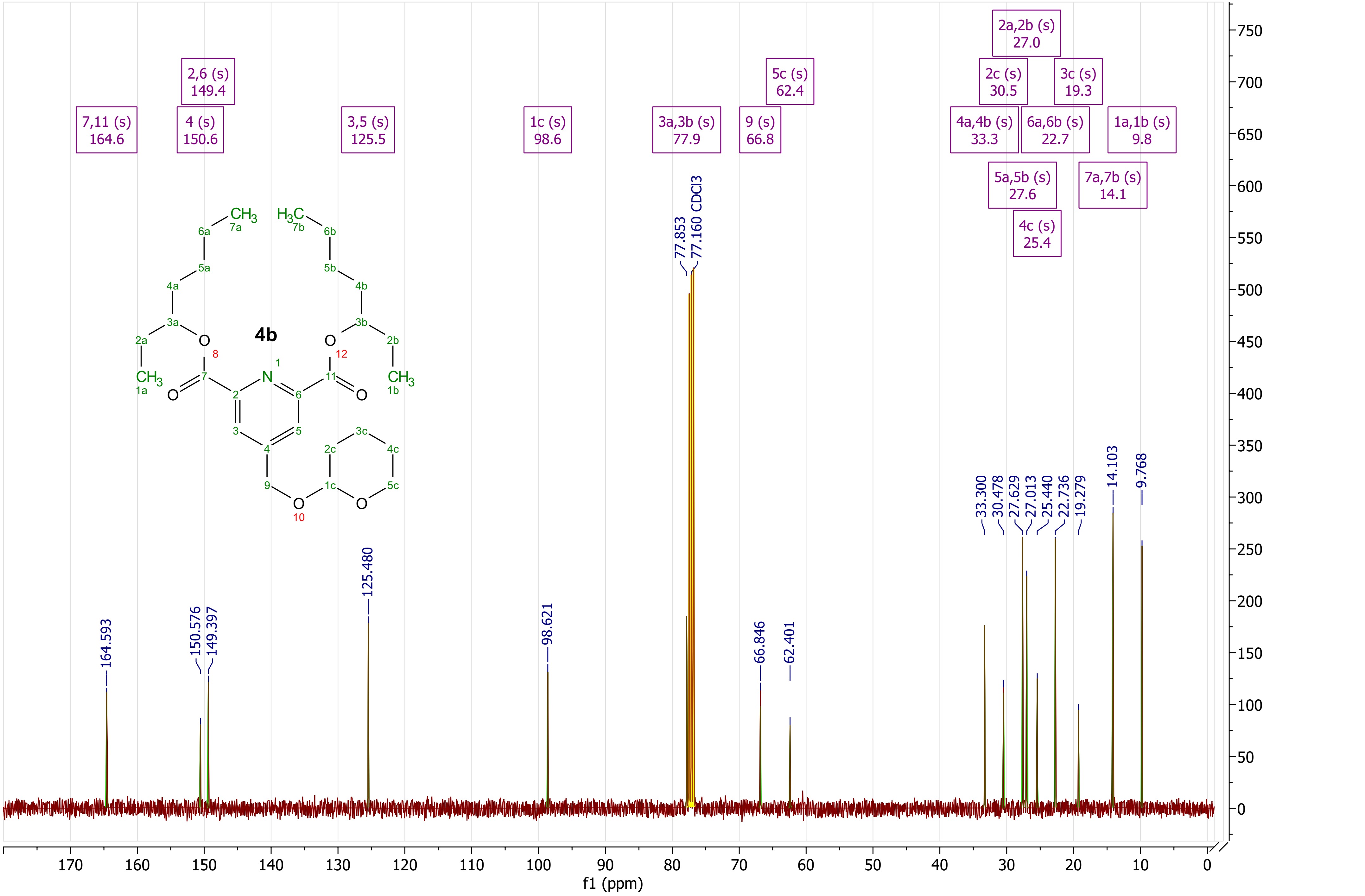

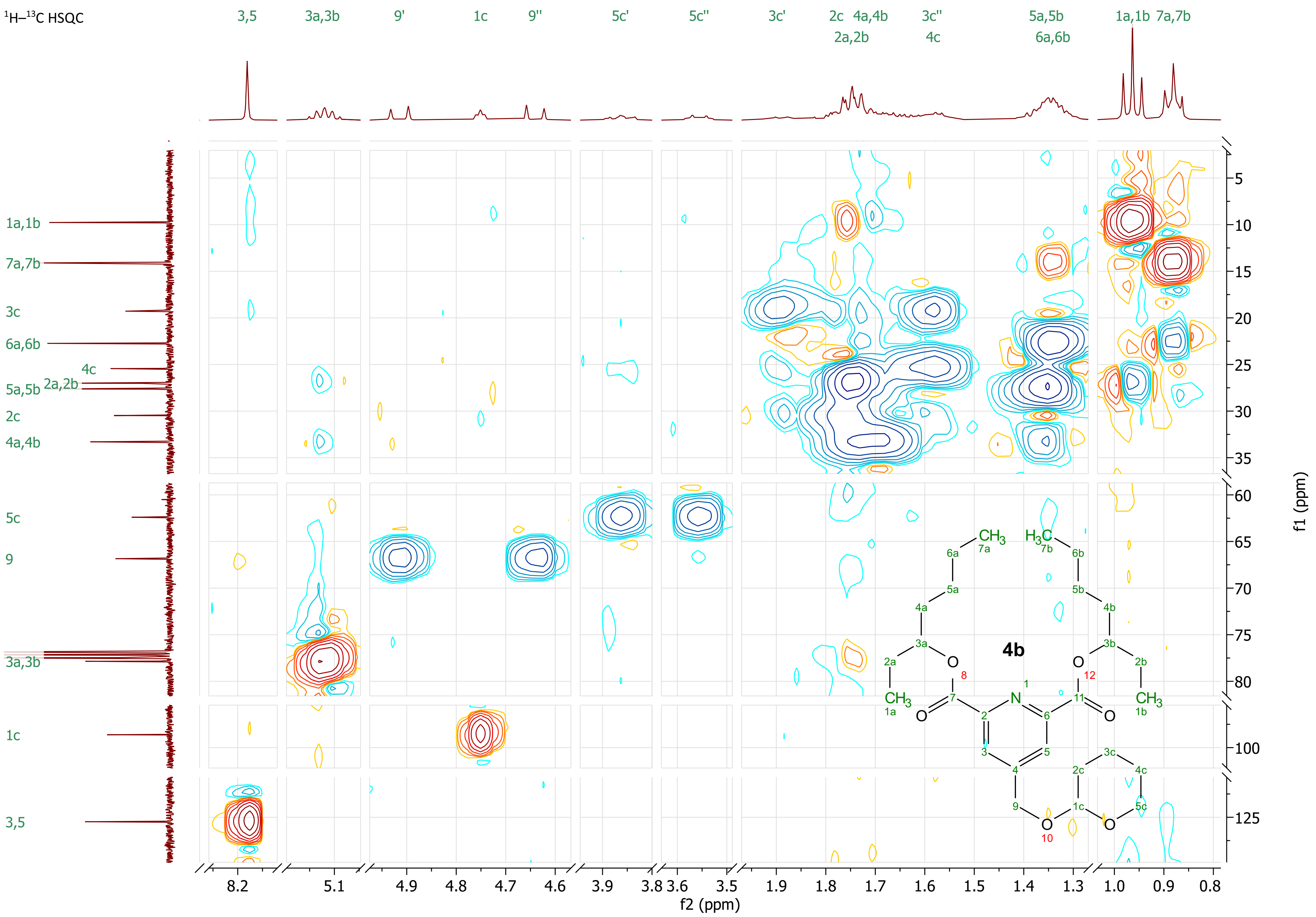

^1H–^{13}C HSQC
3,5
3a,3b
9'
1c
9''
5c'
5c''
3c'
2c 4a,4b
2a,2b
3c''
4c
5a,5b
6a,6b
1a,1b 7a,7b
1a,1b
7a,7b
3c
6a,6b
4c
5a,5b 2a,2b
2c
4a,4b
5c
9
3a,3b
1c
3,5
4b
f1 (ppm)
f2 (ppm)

[Back ↑]

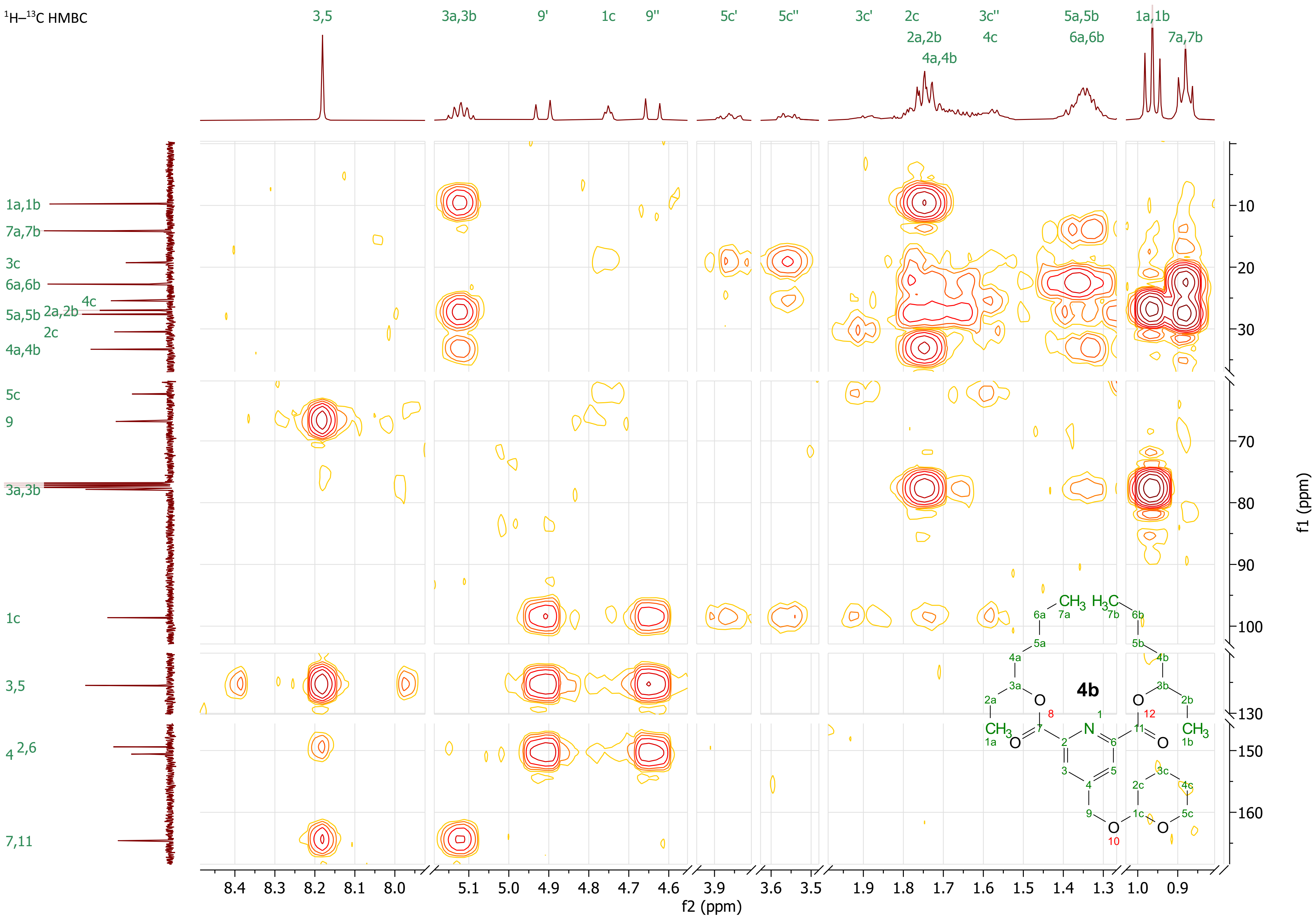
^{1}H–^{13}C HMBC
3,5
3a,3b
9'
1c
9''
5c'
5c''
3c'
2c
2a,2b
4a,4b
3c''
4c
5a,5b
6a,6b
1a,1b
7a,7b
5c
9
1c
3,5
4 2,6
7,11
4b
f2 (ppm)
f1 (ppm)

[Back ↑]

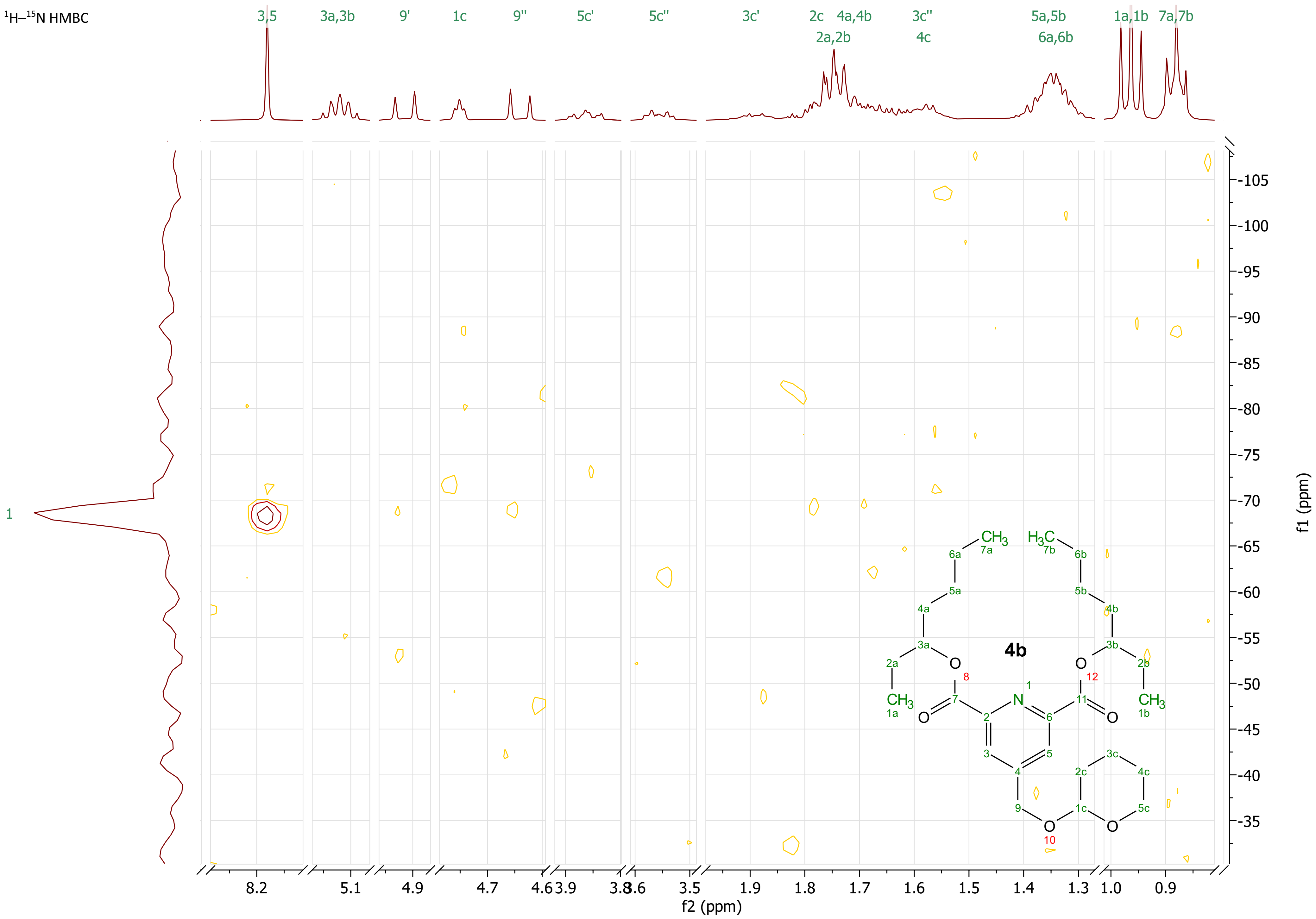

^{1}H–^{15}N HMBC
3,5
3a,3b
9'
1c
9''
5c'
5c''
3c'
2c
2a,2b
4a,4b
3c''
4c
5a,5b
6a,6b
1a,1b
7a,7b
1
4b
f2 (ppm)
f1 (ppm)

[Back ↑]

^{15}N NMR (41 MHz, $CDCl_3$) δ -68.6. – Projection f1

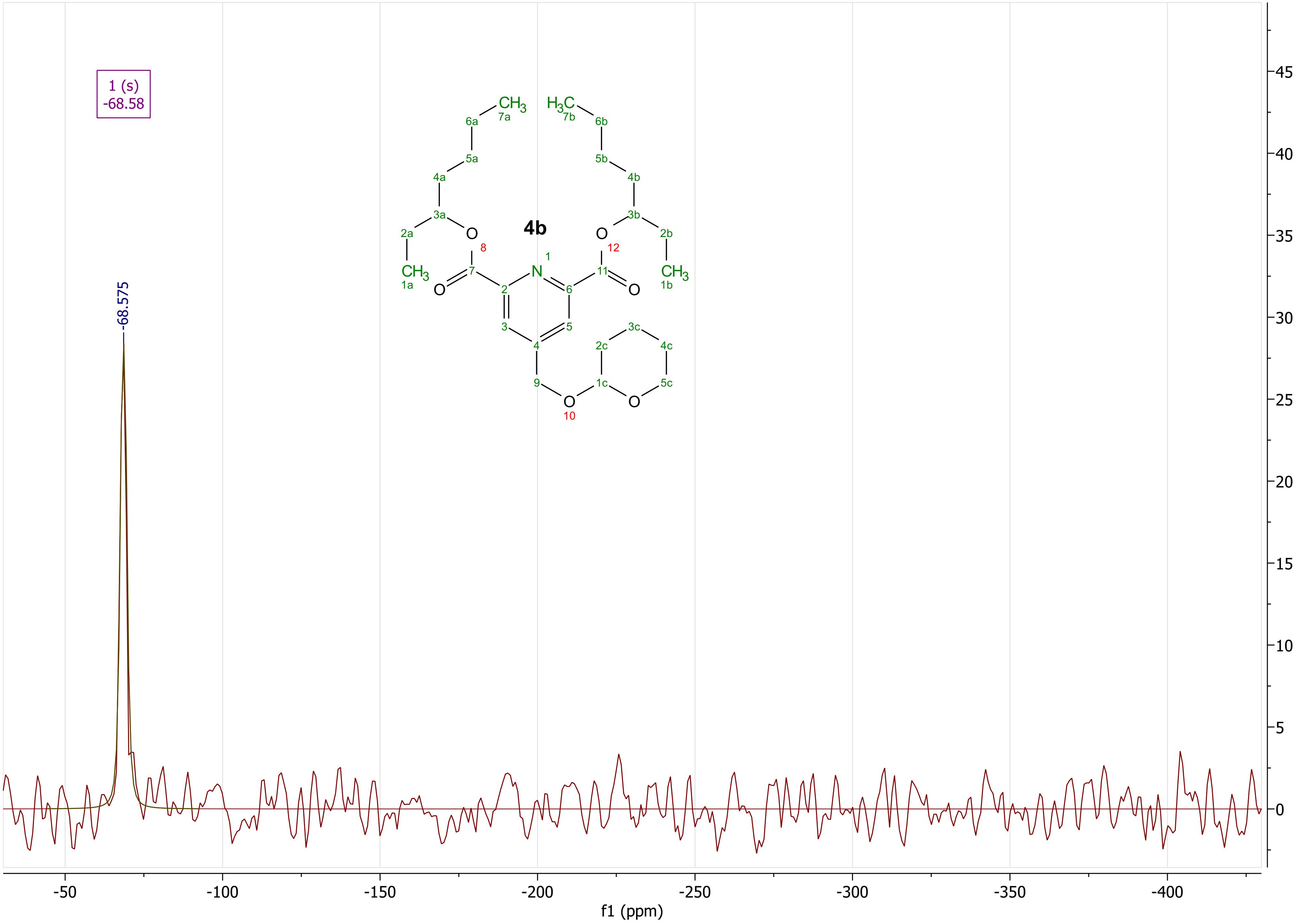

^{1}H NMR (400 MHz, $CDCl_3$) δ 8.27 (t, *J* = 0.9 Hz, 2H), 7.74 (s, 2H), 7.67 (d, *J* = 7.6 Hz, 2H), 7.59 (d, *J* = 8.0 Hz, 2H), 7.48 (t, *J* = 7.7 Hz, 2H), 5.48 (s, 4H), 4.87 (s, 2H), 2.43 (br s, 1H).

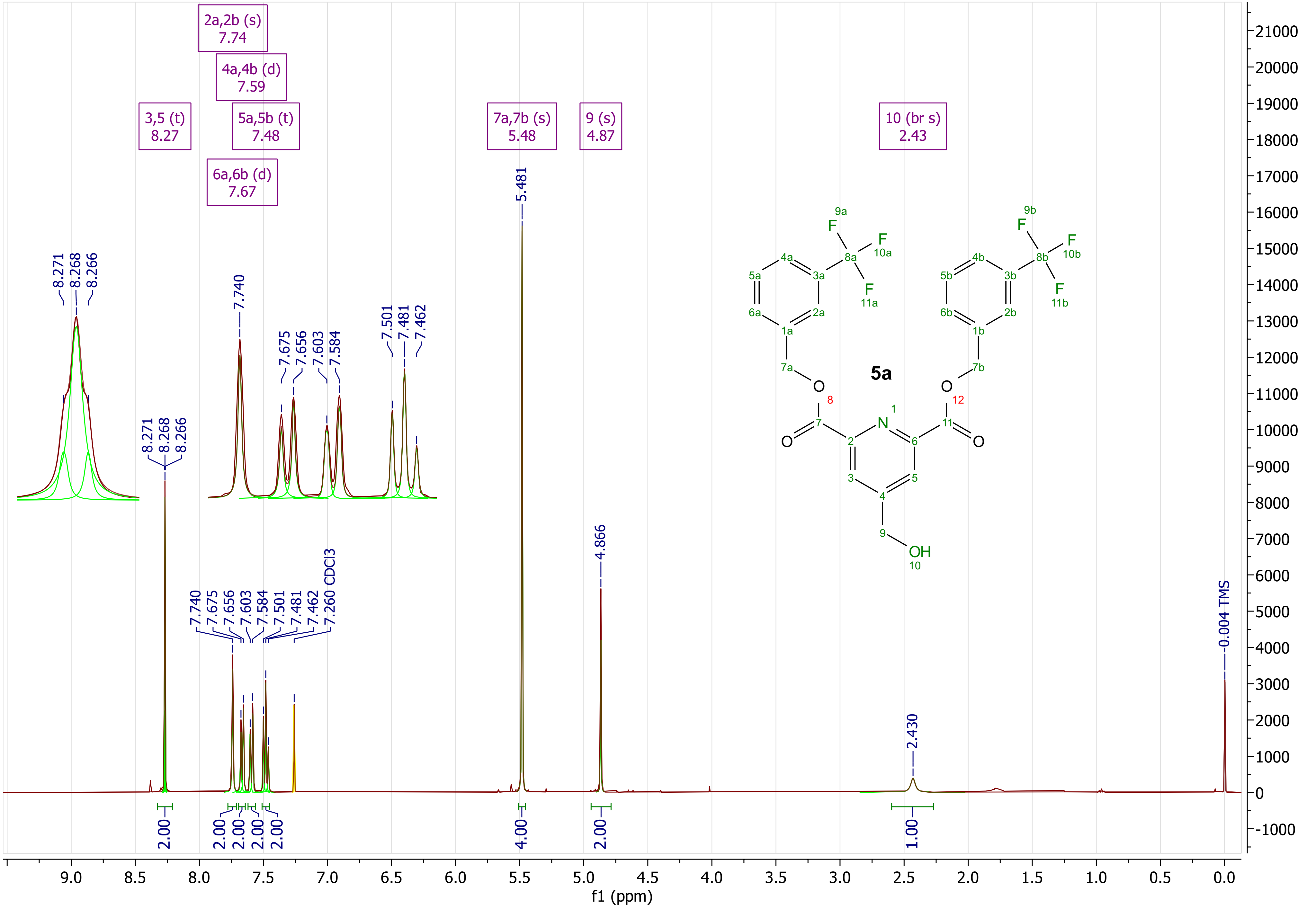

[Back ↑]

^{13}C NMR (101 MHz, $CDCl_3$) δ 164.5 (2C), 153.6, 148.4 (2C), 136.5 (2C), 132.0 (app q, *J* = 1.4 Hz, 2C), 131.2 (q, *J* = 32.6 Hz, 2C), 129.3 (2C), 125.6 (2C), 125.6 – 125.3 (m, 4C), 124.1 (q, *J* = 272.4 Hz, 2C), 67.0 (2C), 62.9.

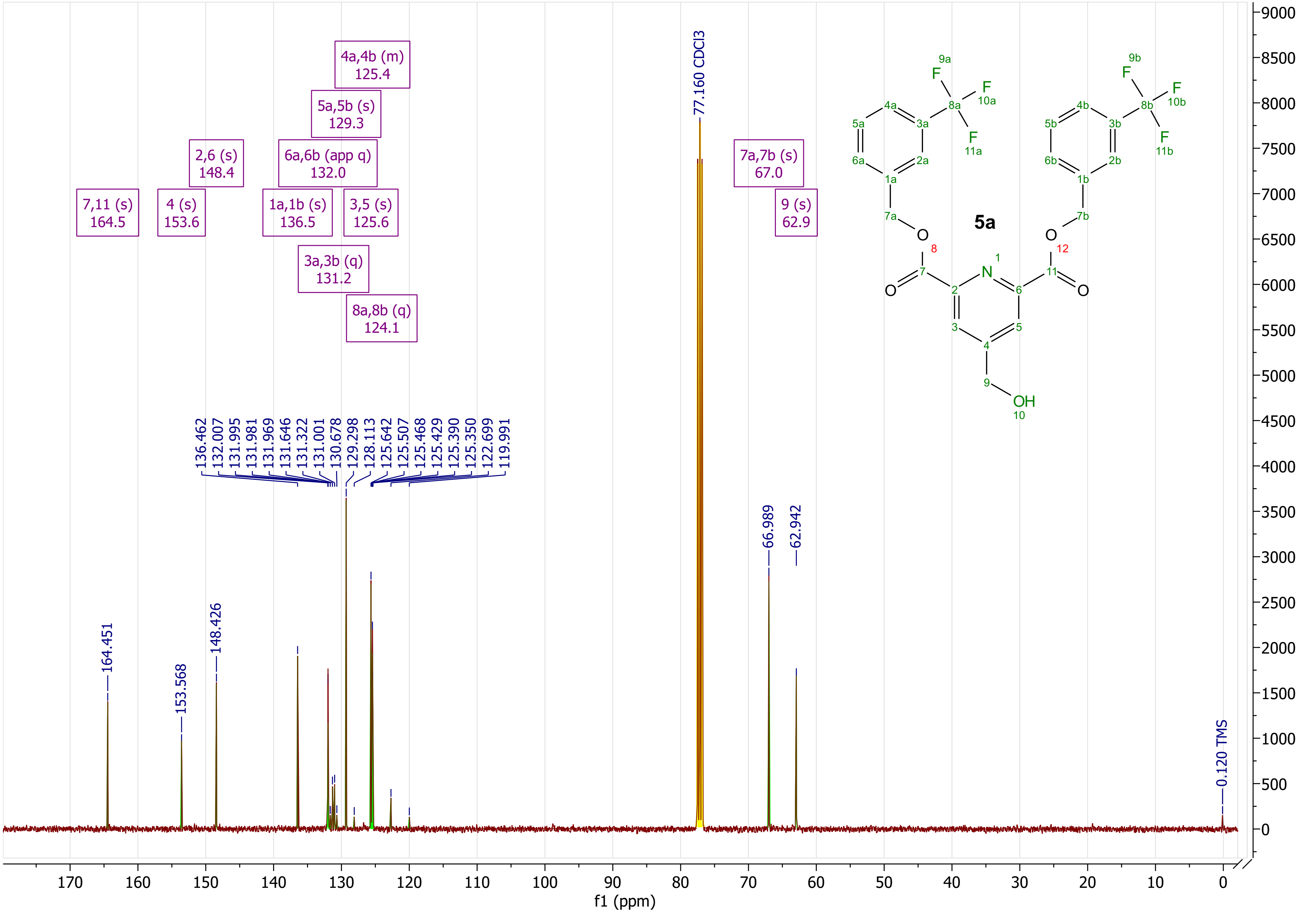

[Back ↑]

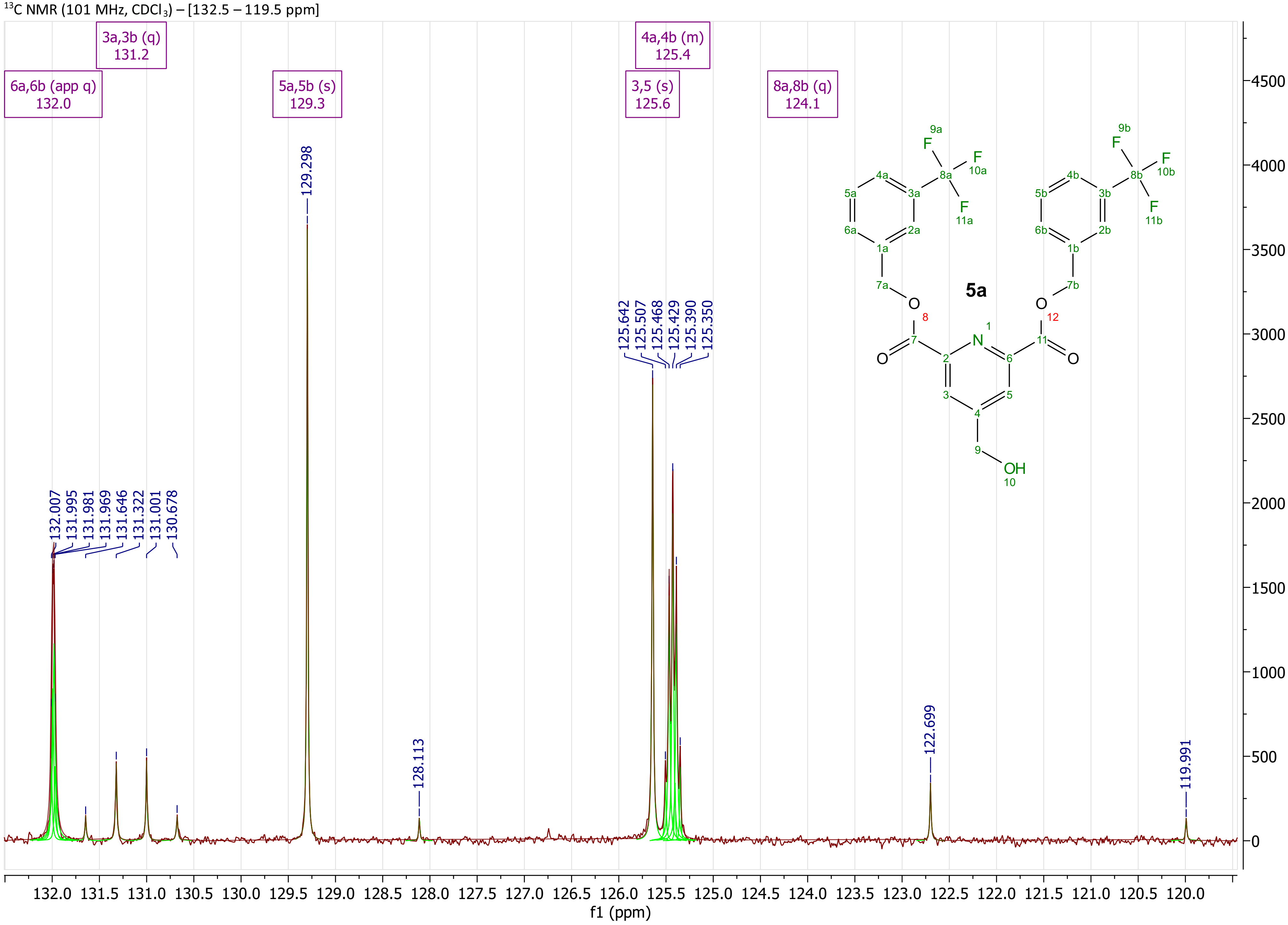

^{13}C NMR (101 MHz, CDCl$_3$) – [132.5 – 119.5 ppm]
6a,6b (app q) 132.0
3a,3b (q) 131.2
5a,5b (s) 129.3
4a,4b (m) 125.4
3,5 (s) 125.6
8a,8b (q) 124.1
132.007
131.995
131.981
131.969
131.646
131.322
131.001
130.678
129.298
128.113
125.642
125.507
125.468
125.429
125.390
125.350
122.699
119.991
5a
f1 (ppm)

[Back ↑]

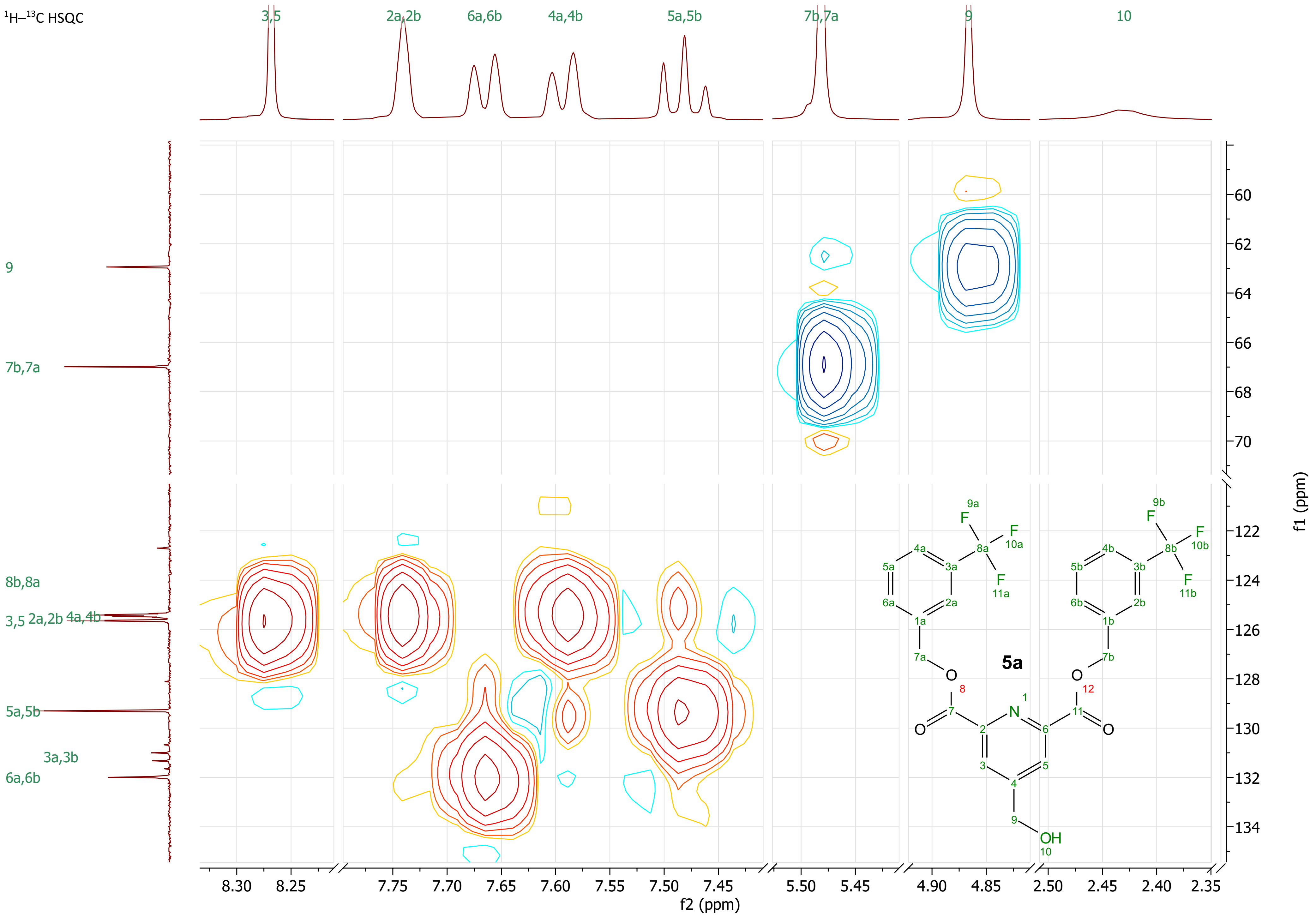

$^{1}H–^{13}C$ HSQC
3,5
2a,2b
6a,6b
4a,4b
5a,5b
7b,7a
9
10
9
7b,7a
8b,8a
3,5 2a,2b 4a,4b
5a,5b
3a,3b
6a,6b
5a
f1 (ppm)
f2 (ppm)

[Back ↑]

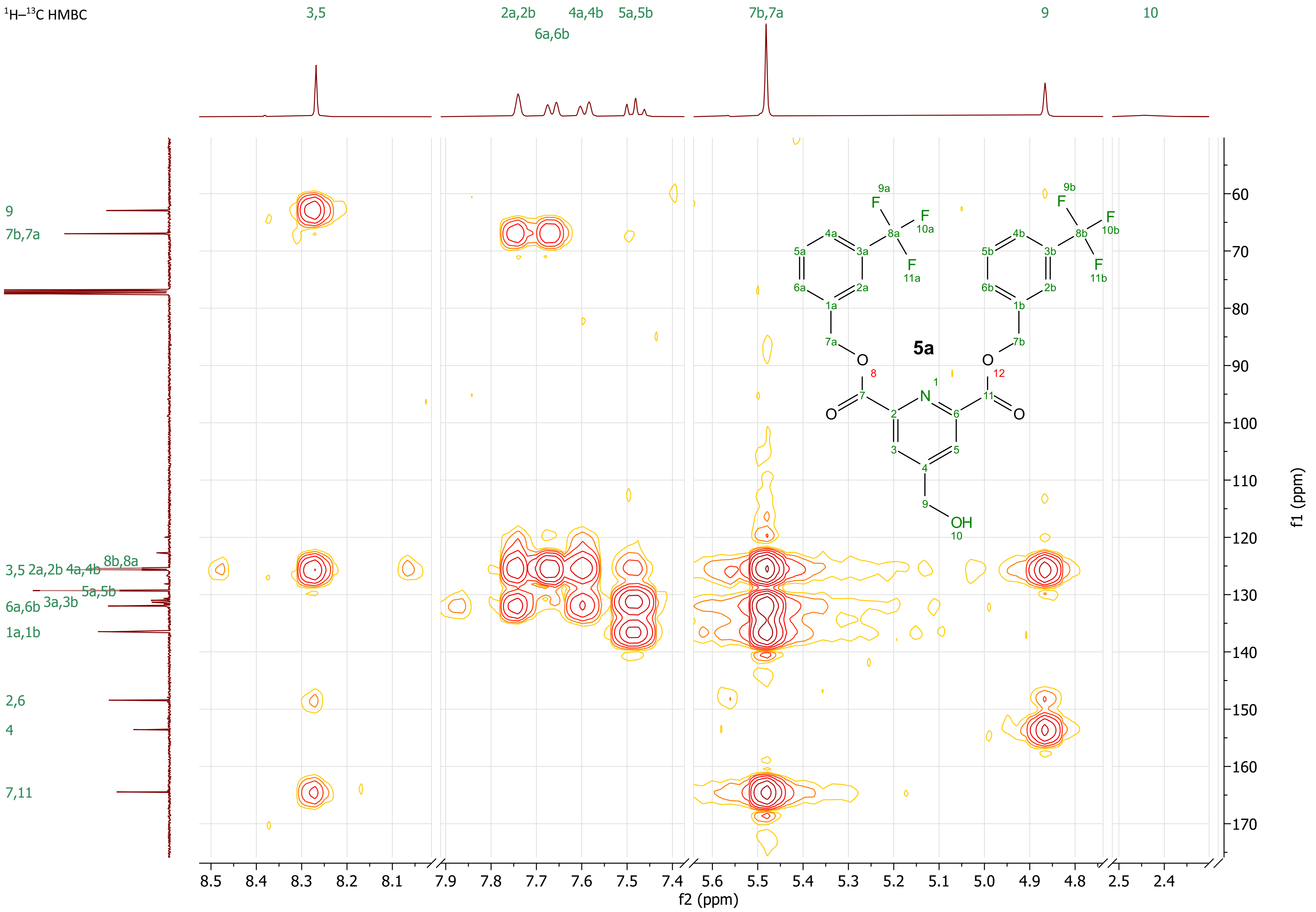
$^{1}H-^{13}C$ HMBC
3,5
2a,2b
6a,6b
4a,4b
5a,5b
7b,7a
9
10
9
7b,7a
3,5 2a,2b 4a,4b
8b,8a
5a,5b
6a,6b 3a,3b
1a,1b
2,6
4
7,11
5a
f2 (ppm)
f1 (ppm)

^{1}H–^{15}N HMBC

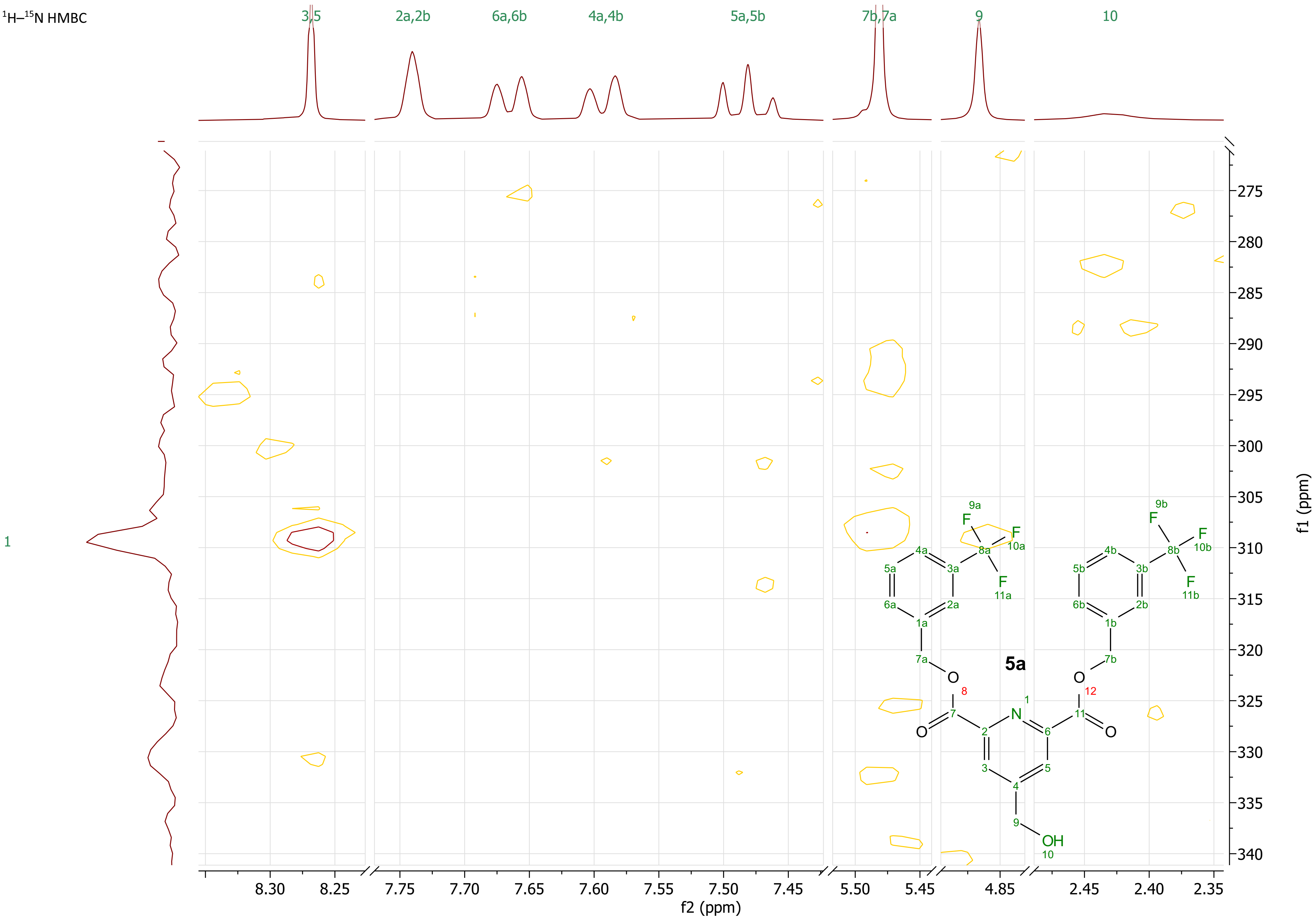

^{15}N NMR (41 MHz, $CDCl_3$) δ 309.33. – Projection f1

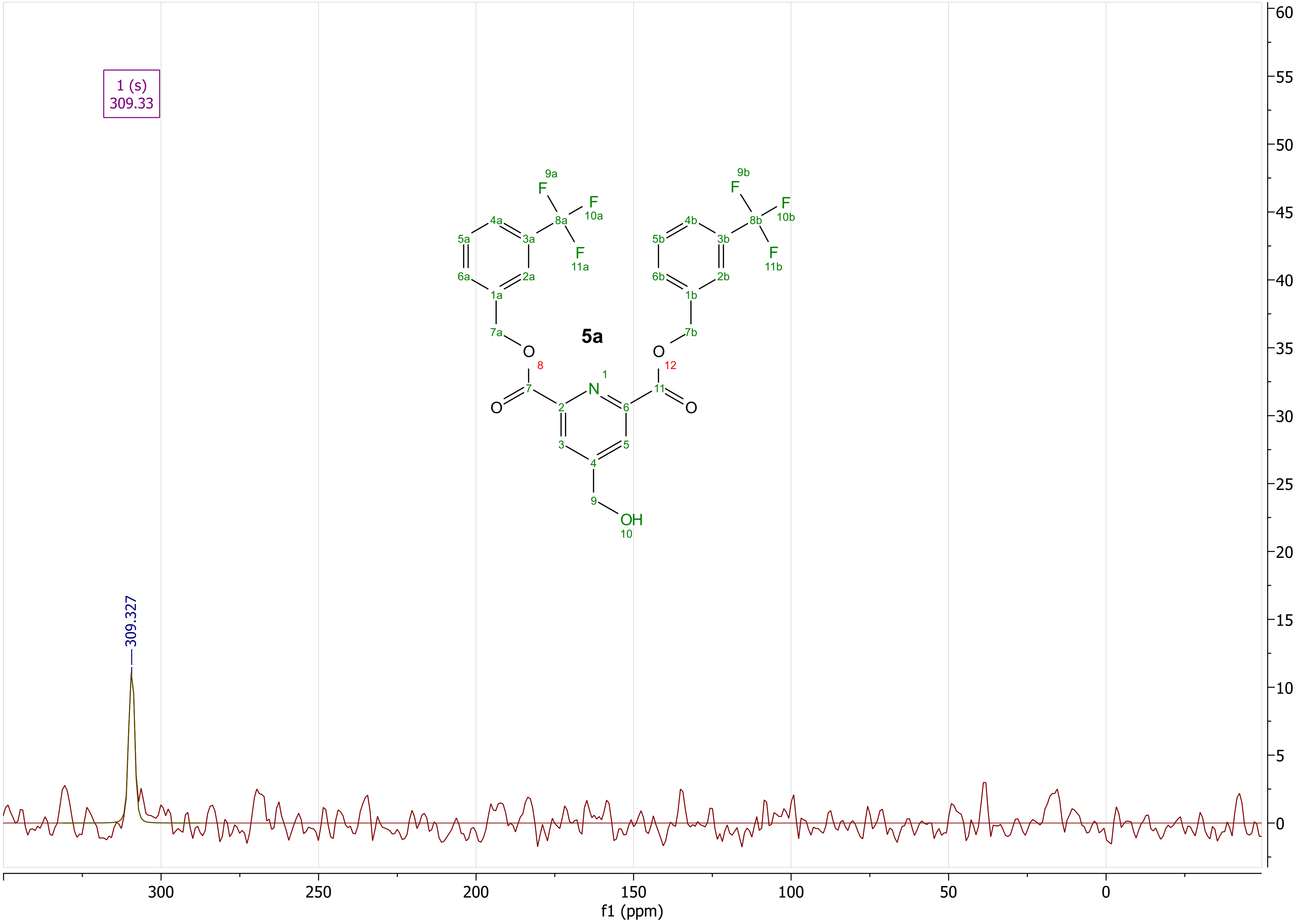

[Back ↑]

^{19}F NMR (376 MHz, $CDCl_3$) δ -62.66.

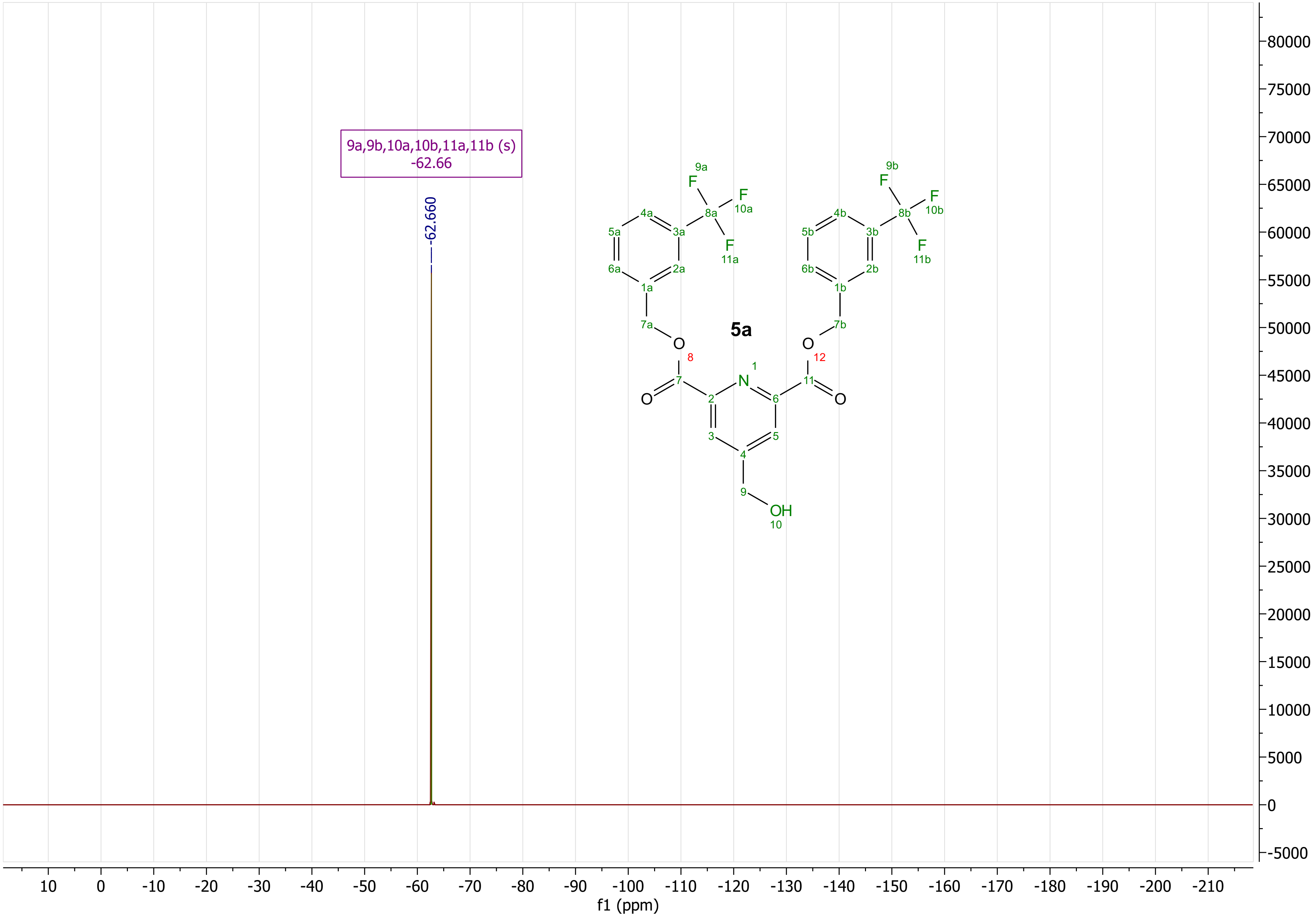

^{1}H NMR (400 MHz, $CDCl_3$) δ 8.18 (app t, *J* = 0.9 Hz, 2H), 5.20 – 5.07 (m, 2H), 4.89 (s, 2H), 2.40 (br s, 1H), 1.81 – 1.60 (m, 8H), 1.46 – 1.25 (m, 8H), 0.96 (t, *J* = 7.4 Hz, 6H), 0.88 (app t, *J* = 7.2 Hz, 6H).

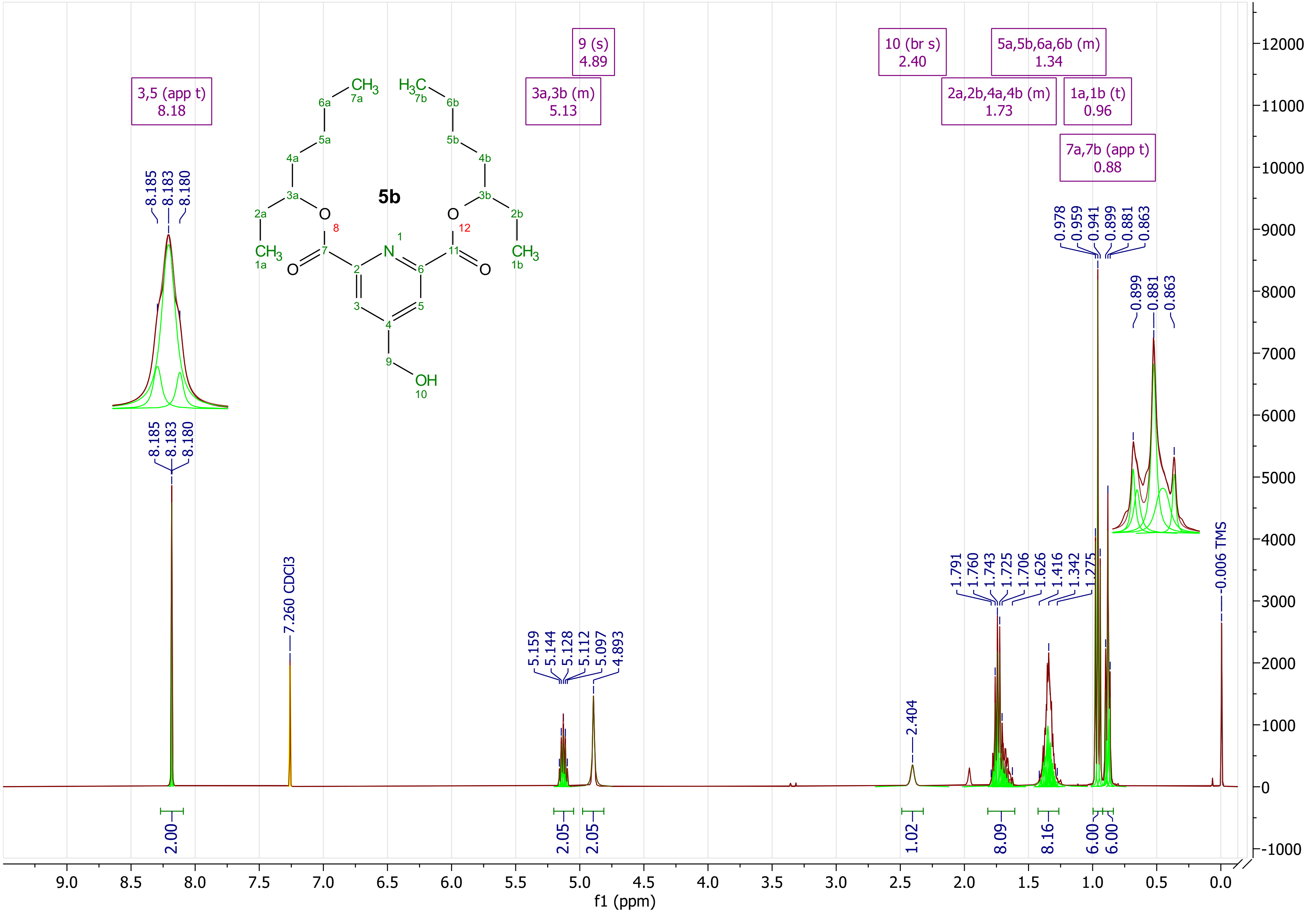

^{13}C NMR (101 MHz, $CDCl_3$) δ 164.6 (2C), 152.9, 149.4 (2C), 124.8 (2C), 78.0 (2C), 63.2, 33.4 (2C), 27.7 (2C), 27.1 (2C), 22.7 (2C), 14.1 (2C), 9.8 (2C).

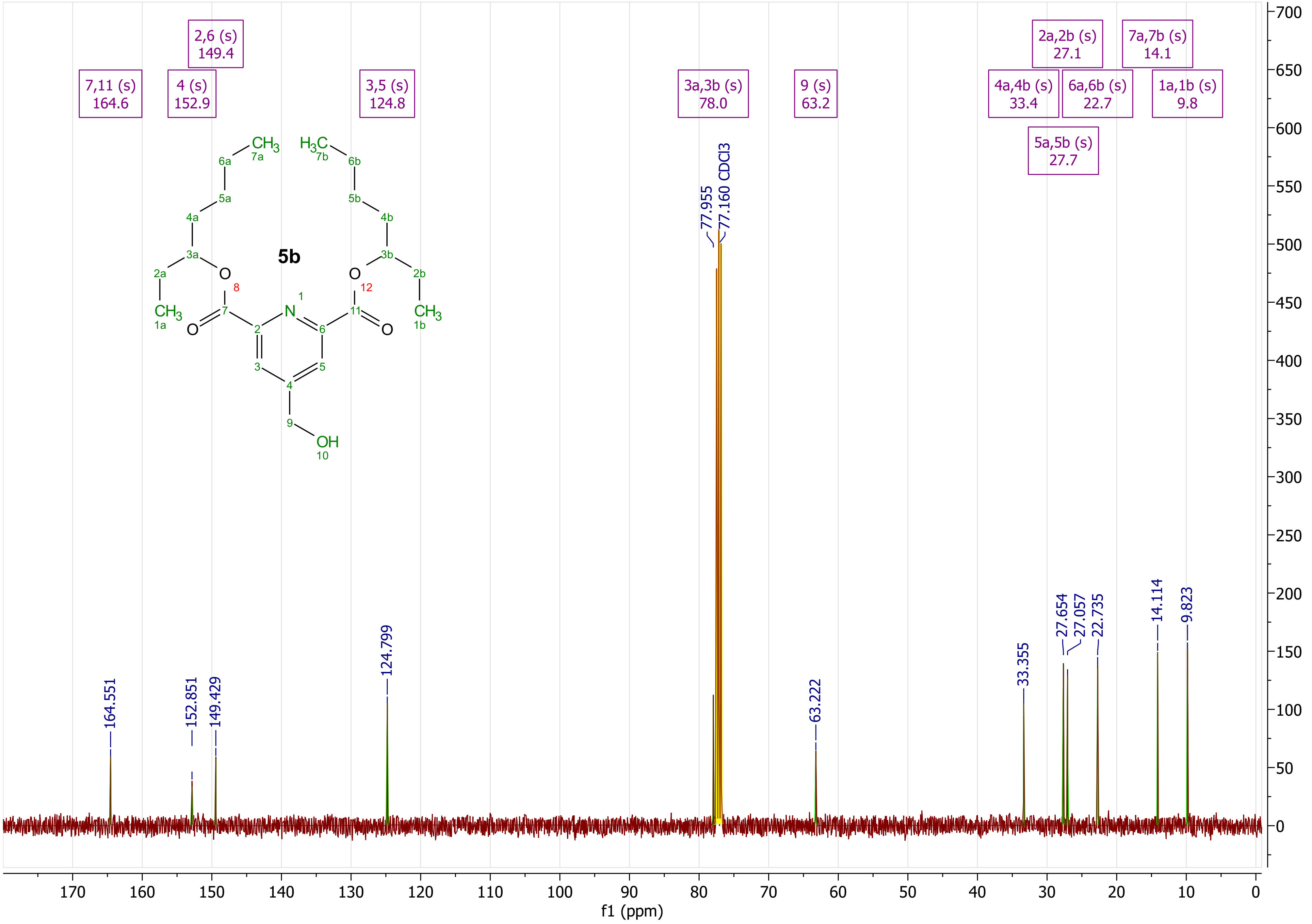

[Back ↑]

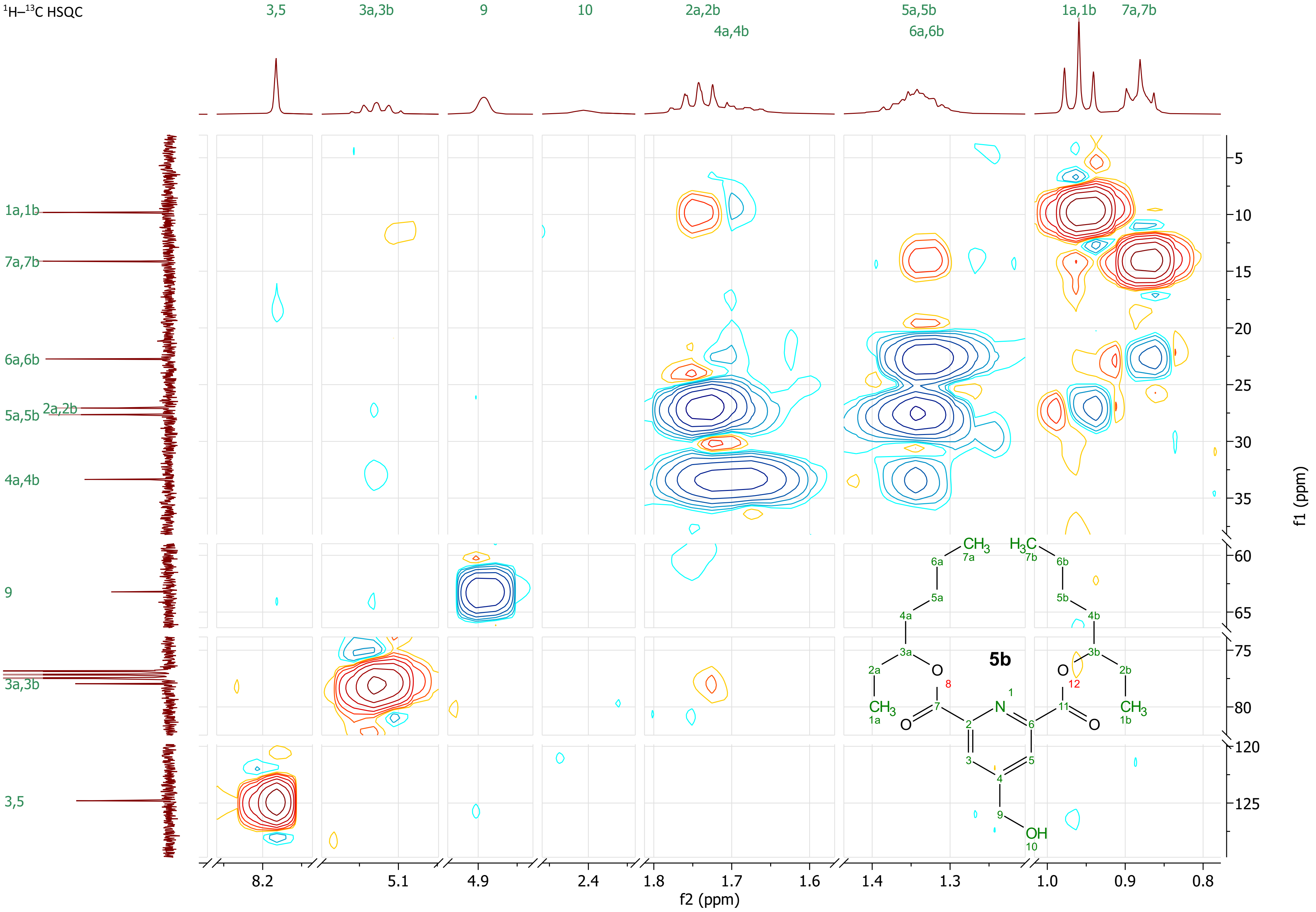

^{1}H–^{13}C HSQC
3,5
3a,3b
9
10
2a,2b
4a,4b
5a,5b
6a,6b
1a,1b
7a,7b
9
3a,3b
3,5
5b
f1 (ppm)
f2 (ppm)

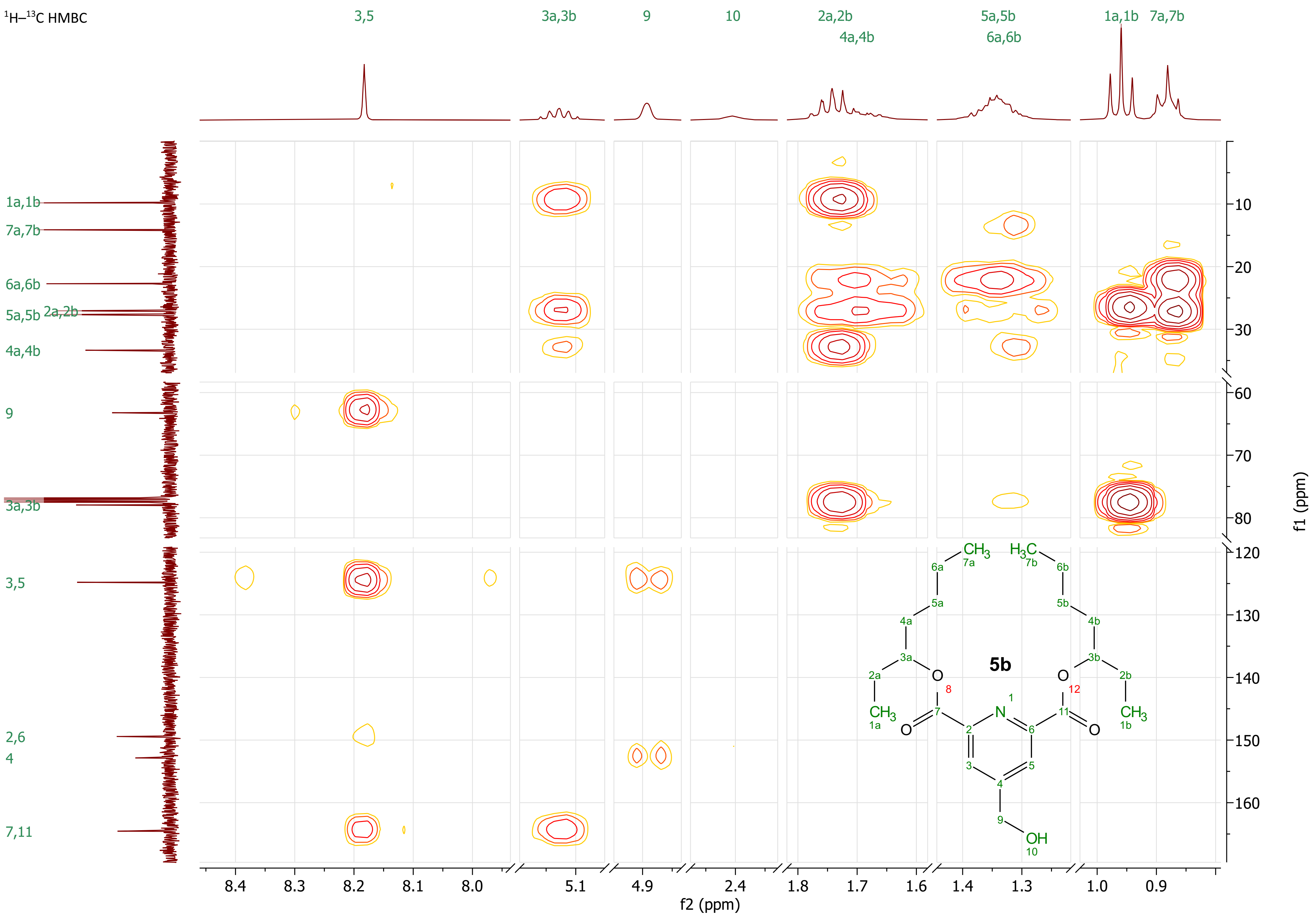

1H–13C HMBC
3,5
3a,3b
9
10
2a,2b
4a,4b
5a,5b
6a,6b
1a,1b
7a,7b
1a,1b
7a,7b
6a,6b
5a,5b
2a,2b
4a,4b
9
3a,3b
3,5
2,6
4
7,11
5b
CH3
H3C
OH
f1 (ppm)
f2 (ppm)

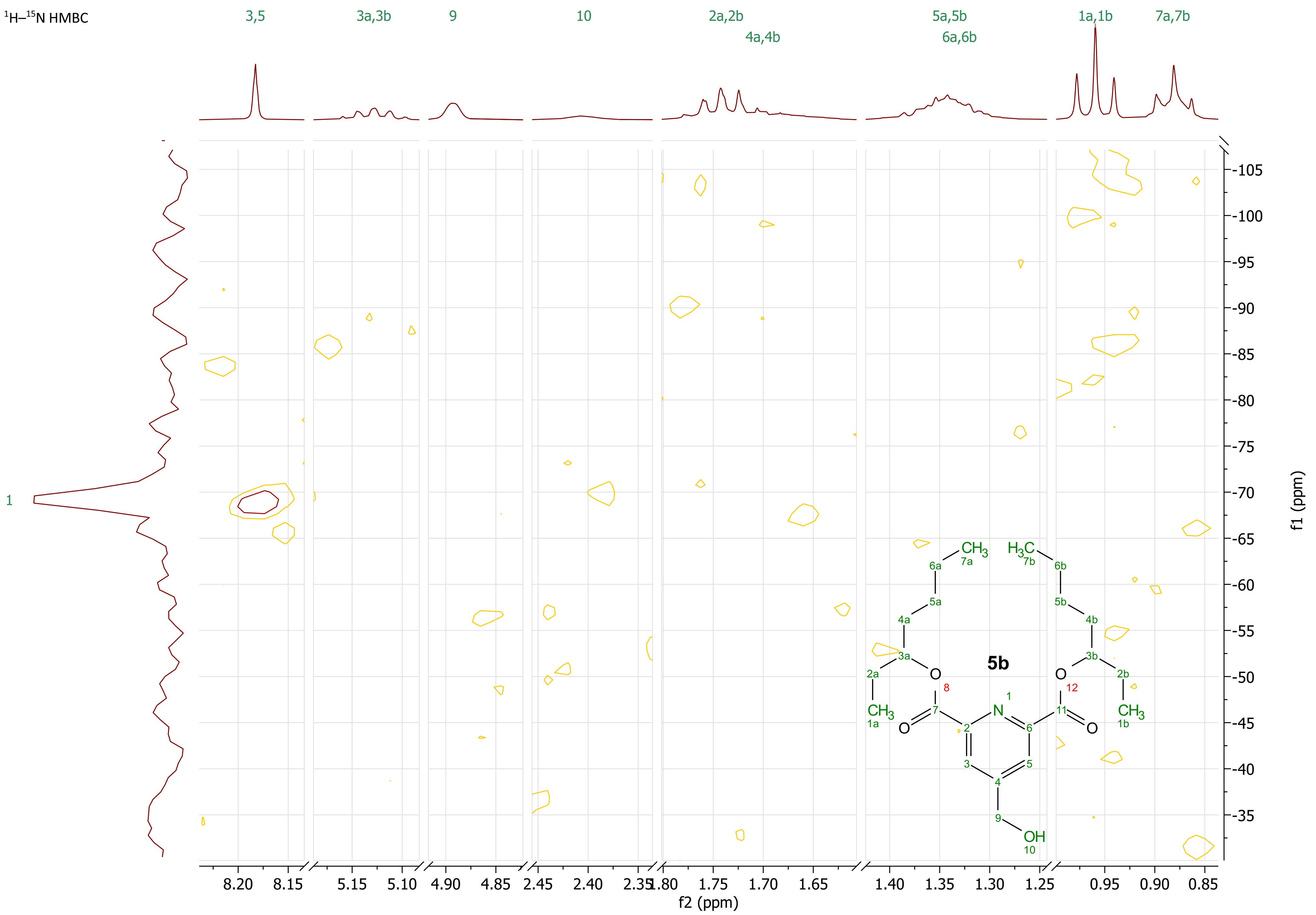

$^{1}H–^{15}N$ HMBC
3,5
3a,3b
9
10
2a,2b
4a,4b
5a,5b
6a,6b
1a,1b
7a,7b
1
5b
f2 (ppm)
f1 (ppm)

[Back ↑]

^{15}N NMR (41 MHz, $CDCl_3$) δ -69.19. – Projection f1

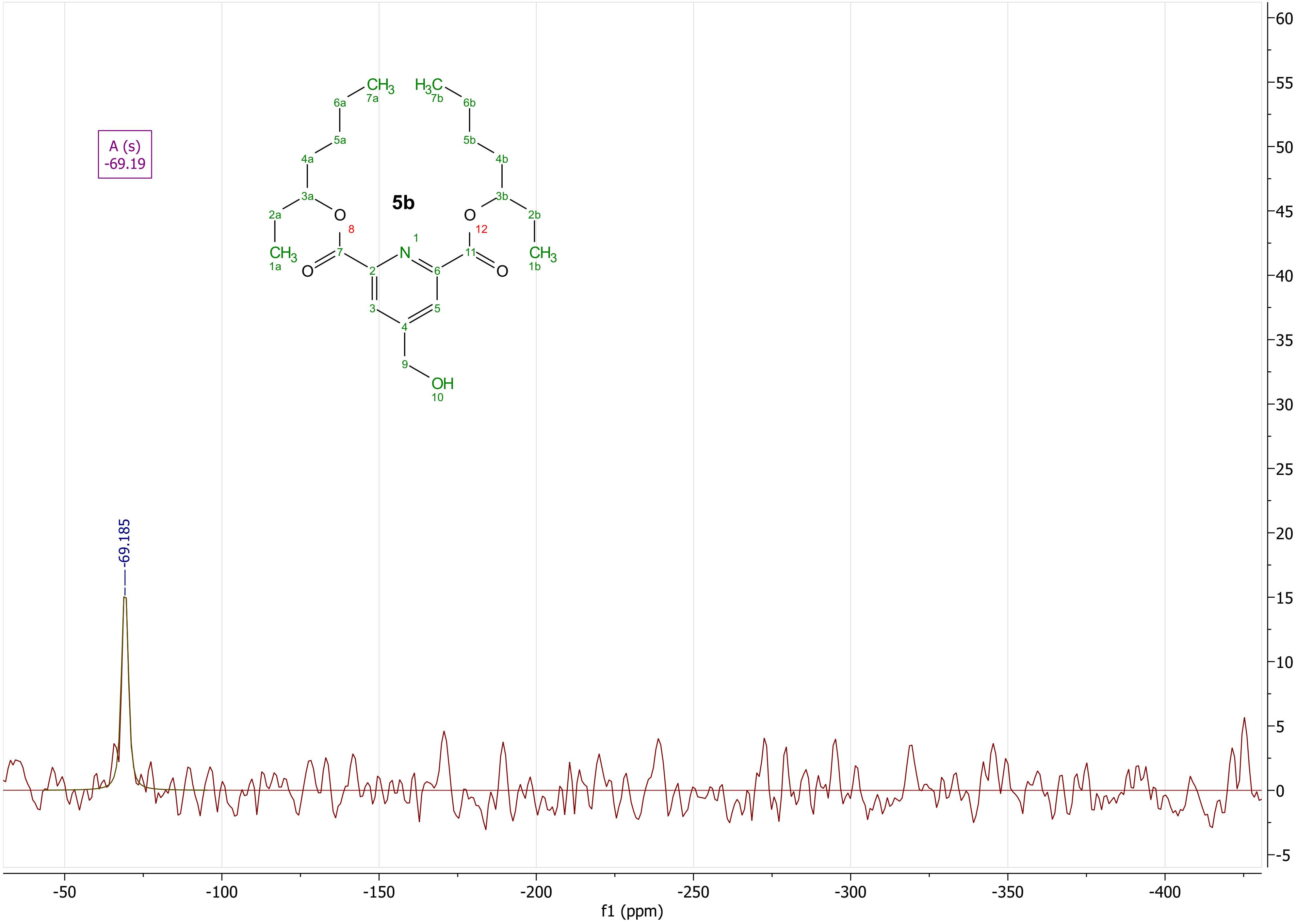

^{1}H NMR (400 MHz, $CDCl_3$) δ 8.36 (app t, *J* = 0.9 Hz, 2H), 8.31 (t, *J* = 6.4 Hz, 2H), 7.48 (s, 4H), 7.49 – 7.44 (m, 2H), 7.41 – 7.33 (m, 2H), 4.89 (dt, *J* = 14.4, 0.9 Hz, 1H), 4.74 (t, *J* = 3.4 Hz, 1H), 4.63 (d, *J* = 6.4 Hz, 4H), 4.59 (dt, *J* = 14.4, 0.8 Hz, 1H), 3.89 – 3.79 (m, 1H), 3.60 – 3.50 (m, 1H), 1.93 – 1.79 (m, 1H), 1.82 – 1.64 (m, 2H), 1.67 – 1.48 (m, 3H).

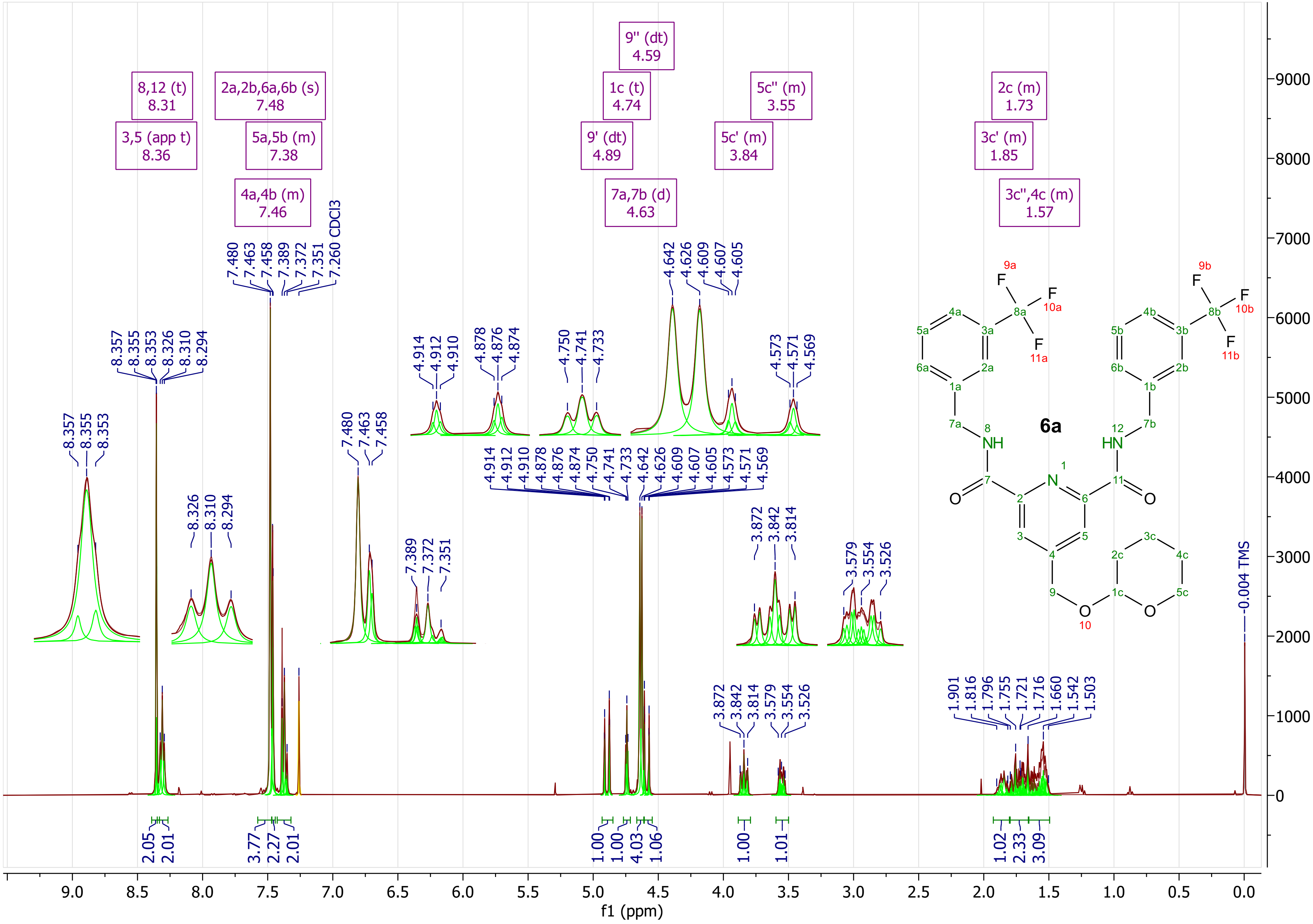

^{13}C NMR (101 MHz, $CDCl_3$) δ 163.9 (2C), 152.3, 148.8 (2C), 139.2 (2C), 131.2 (2C), 131.1 (q, *J* = 32.2 Hz, 2C), 129.3 (2C), 124.5 (q, *J* = 3.8 Hz, 2C), 124.4 (q, *J* = 3.8 Hz, 2C), 124.0 (q, *J* = 271.8 Hz, 2C), 123.6 (2C), 98.7, 66.9, 62.3, 43.1 (2C), 30.4, 25.4, 19.2.

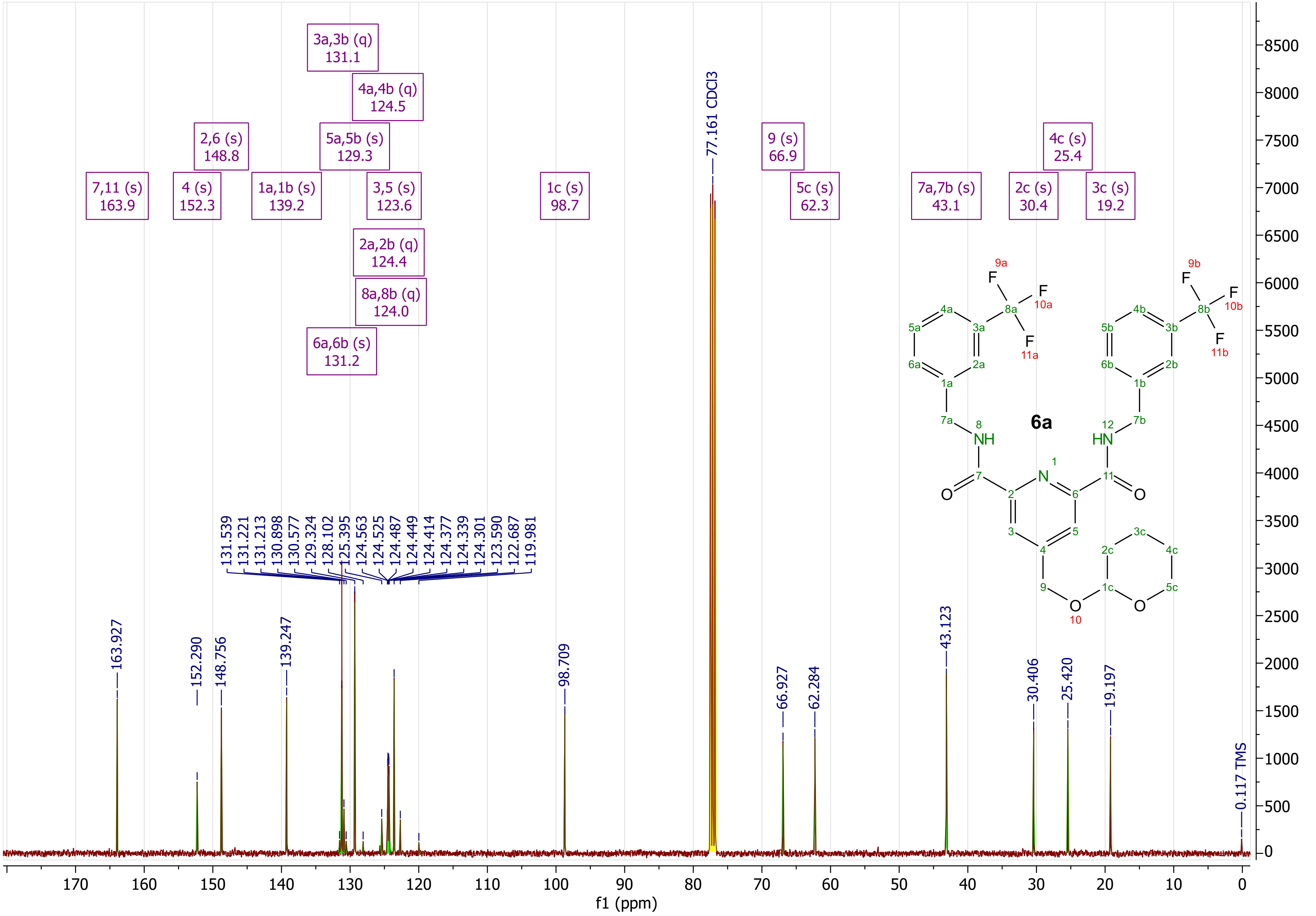

^{13}C NMR (101 MHz, $CDCl_3$) – [132.5 – 119.5 ppm]

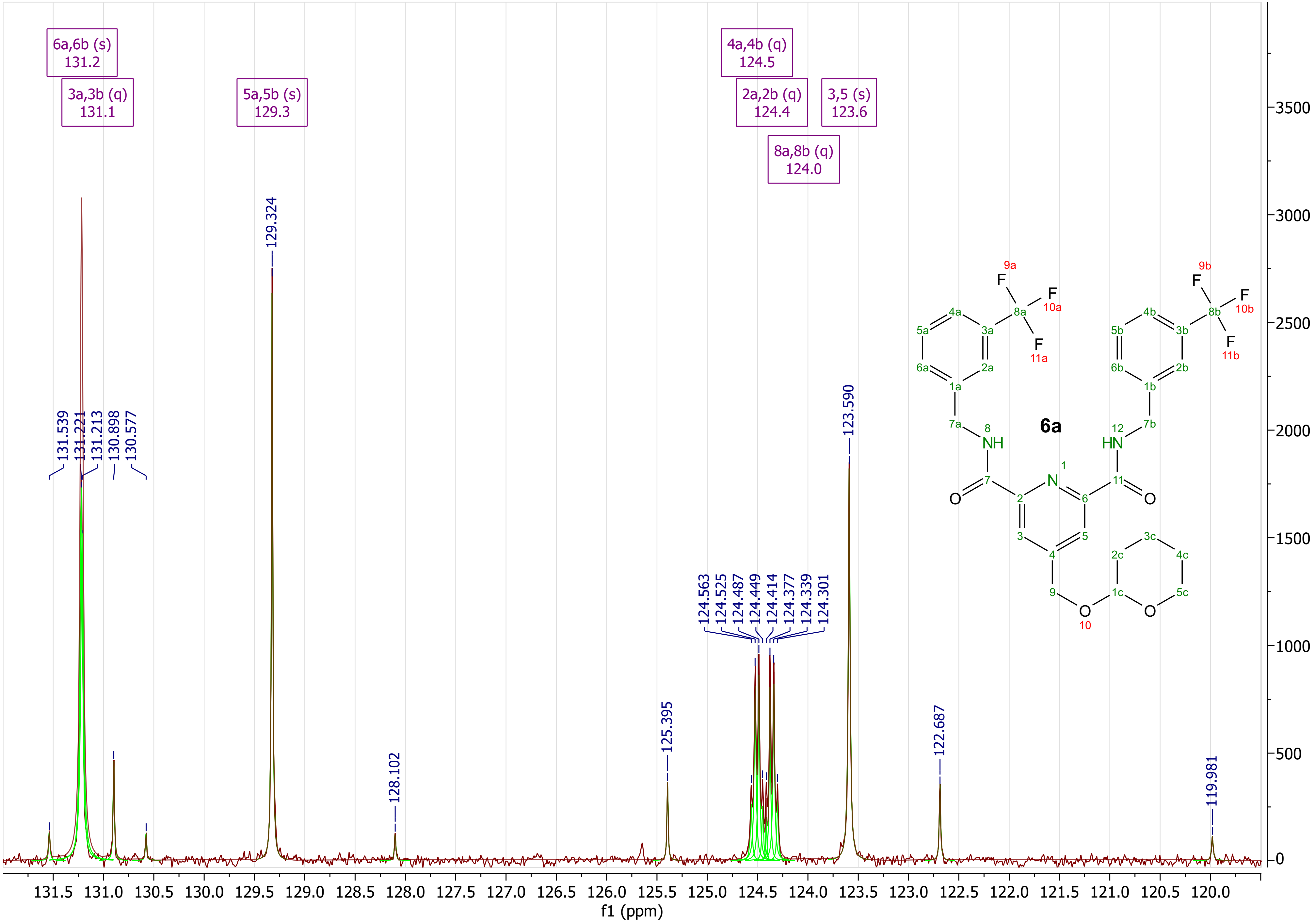

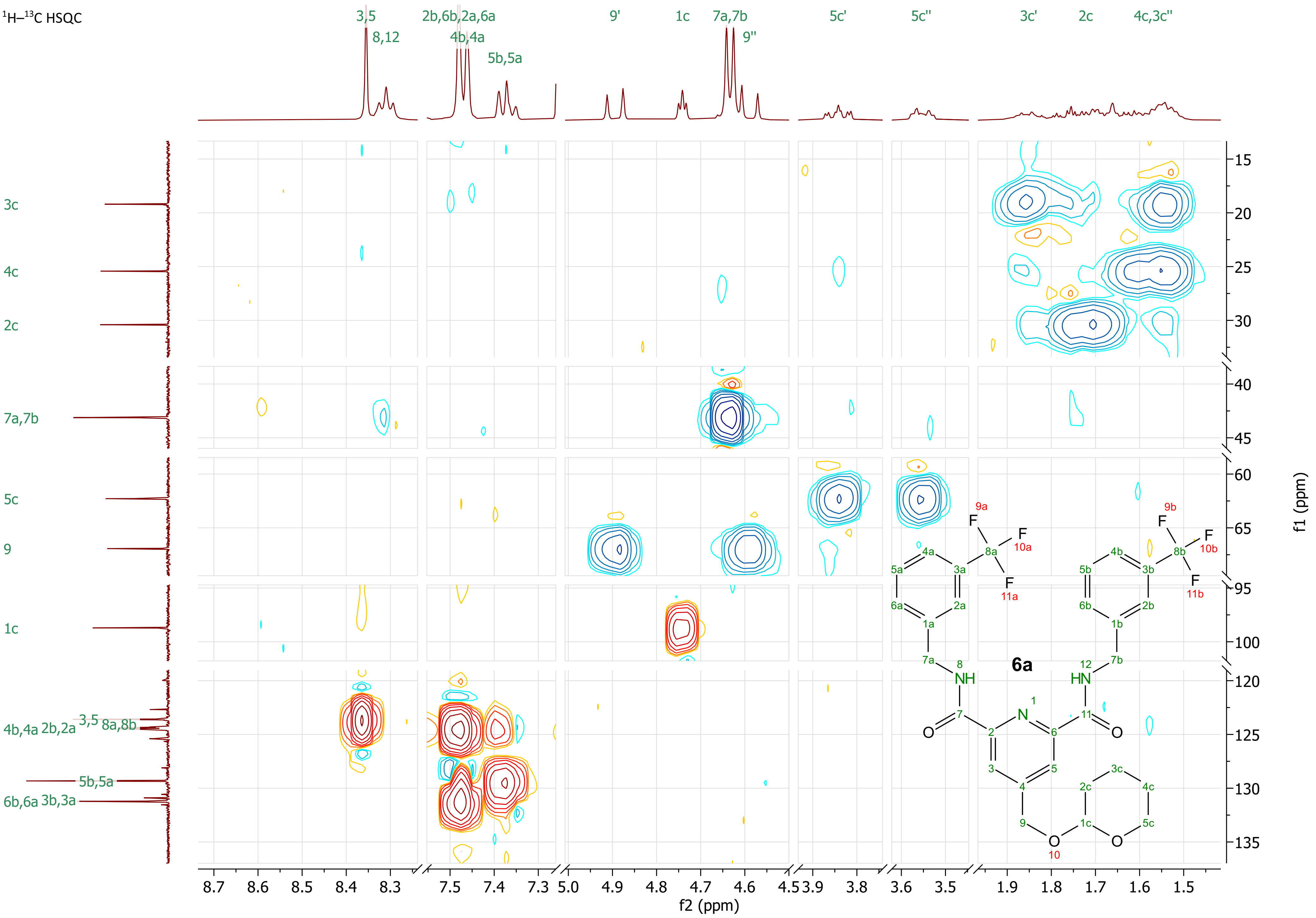

¹H–¹³C HSQC
3,5
8,12
2b,6b,2a,6a
4b,4a
5b,5a
9'
1c
7a,7b
9''
5c'
5c''
3c'
2c
4c,3c''
3c
4c
2c
7a,7b
5c
9
1c
4b,4a 2b,2a
3,5
8a,8b
5b,5a
6b,6a 3b,3a
f2 (ppm)
f1 (ppm)
6a

[Back ↑]

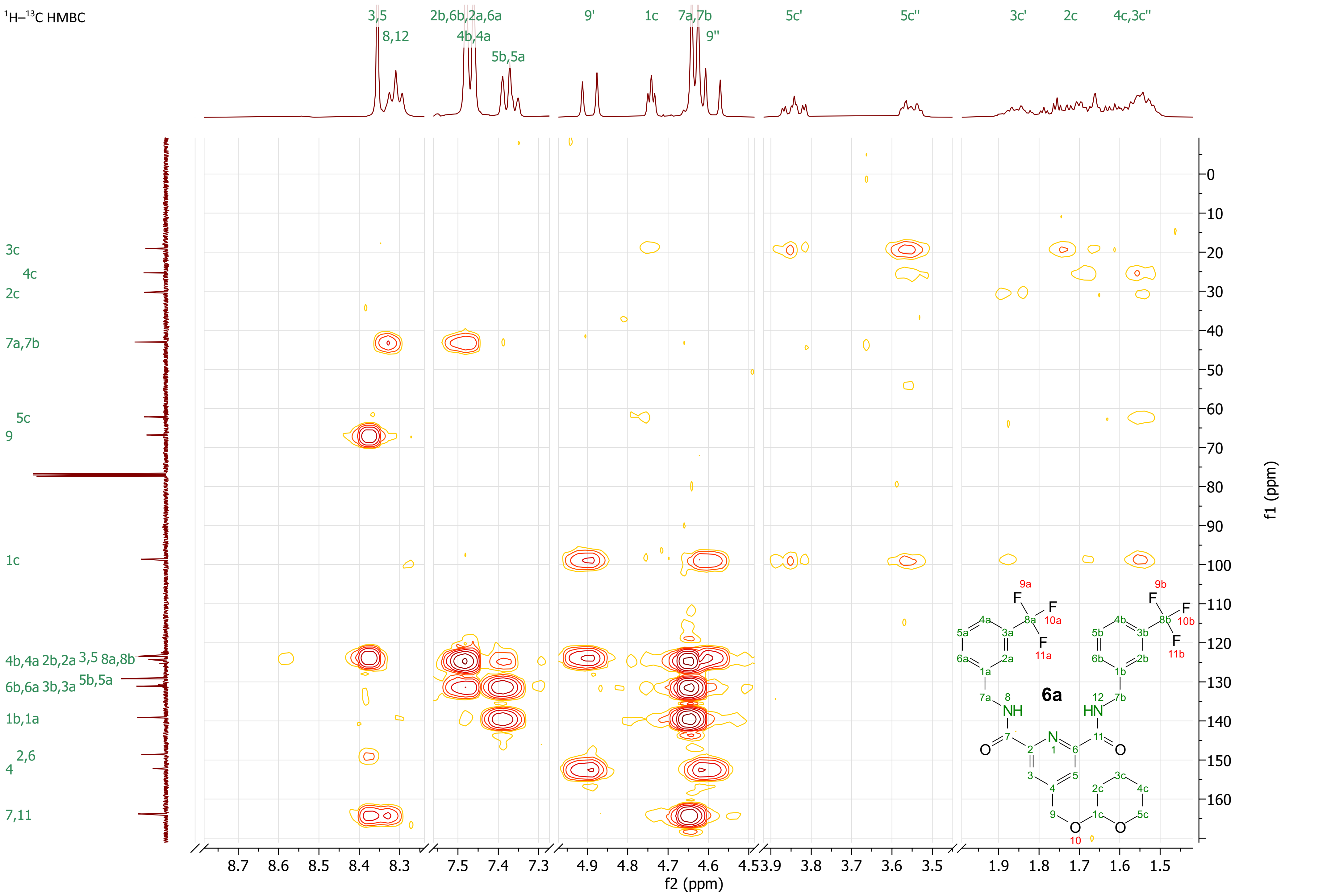

^{1}H–^{13}C HMBC
3,5
8,12
2b,6b,2a,6a
4b,4a
5b,5a
9'
1c
7a,7b
9''
5c'
5c''
3c'
2c
4c,3c''
3c
4c
2c
7a,7b
5c
9
1c
4b,4a 2b,2a 3,5 8a,8b
6b,6a 3b,3a 5b,5a
1b,1a
2,6
4
7,11
6a
f2 (ppm)
f1 (ppm)

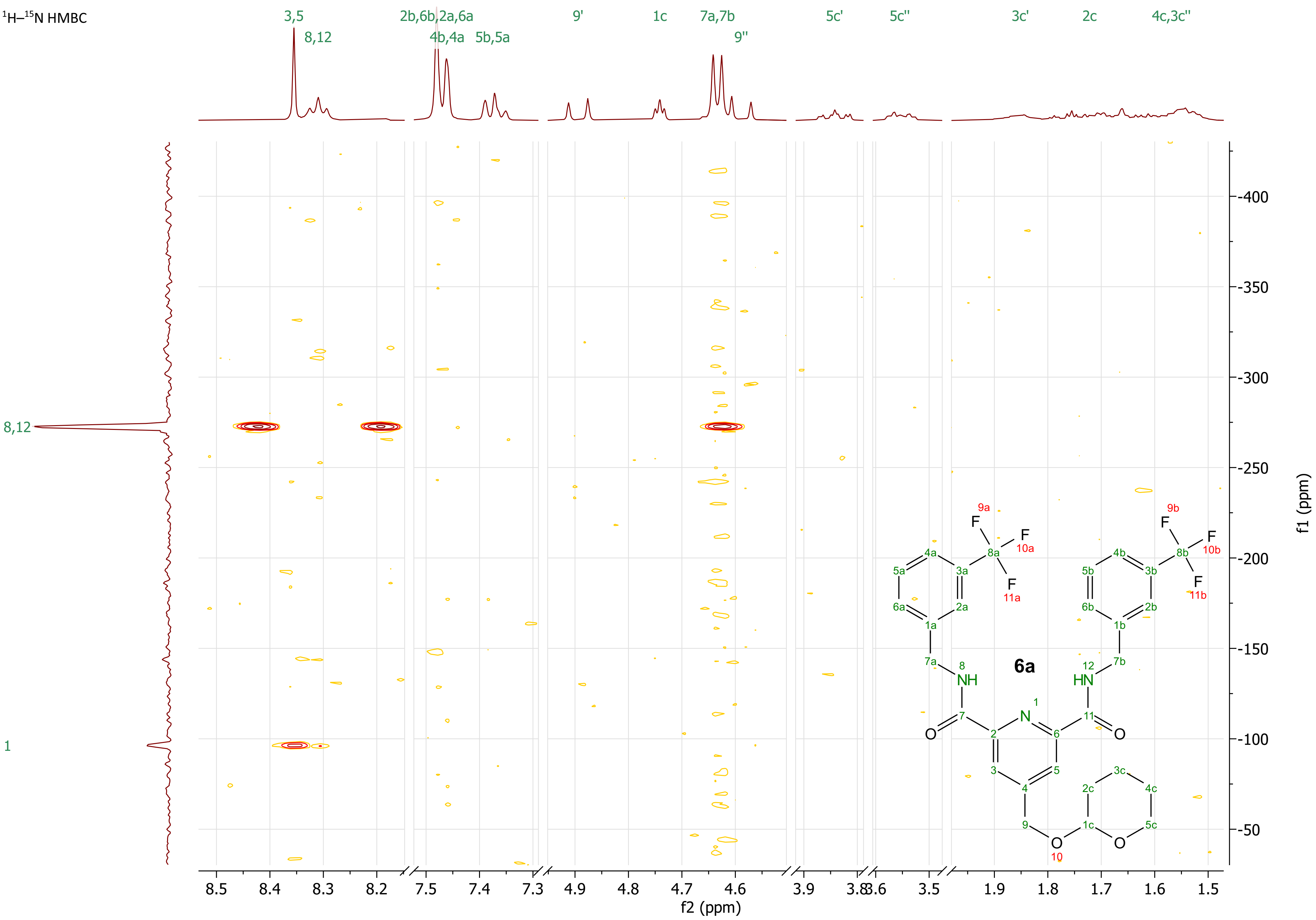
^{1}H–^{15}N HMBC
3,5
8,12
2b,6b,2a,6a
4b,4a
5b,5a
9'
1c
7a,7b
9''
5c'
5c''
3c'
2c
4c,3c''
8,12
1
6a
f2 (ppm)
f1 (ppm)

[Back ↑]

^{15}N NMR (41 MHz, $CDCl_3$) δ -96.35, -272.70. – Projection f1

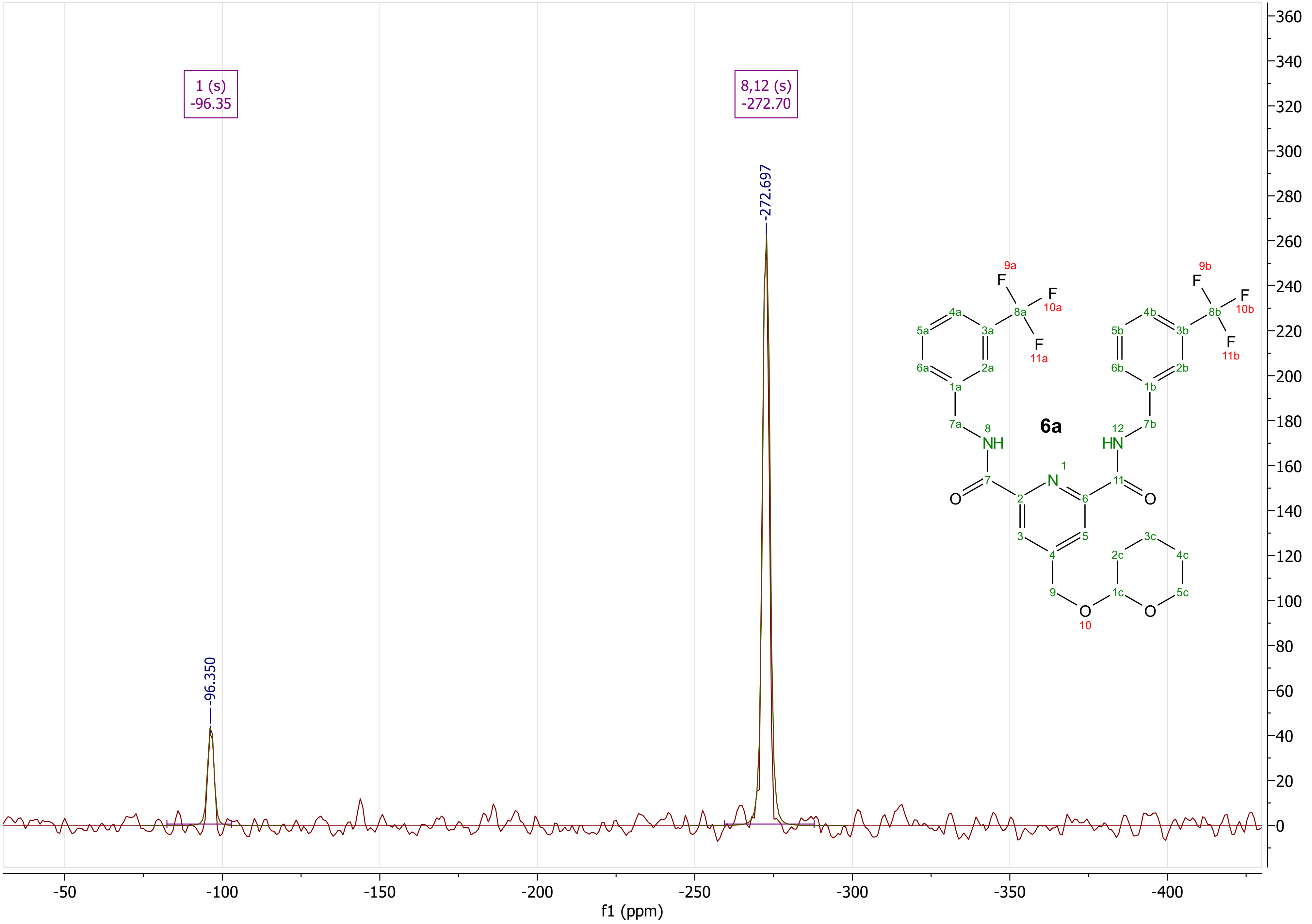

[Back ↑]

^{1}H NMR (400 MHz, $CDCl_3$) δ 8.33 (app t, *J* = 0.9 Hz, 2H), 7.41 (d, *J* = 8.9 Hz, 2H), 4.90 (dt, *J* = 14.2, 0.9 Hz, 1H), 4.75 (t, *J* = 3.4 Hz, 1H), 4.61 (dt, *J* = 14.3, 0.8 Hz, 1H), 4.14 – 3.98 (m, 2H), 3.92 – 3.78 (m, 1H), 3.63 – 3.47 (m, 1H), 1.96 – 1.83 (m, 1H), 1.83 – 1.58 (m, 7H), 1.62 – 1.45 (m, 6H), 1.44 – 1.27 (m, 8H), 0.97 (td, *J* = 7.4, 1.2 Hz, 6H), 0.94 – 0.80 (m, 6H).

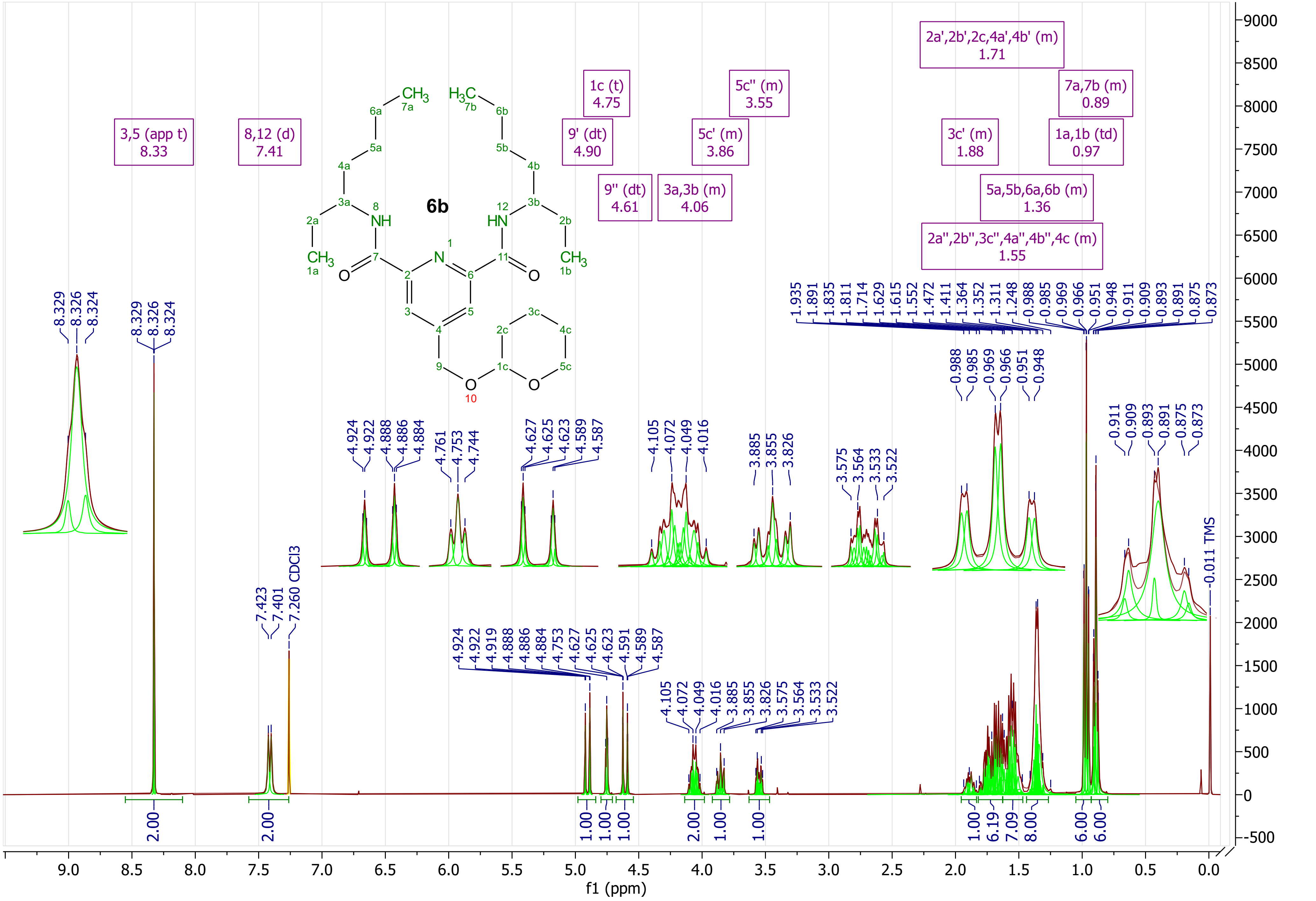

[Back ↑]

^{13}C NMR (101 MHz, $CDCl_3$) δ 163.3 (2C), 152.0, 149.3 (2C), 123.0 (2C), 98.6, 67.0, 62.1, 51.0 (2C), 34.4 (2C), 30.4, 28.2 (2C), 28.1 (d, J = 1.1 Hz, 2C), 25.5, 22.8 (d, J = 1.5 Hz, 2C), 19.1, 14.2 (2C), 10.3 (2C).

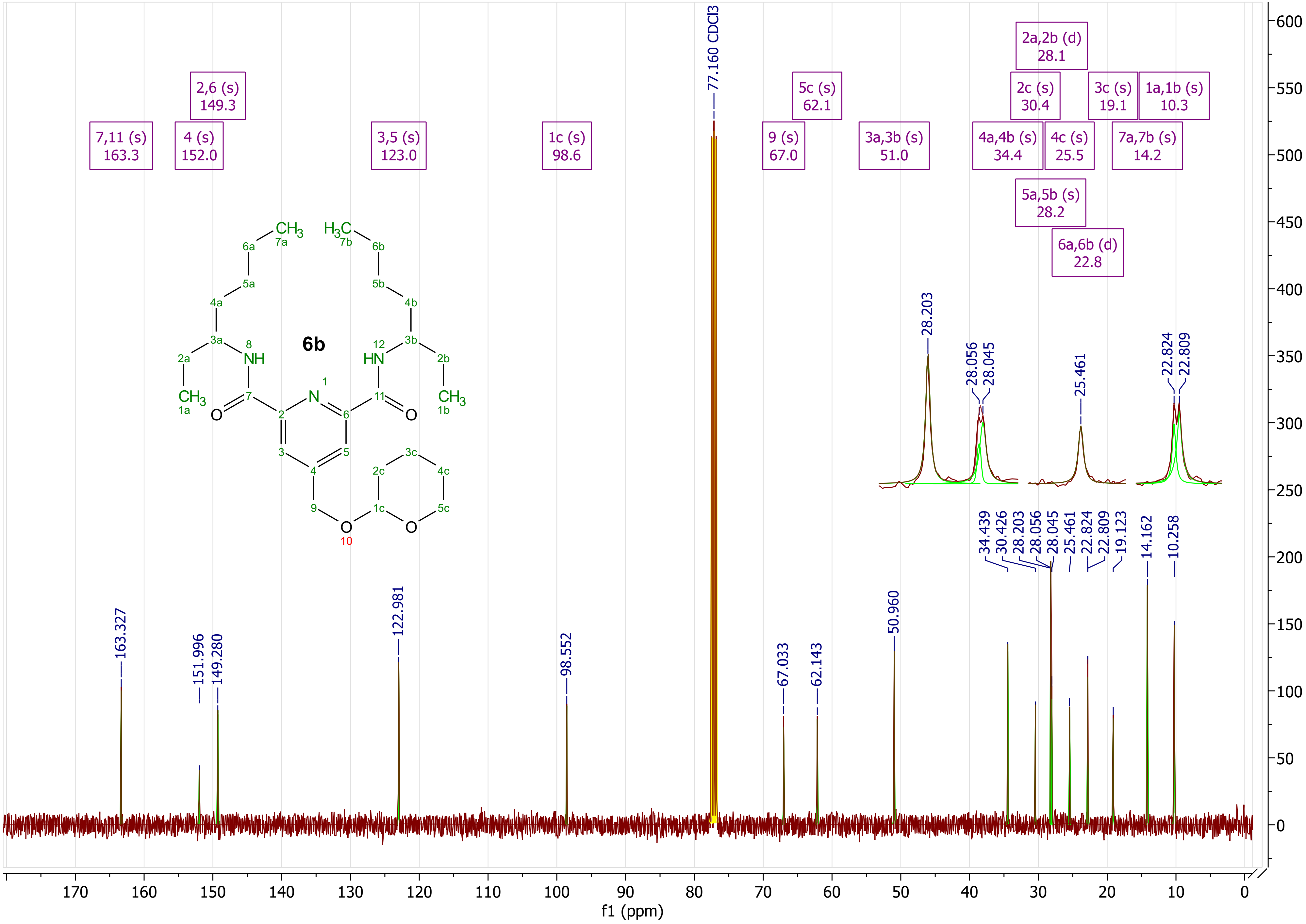

[Back ↑]

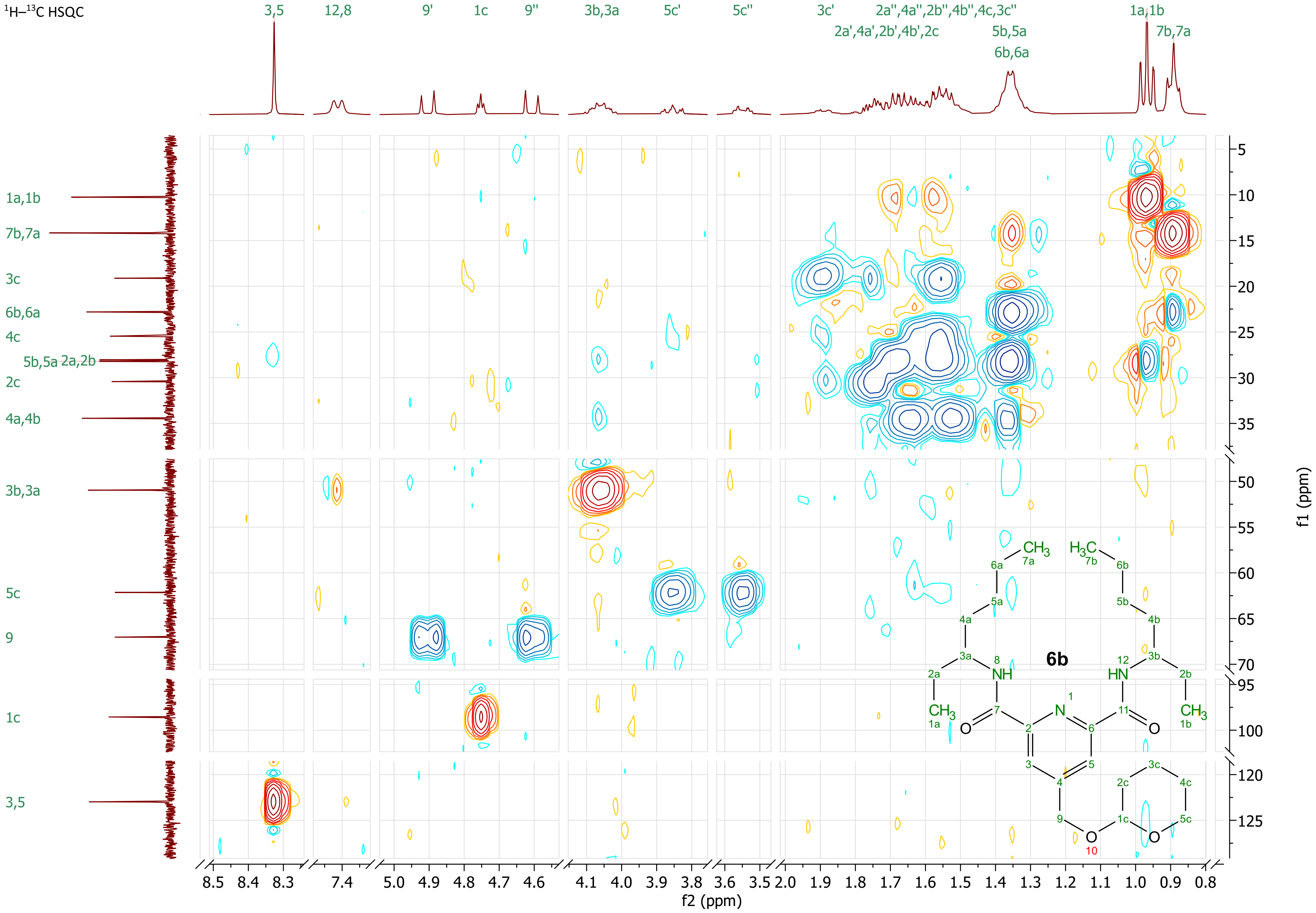

$^{1}H–^{13}C$ HSQC
3,5
12,8
9'
1c
9''
3b,3a
5c'
5c''
3c'
2a'',4a'',2b'',4b'',4c,3c''
2a',4a',2b',4b',2c
5b,5a
6b,6a
1a,1b
7b,7a
3c
4c
5b,5a 2a,2b
2c
4a,4b
5c
9
f1 (ppm)
f2 (ppm)
6b

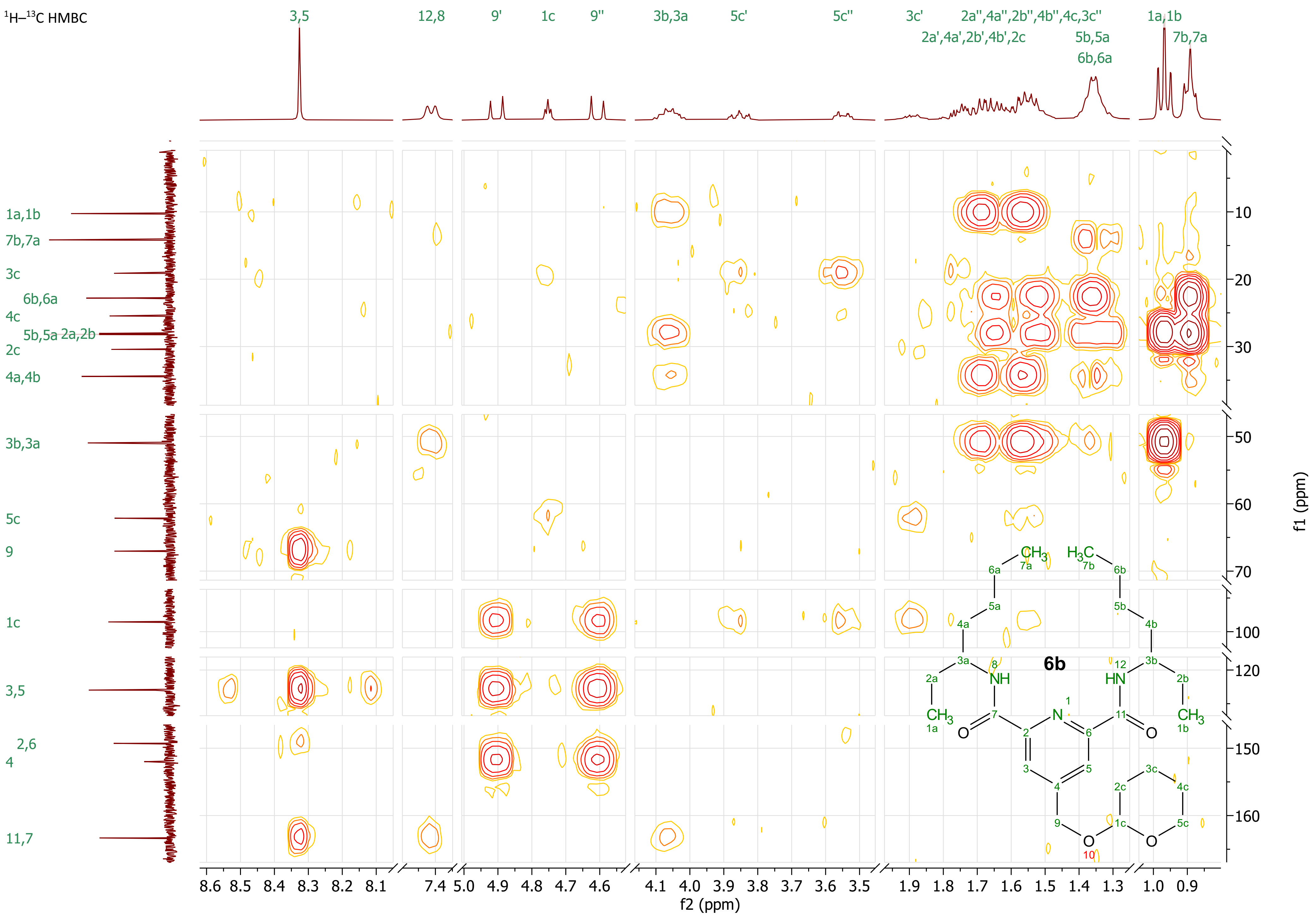

1H–13C HMBC
3,5
12,8
9'
1c
9''
3b,3a
5c'
5c''
3c'
2a'',4a'',2b'',4b'',4c,3c''
2a',4a',2b',4b',2c
5b,5a
6b,6a
1a,1b
7b,7a
1a,1b
7b,7a
3c
6b,6a
4c
5b,5a
2a,2b
2c
4a,4b
3b,3a
5c
9
1c
3,5
2,6
4
11,7
6b
f2 (ppm)
f1 (ppm)

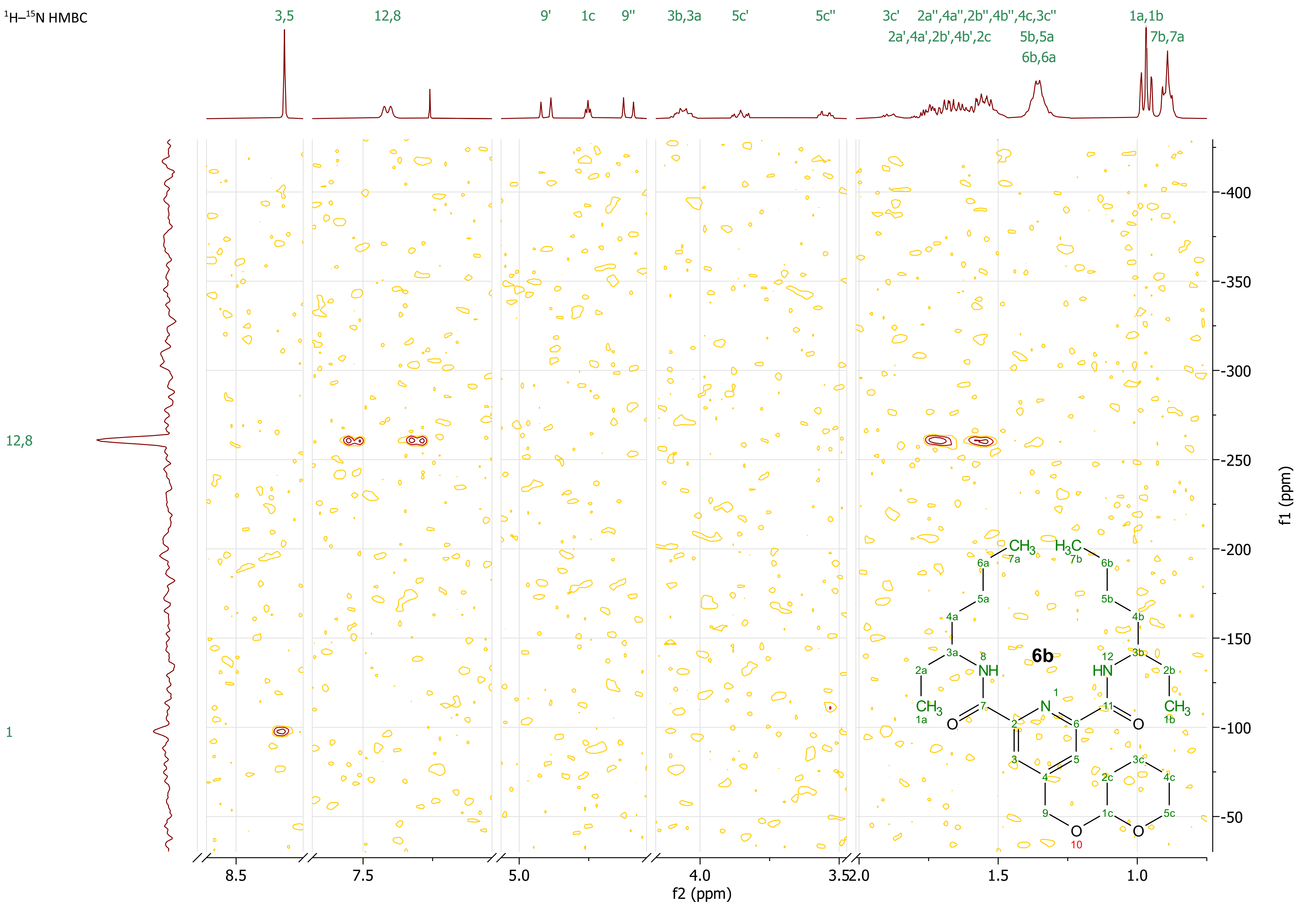
^{1}H–^{15}N HMBC
3,5
12,8
9'
1c
9''
3b,3a
5c'
5c''
3c'
2a'',4a'',2b'',4b'',4c,3c''
2a',4a',2b',4b',2c
5b,5a
6b,6a
1a,1b
7b,7a
12,8
1
6b
f1 (ppm)
f2 (ppm)

[Back ↑]

^{15}N NMR (41 MHz, $CDCl_3$) δ -97.7, -260.9. – Projection f1

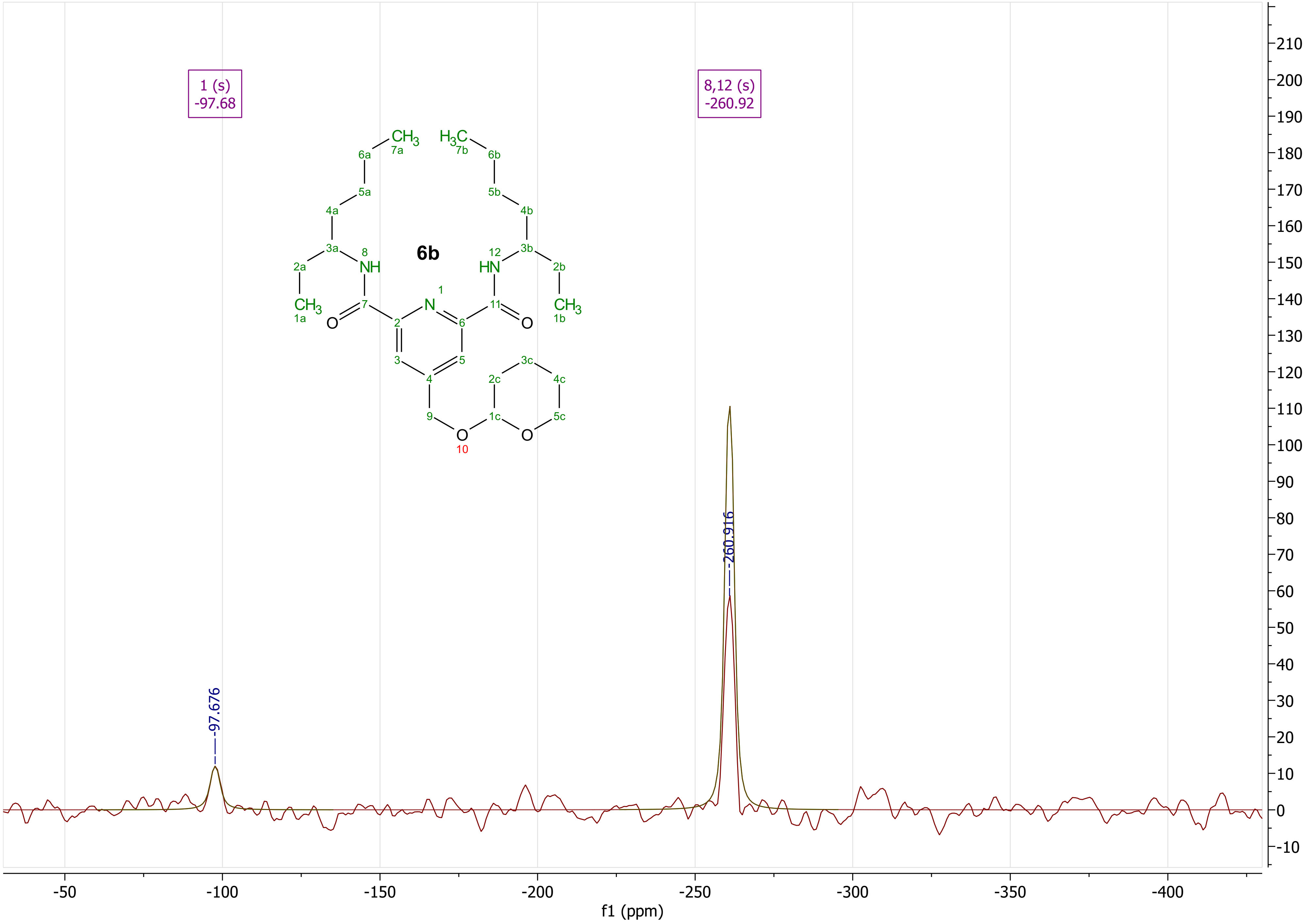

^1H NMR (400 MHz, $CDCl_3$) δ 8.42 (t, *J* = 6.4 Hz, 2H), 8.32 (app t, *J* = 0.9 Hz, 2H), 7.47 – 7.39 (m, 6H), 7.33 (t, *J* = 7.8 Hz, 2H), 4.75 (d, *J* = 5.5 Hz, 2H), 4.58 (d, *J* = 6.3 Hz, 4H), 3.57 (t, *J* = 5.9 Hz, 1H).

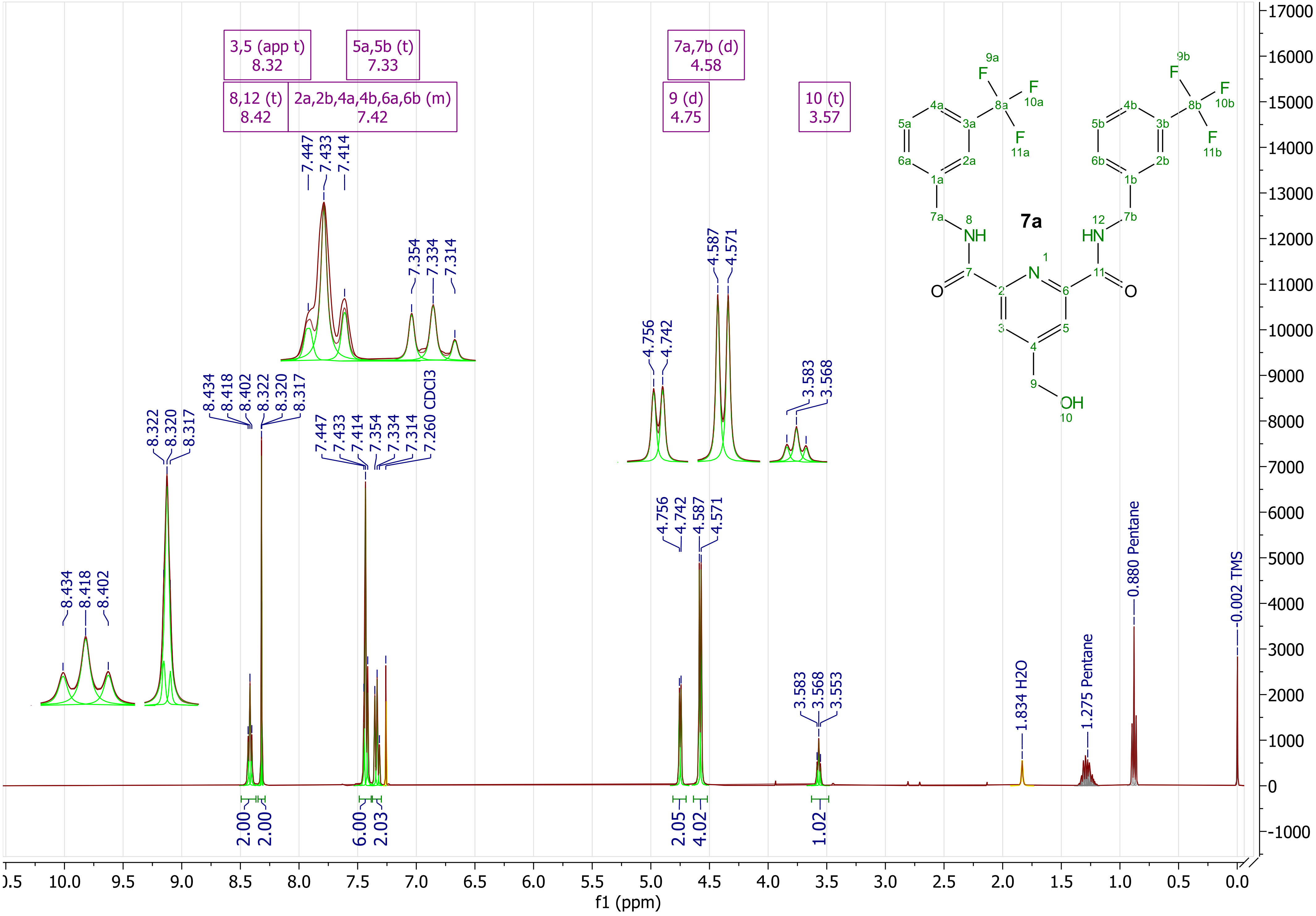

[Back ↑]

^{1}H NMR (400 MHz, DMSO-d_6) δ 9.93 (t, J = 6.4 Hz, 2H), 8.22 (app t, J = 0.9 Hz, 2H), 7.71 – 7.66 (m, 2H), 7.66 – 7.63 (m, 2H), 7.63 – 7.61 (m, 2H), 7.60 – 7.55 (m, 2H), 5.66 (t, J = 5.8 Hz, 1H), 4.71 (t, J = 5.5 Hz, 6H).

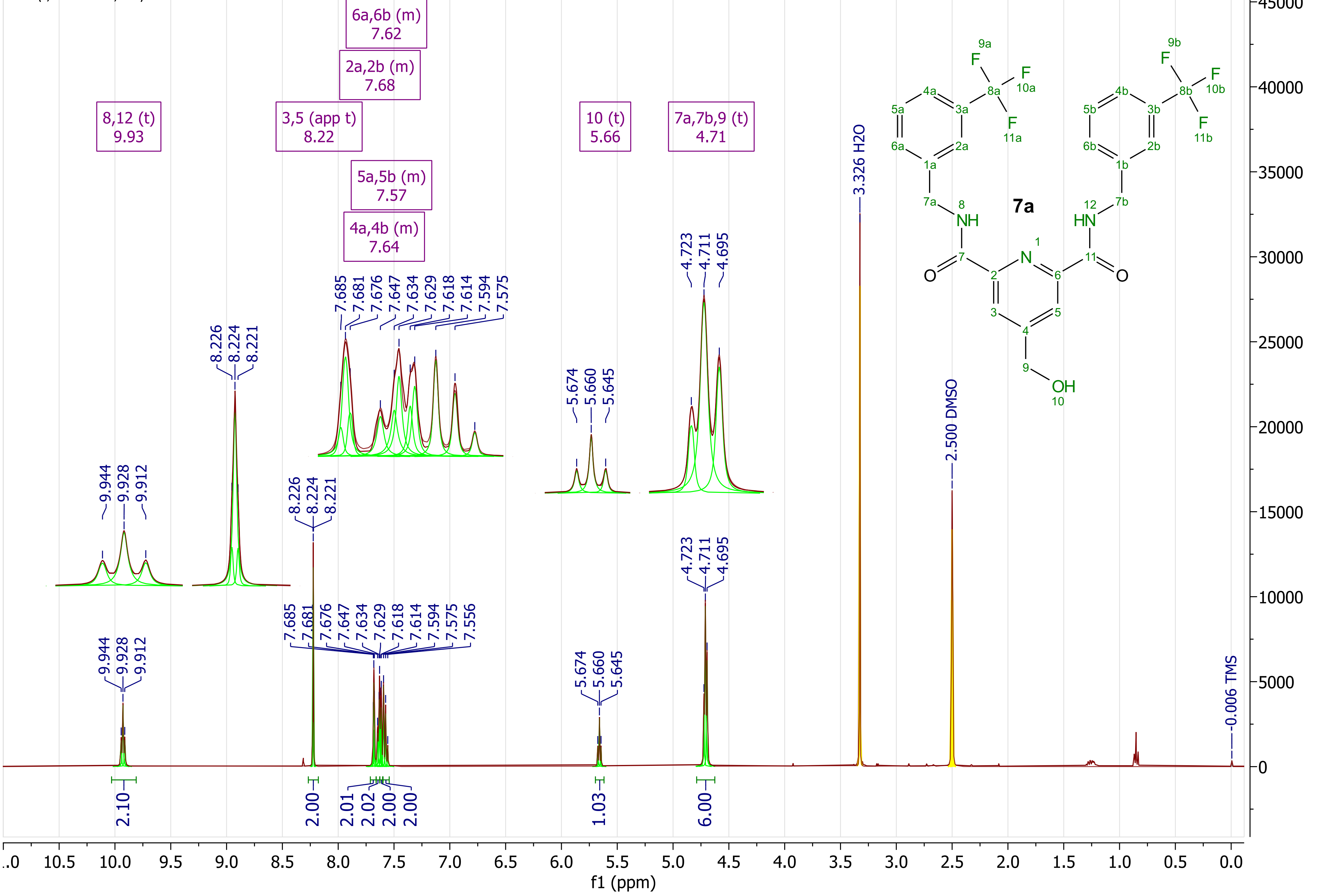

[Back ↑]

^{1}H NMR (400 MHz) – Spectra comparison – [8.1 – 10.2 ppm]

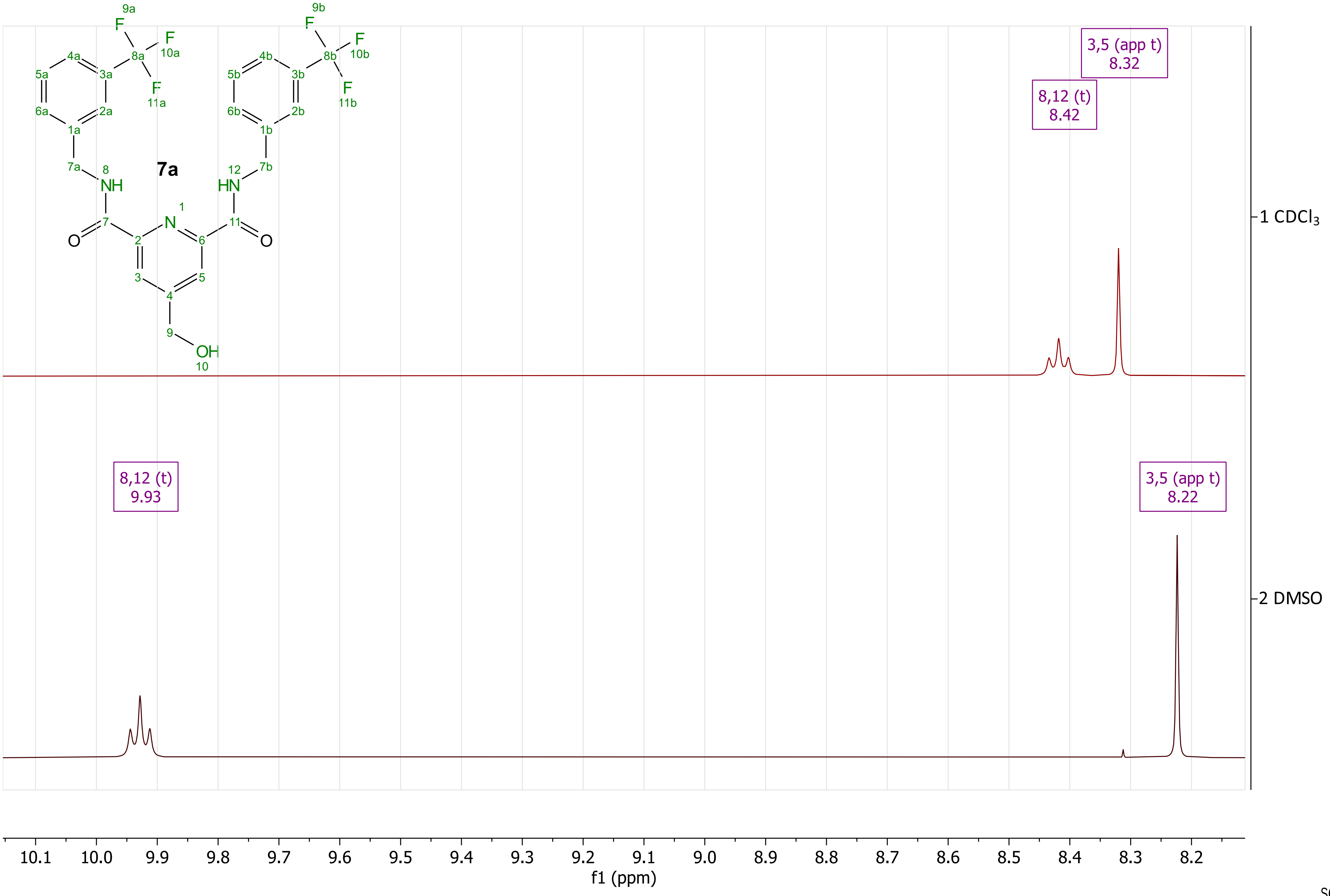

[Back ↑]

^{13}C NMR (101 MHz, $CDCl_3$) δ 164.2 (2C), 154.9, 148.6 (2C), 139.1 (2C), 131.1 (2C), 131.0 (q, J = 32.1 Hz, 2C), 129.3 (2C), 124.5 (q, J = 3.8 Hz, 2C), 124.3 (q, J = 3.8 Hz, 2C), 124.0 (q, J = 272.3 Hz, 2C), 122.9 (2C), 63.0, 43.1 (2C).

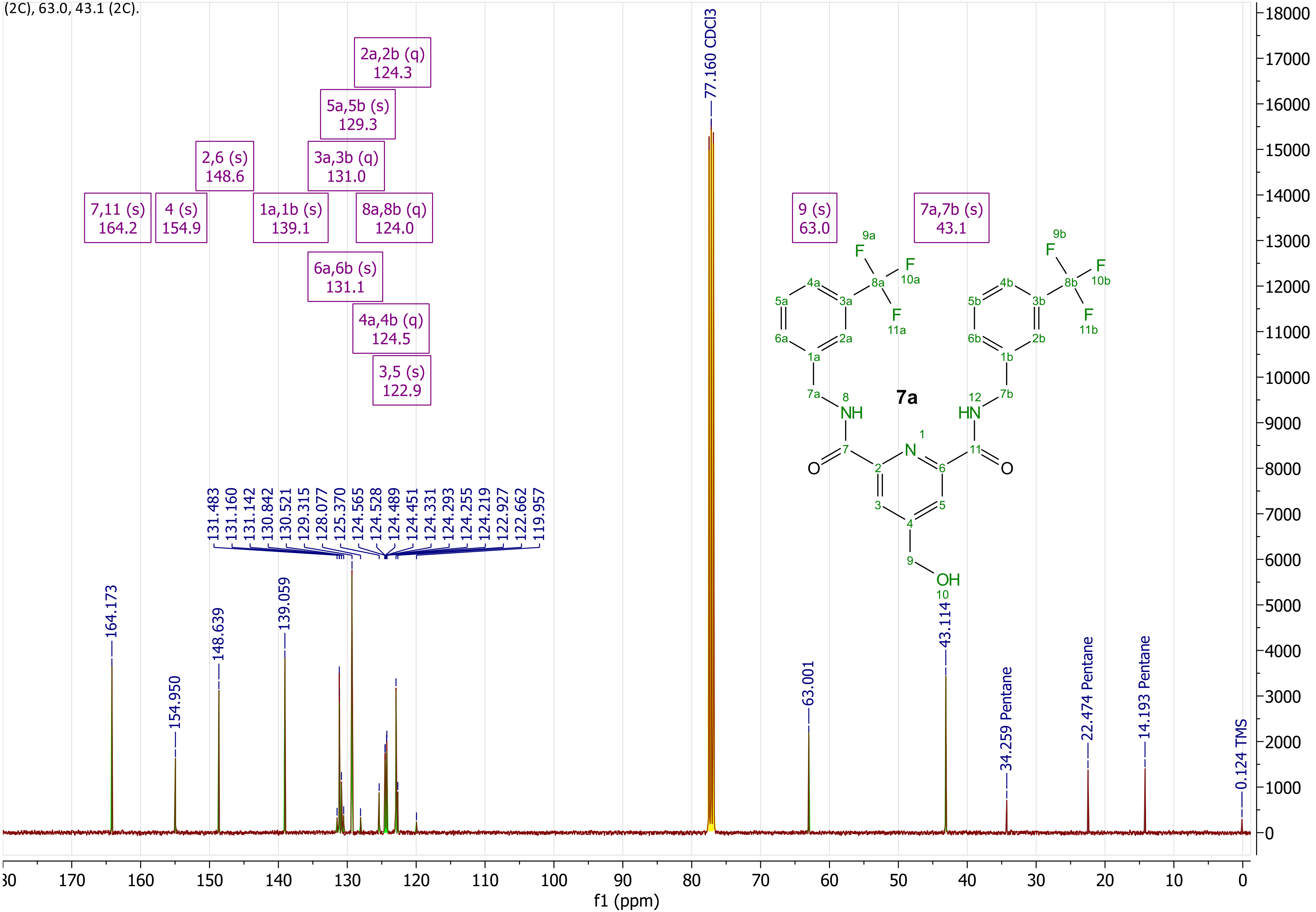

[Back ↑]

^{13}C NMR (101 MHz, $CDCl_3$) – [119.0 – 132.0 ppm]

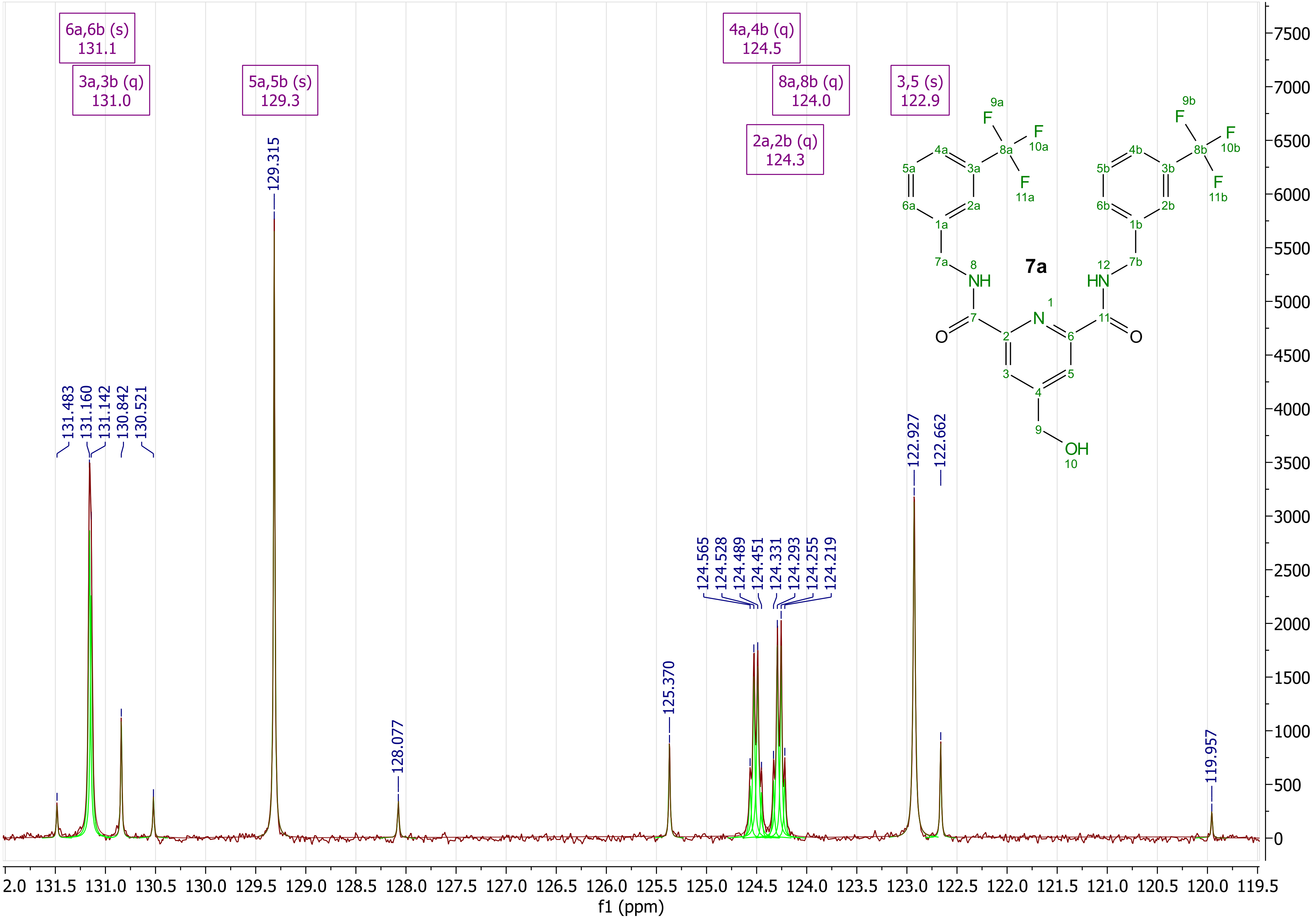

[Back ↑]

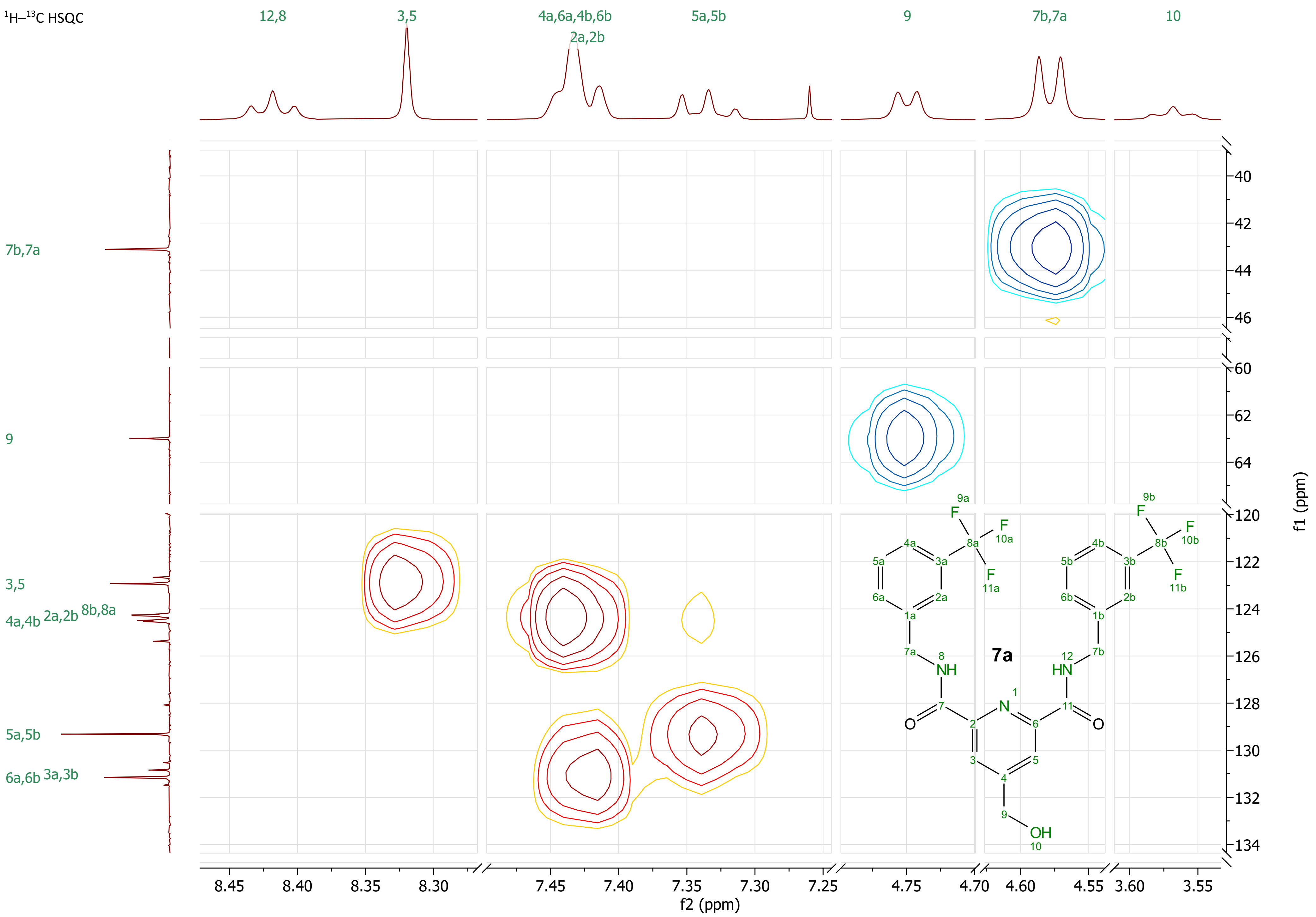

^{1}H–^{13}C HSQC
12,8
3,5
4a,6a,4b,6b
2a,2b
5a,5b
9
7b,7a
10
7b,7a
9
3,5
4a,4b 2a,2b 8b,8a
5a,5b
6a,6b 3a,3b
7a
f2 (ppm)
f1 (ppm)

[Back ↑]

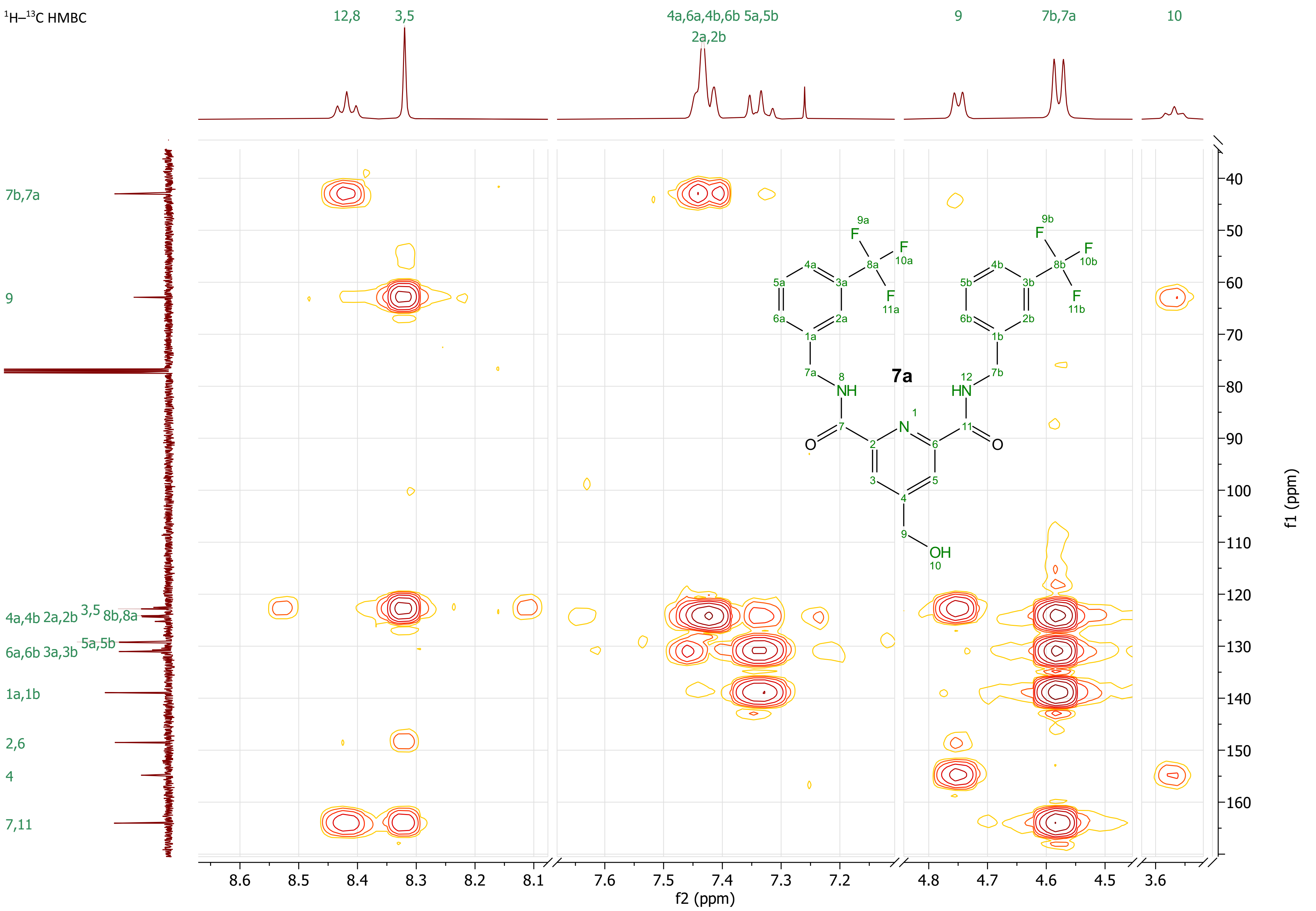
$^{1}H–^{13}C$ HMBC
12,8
3,5
4a,6a,4b,6b 5a,5b
2a,2b
9
7b,7a
10
7b,7a
9
4a,4b 2a,2b 3,5 8b,8a
6a,6b 3a,3b 5a,5b
1a,1b
2,6
4
7,11
7a
f1 (ppm)
f2 (ppm)

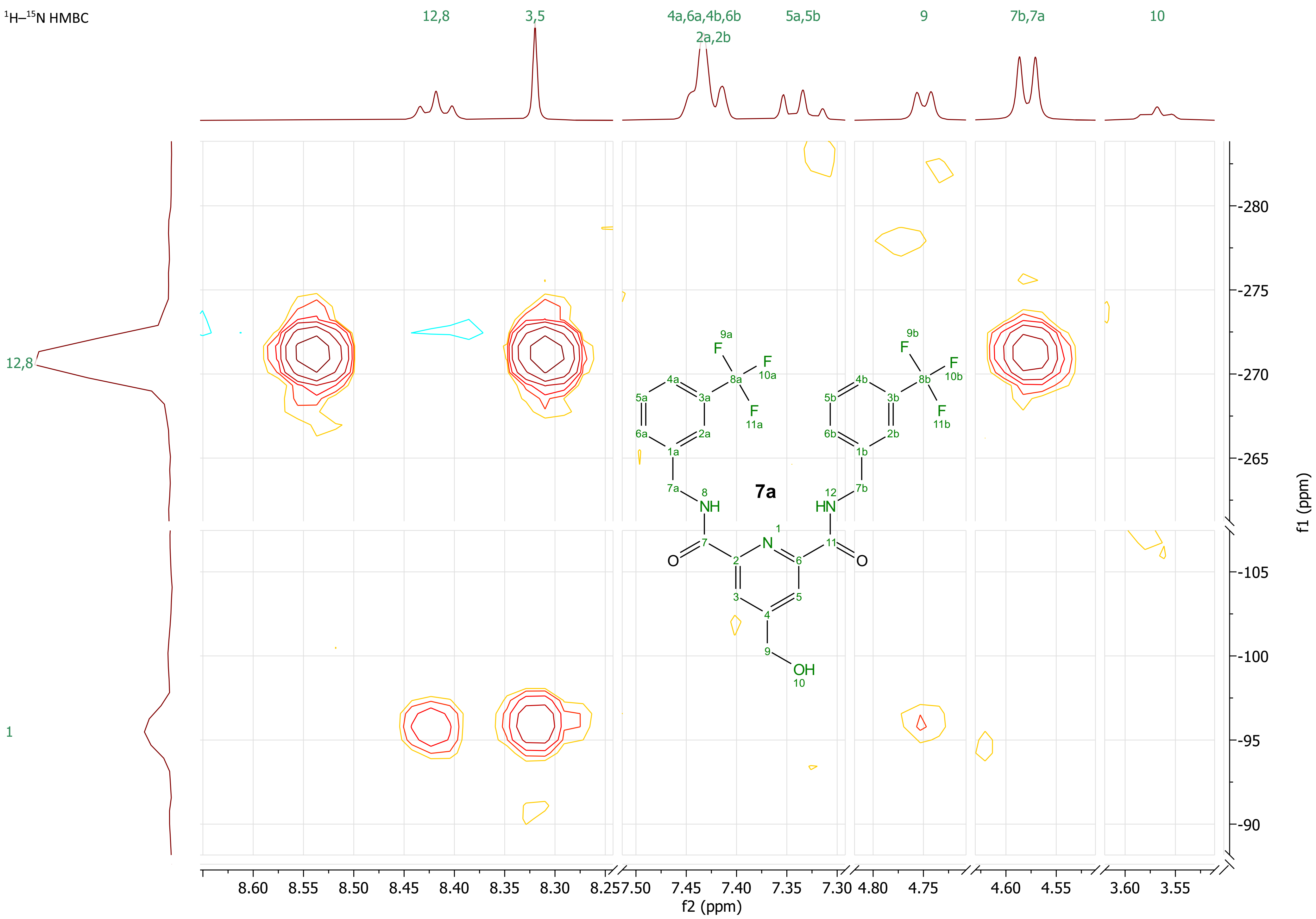

^{1}H–^{15}N HMBC
12,8
3,5
4a,6a,4b,6b
2a,2b
5a,5b
9
7b,7a
10
12,8
1
7a
f2 (ppm)
f1 (ppm)

^{15}N NMR (41 MHz, $CDCl_3$) δ -95.53, -270.68. – Projection f1

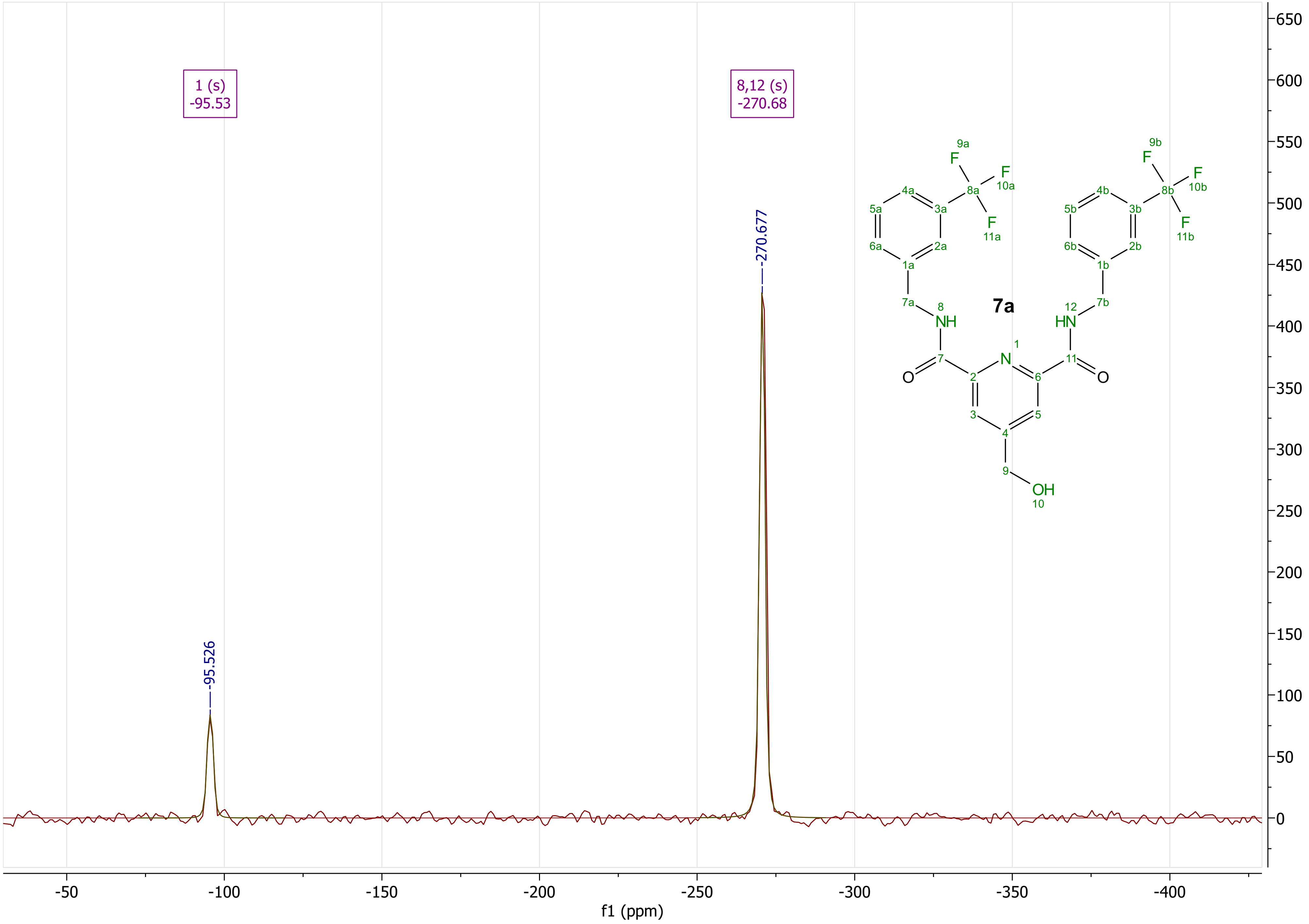

[Back ↑]

^{19}F NMR (376 MHz, $CDCl_3$) δ -62.64.

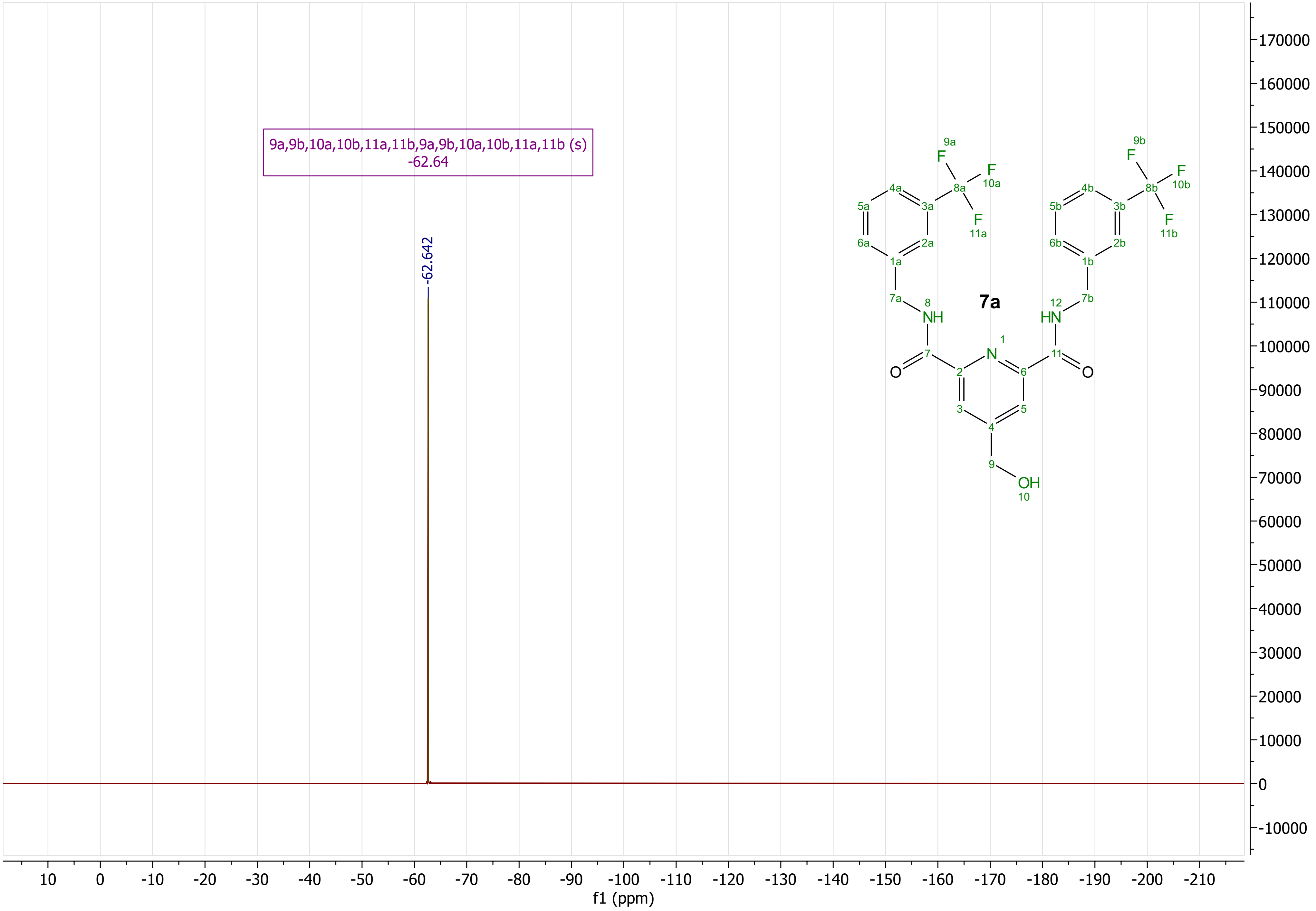

[Back ↑]

^{1}H NMR (400 MHz, $CDCl_3$) δ 8.38 (app t, *J* = 0.8 Hz, 2H), 7.45 (d, *J* = 9.3 Hz, 2H), 4.85 (s, 2H), 4.11 – 3.98 (m, 2H), 3.86 (s, 1H), 1.76 – 1.58 (m, 4H), 1.61 – 1.43 (m, 4H), 1.43 – 1.26 (m, 8H), 0.95 (t, *J* = 7.4 Hz, 6H), 0.88 (app t, *J* = 6.5 Hz, 6H).

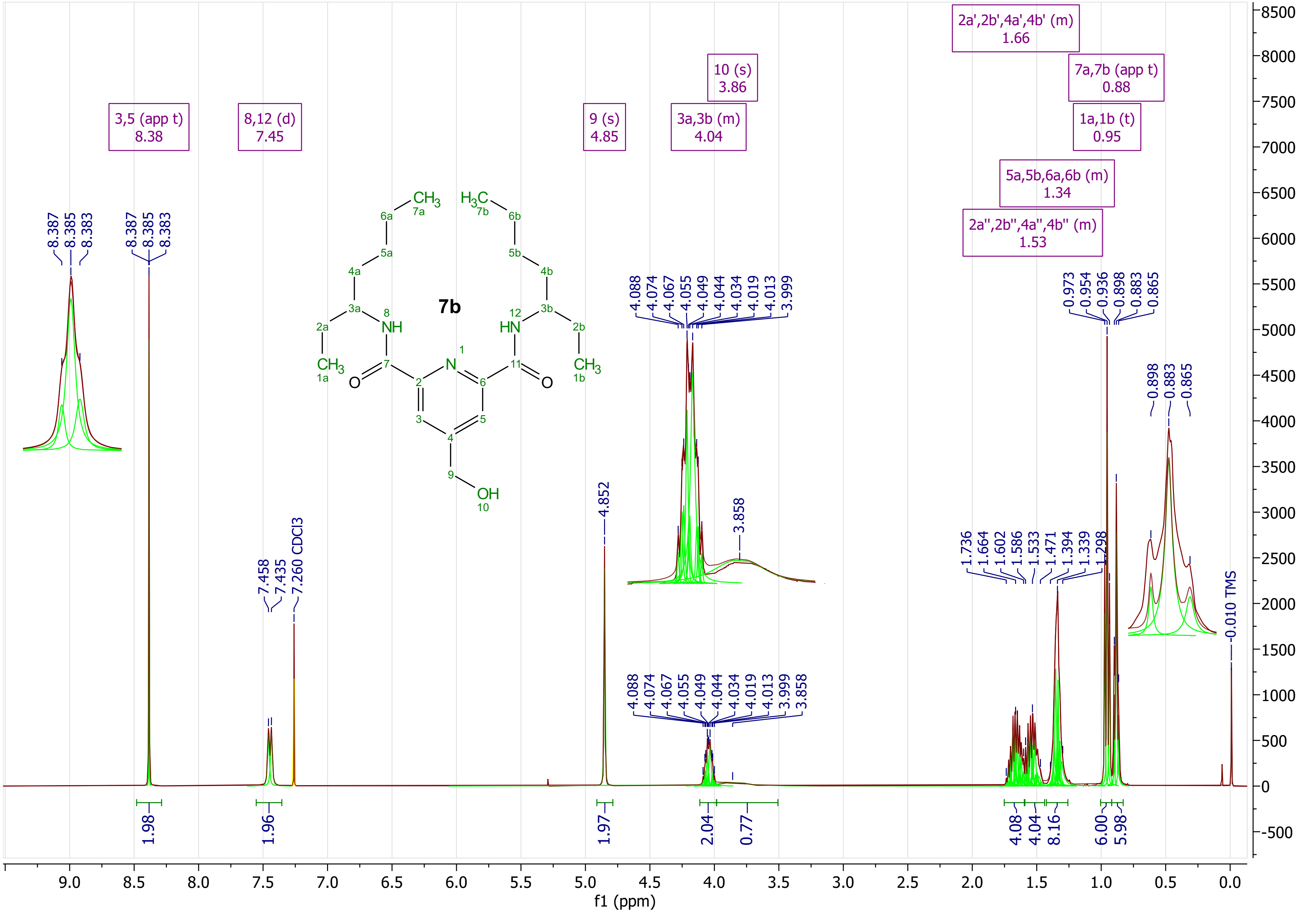

^{13}C NMR (101 MHz, $CDCl_3$) δ 163.5 (2C), 155.0, 149.2 (2C), 122.6 (2C), 63.3, 51.0 (2C), 34.4 (2C), 28.2 (2C), 28.0 (d, J = 1.5 Hz, 2C), 22.8 (d, J = 1.6 Hz, 2C), 14.1 (2C), 10.2 (2C).

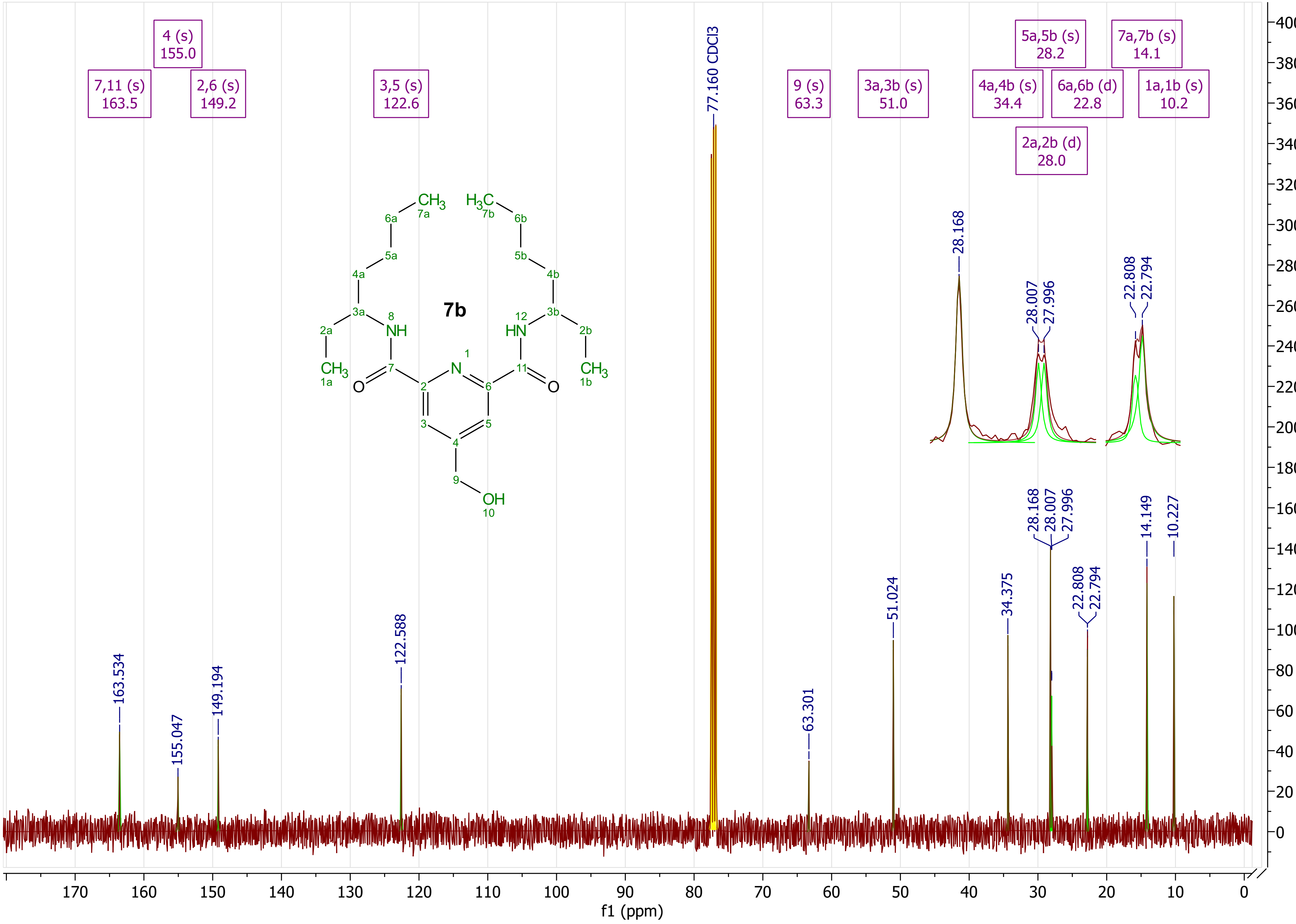

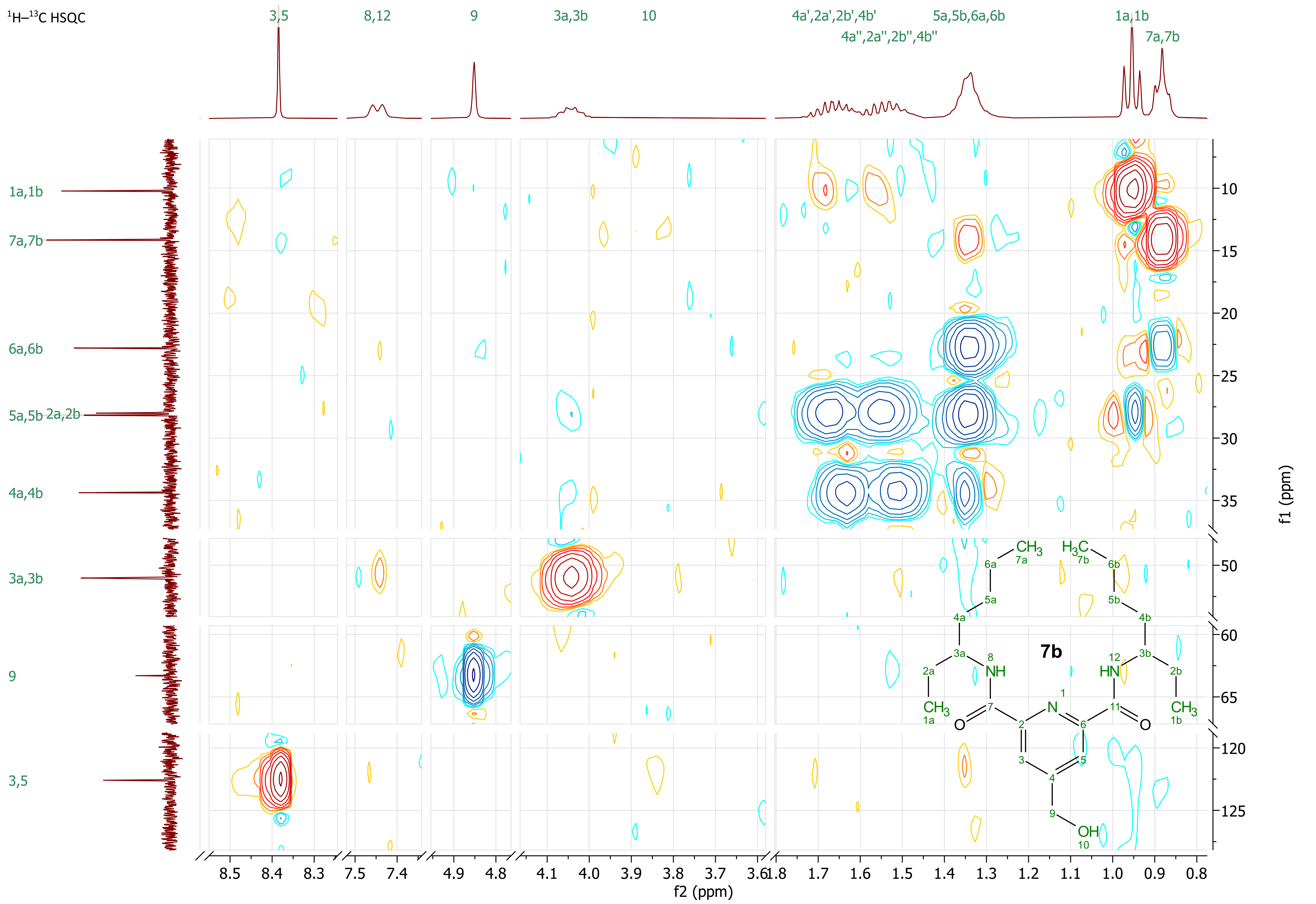

1H–13C HSQC
3,5
8,12
9
3a,3b
10
4a',2a',2b',4b'
4a'',2a'',2b'',4b''
5a,5b,6a,6b
1a,1b
7a,7b
1a,1b
7a,7b
6a,6b
5a,5b 2a,2b
4a,4b
3a,3b
9
3,5
7b
f1 (ppm)
f2 (ppm)

[Back ↑]

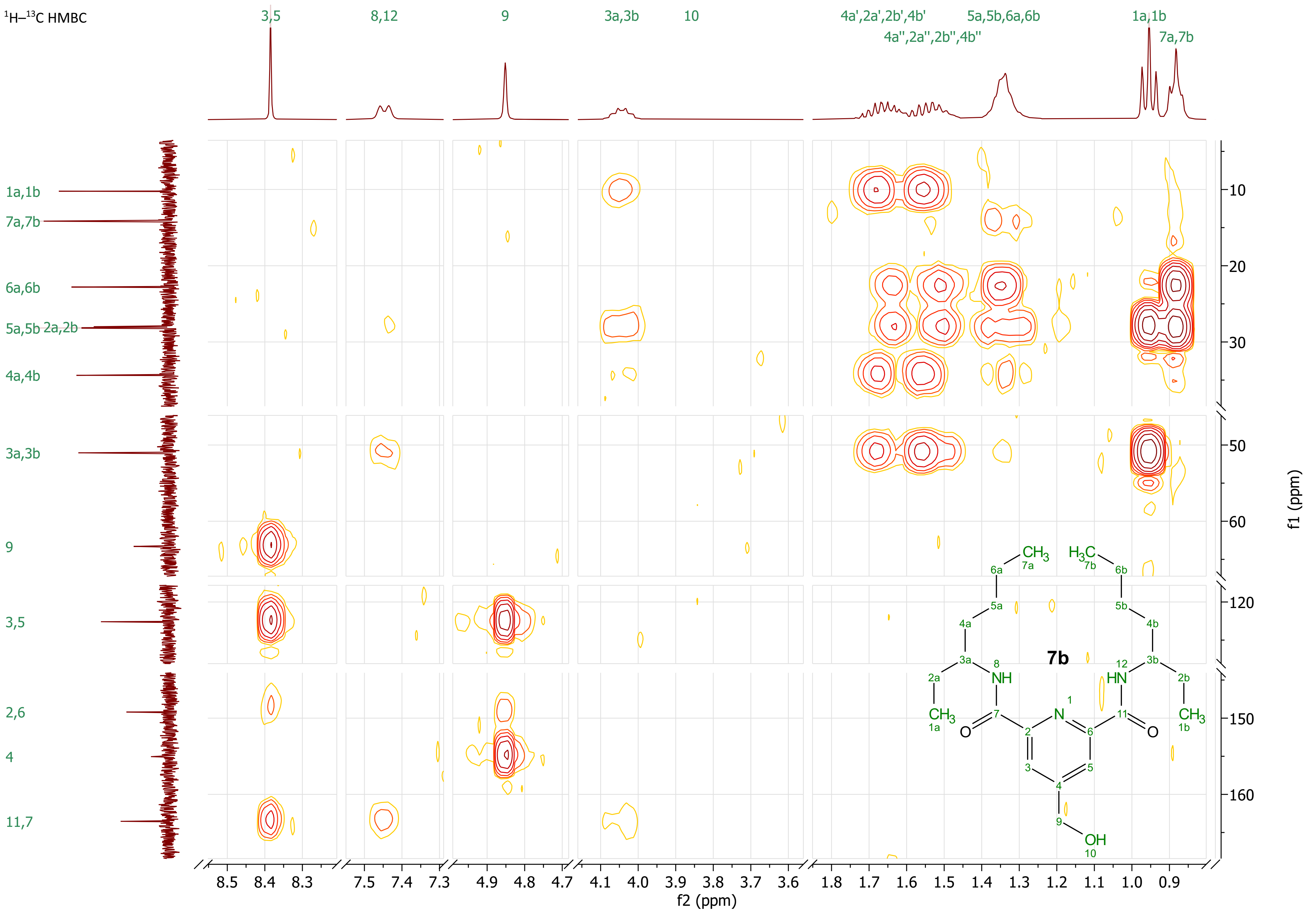

$^{1}H–^{13}C$ HMBC
3,5
8,12
9
3a,3b
10
4a',2a',2b',4b'
4a'',2a'',2b'',4b''
5a,5b,6a,6b
1a,1b
7a,7b
1a,1b
7a,7b
6a,6b
5a,5b 2a,2b
4a,4b
3a,3b
9
3,5
2,6
4
11,7
7b
f2 (ppm)
f1 (ppm)

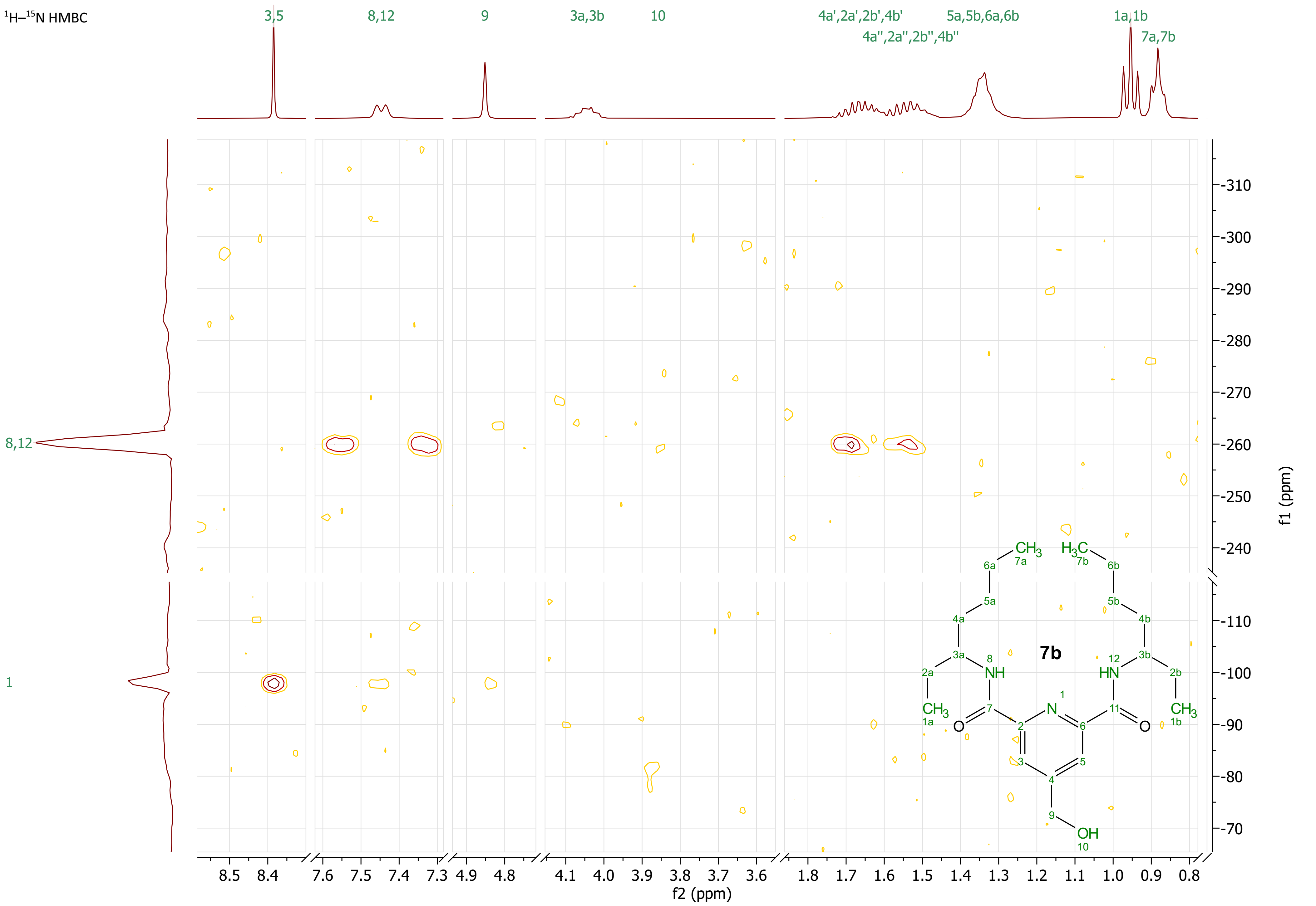
^{1}H–^{15}N HMBC
3,5
8,12
9
3a,3b
10
4a',2a',2b',4b'
4a'',2a'',2b'',4b''
5a,5b,6a,6b
1a,1b
7a,7b
8,12
1
7b
f1 (ppm)
f2 (ppm)

^{15}N NMR (41 MHz, $CDCl_3$) δ -98.26, -260.27. – Projection f1

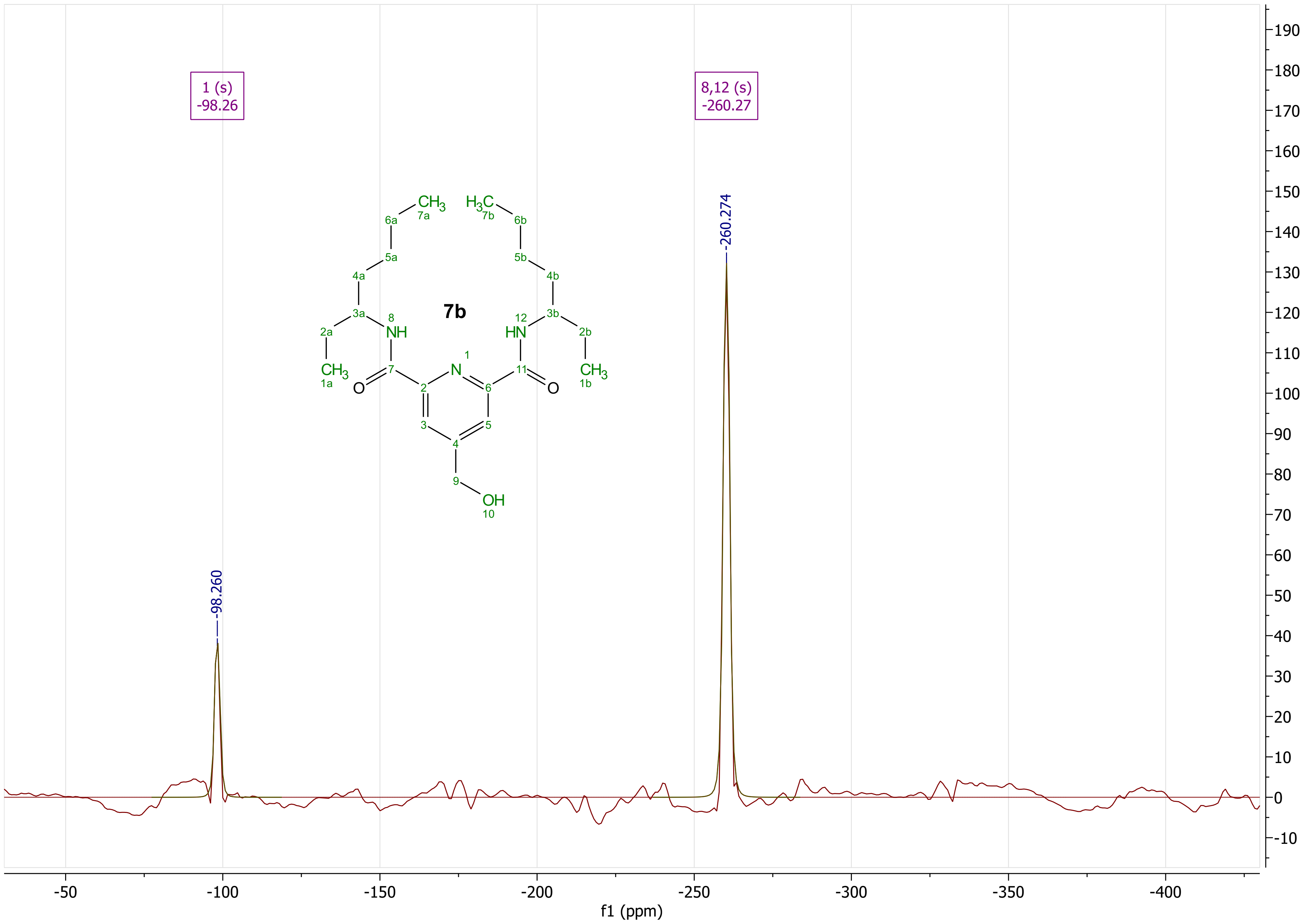

[Back ↑]

^{1}H NMR (400 MHz, $CDCl_3$) δ 8.33 (app t, *J* = 0.9 Hz, 2H), 7.82 (t, *J* = 6.1 Hz, 2H), 4.82 (app d, *J* = 4.4 Hz, 2H), 3.91 (br s, 1H), 3.55 – 3.32 (m, 4H), 1.72 – 1.55 (m, 4H), 1.43 – 1.20 (m, 20H), 0.86 (app t, *J* = 7.1, 6.7 Hz, 6H).

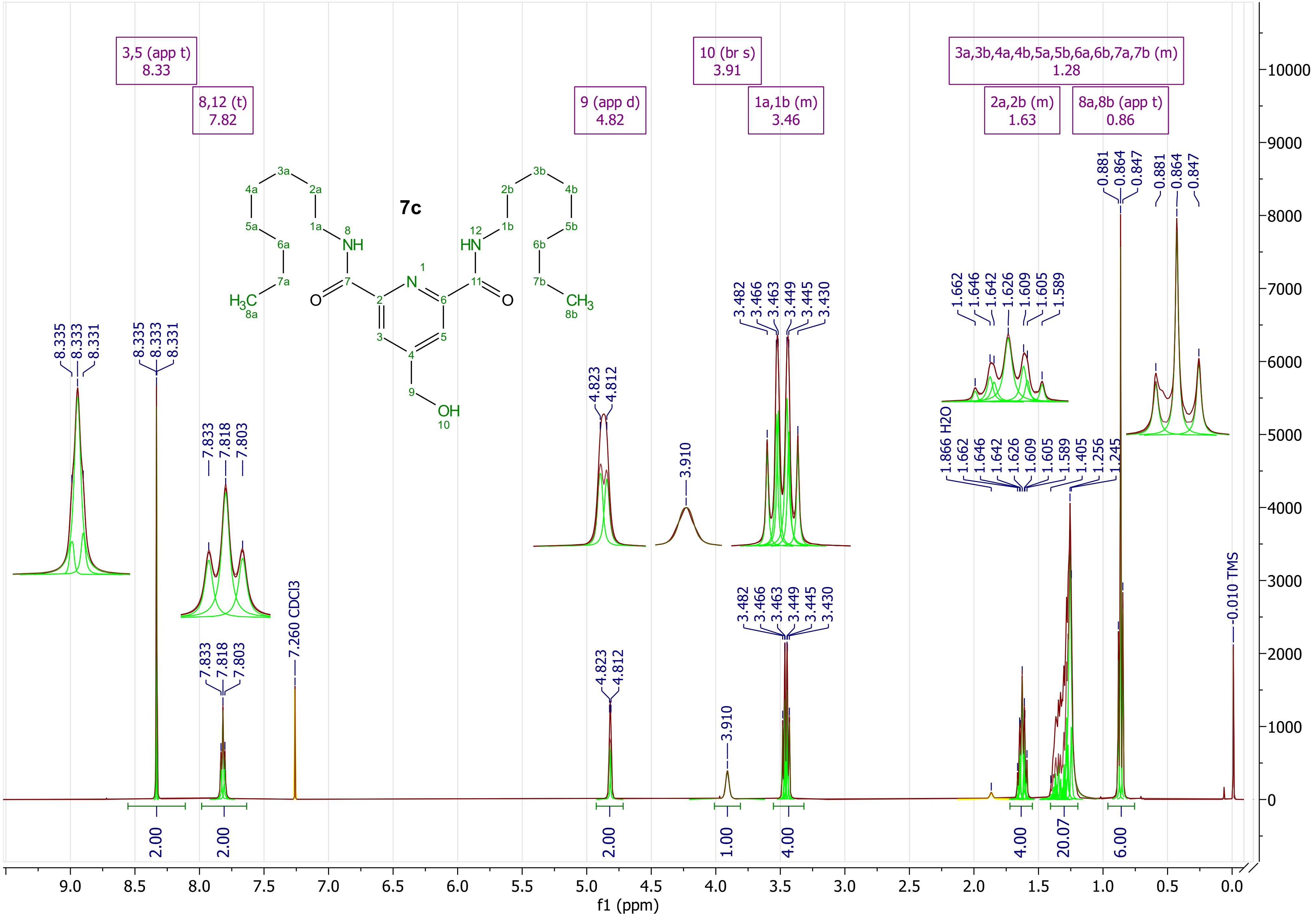

^{13}C NMR (101 MHz, $CDCl_3$) δ 163.9 (2C), 154.9, 149.1 (2C), 122.5 (2C), 63.3, 39.9 (2C), 31.9 (2C), 29.8 (2C), 29.43 (2C), 29.37 (2C), 27.2 (2C), 22.8 (2C), 14.2 (2C).

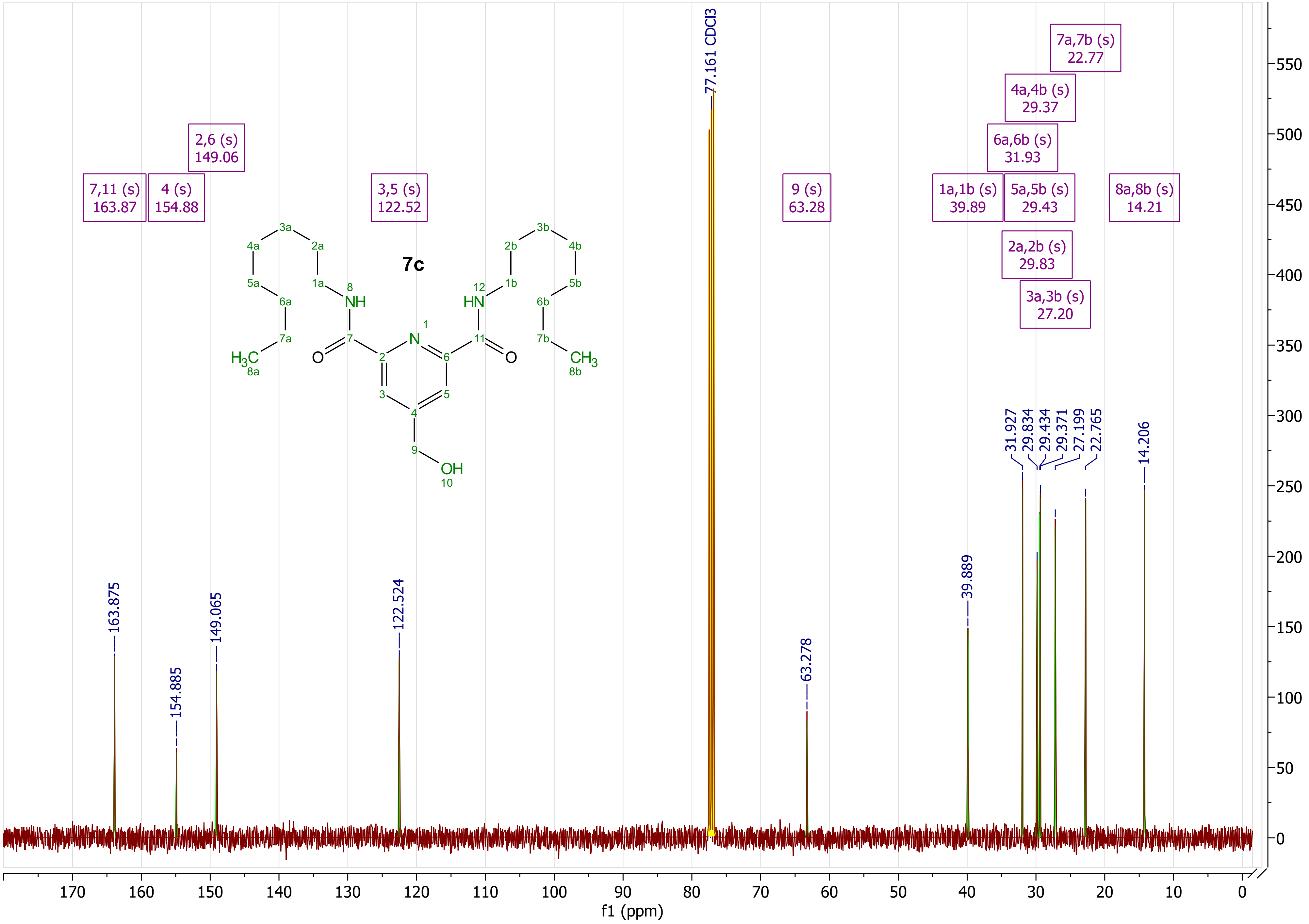

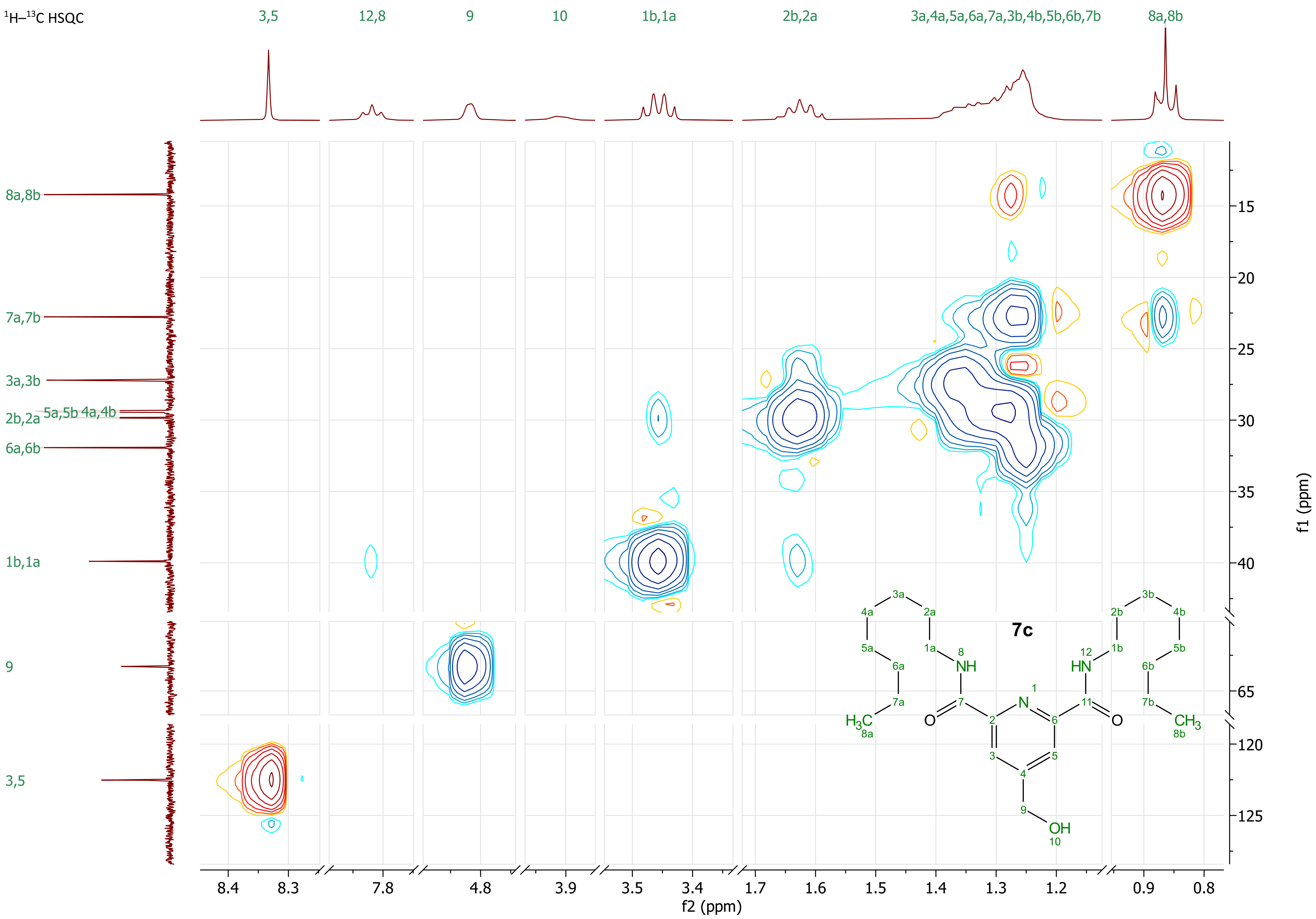

1H–13C HSQC
3,5
12,8
9
10
1b,1a
2b,2a
3a,4a,5a,6a,7a,3b,4b,5b,6b,7b
8a,8b
7a,7b
3a,3b
2b,2a
5a,5b 4a,4b
6a,6b
7c
NH
HN
N
OH
O
H3C
CH3
f1 (ppm)
f2 (ppm)

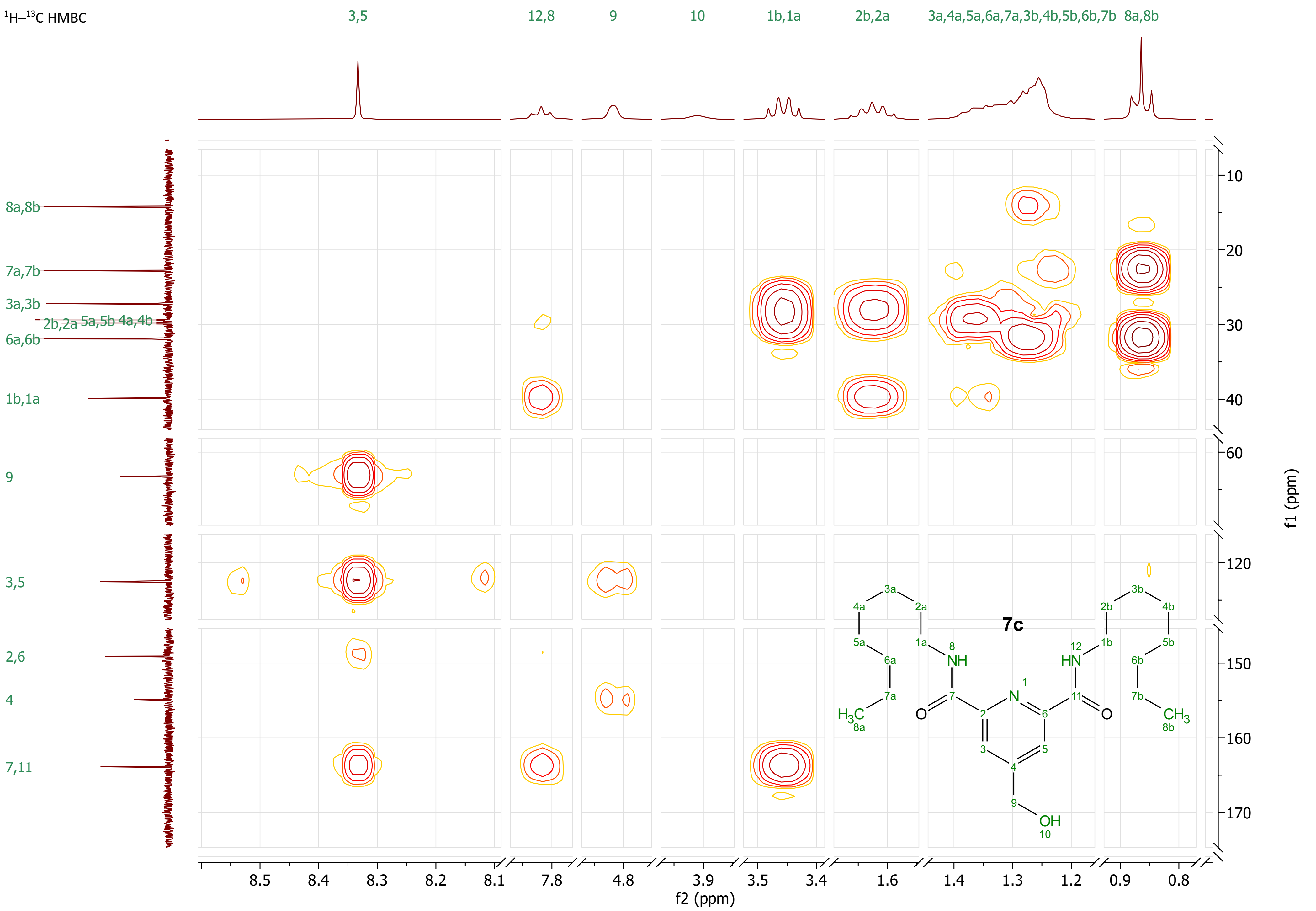
^1H–^{13}C HMBC
3,5
12,8
9
10
1b,1a
2b,2a
3a,4a,5a,6a,7a,3b,4b,5b,6b,7b
8a,8b
7a,7b
3a,3b
2b,2a
5a,5b
4a,4b
6a,6b
2,6
4
7,11
7c
f2 (ppm)
f1 (ppm)

[Back ↑]

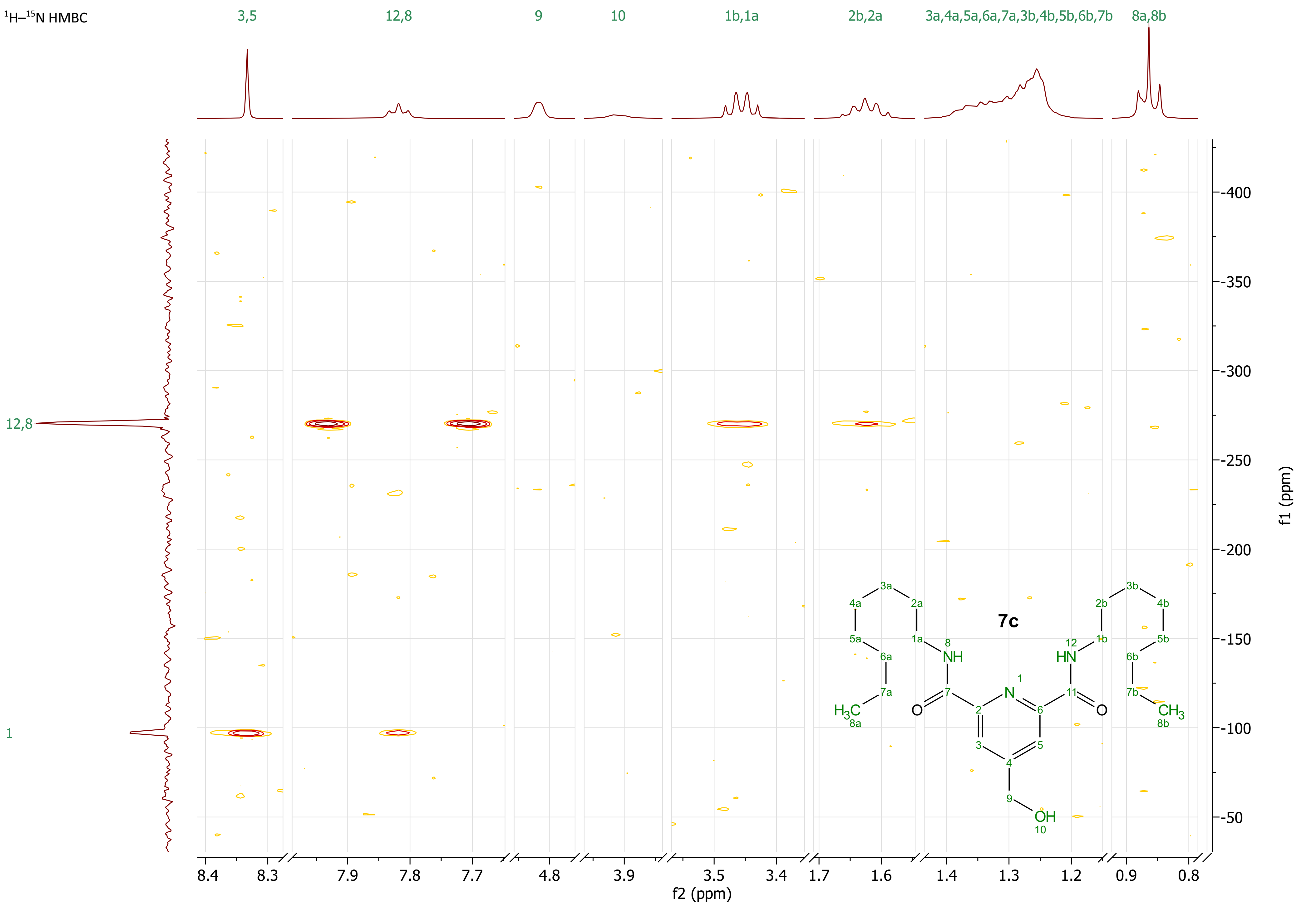

^{1}H–^{15}N HMBC
3,5
12,8
9
10
1b,1a
2b,2a
3a,4a,5a,6a,7a,3b,4b,5b,6b,7b
8a,8b
12,8
1
7c
f2 (ppm)
f1 (ppm)

^{15}N NMR (41 MHz, $CDCl_3$) δ -97.27, -270.50. – Projection f1

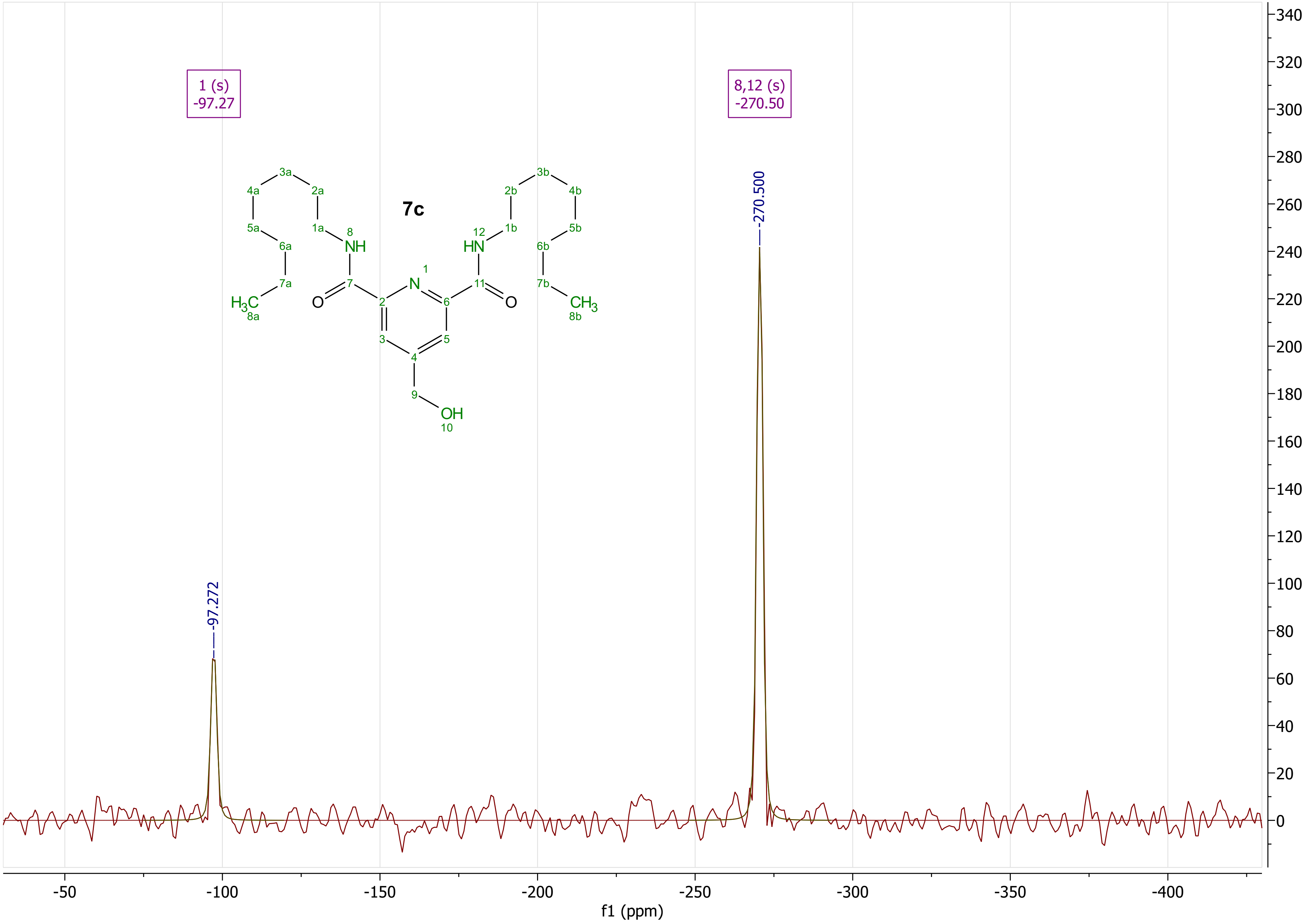

[Back ↑]

^{1}H NMR (400 MHz, $CDCl_3$) δ 7.82 – 7.67 (mm, 2H), 7.62 – 7.28 (mm, 8H), 4.79 – 4.68 (mm, 2H), 4.84 – 4.47 (ms, 4H), 3.43 – 3.25 (mm, 1H), 3.08 – 2.79 (ms, 6H). – Mixture of confomers.

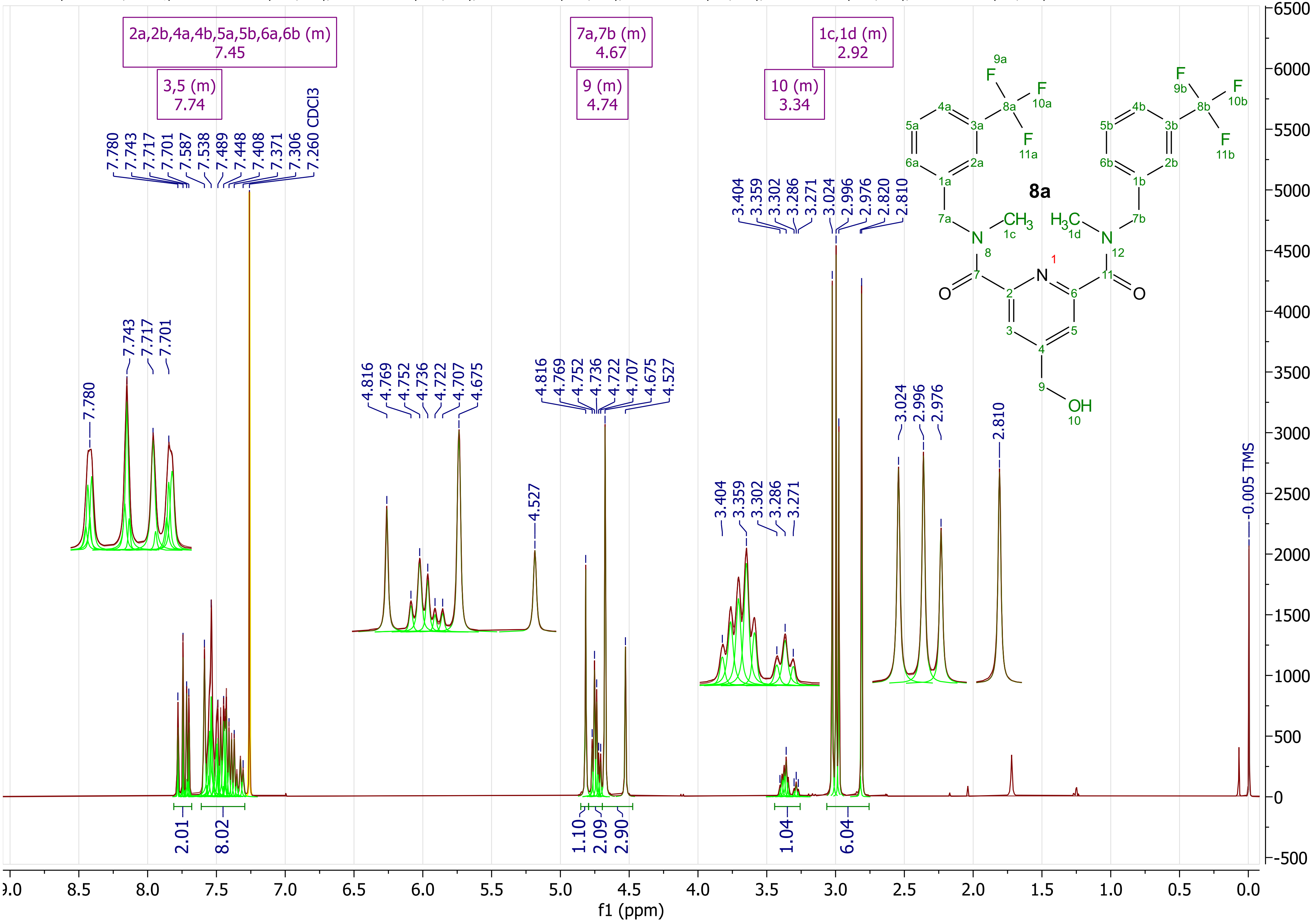

[Back ↑]

^{13}C NMR (101 MHz, $CDCl_3$) δ 168.9 – 168.6 (ms, 2C), 154.4 – 153.9 (ms), 152.8 – 152.5 (ms, 2C), 138.0 – 137.6 (ms, 2C), 131.8 – 130.7 (mm, 2C), 129.4 (2C), 129.4 – 129.1 (mm, 2C), 125.1 – 124.5 (mm, 2C), 124.7 – 124.1 (mm, 2C), 128.4 – 119.9 (mm, 2C), 122.5 – 121.9 (ms, 2C), 63.0 – 62.8 (ms), 54.9 – 50.8 (ms, 2C), 37.3 – 33.4 (ms, 2C). Mixture of conformers

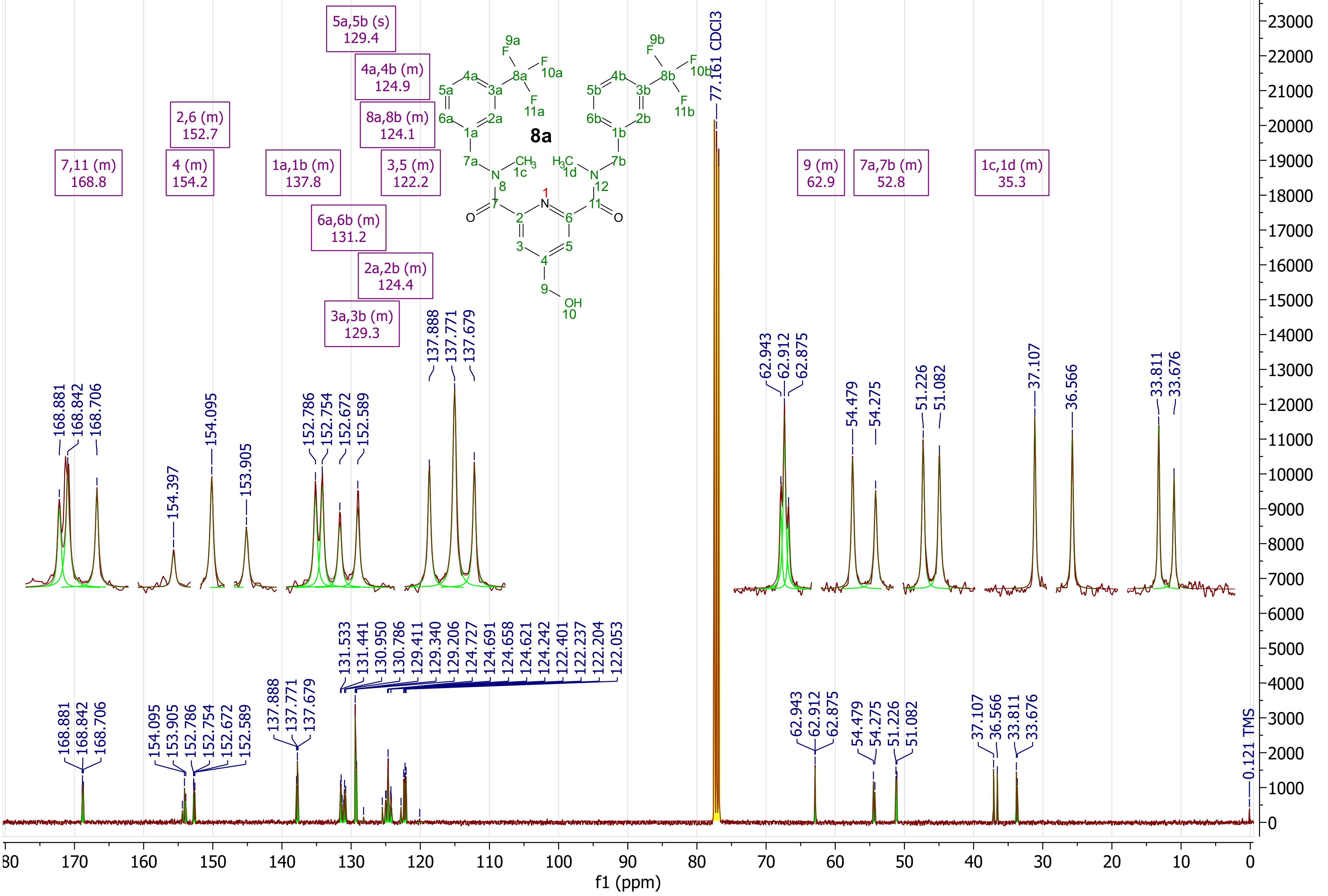

^{13}C NMR (101 MHz, $CDCl_3$) – [132.5 – 119.5 ppm]

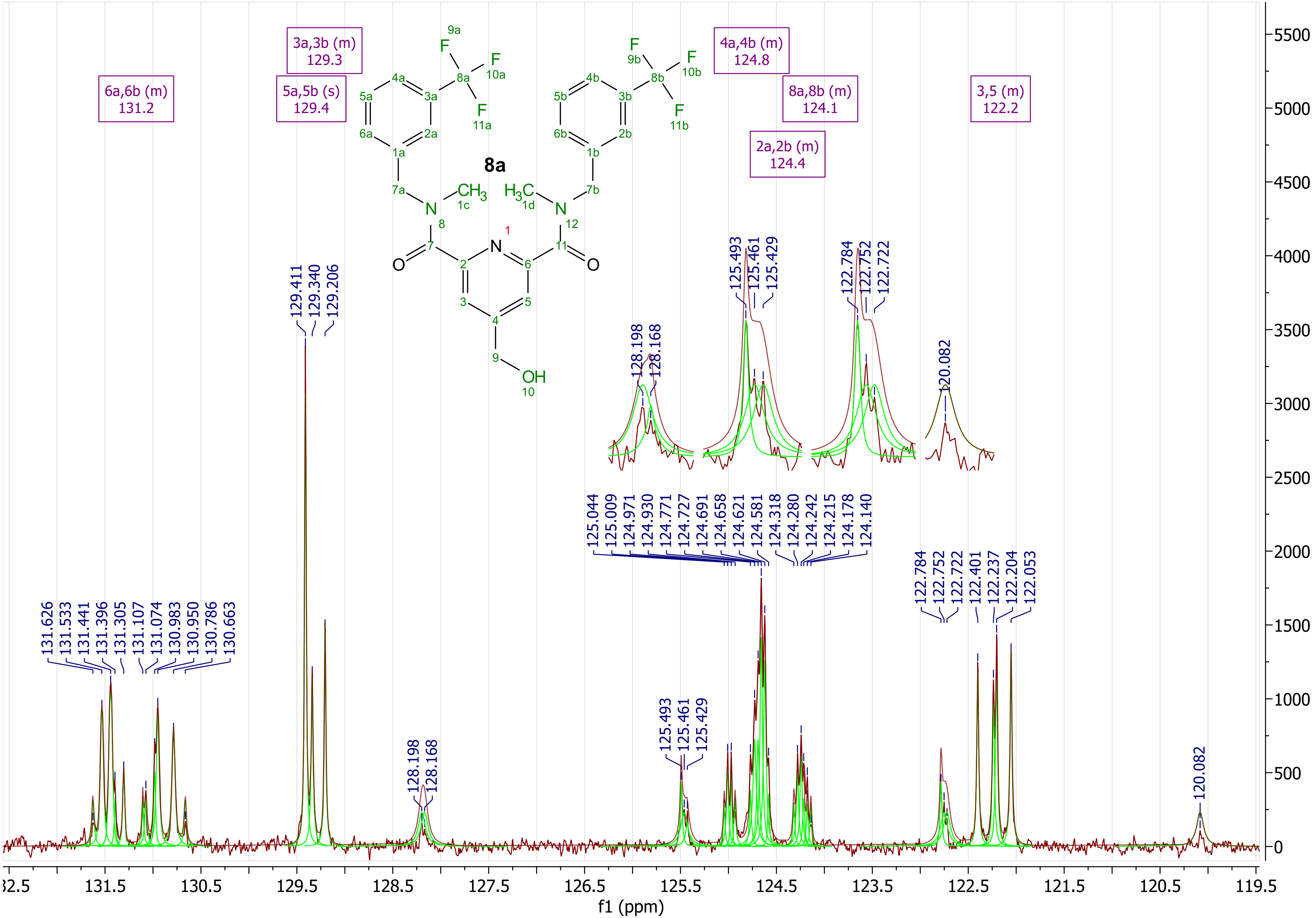

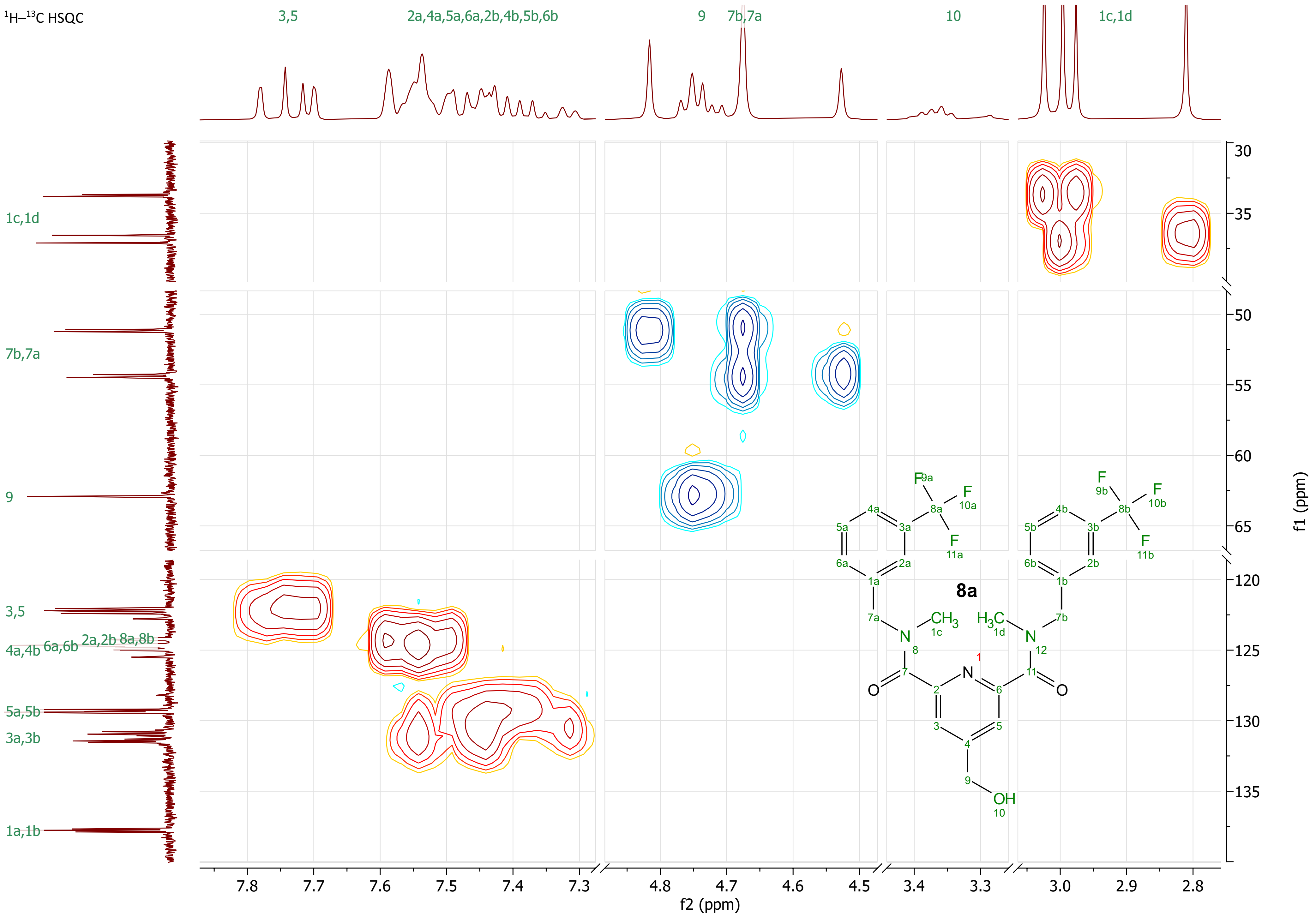

^{1}H–^{13}C HSQC
3,5
2a,4a,5a,6a,2b,4b,5b,6b
9
7b,7a
10
1c,1d
3,5
4a,4b
6a,6b
2a,2b
8a,8b
5a,5b
3a,3b
1a,1b
f2 (ppm)
f1 (ppm)
8a

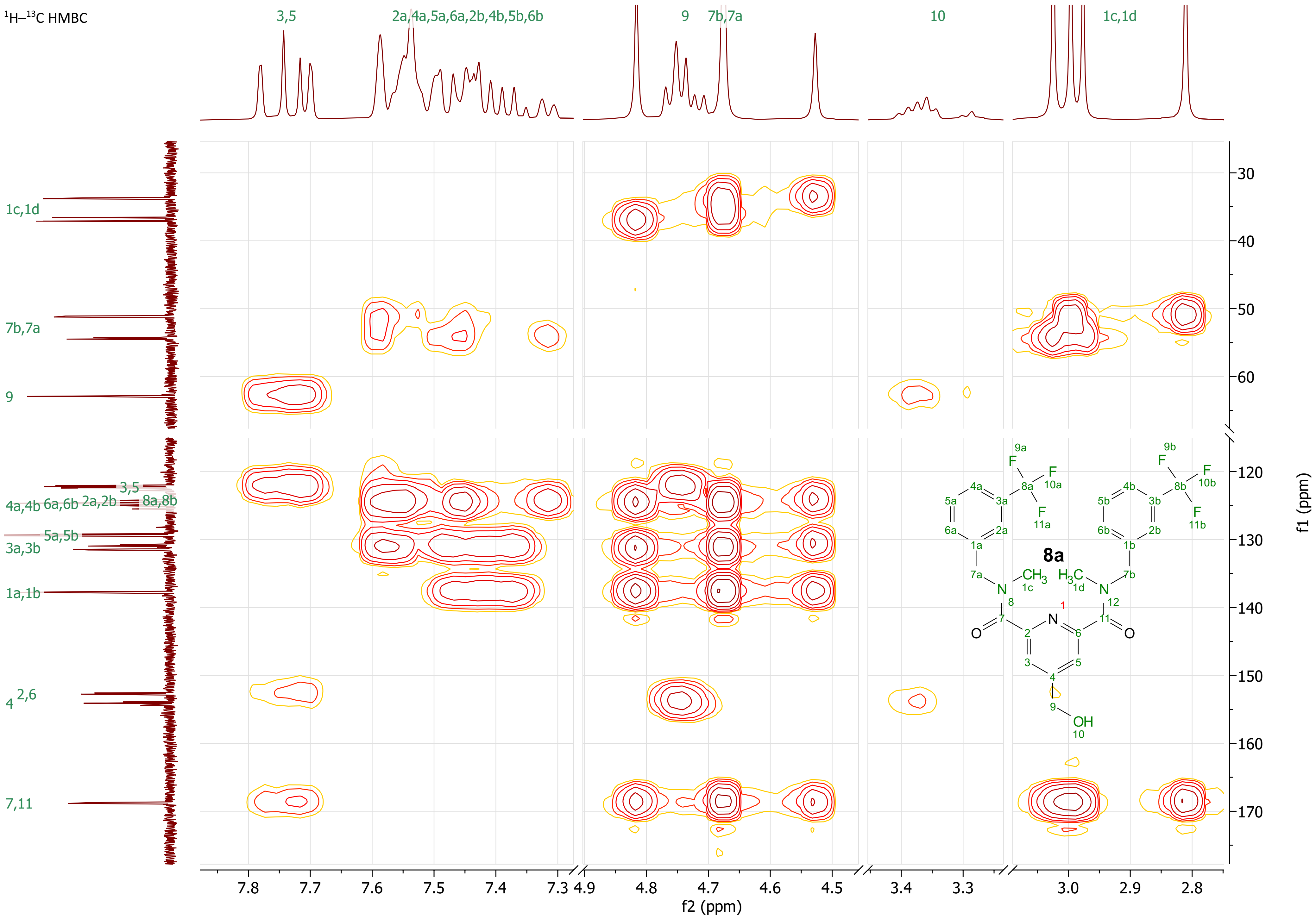
¹H–¹³C HMBC
3,5
2a,4a,5a,6a,2b,4b,5b,6b
9
7b,7a
10
1c,1d
1c,1d
7b,7a
9
3,5
4a,4b 6a,6b 2a,2b 8a,8b
5a,5b
3a,3b
1a,1b
4 2,6
7,11
8a
f2 (ppm)
f1 (ppm)

[Back ↑]

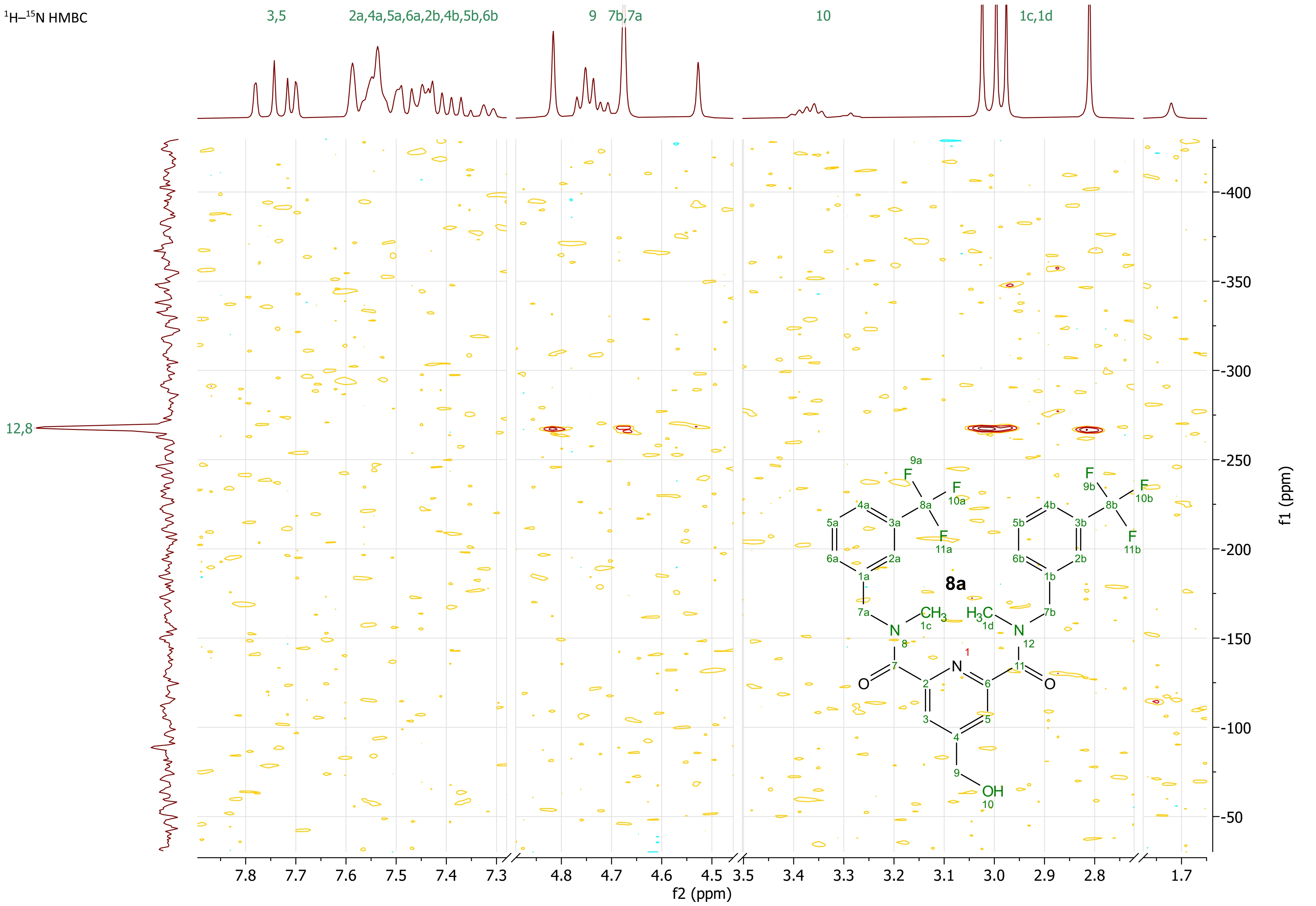

^{1}H–^{15}N HMBC
3,5
2a,4a,5a,6a,2b,4b,5b,6b
9
7b,7a
10
1c,1d
12,8
8a
f1 (ppm)
f2 (ppm)

^{15}N NMR (41 MHz, CDCl$_3$) δ -267.74. – Projection f1

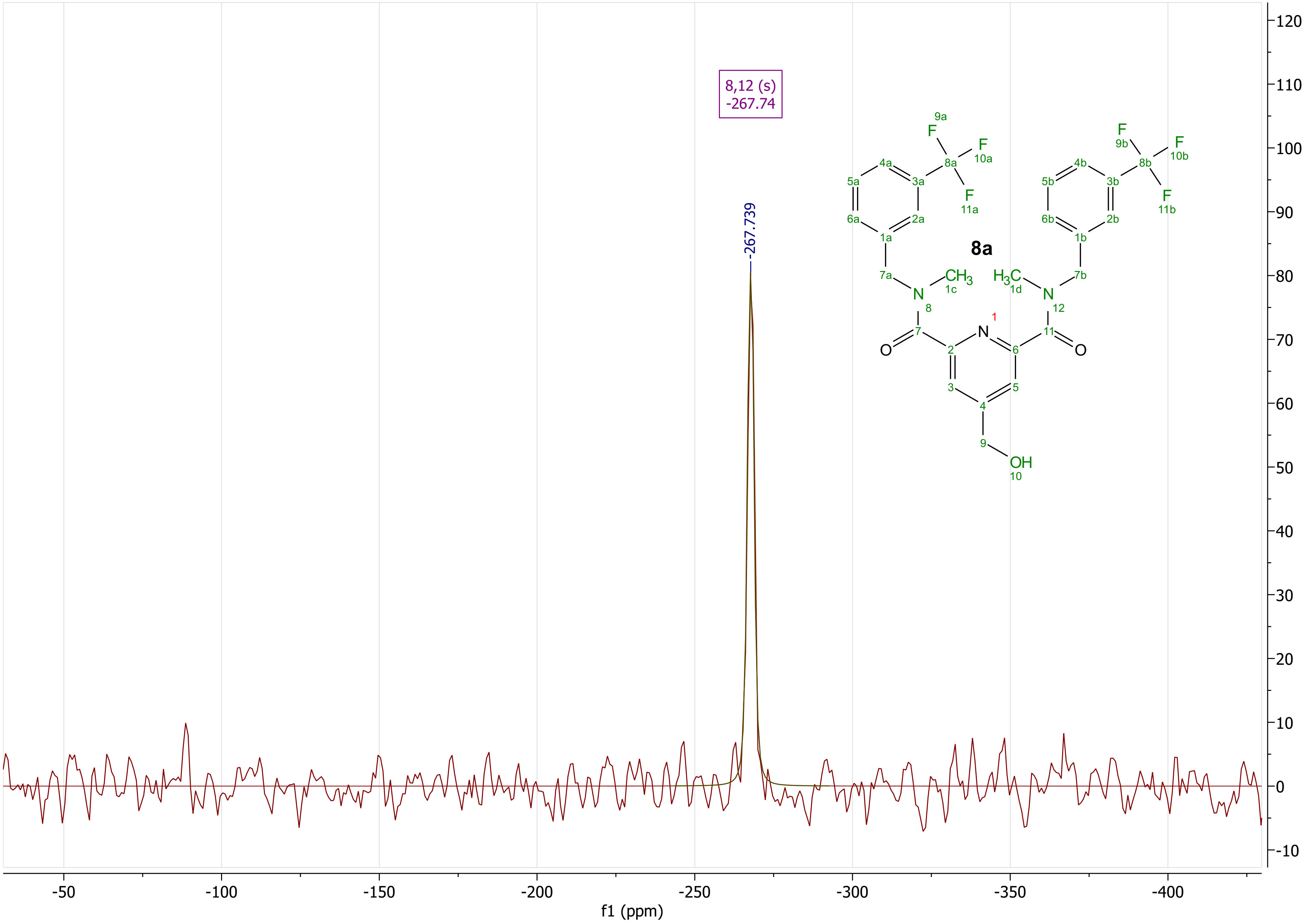

^{19}F NMR (376 MHz, $CDCl_3$) δ -51.75 – -76.71 (m).

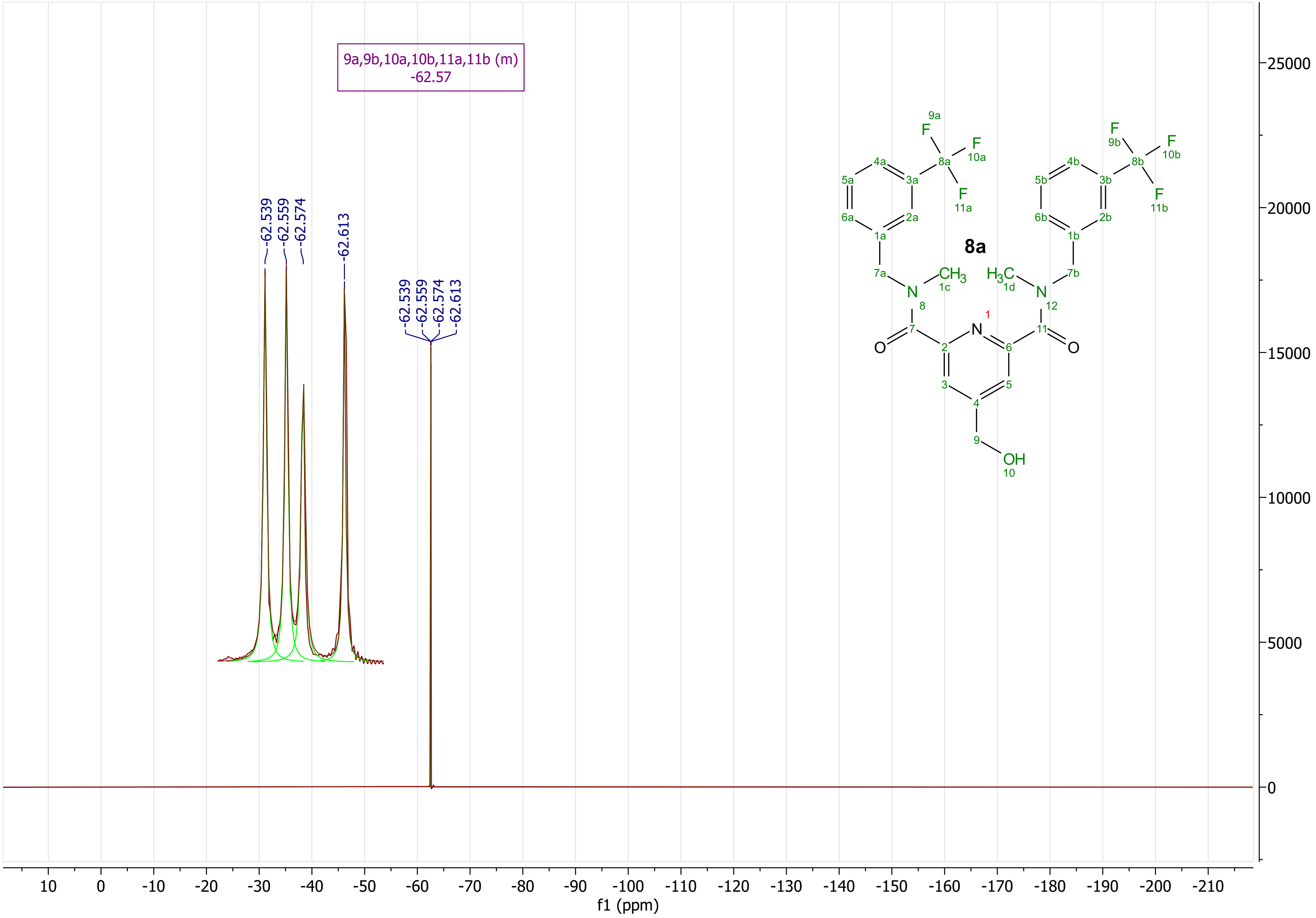

[Back ↑]

^{1}H NMR (400 MHz, DMSO-d_6) δ 7.70 – 7.59 (mm, 2H), 7.70 – 7.55 (mm, 4H), 7.70 – 7.44 (mm, 4H), 5.65 – 5.56 (mm, 1H), 4.83 – 4.43 (ms, 4H), 4.69 – 4.57 (mm, 2H), 2.95 – 2.63 (ms, 6H).

Mixture of conformers

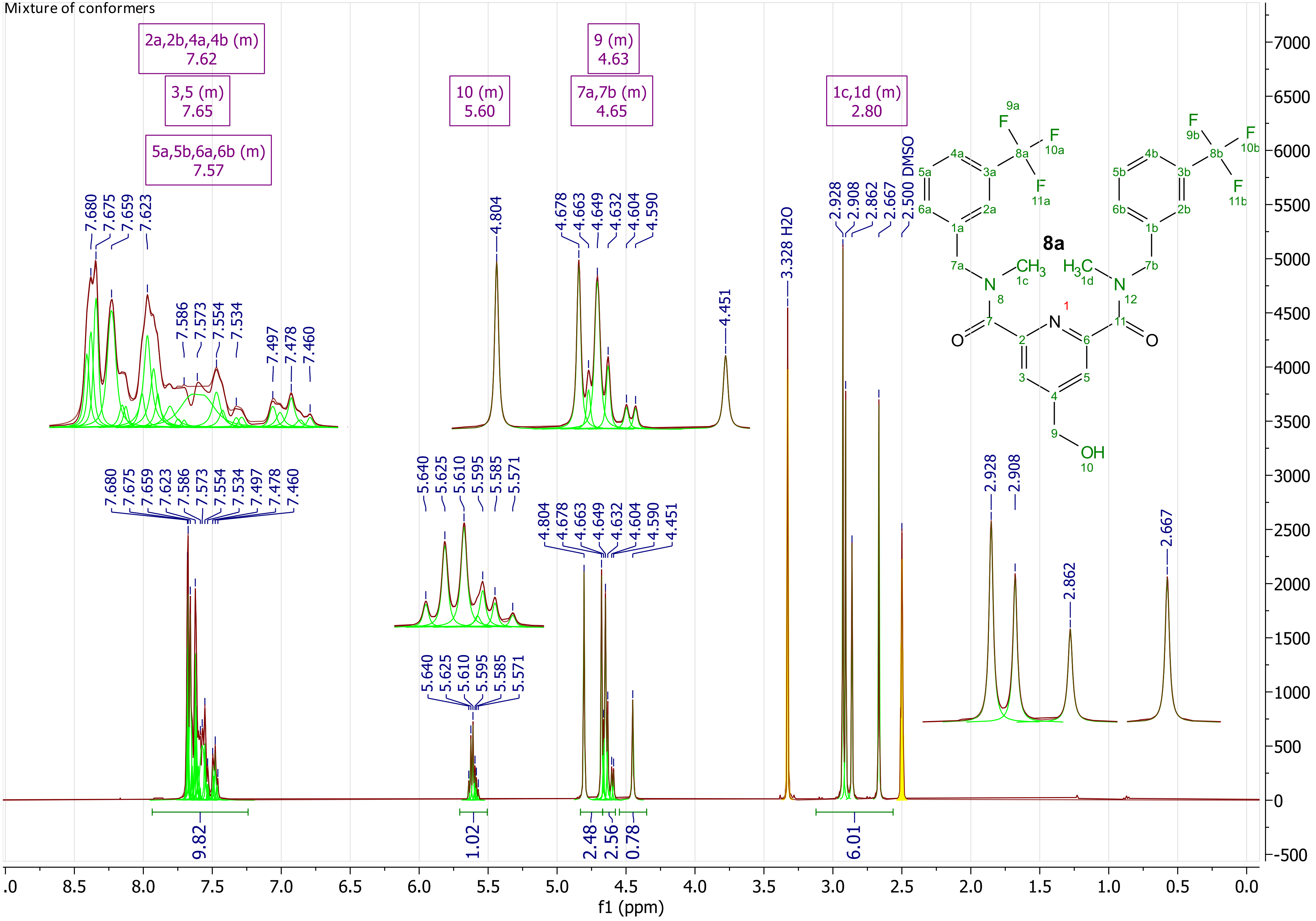

[Back ↑]

^{13}C NMR (101 MHz, DMSO-d_6) δ 168.2 – 167.9 (ms, 2C), 155.2 – 154.8 (ms), 152.7 – 152.5 (ms, 2C), 138.8 – 138.3 (ms, 2C), 131.7 – 131.2 (ms, 2C), 129.8 – 129.3 (ms, 2C), 129.7 – 128.7 (mm, 2C), 128.4 – 119.7 (mm, 2C), 124.6 – 123.8 (mm, 4C), 121.2 – 120.8 (ms, 2C), 61.4 – 61.1 (ms), 53.3 – 49.5 (ms, 2C), 36.8 – 32.6 (ms, 2C). Mixture of conformers

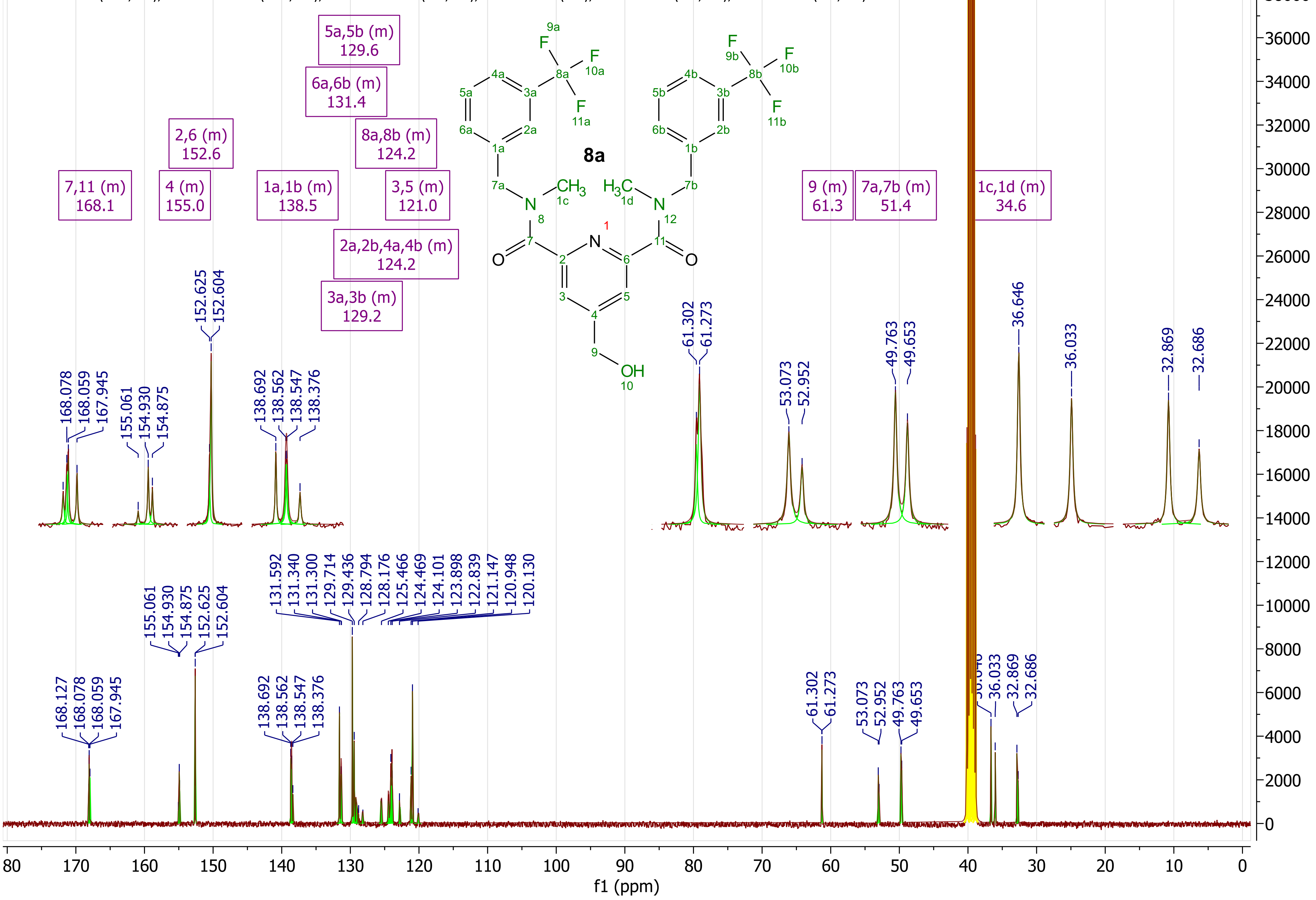

^{13}C NMR (101 MHz, DMSO-d_6) – [132.5 – 119.5 ppm]

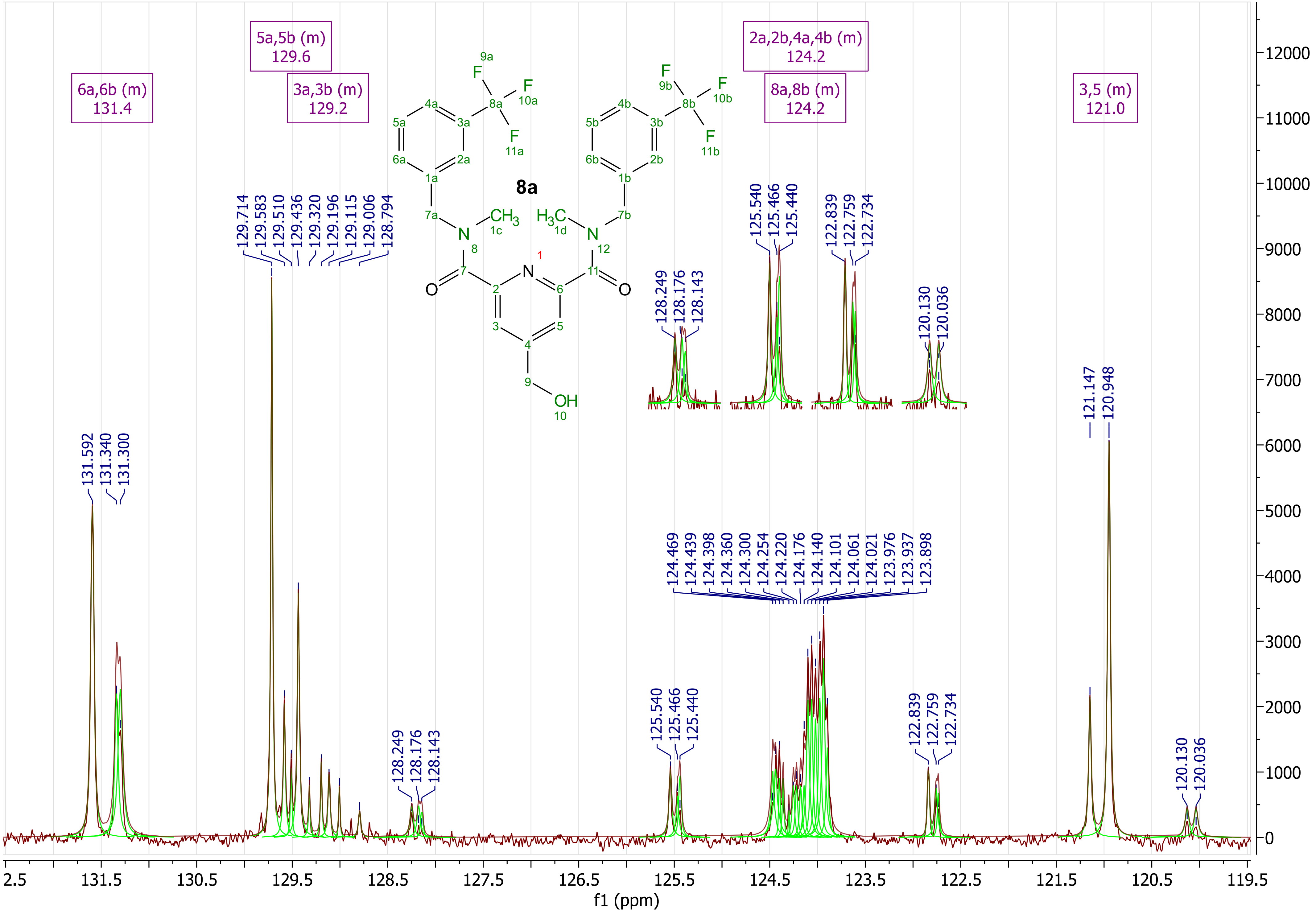

^1H–^{13}C HSQC

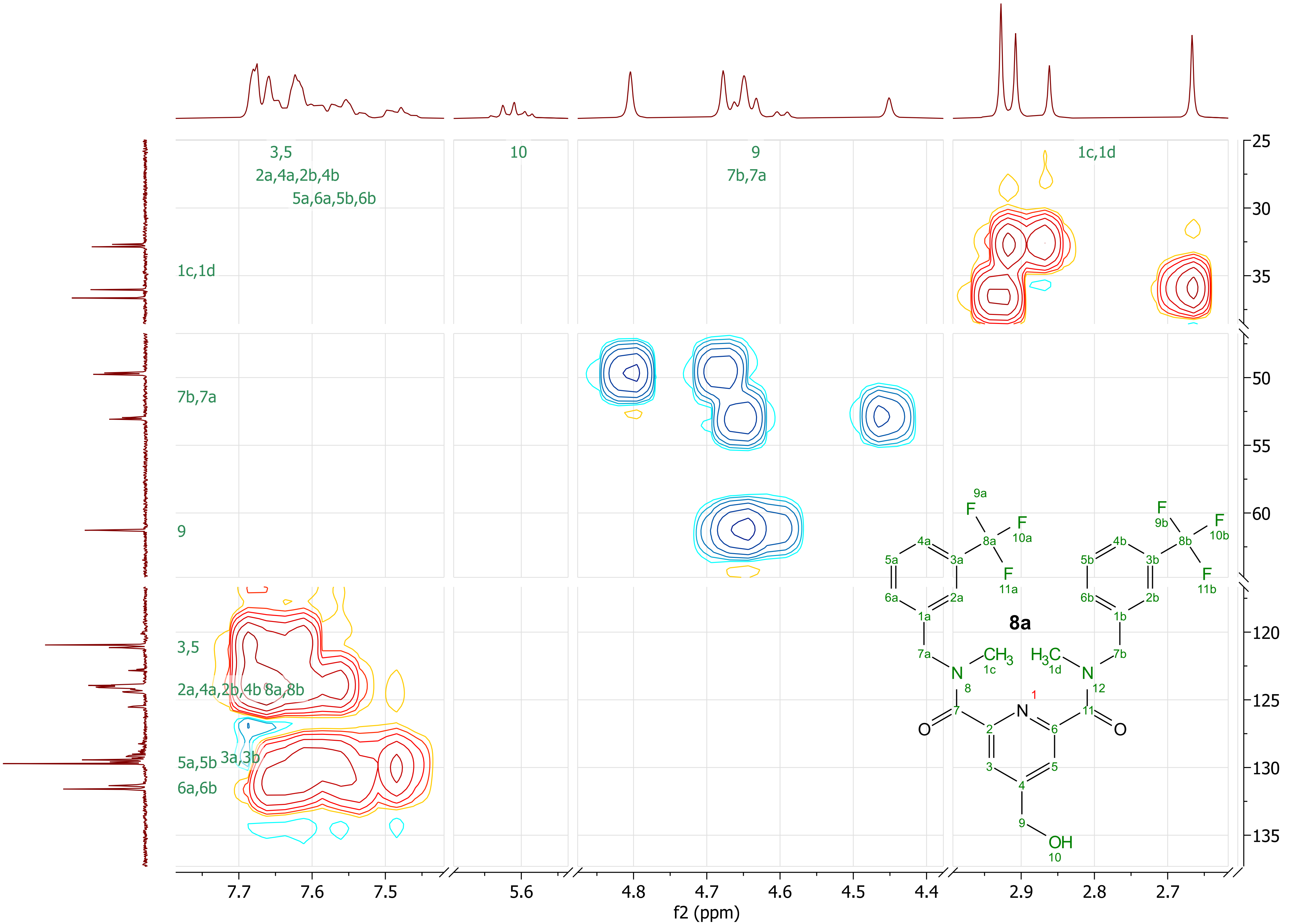
3,5
2a,4a,2b,4b
5a,6a,5b,6b
10
9
7b,7a
1c,1d
1c,1d
7b,7a
9
3,5
2a,4a,2b,4b 8a,8b
5a,5b 3a,3b
6a,6b
8a
f2 (ppm)
f1 (ppm)

$^{1}H–^{13}C$ HMBC

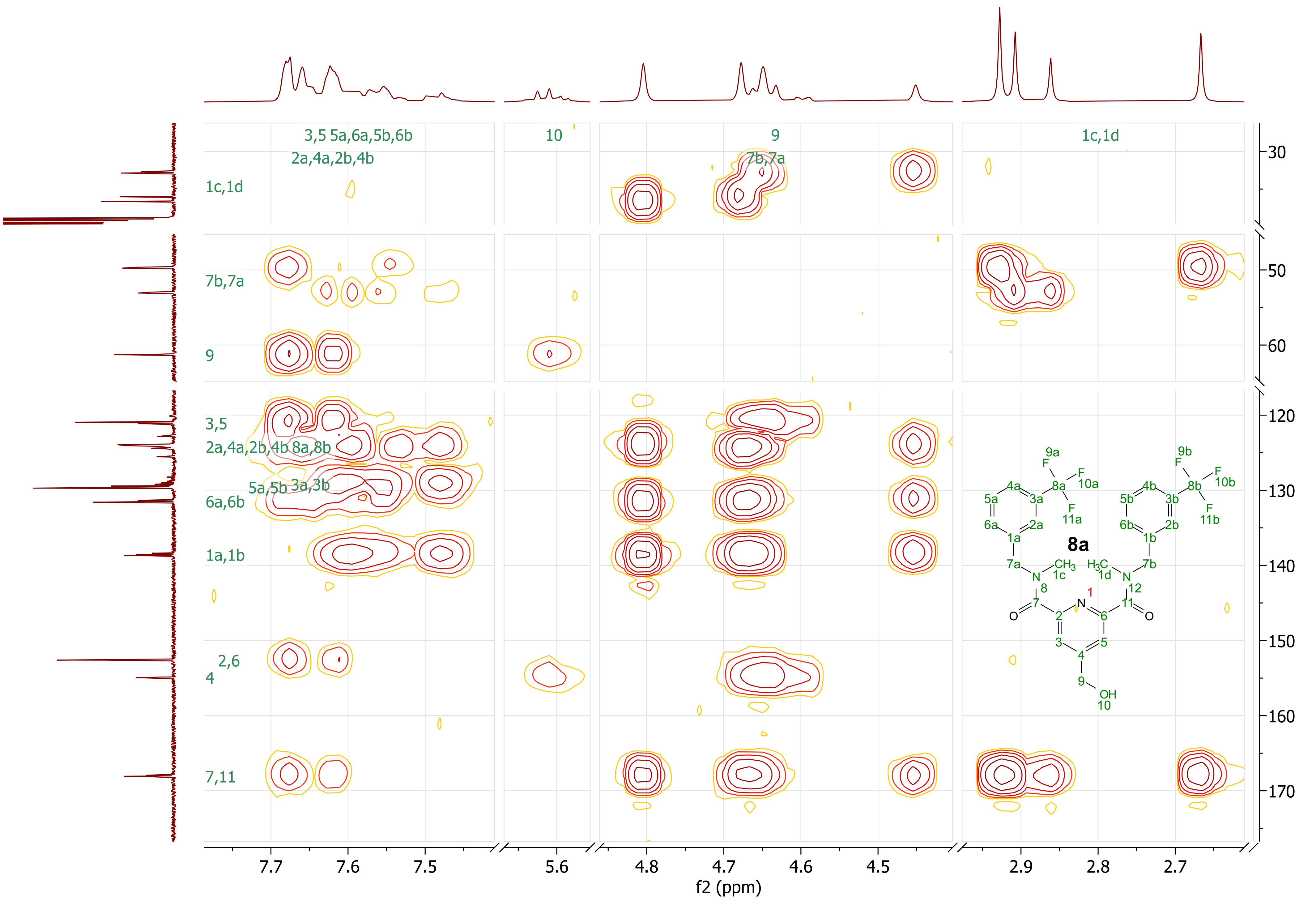
3,5 5a,6a,5b,6b
2a,4a,2b,4b
10
9
7b,7a
1c,1d
1c,1d
7b,7a
9
3,5
2a,4a,2b,4b 8a,8b
5a,5b 3a,3b
6a,6b
1a,1b
2,6
4
7,11
8a
f1 (ppm)
f2 (ppm)
7.7
7.6
7.5
5.6
4.8
4.7
4.6
4.5
2.9
2.8
2.7
30
50
60
120
130
140
150
160
170

^{1}H–^{15}N HMBC

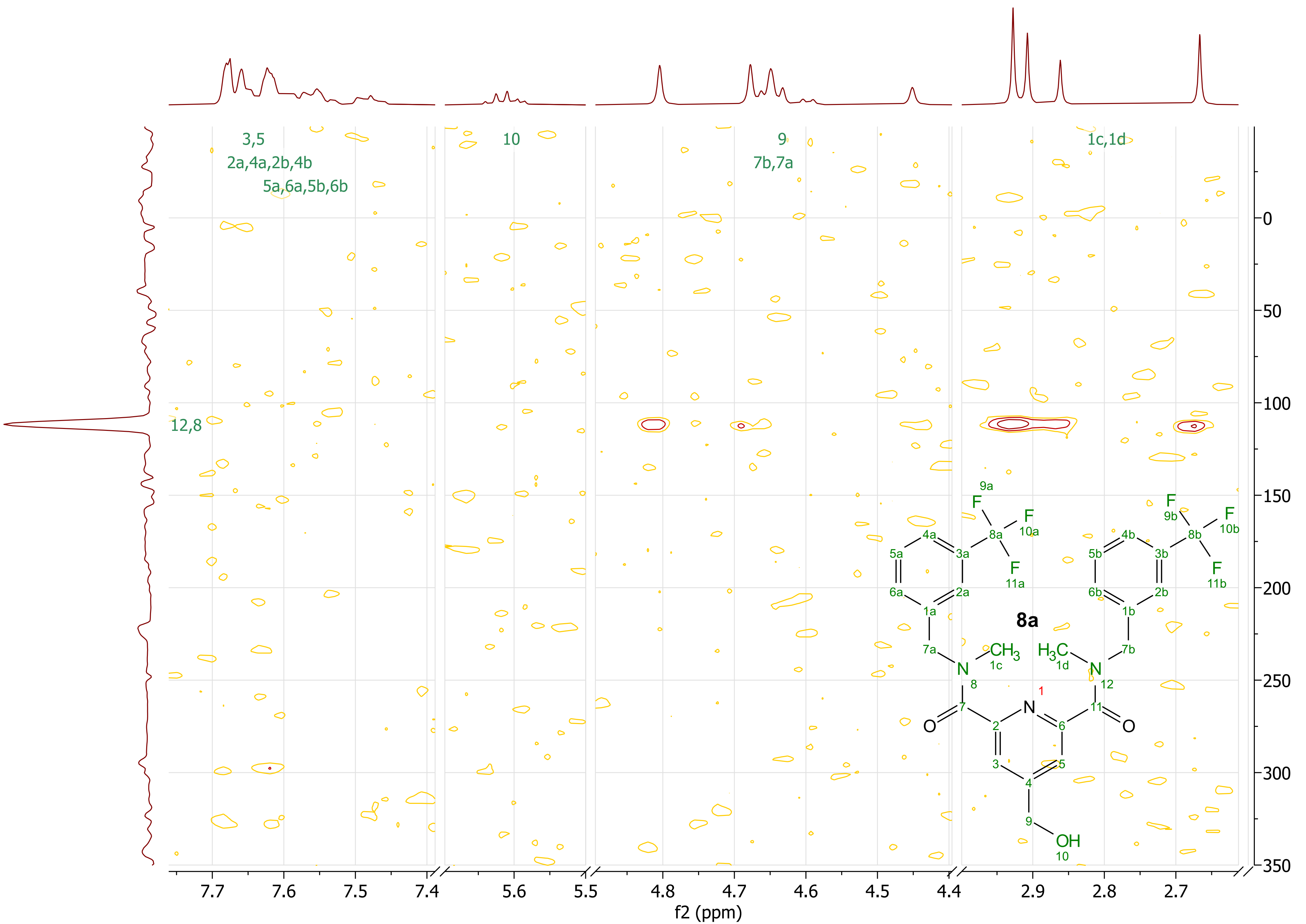

^{15}N NMR (41 MHz, DMSO-d_6) δ 111.68. – Projection f1

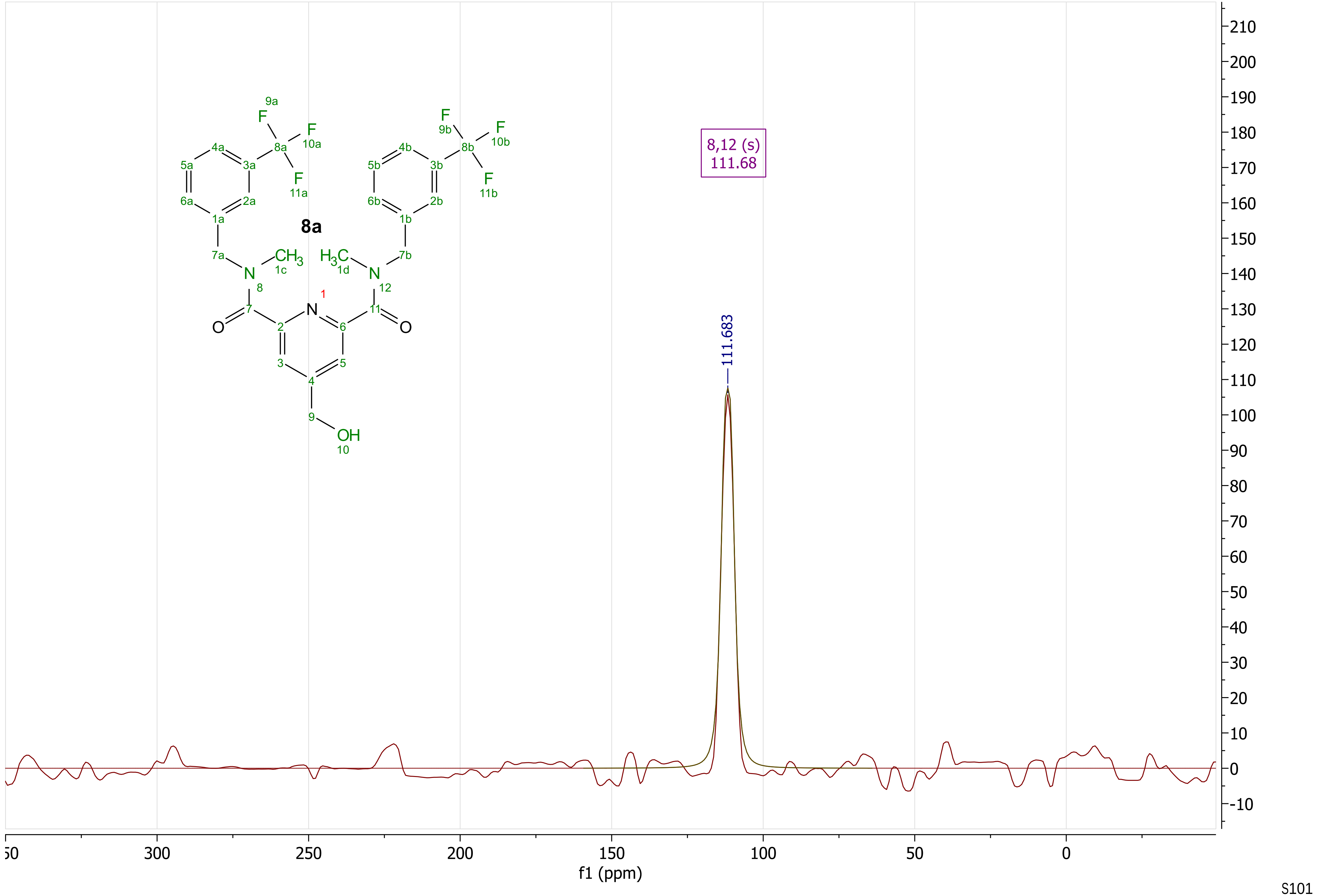

[Back ↑]

^{19}F NMR (376 MHz, DMSO-d_6) δ -58.40 – -62.88 (m).

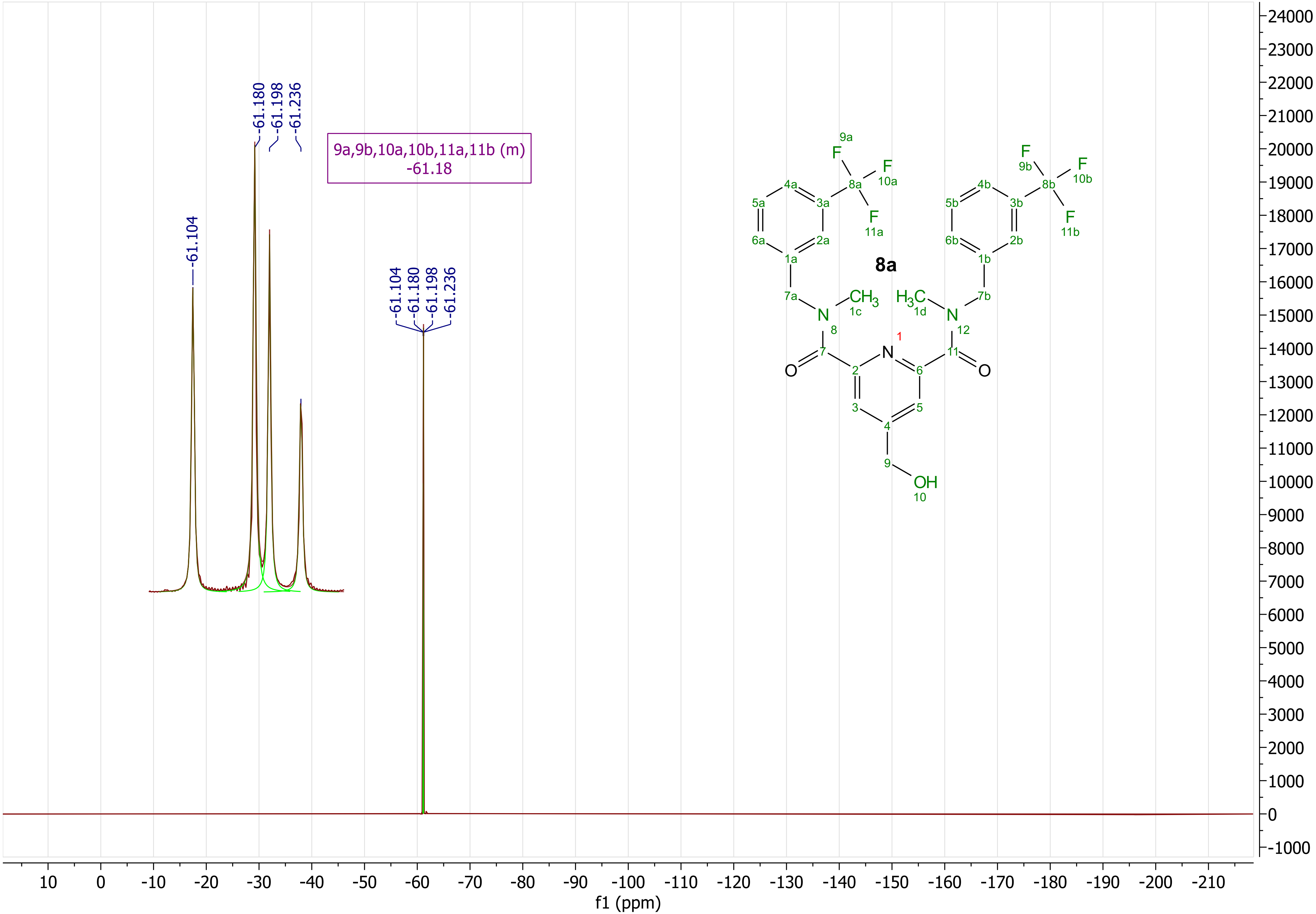

[Back ↑]

^{1}H NMR (400 MHz, DMSO-d_6, 100 °C) δ 7.72 – 7.48 (m, 10H), 5.29 (br s, 1H), 4.73 (br s, 4H), 4.64 (s, 2H), 2.92 (br s, 6H). – Mixture of conformers

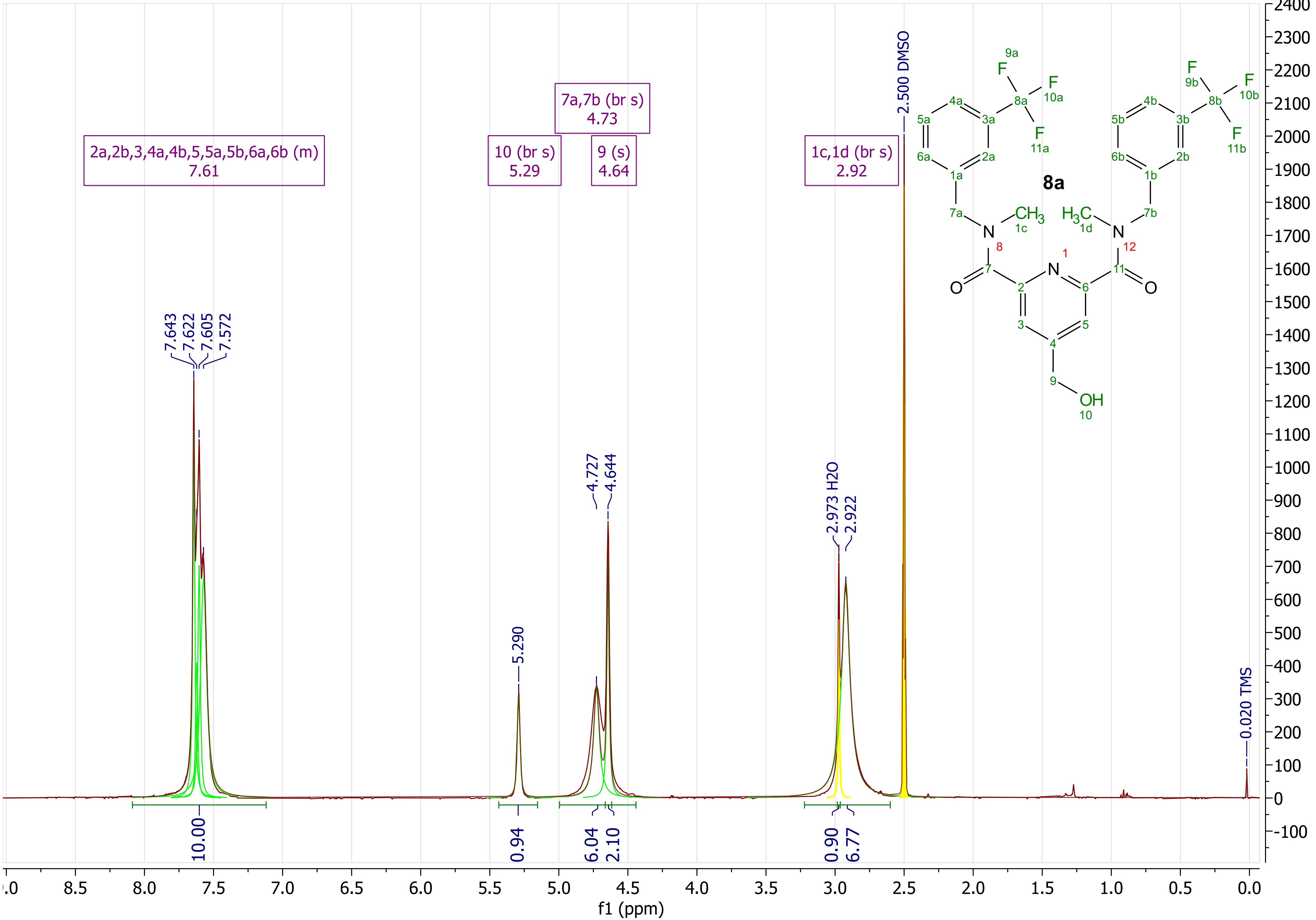

[Back ↑]

^{13}C NMR (101 MHz, DMSO-d_6, 100 °C) δ 167.5 (2C), 154.2, 152.4 (2C), 138.2 (2C), 130.9 (2C), 129.1 (q, J = 31.7 Hz, 2C), 128.9 (2C), 123.6 (q, J = 272.4 Hz, 2C), 124.1 – 122.9 (m, 4C), 120.5 (2C), 61.1, 53.5 – 48.8 (ms, 2C), 36.6 – 31.8 (ms, 2C). – Mixture of conformers

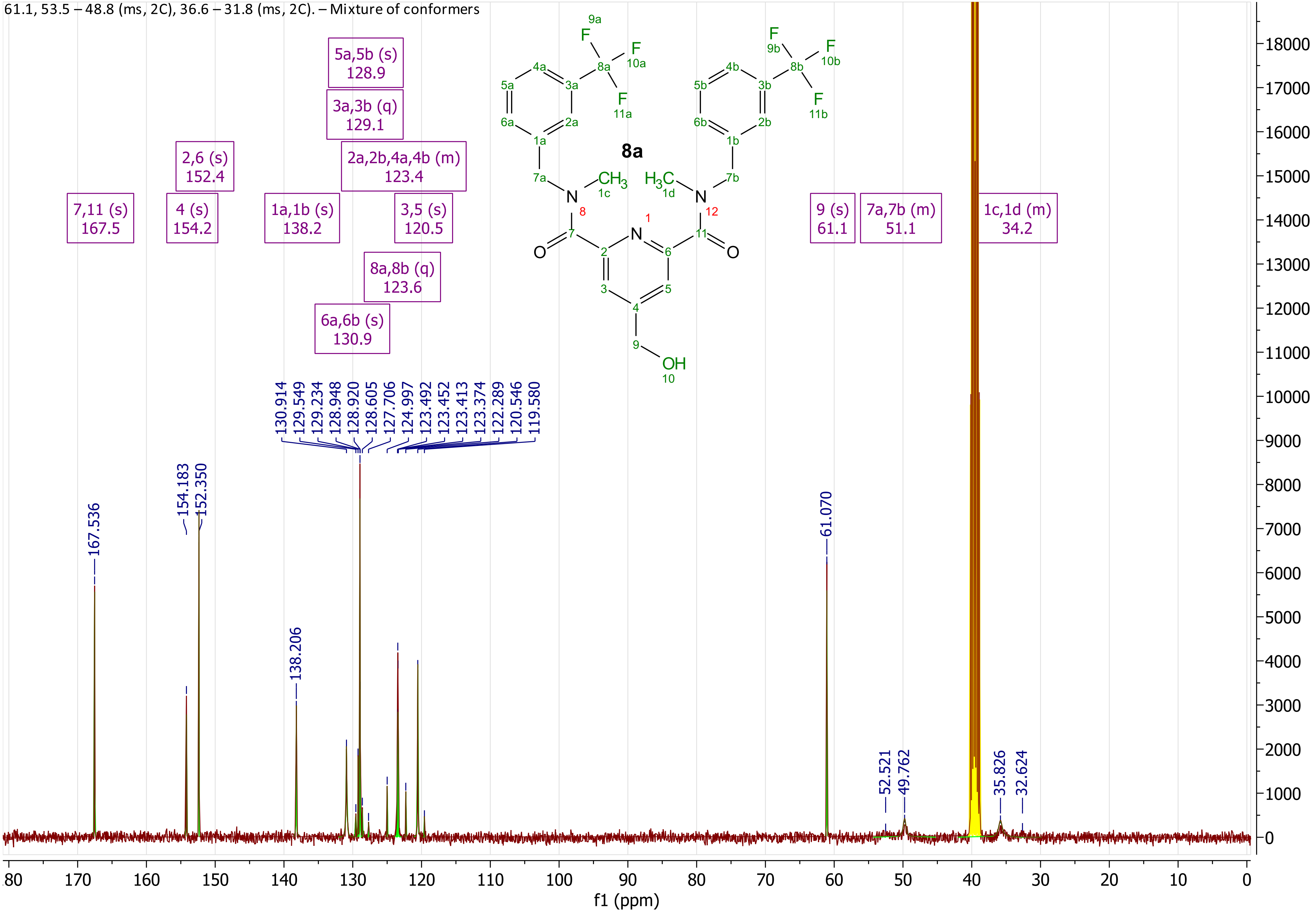

^{13}C NMR (101 MHz, DMSO-d_6, 100 °C) – [132.5 – 119.5 ppm]

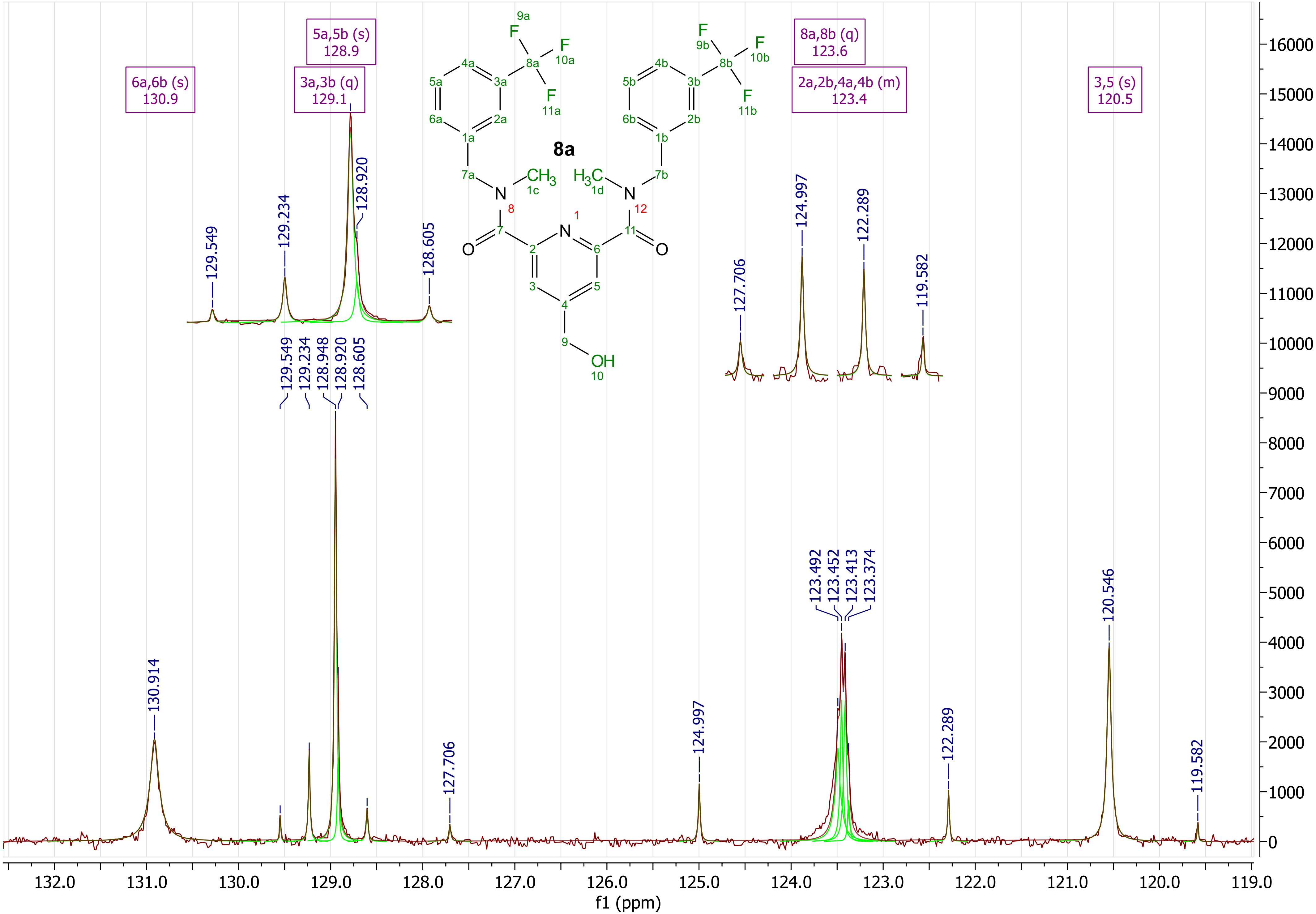

[Back ↑]

^{19}F NMR (376 MHz, DMSO-d_6, 100 °C) δ -61.32.

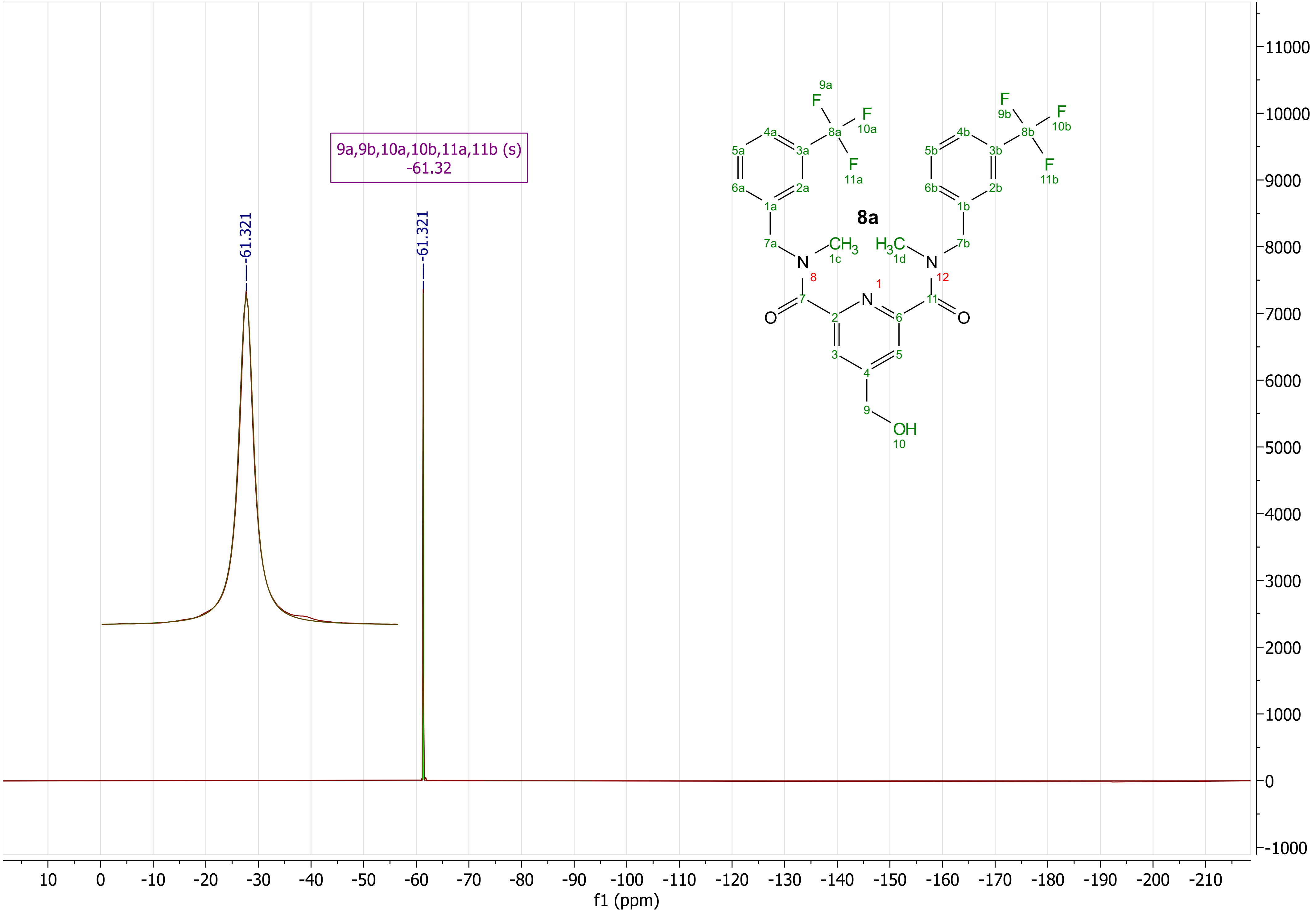

[Back ↑]

^{1}H NMR (400 MHz) – Spectra comparison

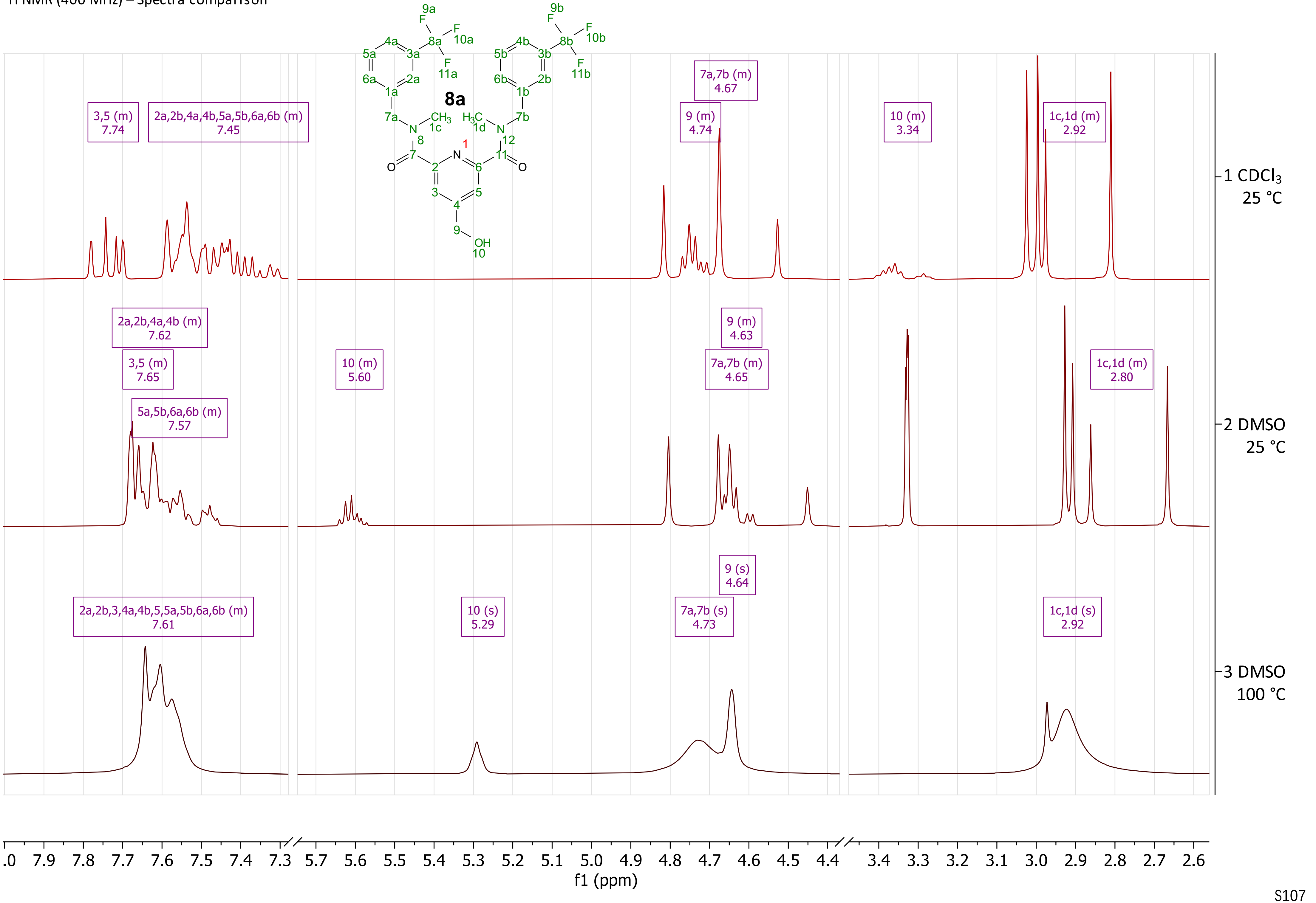

^{13}C NMR (101 MHz) – Spectra comparison

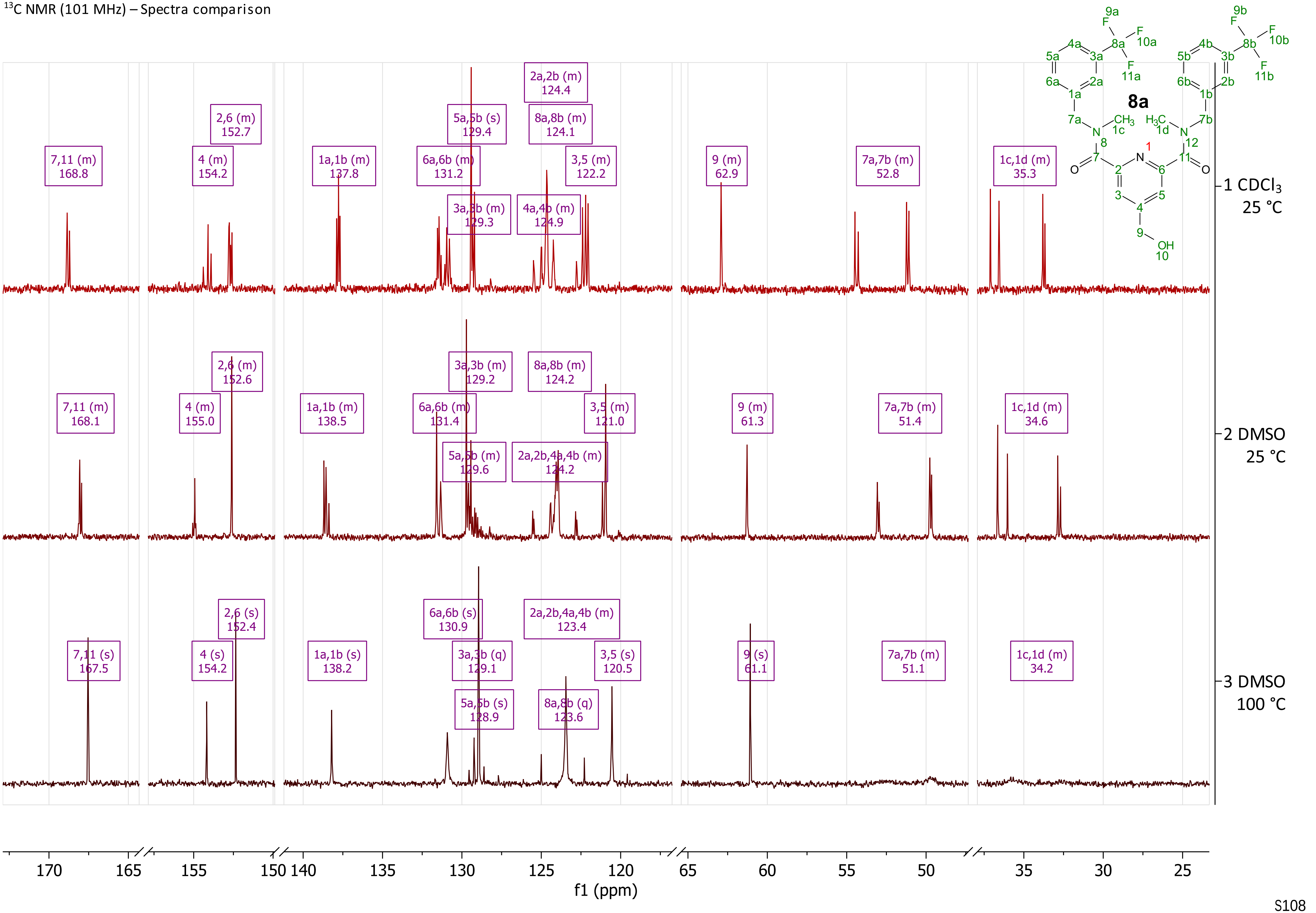

[Back ↑]

^{13}C NMR (101 MHz) – Spectra comparison [132.5 – 119 ppm]

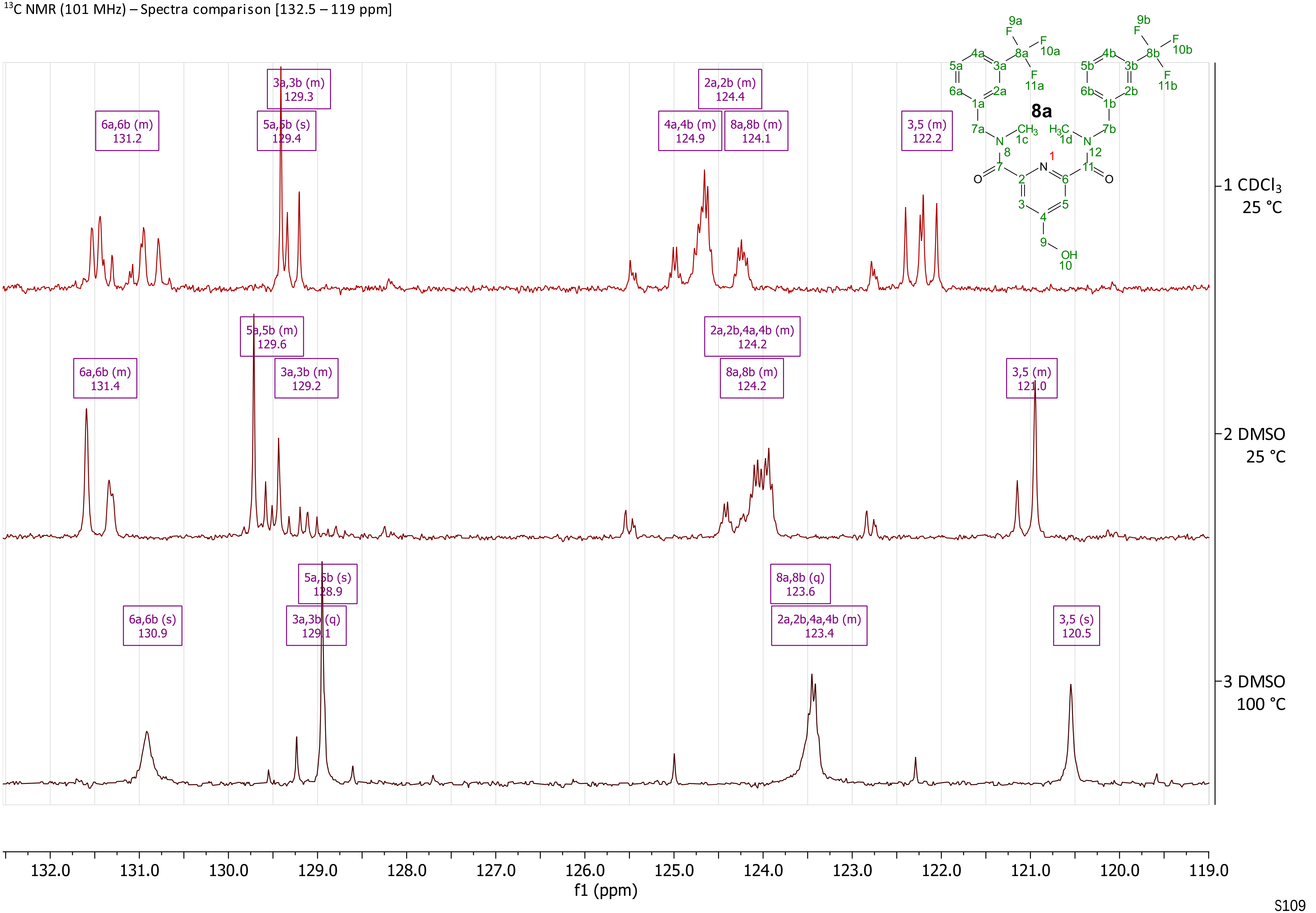

[Back ↑]

^{1}H NMR (400 MHz, $CDCl_3$) δ 7.60 – 7.45 (mm, 2H), 4.75 – 4.66 (mm, 2H), 4.66 – 3.26 (mm, 3H), 2.93 – 2.63 (ms, 6H), 1.62 – 1.37 (mm, 4H), 1.61 – 1.25 (mm, 4H), 1.40 – 1.06 (mm, 8H), 0.97 – 0.71 (mm, 12H). – Mixture of conformers.

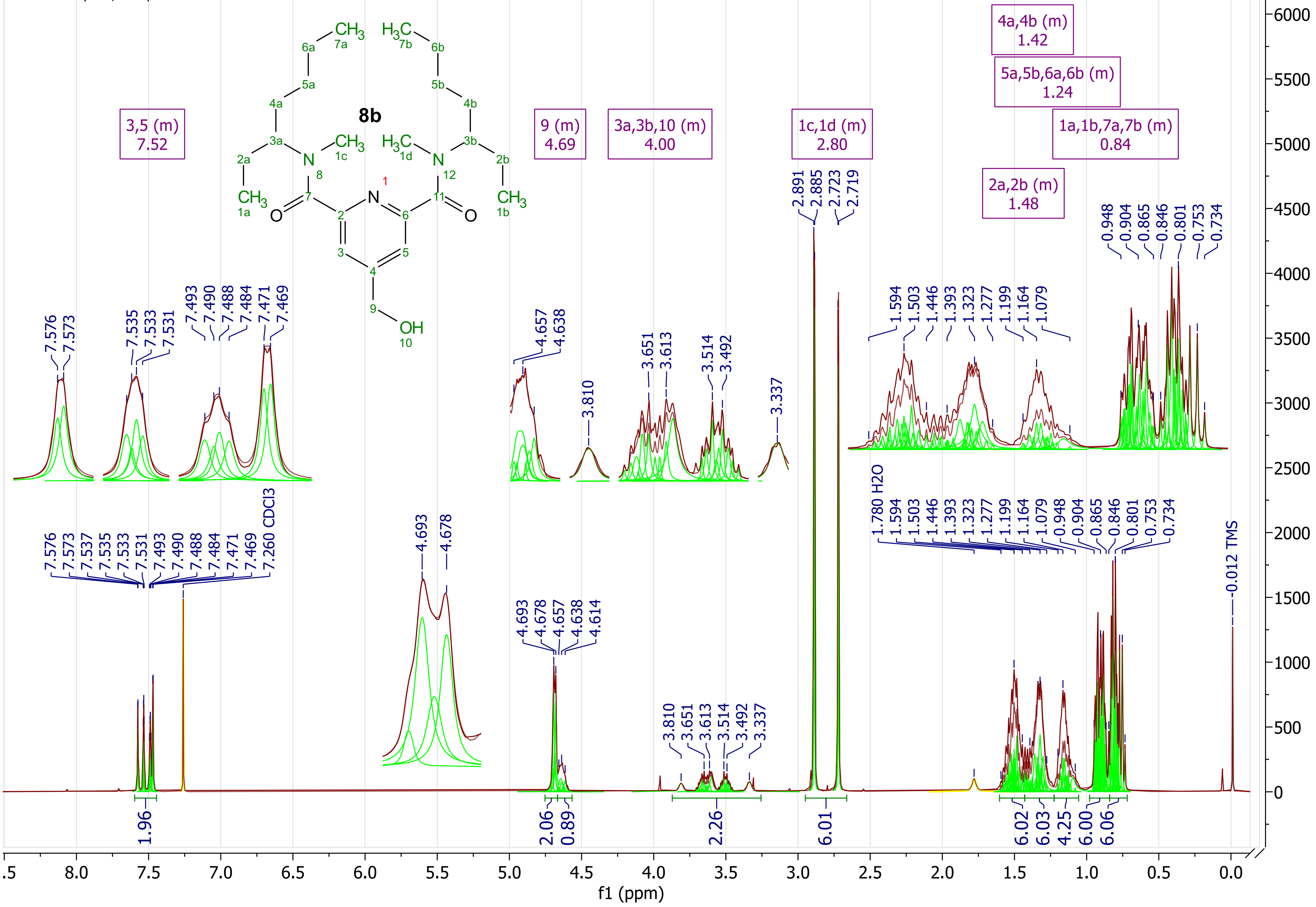

[Back ↑]

^{13}C NMR (101 MHz, $CDCl_3$) δ 170.3 – 169.5 (ms, 2C), 154.4 – 153.3 (ms, 3C), 121.2 – 120.6 (ms, 2C), 63.1 – 62.8 (ms), 60.0 – 54.6 (ms, 2C), 32.8 – 31.6 (ms, 2C), 28.6 – 28.2 (ms, 2C), 30.4 – 25.8 (ms, 2C), 25.4 – 25.0 (ms, 2C), 22.8 – 22.6 (ms, 2C), 14.3 – 13.9 (ms, 2C), 11.0 – 10.6 (ms, 2C). – Mixture of conformers.

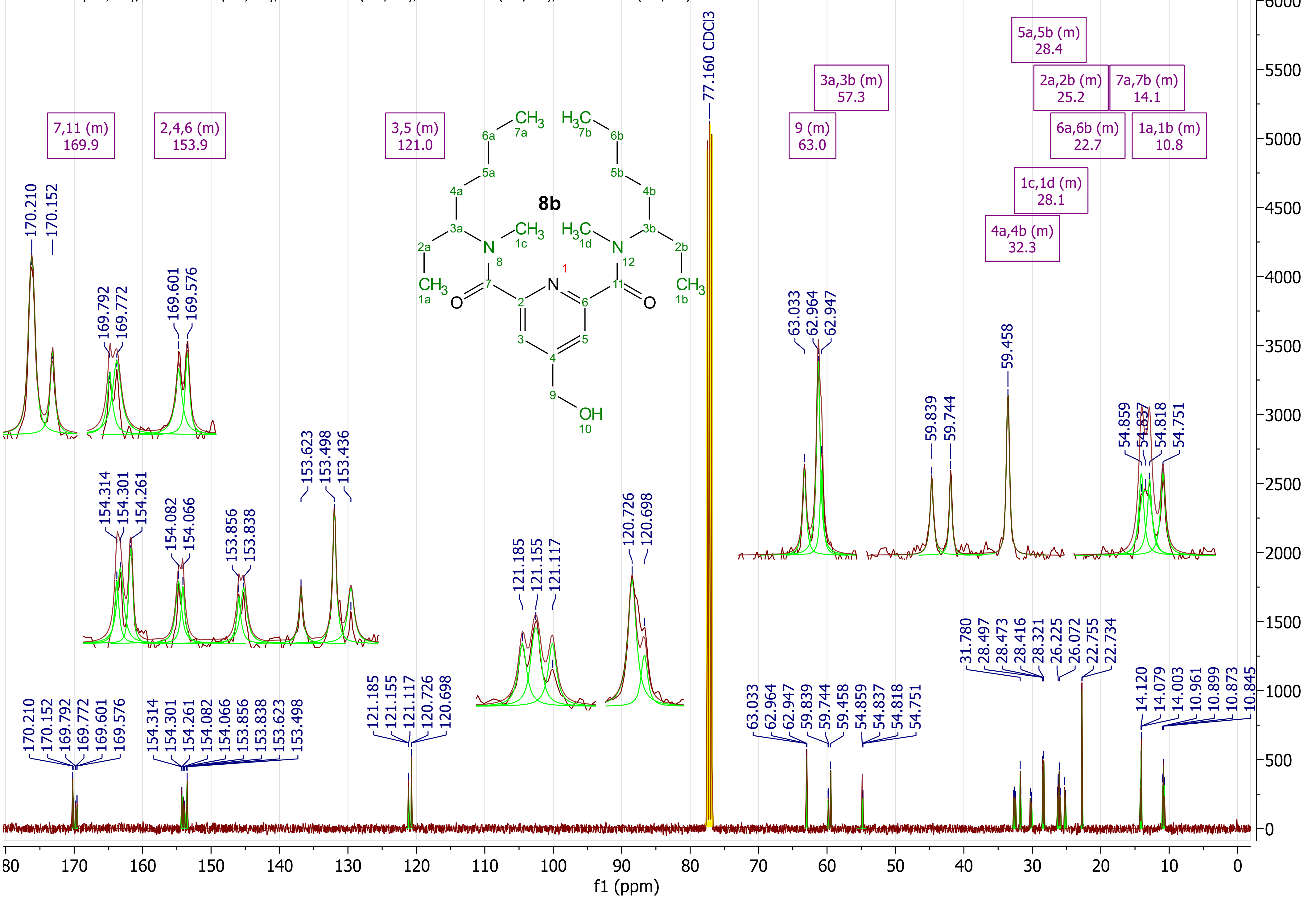

[Back ↑]

^{13}C NMR (101 MHz, $CDCl_3$) – [34 – 10 ppm]

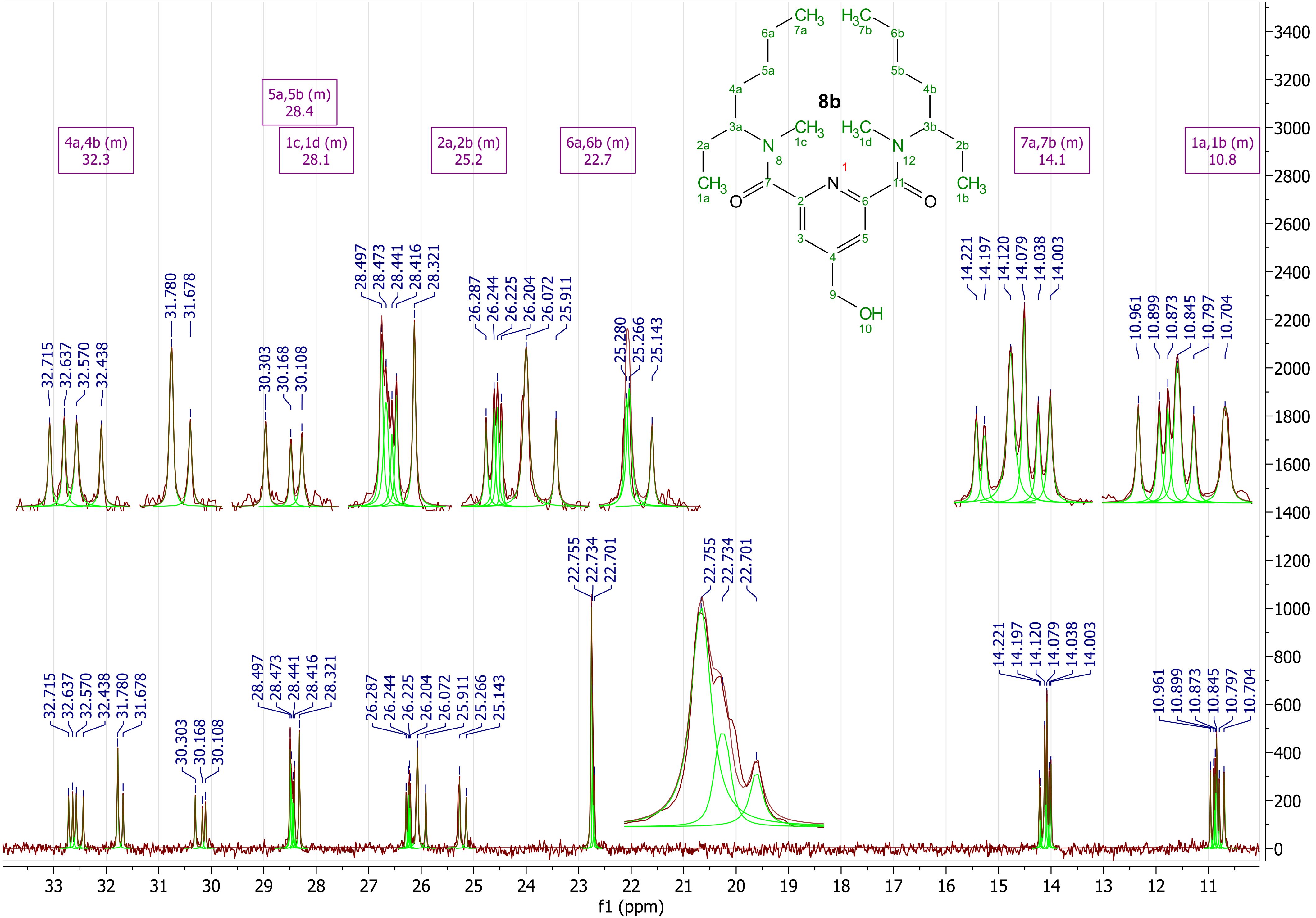

[Back ↑]

^{1}H–^{13}C HSQC

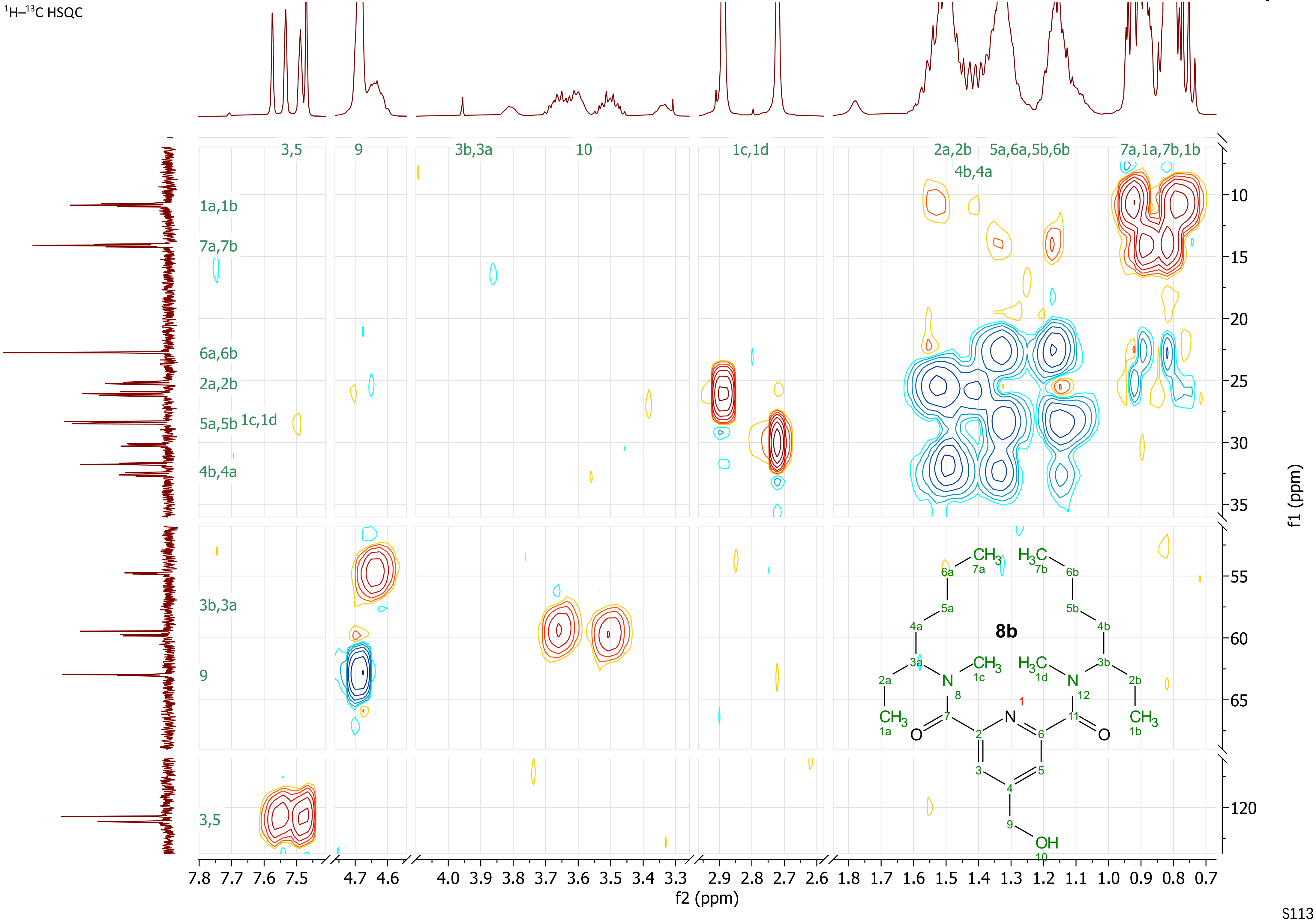

[Back ↑]

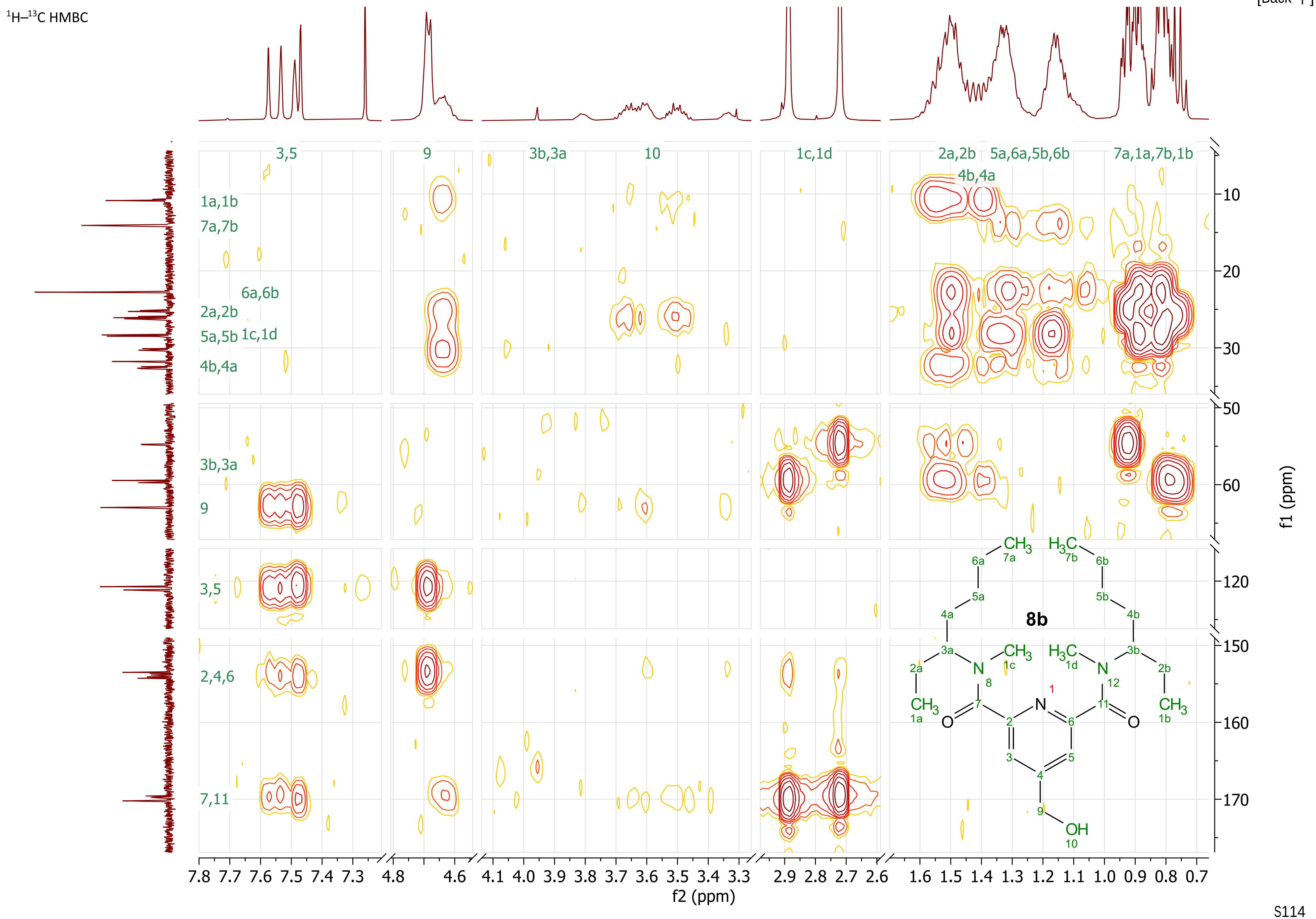

¹H–¹³C HMBC
3,5
9
3b,3a
10
1c,1d
2a,2b
5a,6a,5b,6b
7a,1a,7b,1b
4b,4a
1a,1b
7a,7b
6a,6b
2a,2b
5a,5b
1c,1d
4b,4a
3b,3a
9
3,5
2,4,6
7,11
8b
f1 (ppm)
f2 (ppm)

^{1}H–^{15}N HMBC

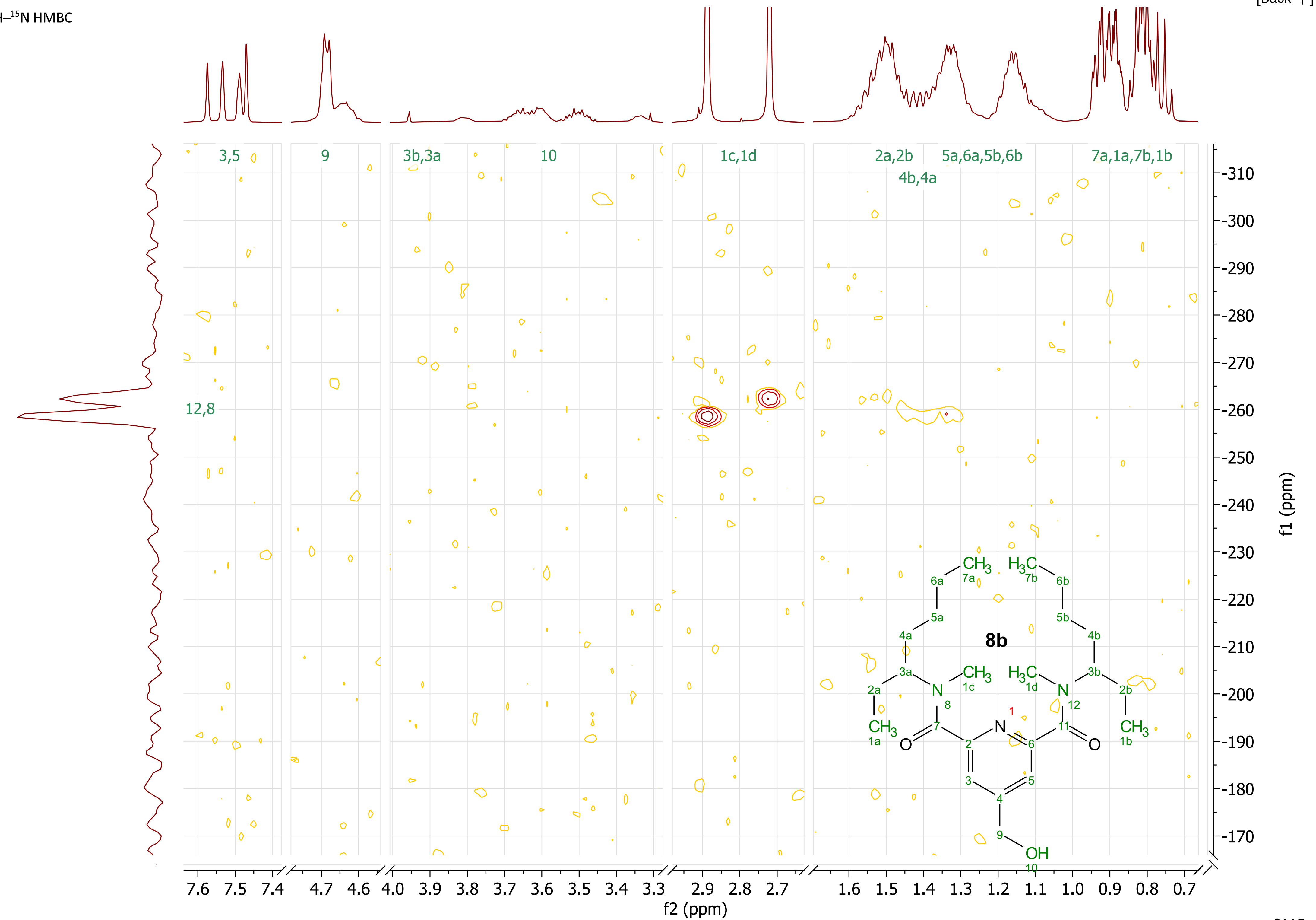
3,5
9
3b,3a
10
1c,1d
2a,2b
4b,4a
5a,6a,5b,6b
7a,1a,7b,1b
12,8
8b
f1 (ppm)
f2 (ppm)
OH

[Back ↑]

^{15}N NMR (41 MHz, $CDCl_3$) δ -242.25 – -278.23 (m). – Projection f1

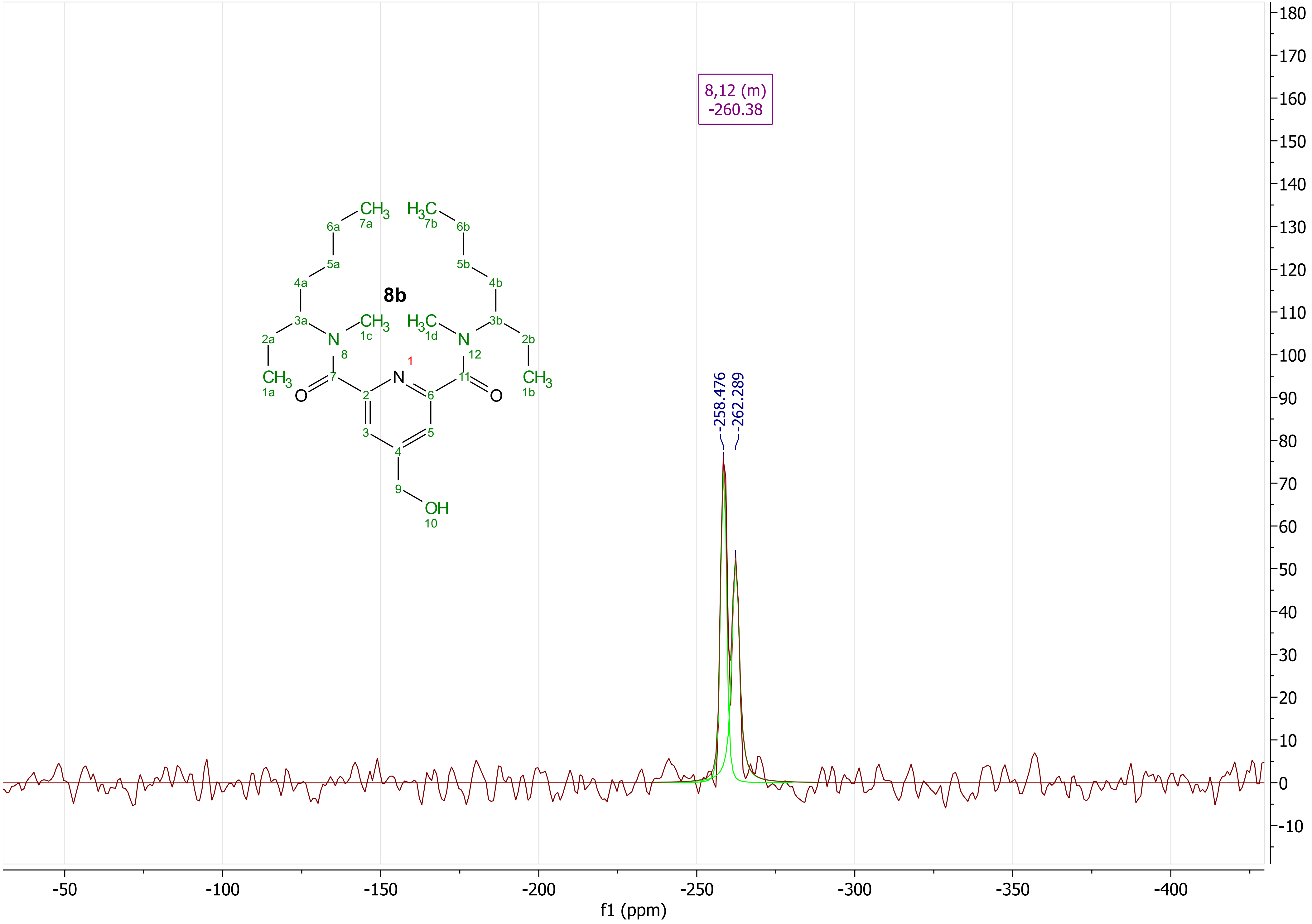

[Back ↑]

^{1}H NMR (400 MHz, DMSO-d_6) δ 7.52 – 7.30 (mm, 2H), 5.59 (br s, 1H), 4.68 – 4.54 (mm, 2H), 4.54 – 3.28 (mm, 2H), 2.79 – 2.58 (ms, 6H), 1.58 – 1.41 (mm, 4H), 1.59 – 1.30 (mm, 4H), 1.32 – 1.03 (mm, 8H), 0.90 – 0.67 (m-, 12H). – Mixture of conformers.

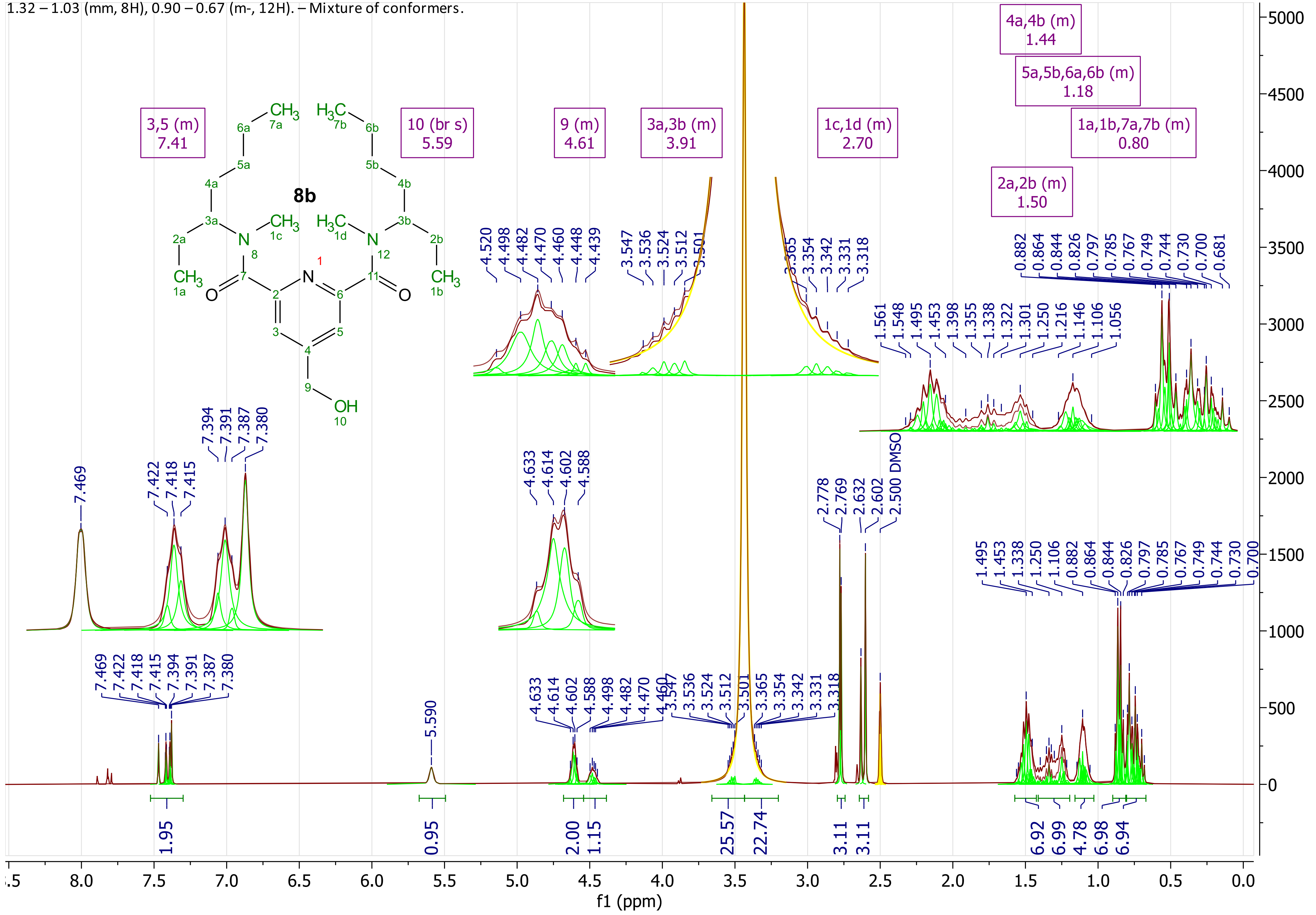

[Back ↑]

^{13}C NMR (101 MHz, DMSO-d_6) δ 169.2 – 168.8 (ms, 2C), 154.7 – 153.8 (ms, 3C), 120.5 – 119.2 (ms, 2C), 61.5 – 61.2 (ms), 59.0 – 53.4 (ms, 2C), 32.0 – 30.7 (ms, 2C), 28.1 – 27.7 (ms, 2C), 29.8 – 25.0 (ms, 2C), 24.6 – 24.3 (ms, 2C), 22.2 – 21.9 (ms, 2C), 14.1 – 13.8 (ms, 2C), 10.8 – 10.5 (ms, 2C). – Mixture of conformers.

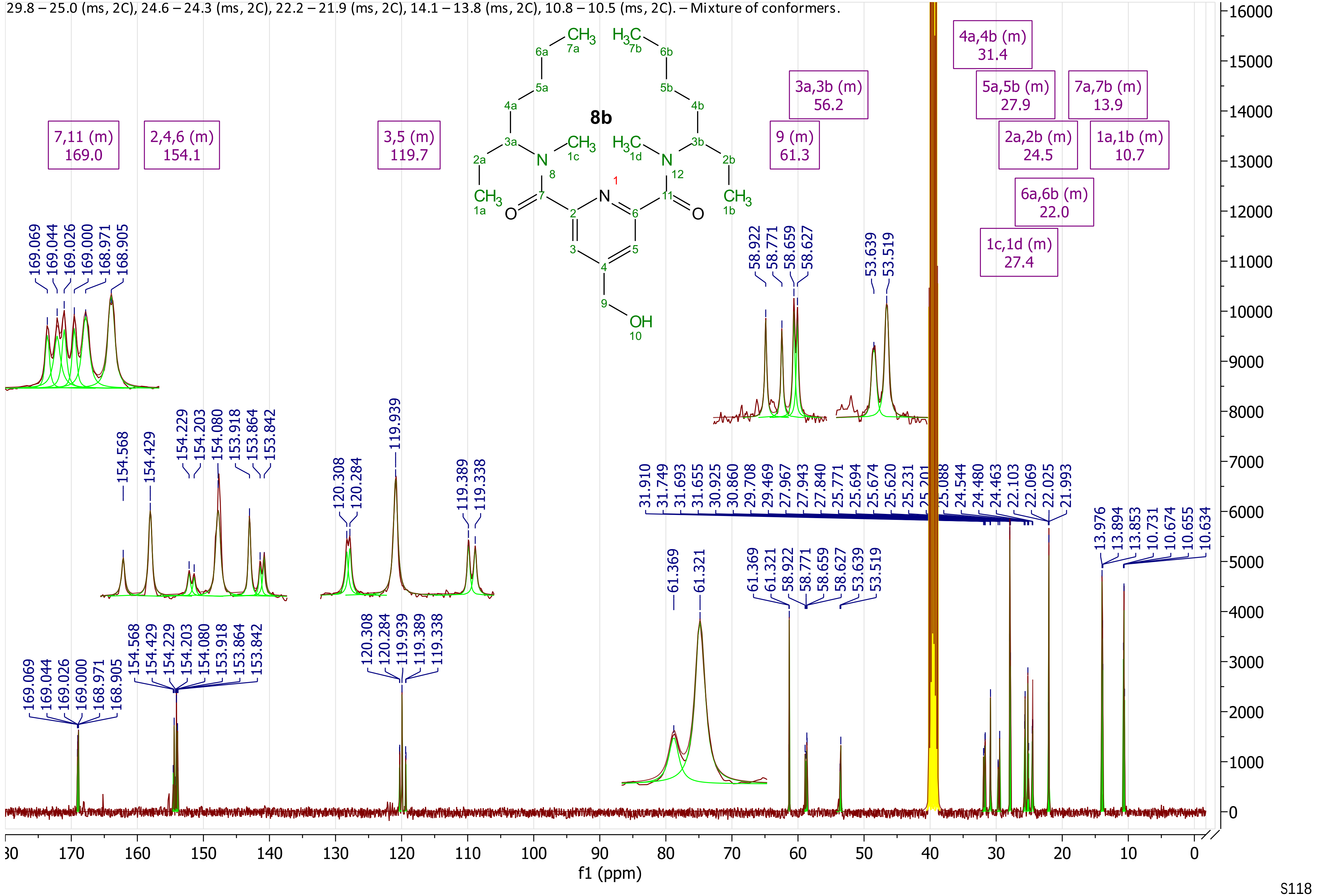

[Back ↑]

^{13}C NMR (101 MHz, DMSO-d_6) – [34 – 10 ppm]

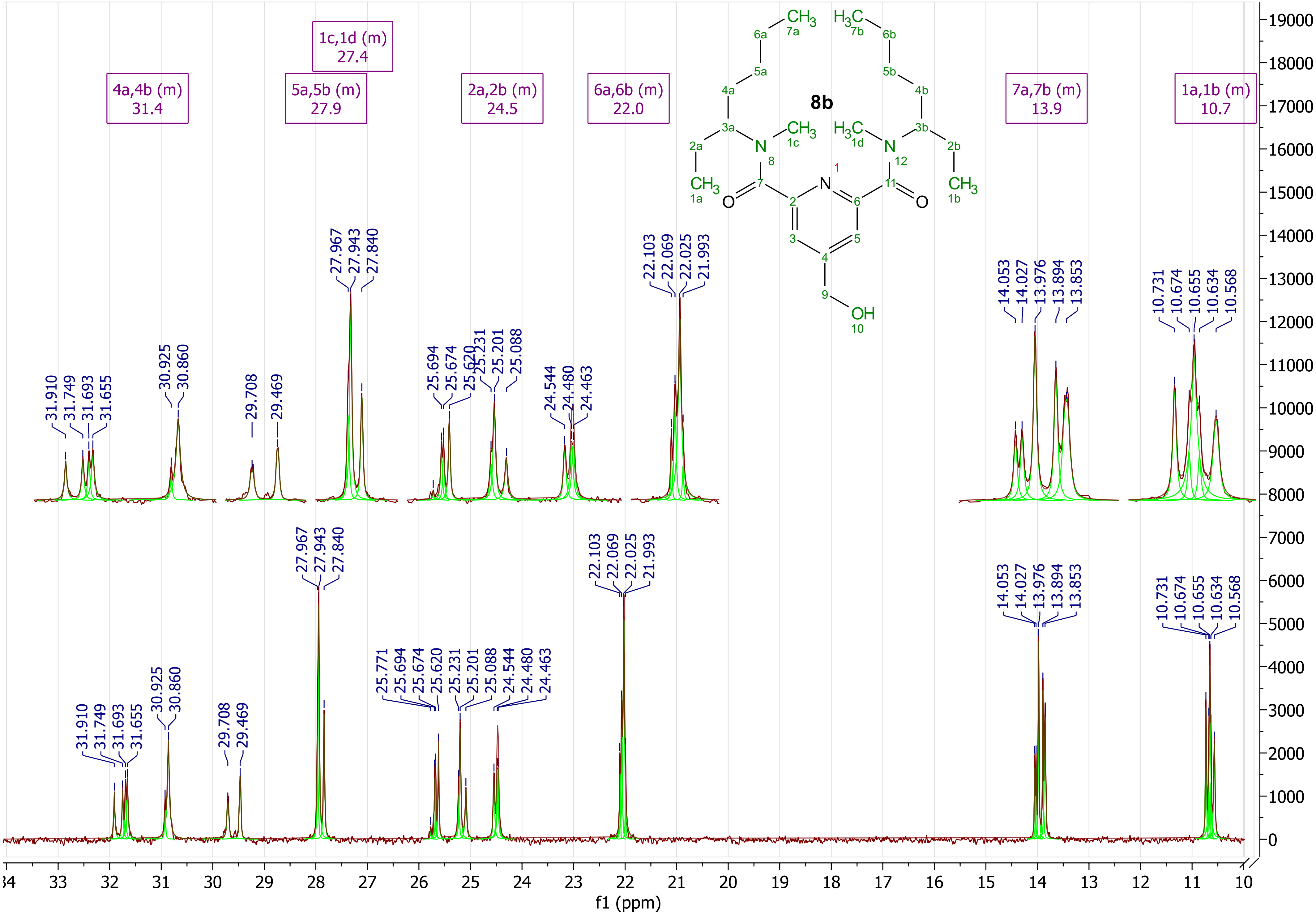

[Back ↑]

^{1}H–^{13}C HSQC

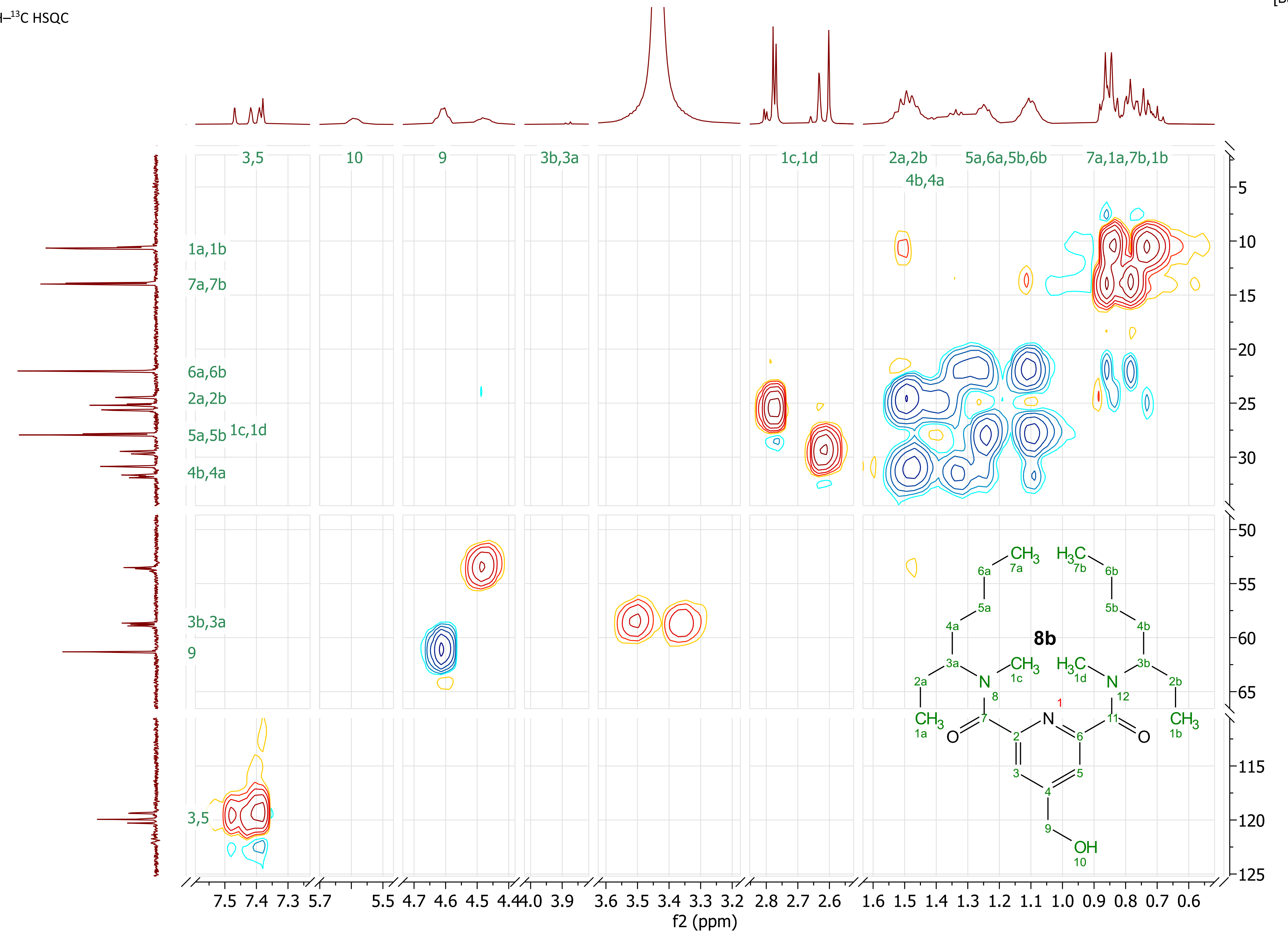

[Back ↑]

^1H–^{13}C HMBC

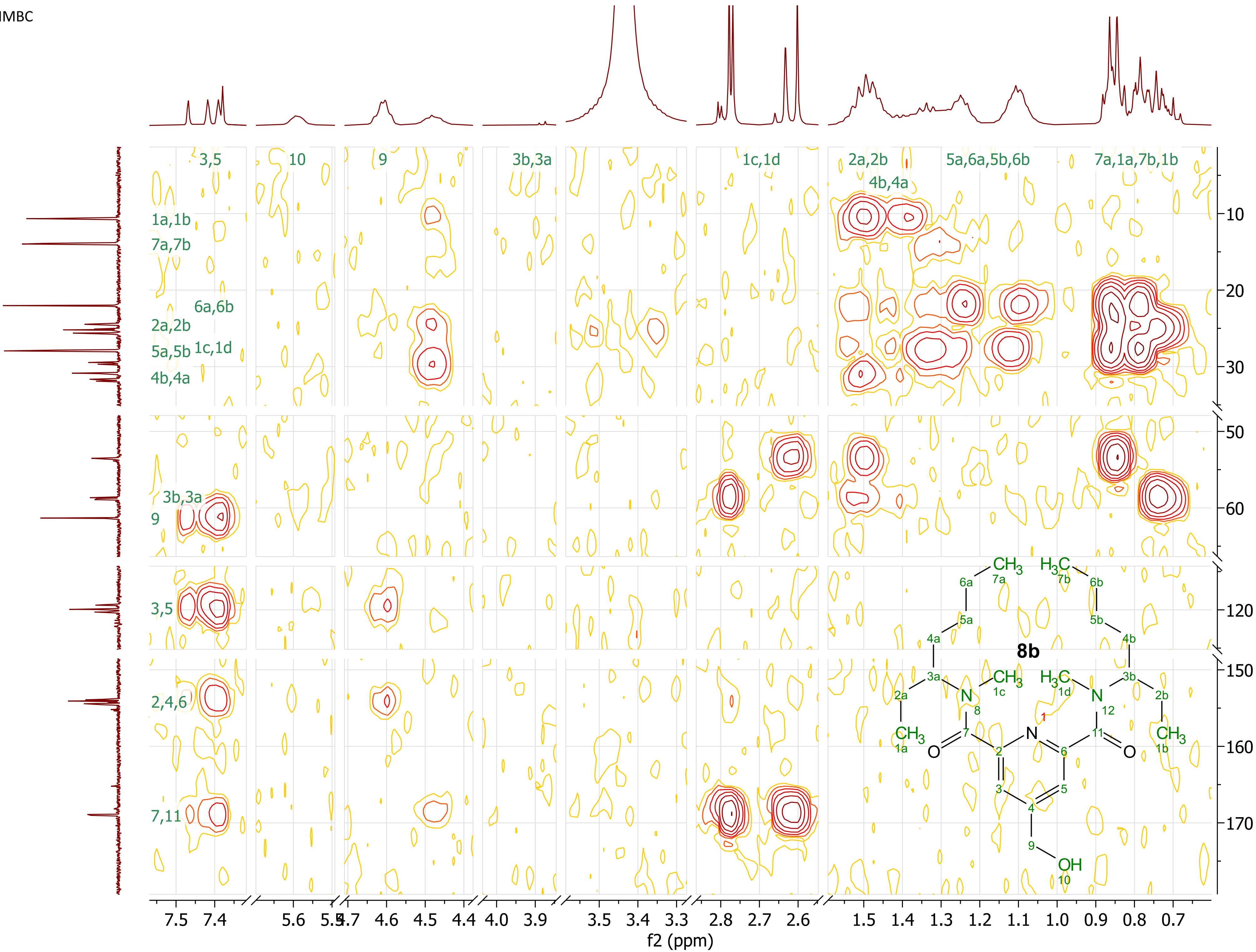

[Back ↑]

^{1}H–^{15}N HMBC

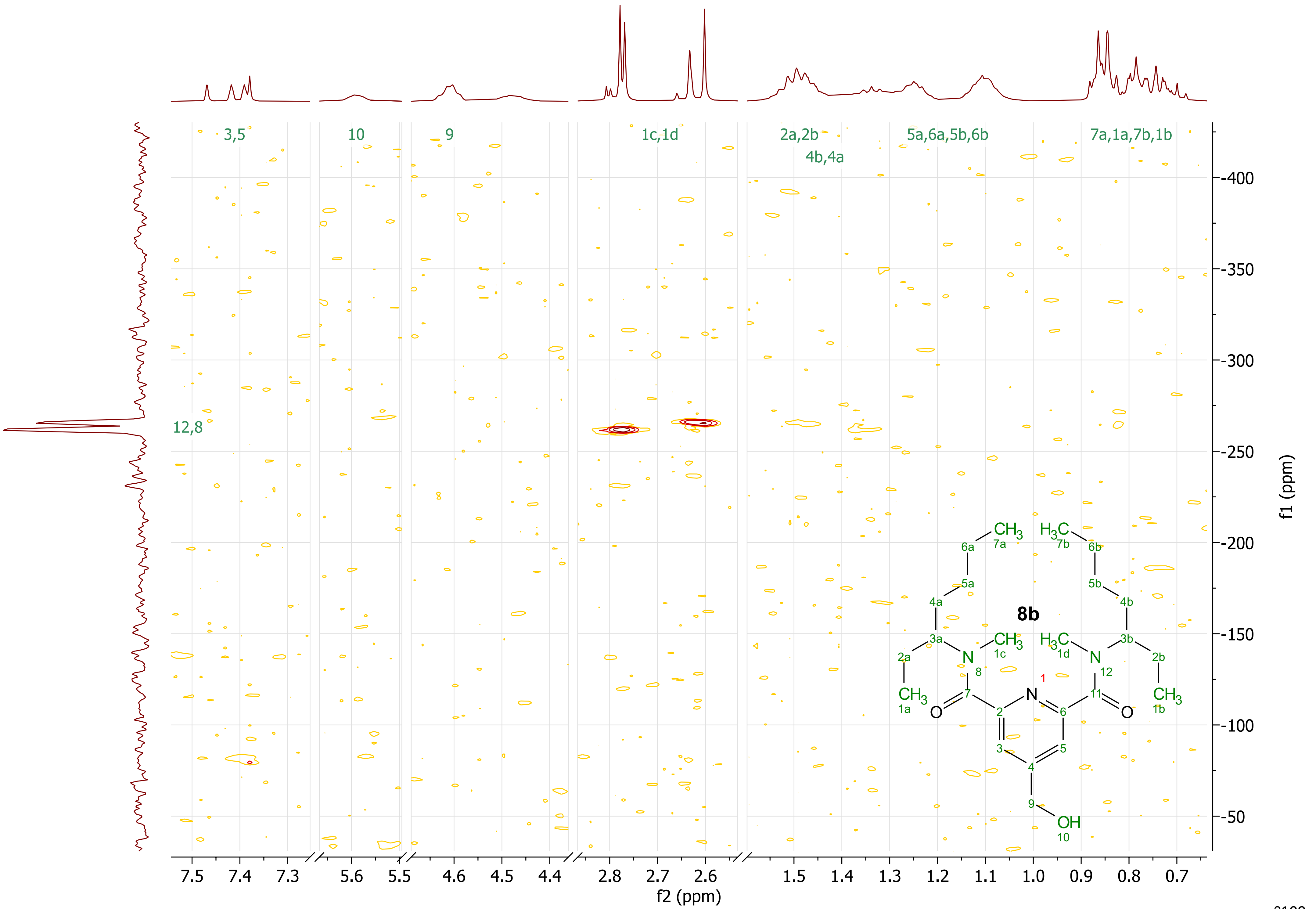

^{15}N NMR (41 MHz, DMSO-d_6) δ -255.41 – -272.53 (ms). – Projection f1

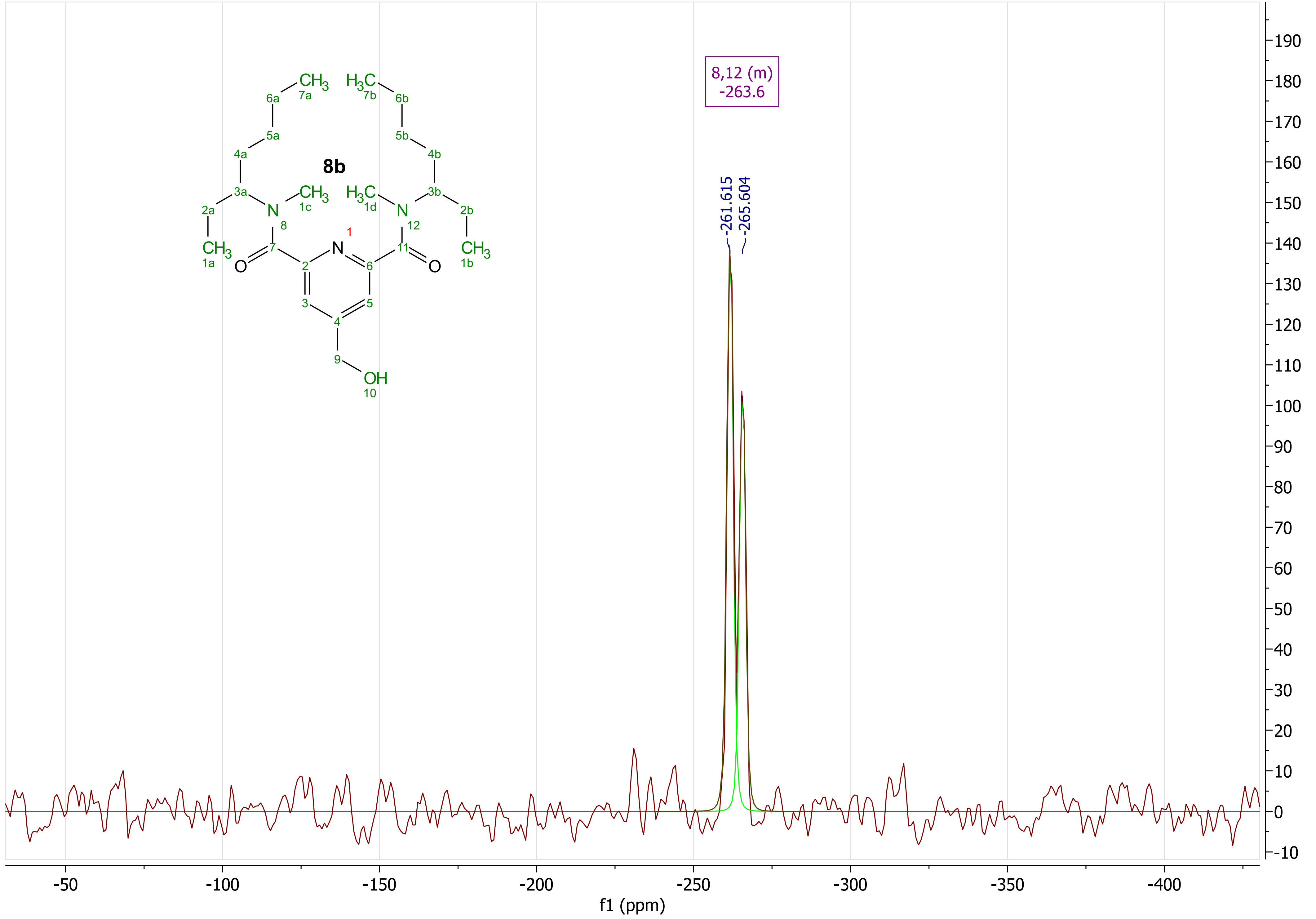

[Back ↑]

^{1}H NMR (400 MHz, DMSO-d_6, 100 °C) δ 7.55 – 7.32 (m, 2H), 5.25 (br s, 1H), 4.62 (s, 2H), 4.55 – 3.38 (mm, 2H), 2.85 – 2.64 (mm, 6H), 1.61 – 1.46 (m, 4H), 1.61 – 1.30 (m, 4H), 1.44 – 1.08 (m, 8H), 0.96 – 0.69 (m, 12H). – Mixture of conformers.

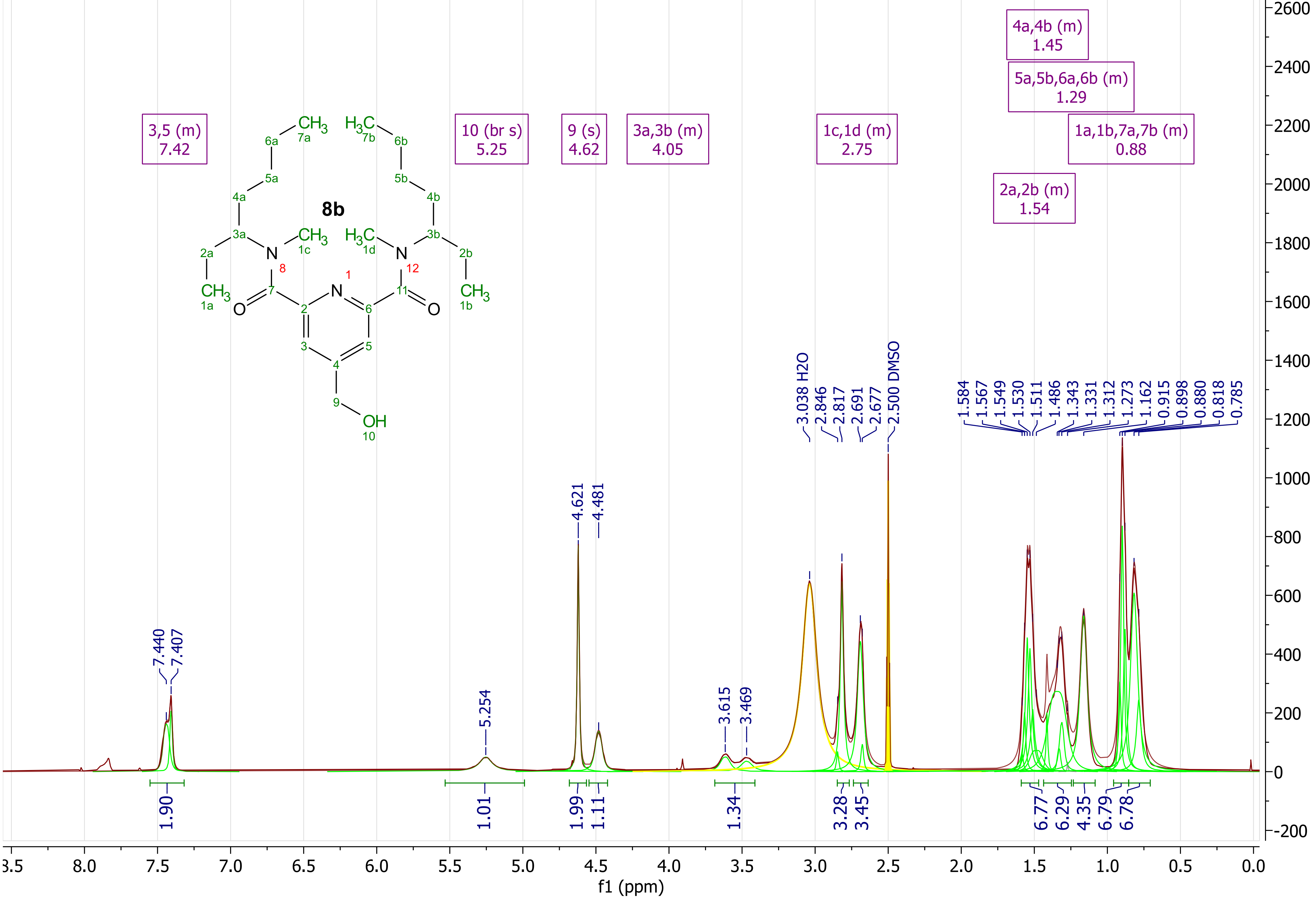

[Back ↑]

^{13}C NMR (101 MHz, DMSO-d_6, 100 °C) δ 168.4 (2C), 153.7 (3C), 120.2 – 118.8 (ms, 2C), 61.1, 58.6 – 53.4 (ms, 2C), 31.6 – 30.2 (ms, 2C), 27.4 (2C), 29.7 – 24.5 (ms, 2C), 24.0 (2C), 21.3 (2C), 13.0 (2C), 9.9 (2C). – Mixture of conformers.

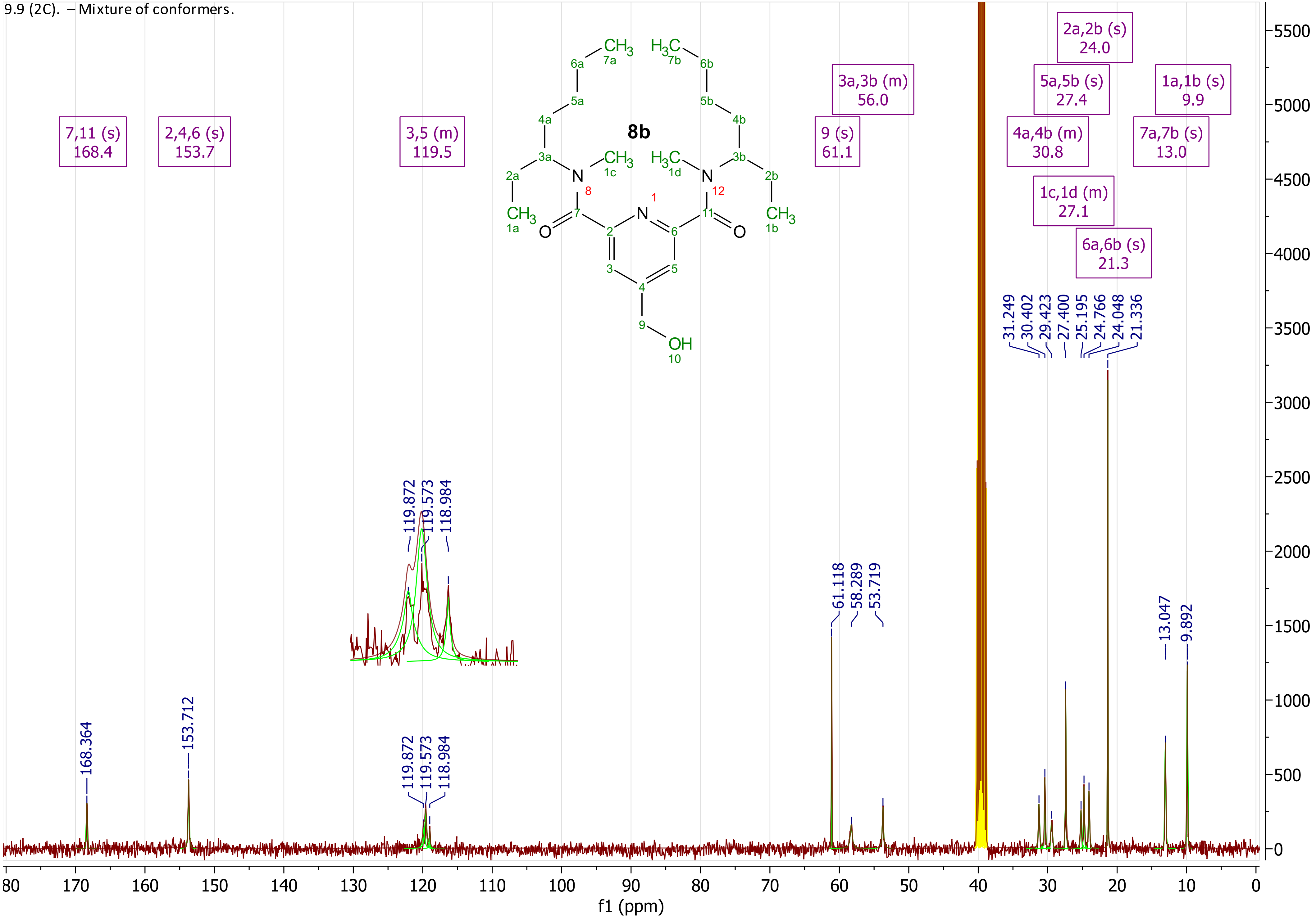

^{13}C NMR (101 MHz, DMSO-d_6) – [33 – 9 ppm]

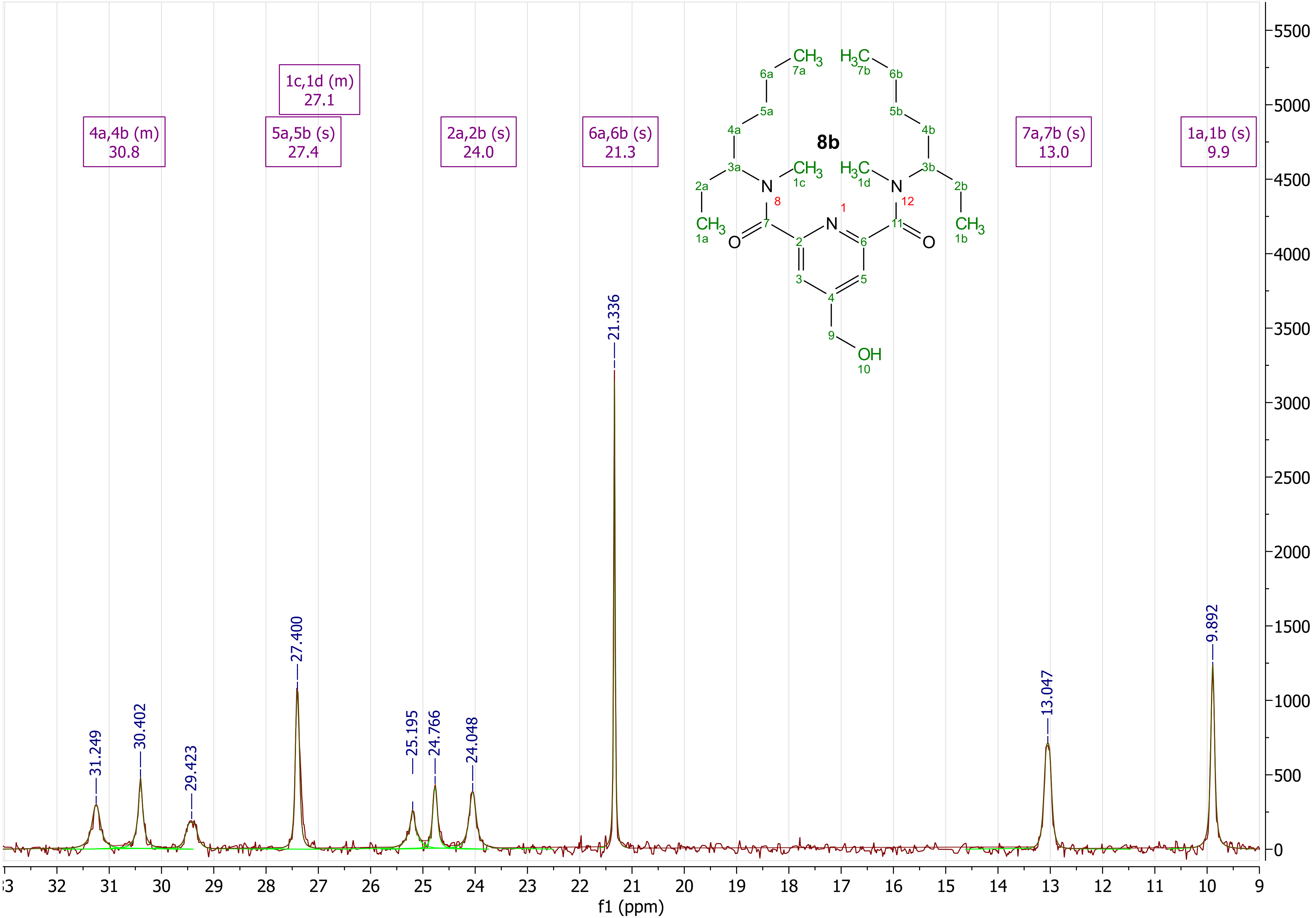

^{1}H–^{13}C HSQC

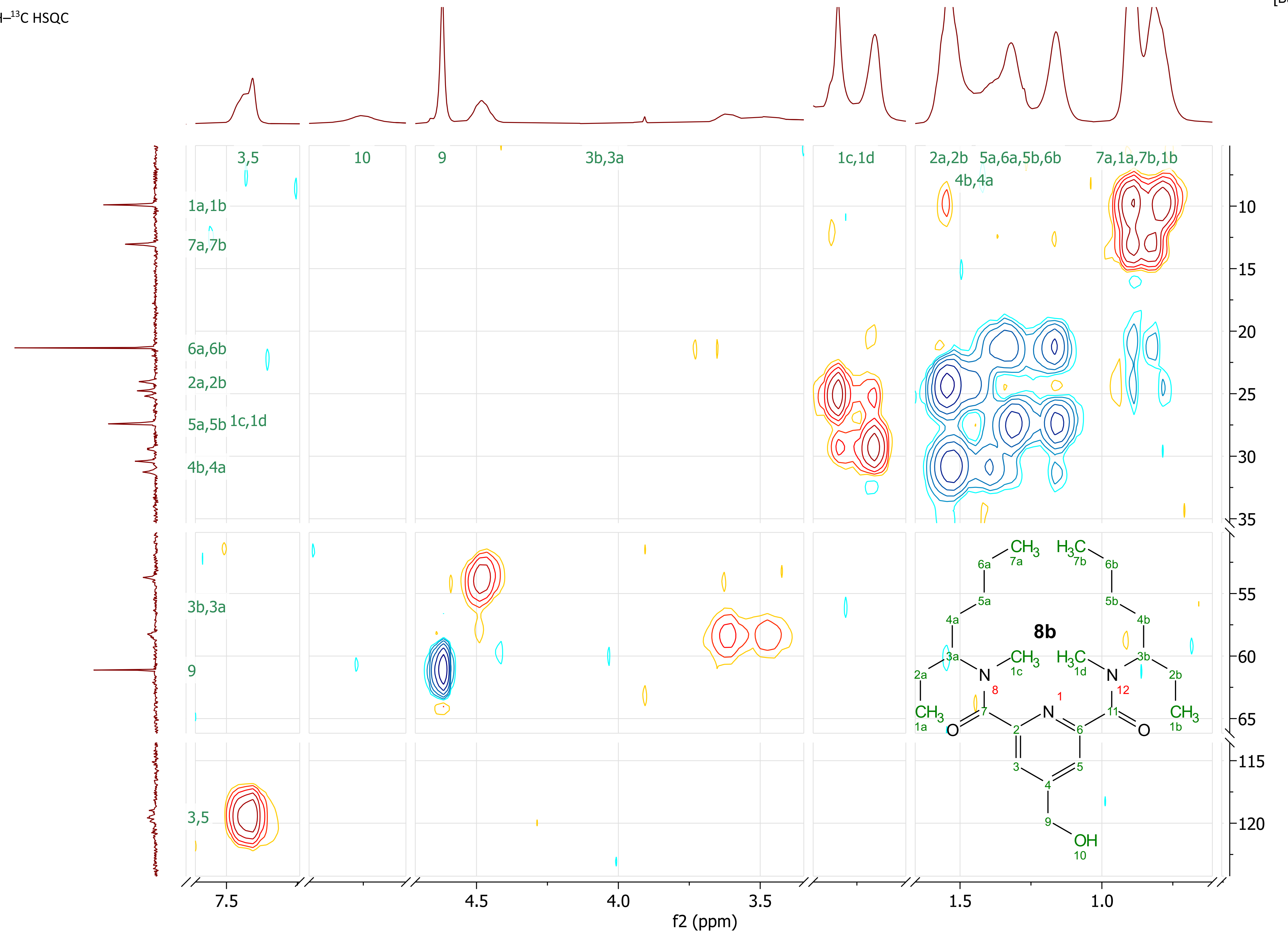
3,5
10
9
3b,3a
1c,1d
2a,2b
5a,6a,5b,6b
4b,4a
7a,1a,7b,1b
1a,1b
7a,7b
6a,6b
2a,2b
5a,5b
1c,1d
4b,4a
3b,3a
9
3,5
8b
f1 (ppm)
f2 (ppm)

[Back ↑]

^{1}H NMR (400 MHz) – Spectra comparison

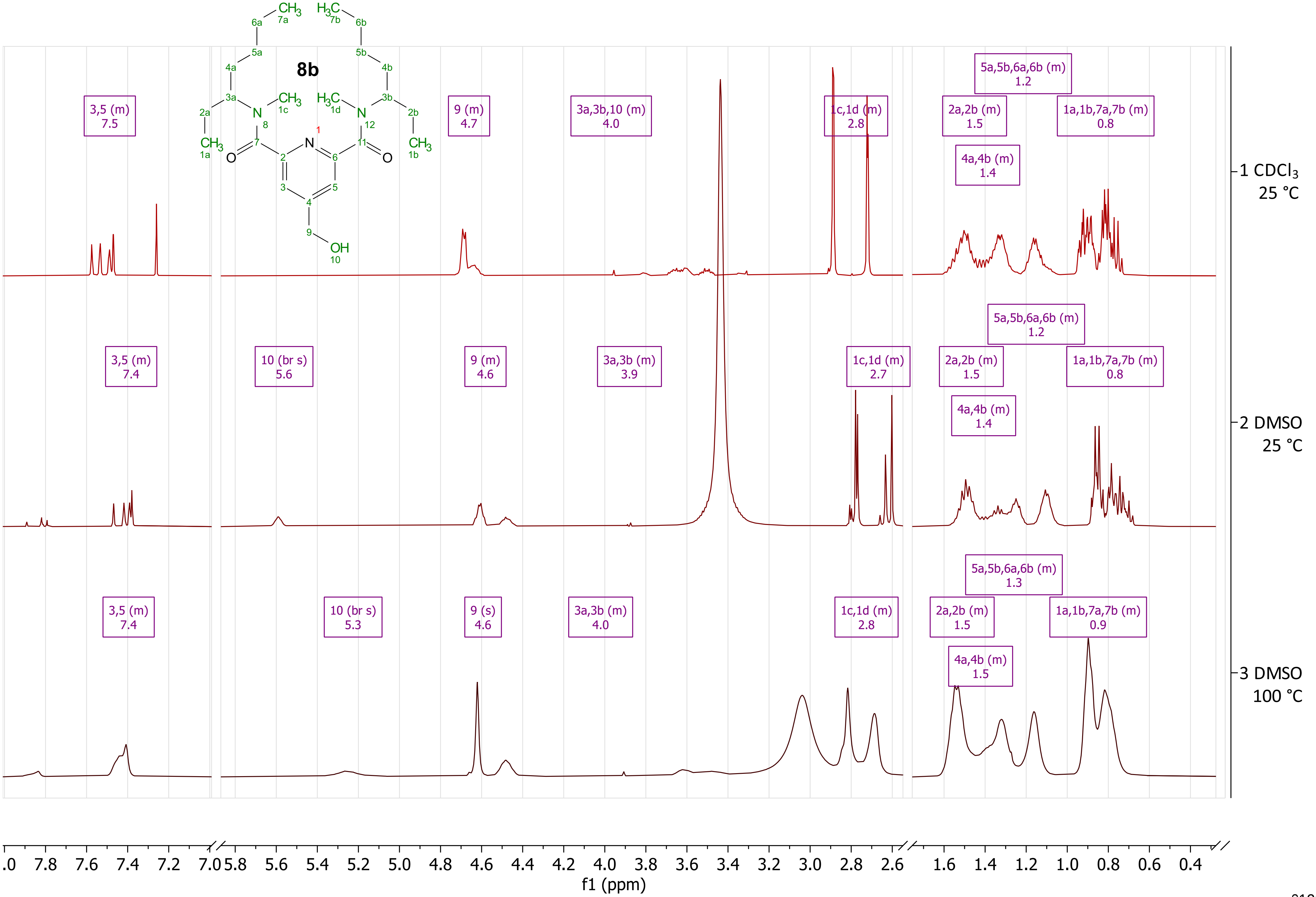

^{13}C NMR (101 MHz) – Spectra comparison

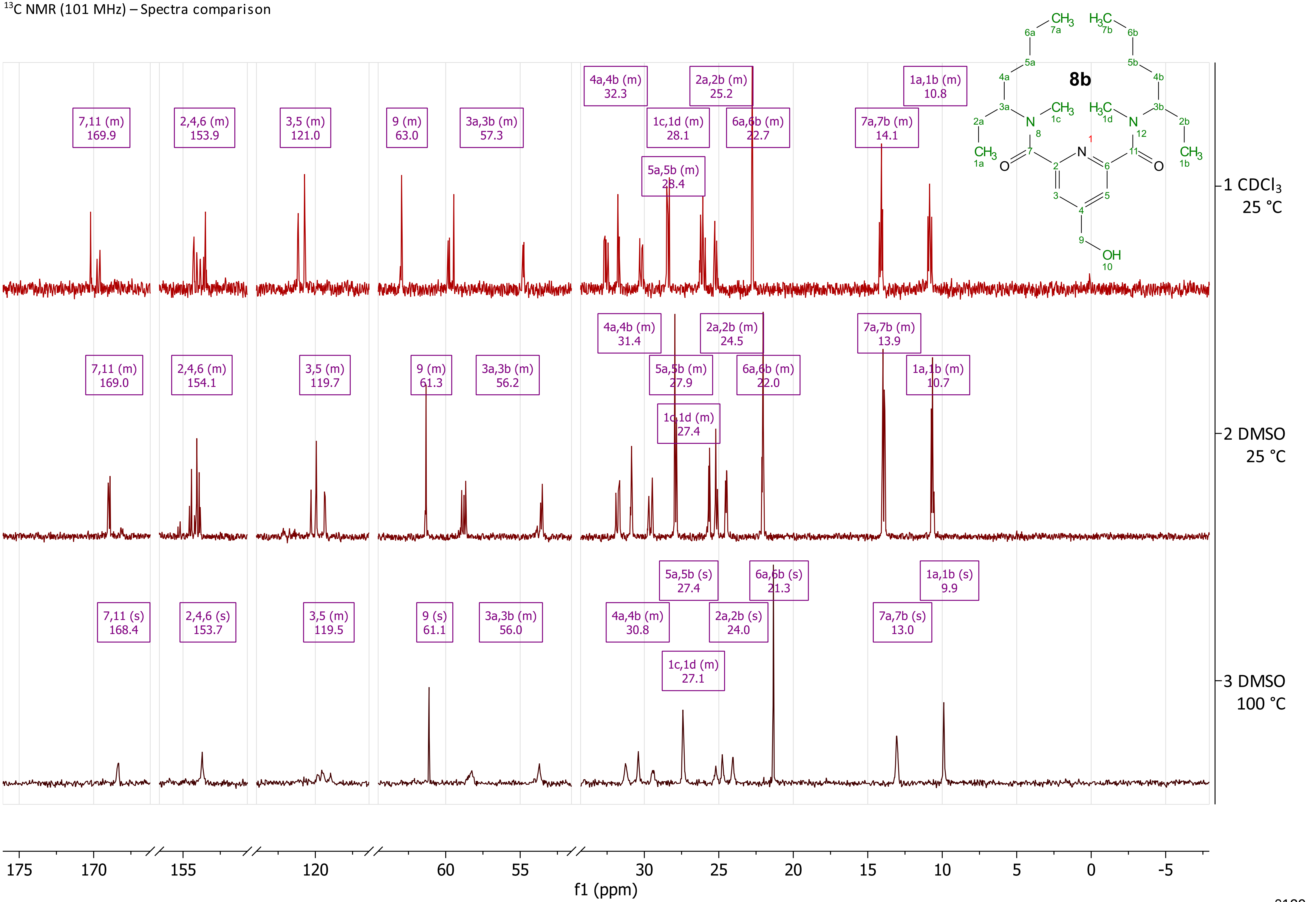

^{1}H NMR (400 MHz, $CDCl_3$) δ 2.62 – 2.51 (m, 1H), 1.46 – 1.35 (m, 2H), 1.35 – 1.17 (m, 8H), 0.87 (t, *J* = 7.5 Hz, 3H), 0.86 (t, *J* = 7.0 Hz, 3H).

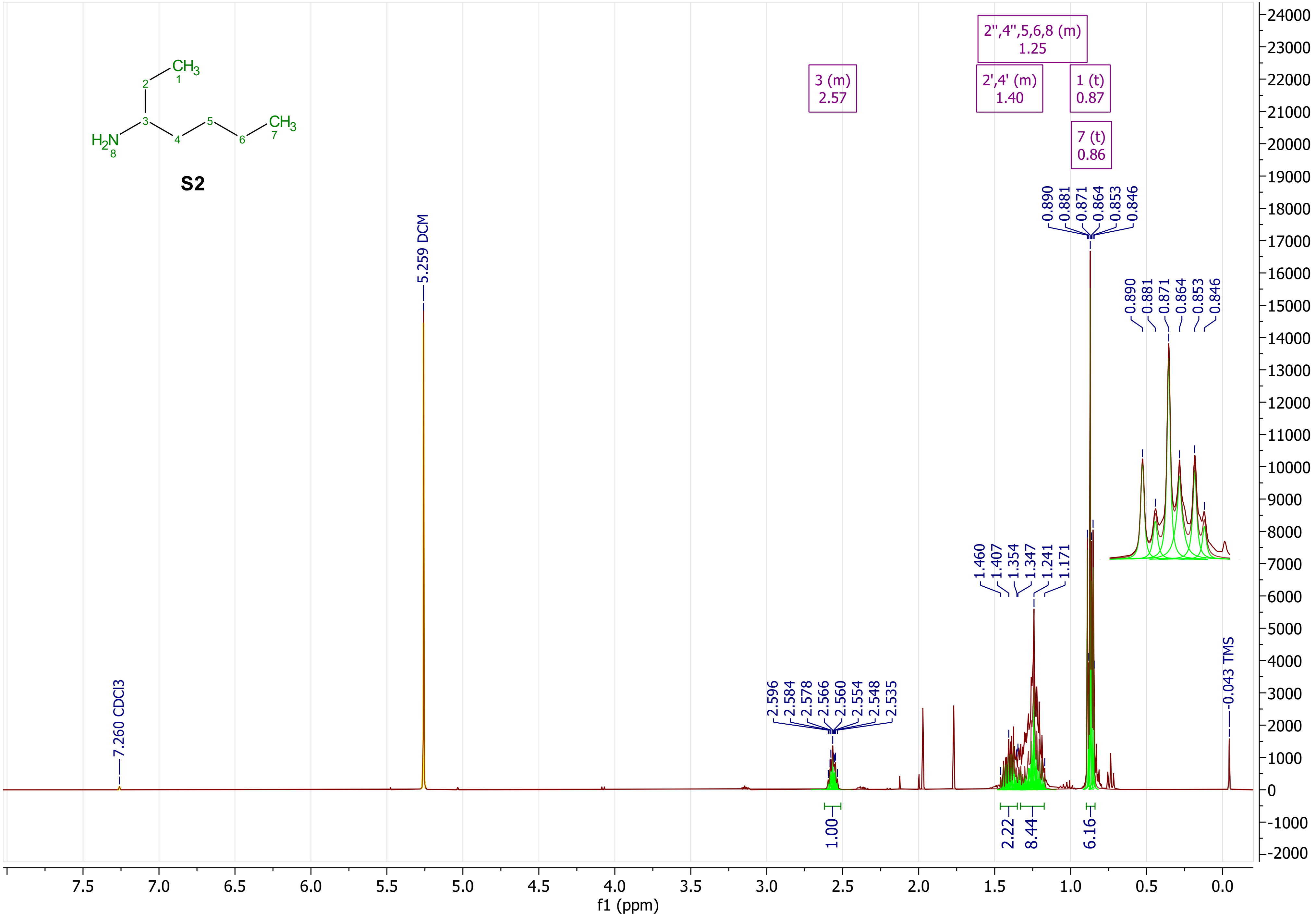

^{13}C NMR (101 MHz, $CDCl_3$) δ 52.8, 37.4, 30.8, 28.5, 23.0, 14.2, 10.5.

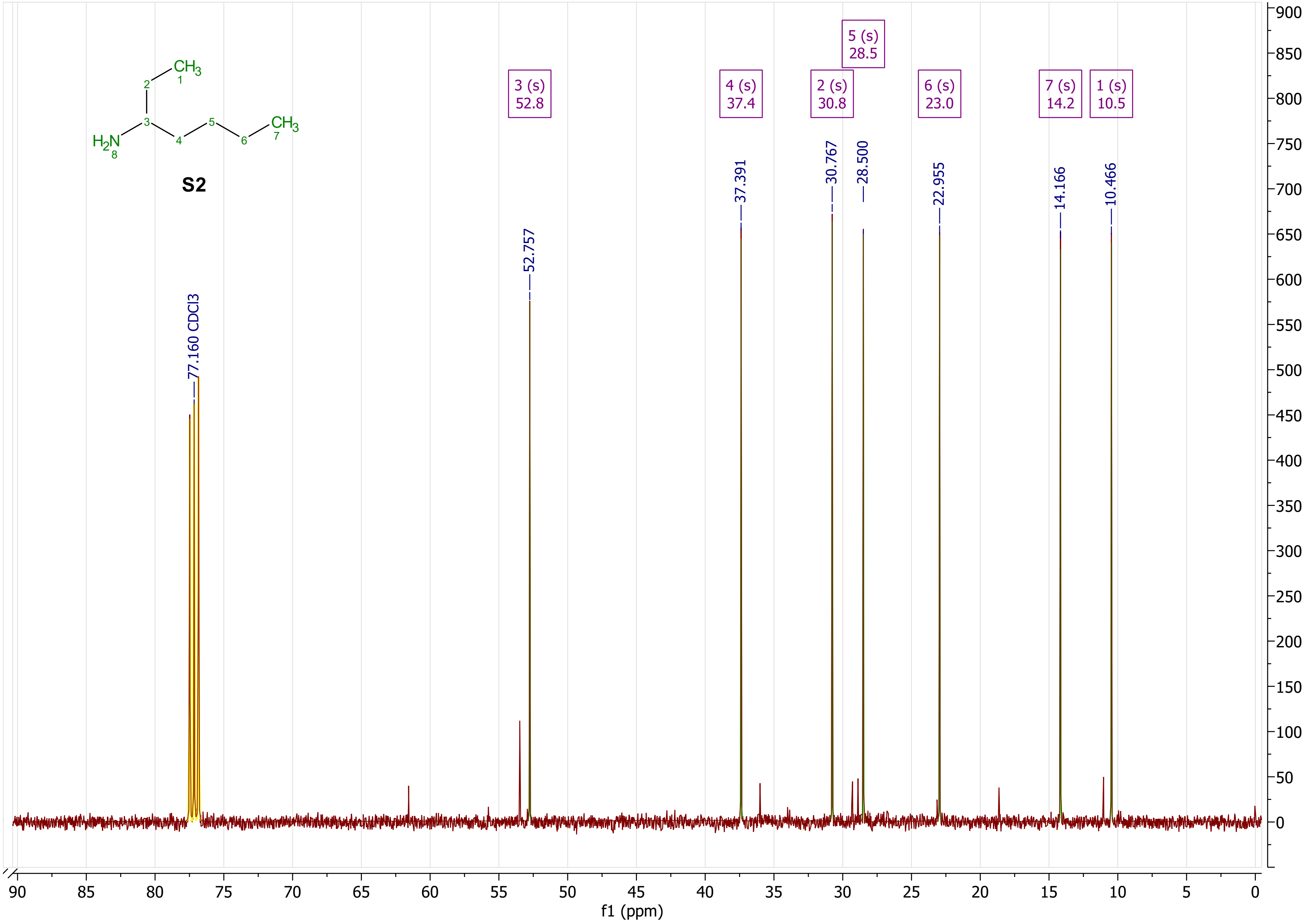

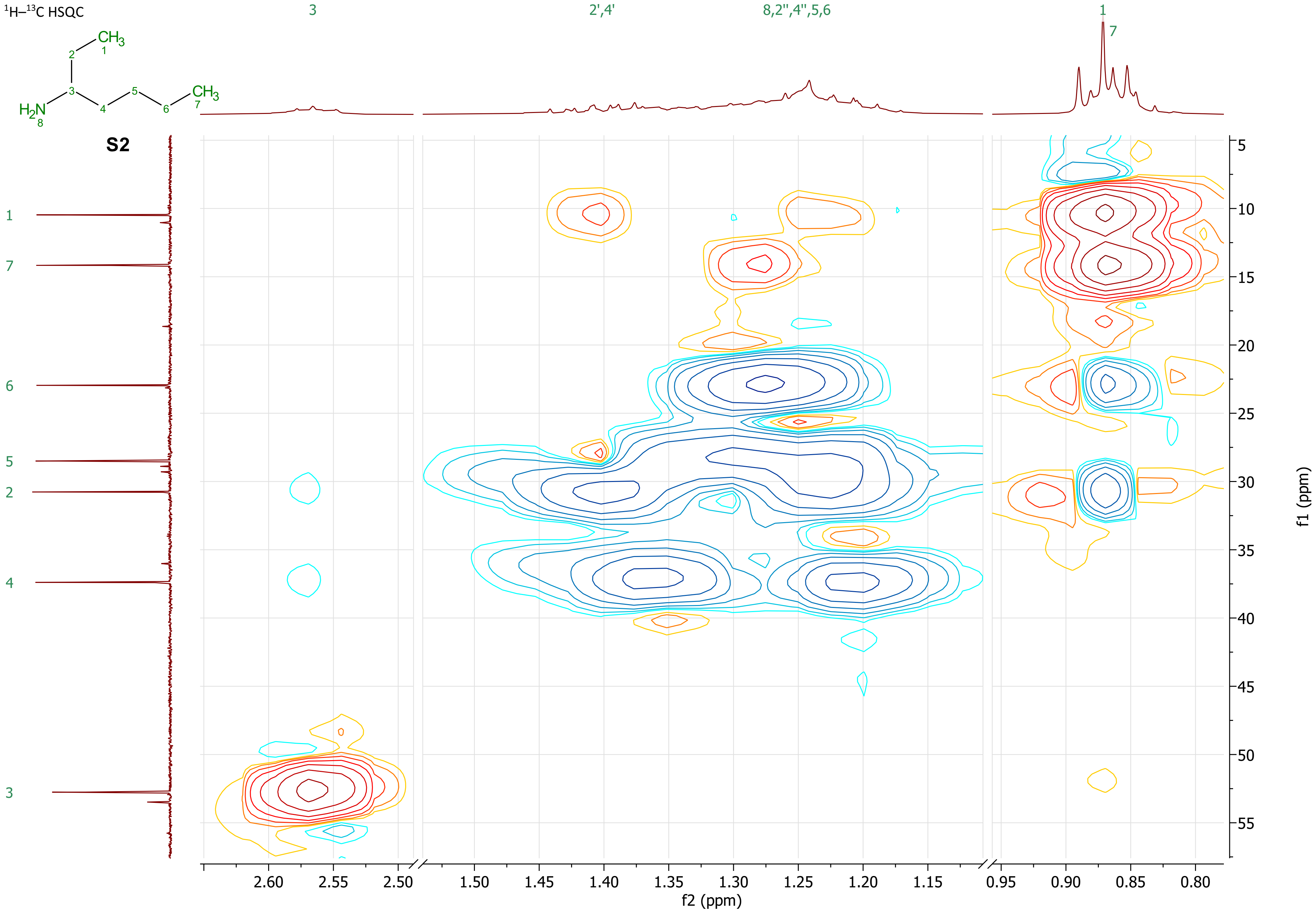

^{1}H–^{13}C HSQC
S2
3
2',4'
8,2'',4'',5,6
1
7
1
7
6
5
2
4
3
f1 (ppm)
f2 (ppm)

[Back ↑]

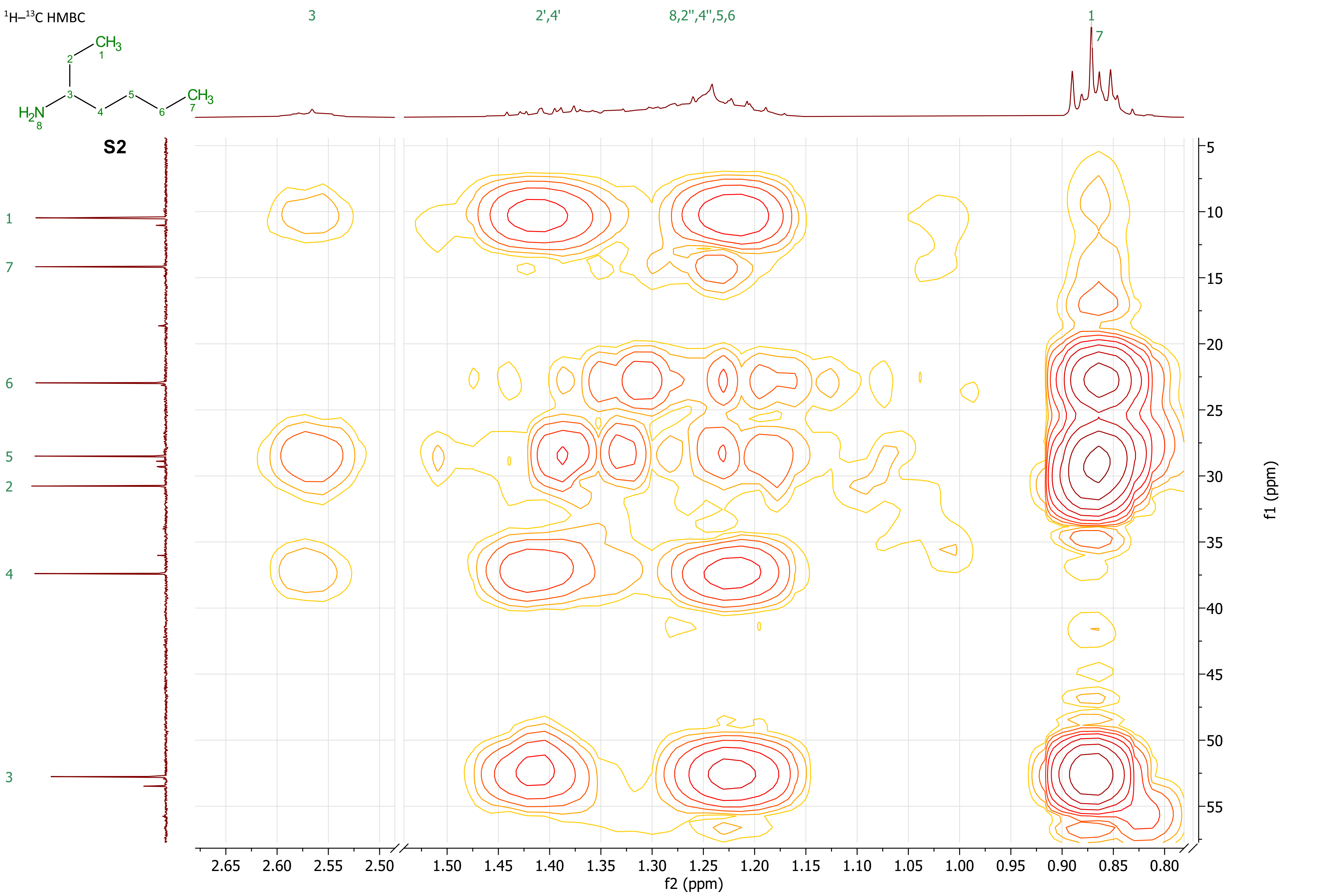

Supporting LC–MS Appendix

Total ion chromatogram (TIC) and photodiode array (PDA) signals, mass spectra (MS), and single mass analyses (SMA) are provided for all tested compounds.

Contents

Compounds		Spectra	Page
Compound	**5a**	TIC/PDA	S136
		MS	S137
		SMA	S138
Compound	**5b**	TIC/PDA	S139
		MS	S140
		SMA	S141
Compound	**7a**	TIC/PDA	S142
		MS	S143
		SMA	S144
Compound	**7b**	TIC/PDA	S145
		MS	S146
		SMA	S147

Compound	**7c**	TIC/PDA	S148
		MS	S149
		SMA	S150
Compound	**8a**	TIC/PDA	S151
		MS	S152
		SMA	S153
Compound	**8b**	TIC/PDA	S154
		MS	S155
		SMA	S156

5a

[Back ↑]

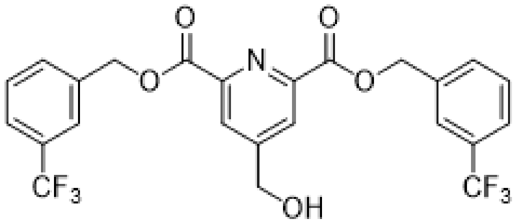

Chemical Formula: $C_{24}H_{17}F_6NO_5$
Exact Mass: 513.1011
Molecular Weight: 513.3924

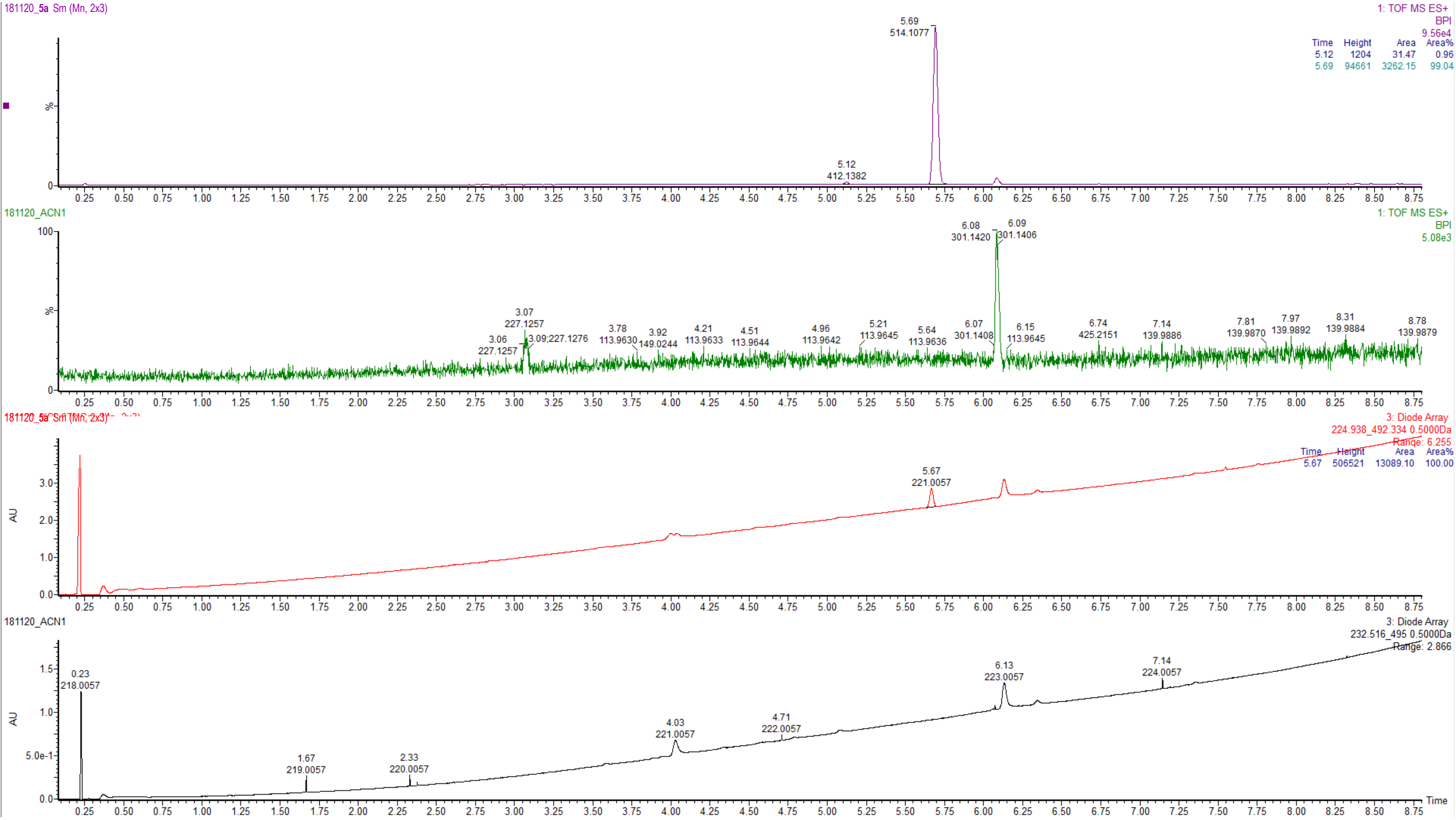

[Back ↑]

5a

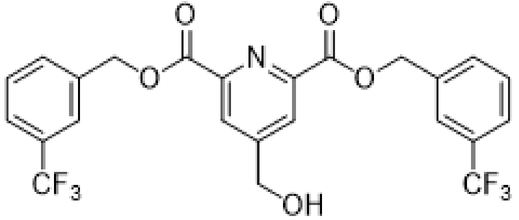

Chemical Formula: $C_{24}H_{17}F_6NO_5$
Exact Mass: 513.1011
Molecular Weight: 513.3924

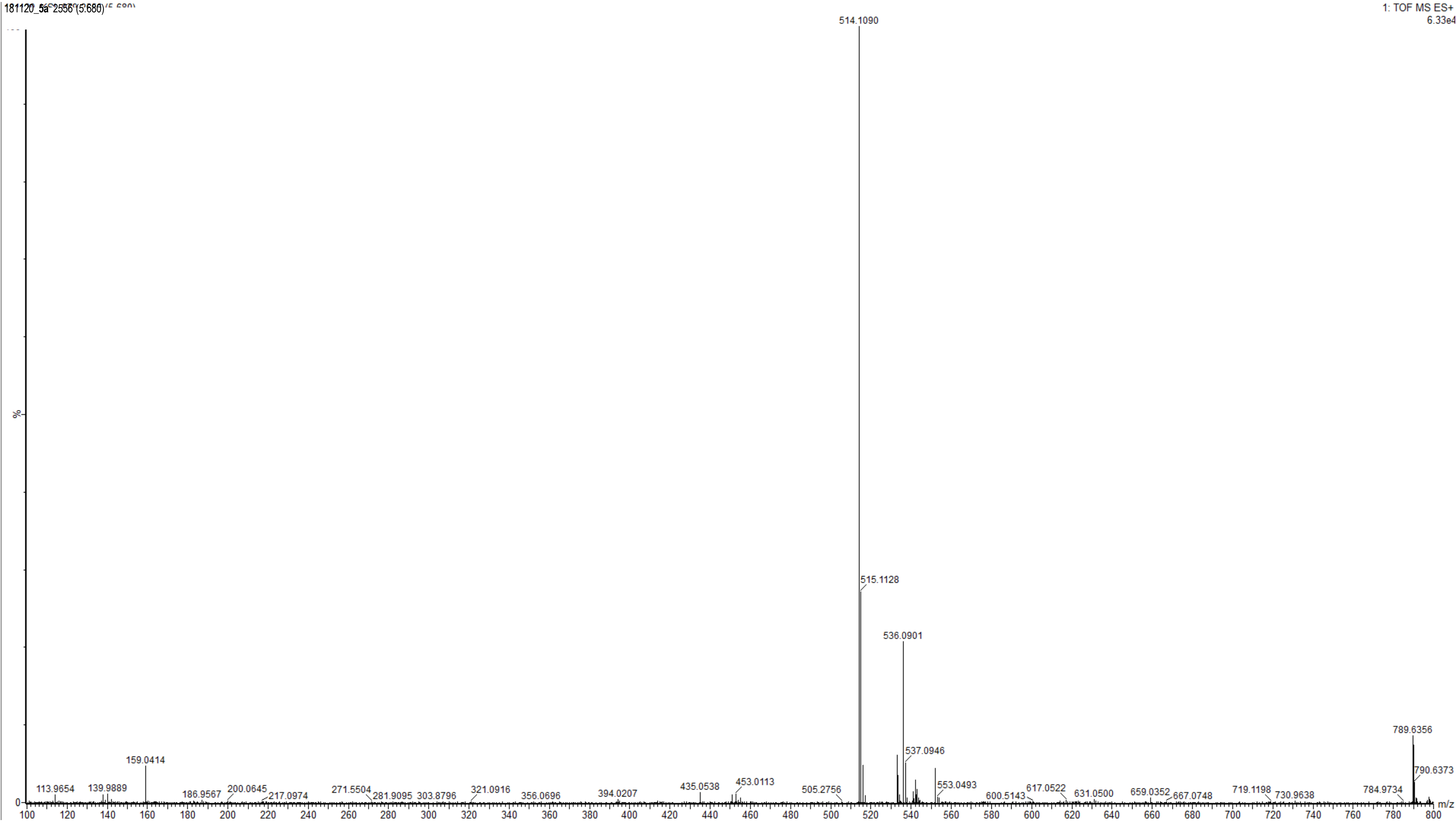

5a

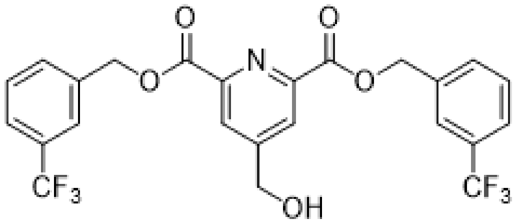

Chemical Formula: $C_{24}H_{17}F_6NO_5$
Exact Mass: 513.1011
Molecular Weight: 513.3924

Single Mass Analysis
Tolerance = 50.0 mDa / DBE: min = -1.5, max = 50.0
Element prediction: Off
Number of isotope peaks used for i-FIT = 5
Monoisotopic Mass, Even Electron Ions
6428 formula(e) evaluated with 564 results within limits (all results (up to 1000) for each mass)
Elements Used:

Mass	Calc. Mass	mDa	PPM	DBE	Formula	i-FIT	i-FIT Norm	Fit Conf %	C	H	N	O	F	Na
514.1090	514.1090	0.0	0.0	13.5	C24 H20 N O7 F3 Na	127.8	2.898	5.51	24	20	1	7	3	1
	514.1091	-0.1	-0.2	24.5	C32 H17 N O5 F	137.0	12.159	0.00	32	17	1	5	1	
	514.1089	0.1	0.2	13.5	C24 H18 N O5 F6	128.9	4.059	1.73	24	18	1	5	6	
	514.1085	0.5	1.0	8.5	C18 H22 N3 O12 F Na	135.1	10.240	0.00	18	22	3	12	1	1
	514.1085	0.5	1.0	8.5	C18 H20 N3 O10 F4	135.3	10.396	0.00	18	20	3	10	4	
	514.1096	-0.6	-1.2	4.5	C15 H21 N3 O11 F5	137.9	13.005	0.00	15	21	3	11	5	
	514.1097	-0.7	-1.4	4.5	C15 H23 N3 O13 F2 Na	137.7	12.833	0.00	15	23	3	13	2	1
	514.1098	-0.8	-1.6	15.5	C23 H20 N3 O11	131.5	6.629	0.13	23	20	3	11		

C24 H18 N O5 F6

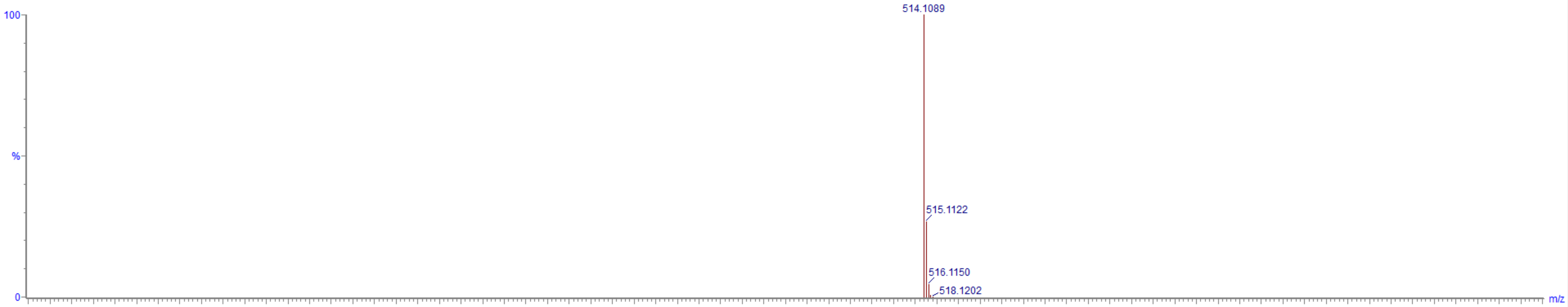

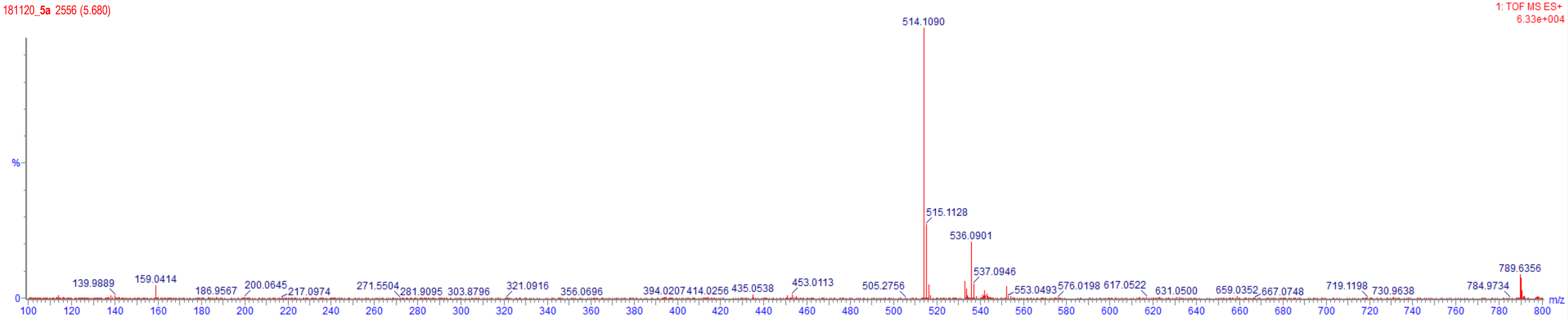

[Back ↑]

5b

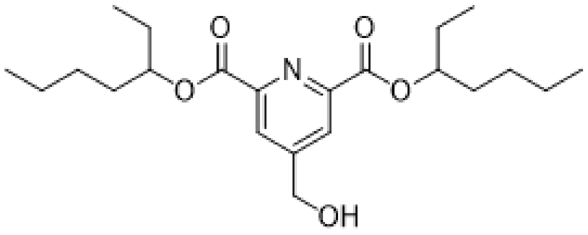

Chemical Formula: $C_{22}H_{35}NO_5$
Exact Mass: 393.2515
Molecular Weight: 393.5240

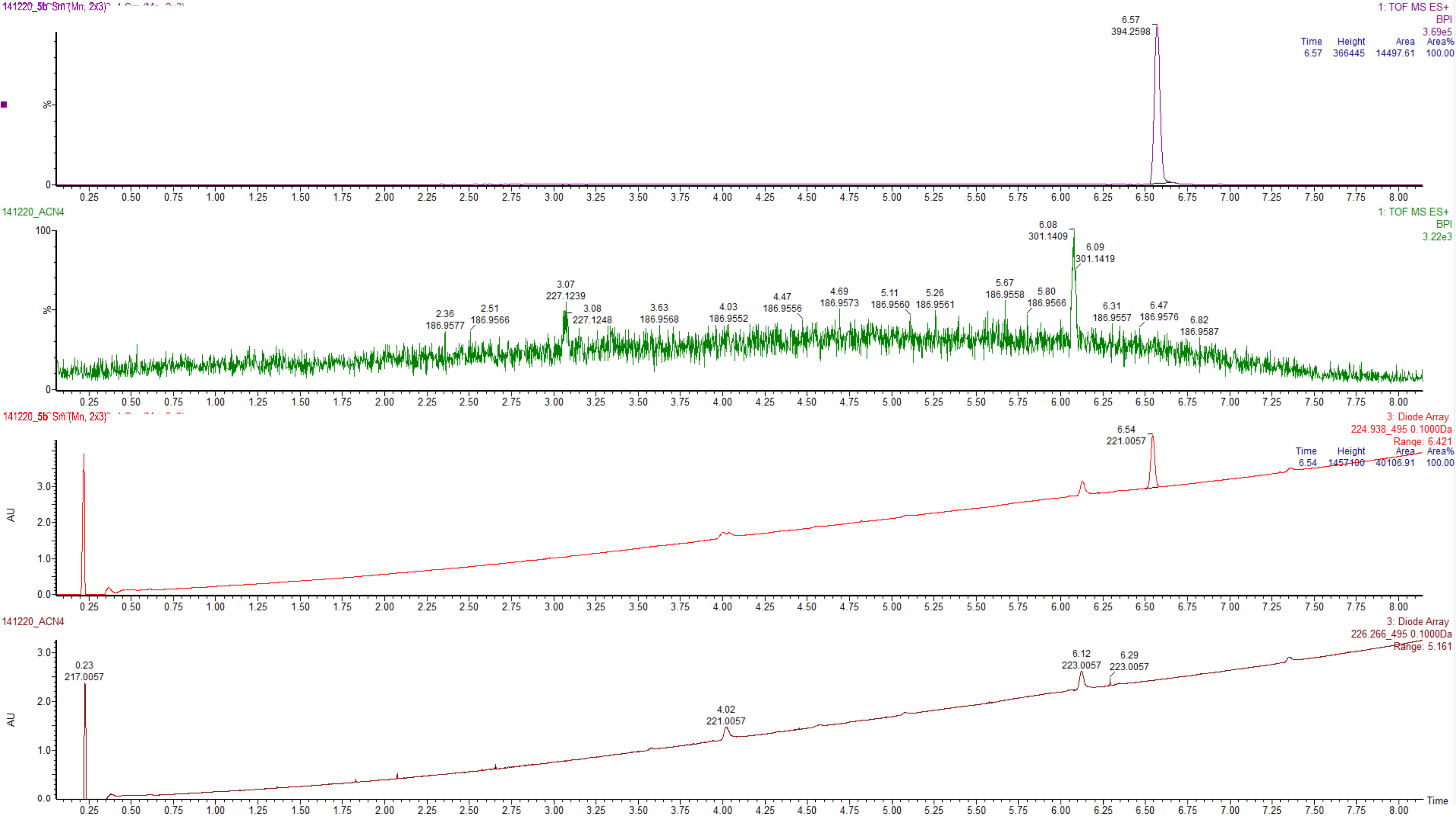

[Back ↑]

5b

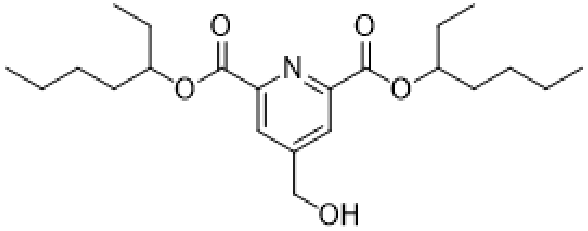

Chemical Formula: $C_{22}H_{35}NO_5$
Exact Mass: 393.2515
Molecular Weight: 393.5240

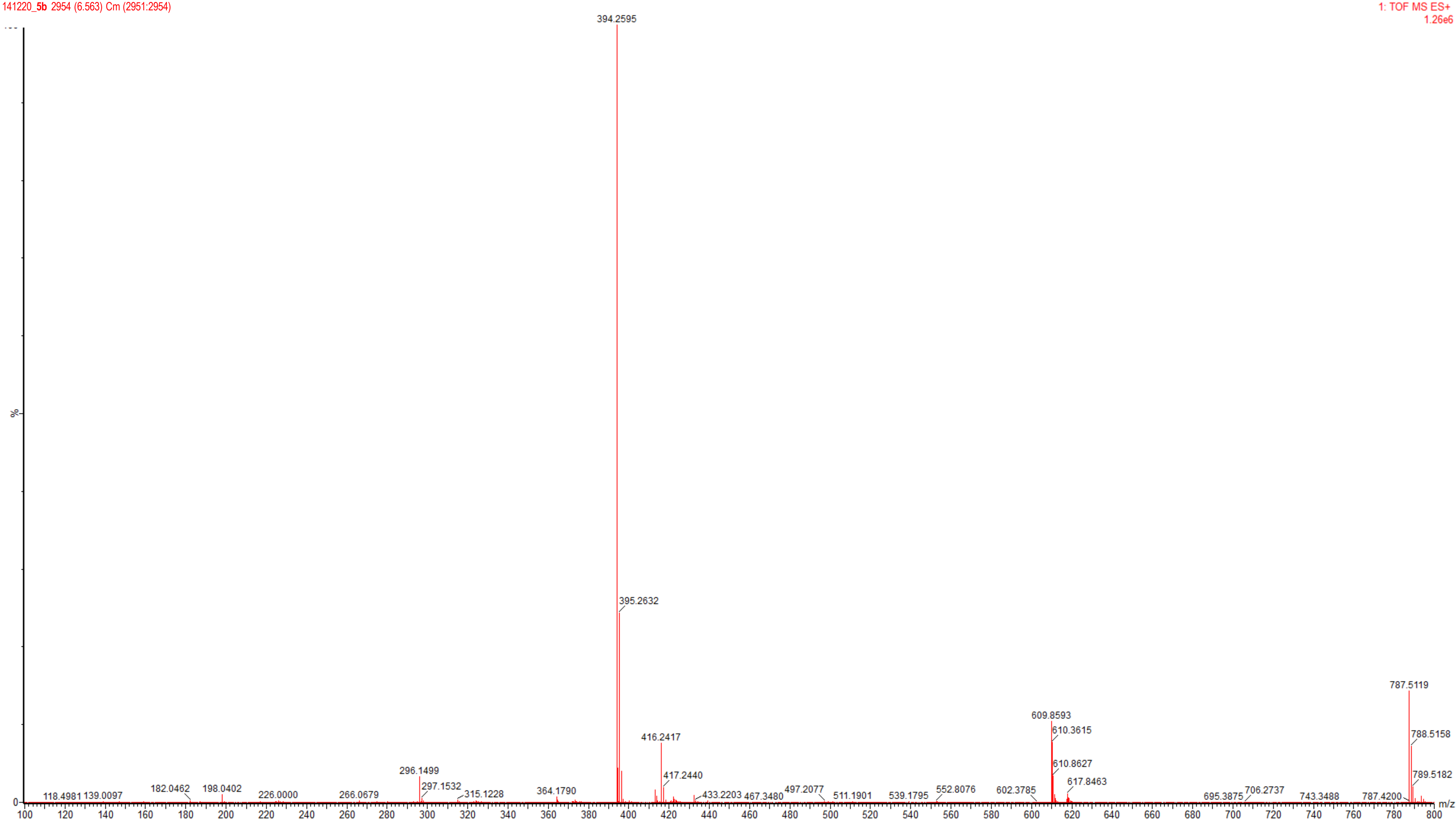

[Back ↑]

5b

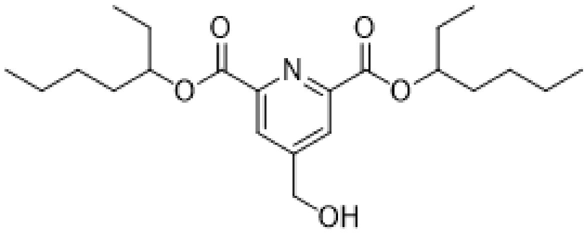

Chemical Formula: $C_{22}H_{35}NO_5$
Exact Mass: 393.2515
Molecular Weight: 393.5240

Single Mass Analysis
Tolerance = 50.0 mDa / DBE: min = -1.5, max = 50.0
Element prediction: Off
Number of isotope peaks used for i-FIT = 5
Monoisotopic Mass, Even Electron Ions
4748 formula(e) evaluated with 263 results within limits (all results (up to 1000) for each mass)
Elements Used:

Mass	Calc. Mass	mDa	PPM	DBE	Formula	i-FIT	i-FIT Norm	Fit Conf %	C	H	N	O	F
394.2595	394.2594	0.1	0.3	3.5	C18 H32 N5 F4	758.9	13.970	0.00	18	32	5		4
	394.2593	0.2	0.5	5.5	C22 H36 N O5	745.0	0.095	90.89	22	36	1	5	
	394.2599	-0.4	-1.0	-1.5	C7 H32 N13 O6	772.4	27.479	0.00	7	32	13	6	
	394.2591	0.4	1.0	7.5	C16 H29 N11 F	765.2	20.320	0.00	16	29	11		1
	394.2589	0.6	1.5	-1.5	C12 H34 N7 O5 F2	767.5	22.571	0.00	12	34	7	5	2
	394.2603	-0.8	-2.0	3.5	C13 H30 N11 O F2	768.5	23.581	0.00	13	30	11	1	2
	394.2605	-1.0	-2.5	1.5	C19 H37 N O6 F	755.5	10.636	0.00	19	37	1	6	1
	394.2605	-1.0	-2.5	-0.5	C15 H33 N5 O F5	763.5	18.647	0.00	15	33	5	1	5

C22 H36 N O5

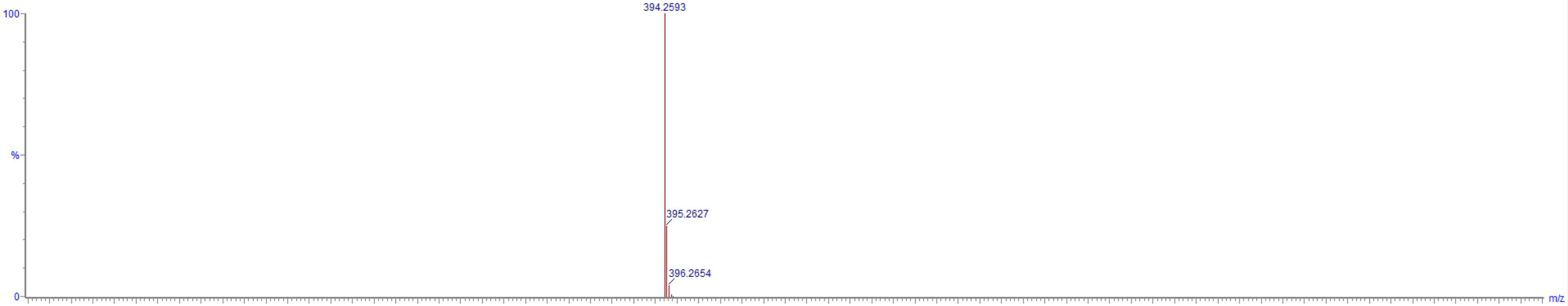

141220_5b 2954 (6.563) Cm (2951:2954)
1: TOF MS ES+
1.26e+006

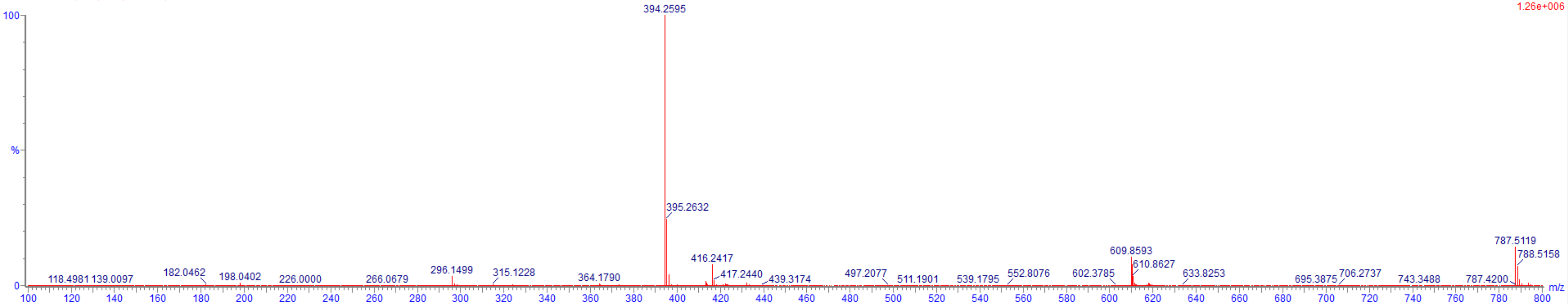

7a

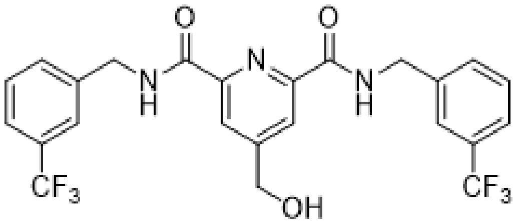

Chemical Formula: $C_{24}H_{19}F_6N_3O_3$
Exact Mass: 511.1331
Molecular Weight: 511.4244

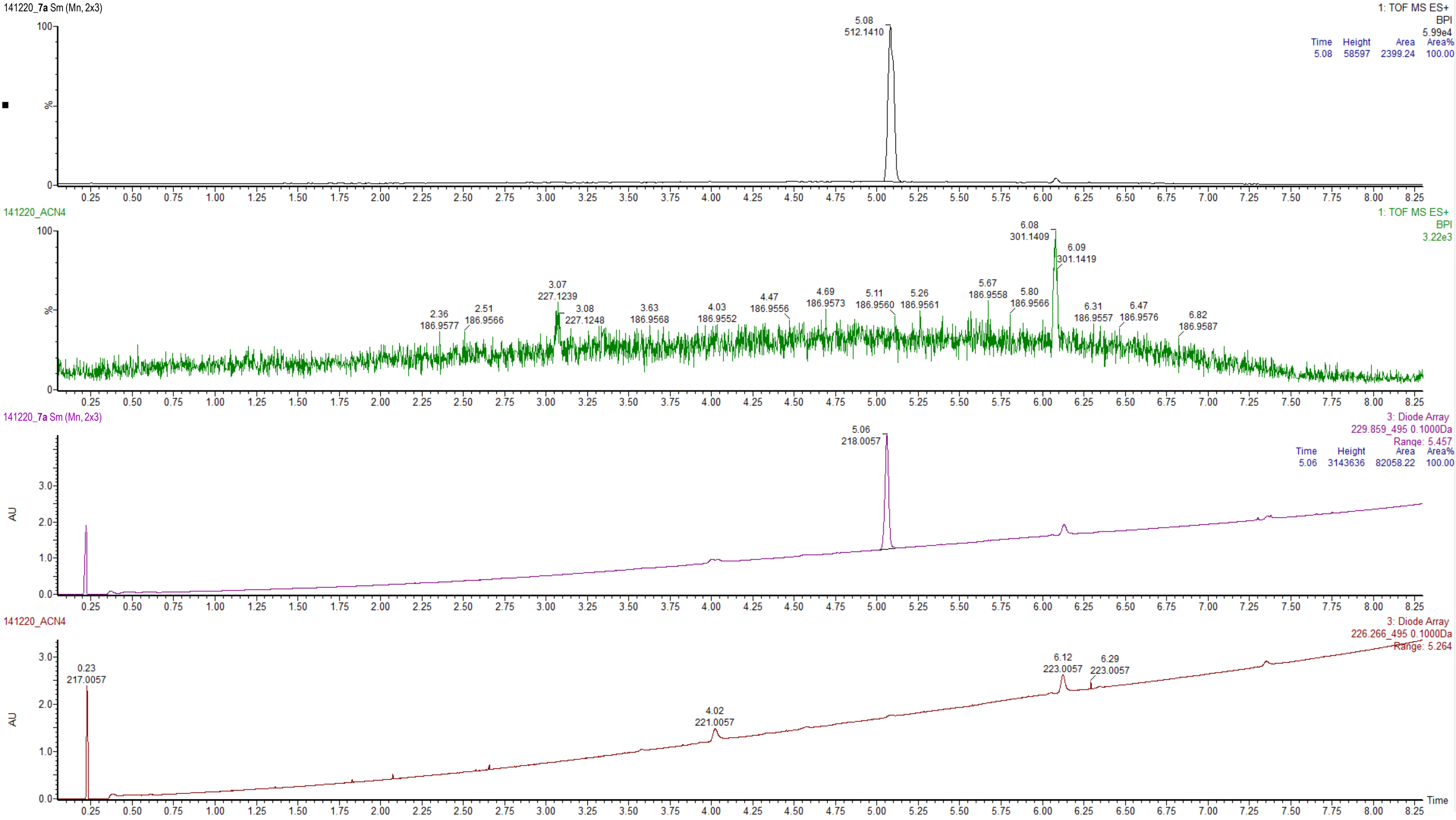

[Back ↑]

7a

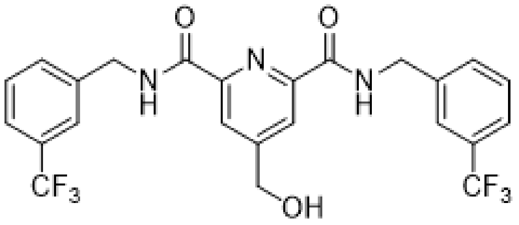

Chemical Formula: $C_{24}H_{19}F_6N_3O_3$
Exact Mass: 511.1331
Molecular Weight: 511.4244

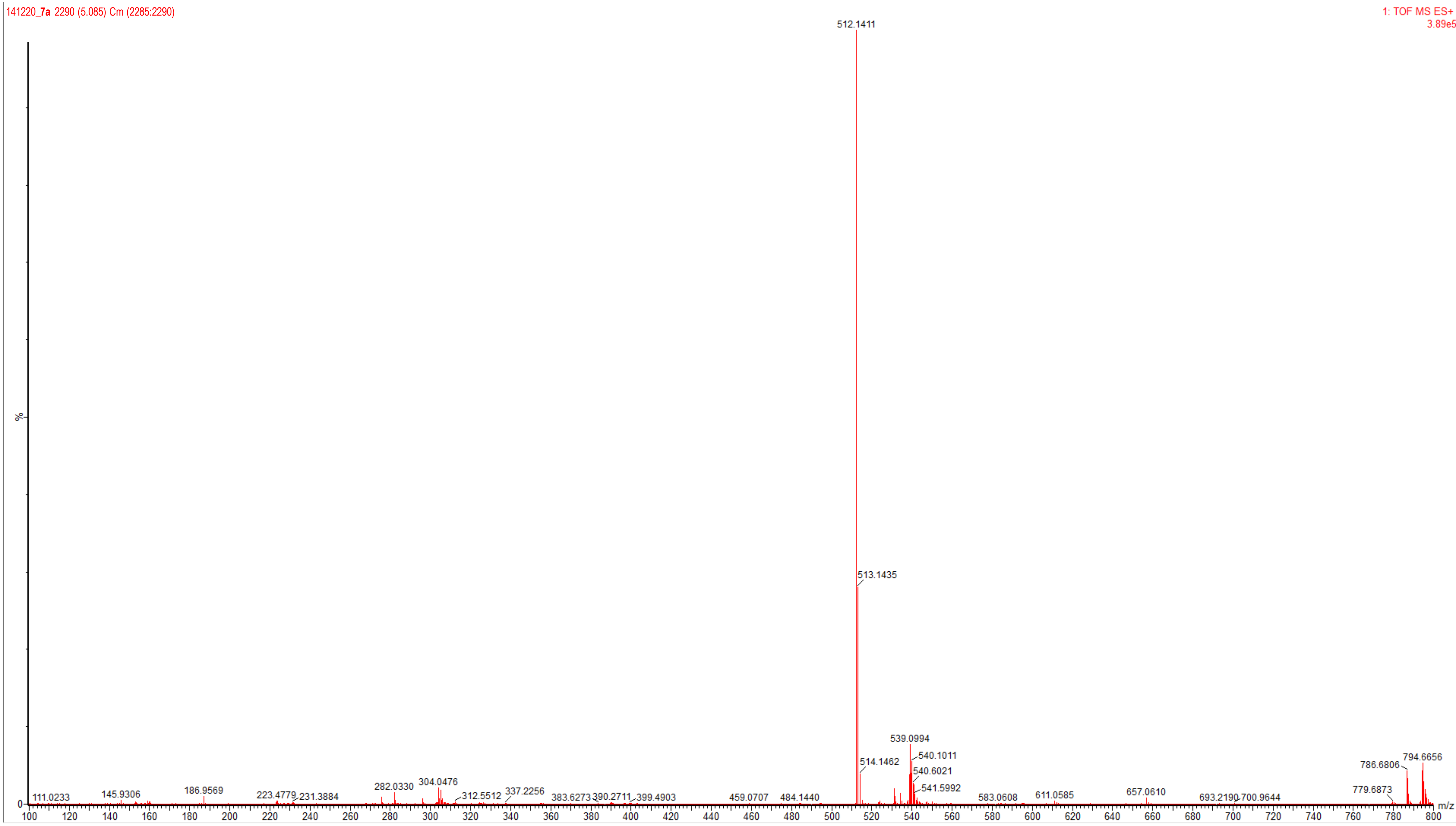

[Back ↑]

7a

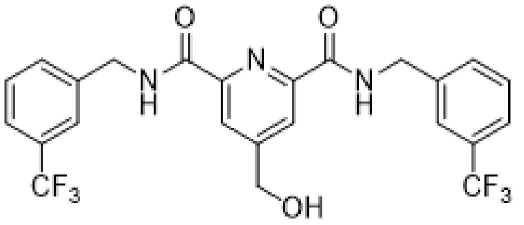

Chemical Formula: $C_{24}H_{19}F_6N_3O_3$
Exact Mass: 511.1331
Molecular Weight: 511.4244

Single Mass Analysis
Tolerance = 50.0 mDa / DBE: min = -1.5, max = 50.0
Element prediction: Off
Number of isotope peaks used for i-FIT = 5
Monoisotopic Mass, Even Electron Ions
7984 formula(e) evaluated with 932 results within limits (all results (up to 1000) for each mass)
Elements Used:

Mass	Calc. Mass	mDa	PPM	DBE	Formula	i-FIT	i-FIT Norm	Fit Conf %	C	H	N	O	F
512.1411	512.1412	-0.1	-0.2	-0.5	C9 H25 N7 O14 F3	597.1	17.785	0.00	9	25	7	14	3
	512.1410	0.1	0.2	24.5	C32 H19 N3 O3 F	597.6	18.293	0.00	32	19	3	3	1
	512.1409	0.2	0.4	13.5	C24 H20 N3 O3 F6	583.8	4.475	1.14	24	20	3	3	6
	512.1409	0.2	0.4	3.5	C7 H22 N13 O14	599.0	19.685	0.00	7	22	13	14	
	512.1414	-0.3	-0.6	6.5	C9 H16 N15 O4 F6	599.2	19.874	0.00	9	16	15	4	6
	512.1414	-0.3	-0.6	8.5	C13 H20 N11 O9 F2	594.4	15.092	0.00	13	20	11	9	2
	512.1415	-0.4	-0.8	17.5	C17 H15 N15 O4 F	589.4	10.052	0.00	17	15	15	4	1
	512.1416	-0.5	-1.0	6.5	C19 H27 N O14 F	594.0	14.712	0.00	19	27	1	14	1

C24 H20 N3 O3 F6

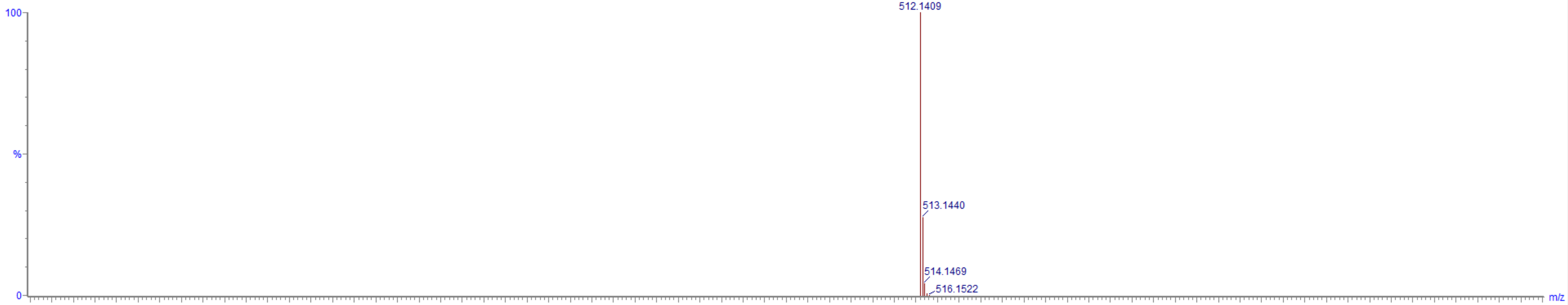

141220_7a 2290 (5.085) Cm (2285:2290)
1: TOF MS ES+
3.89e+005

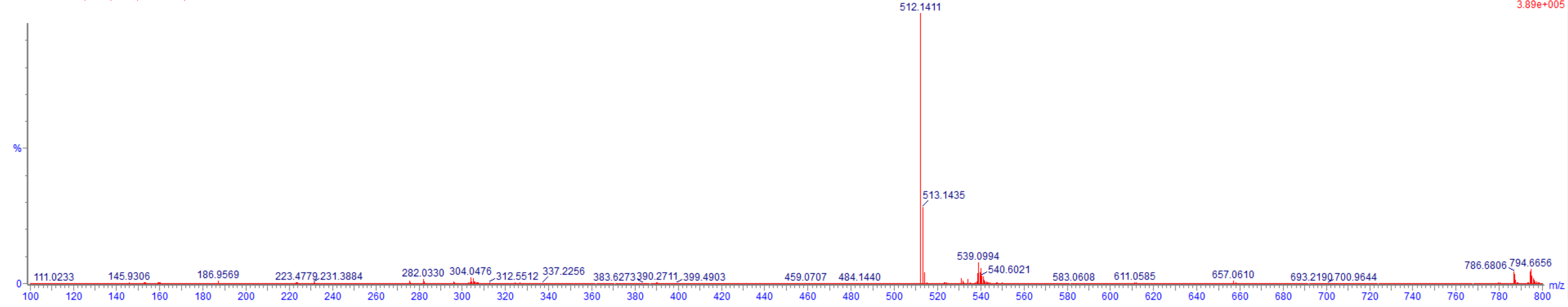

7b

[Back ↑]

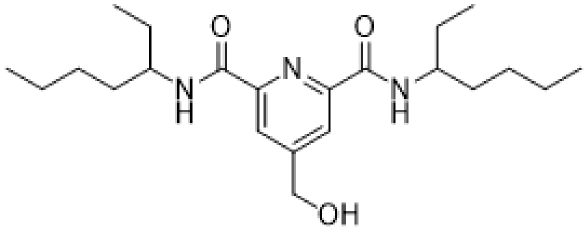

Chemical Formula: $C_{22}H_{37}N_3O_3$
Exact Mass: 391.2835
Molecular Weight: 391.5560

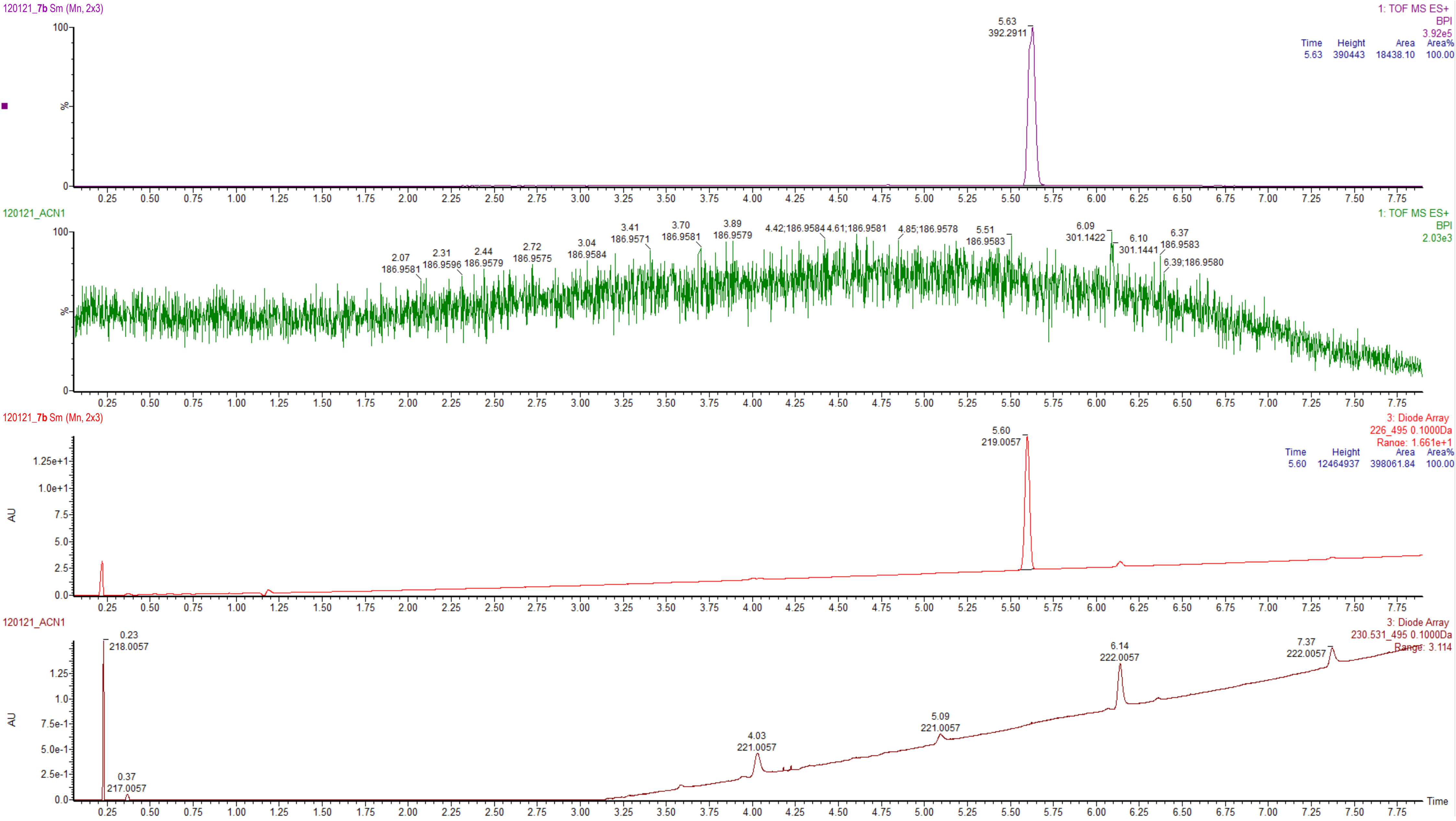

7b

[Back ↑]

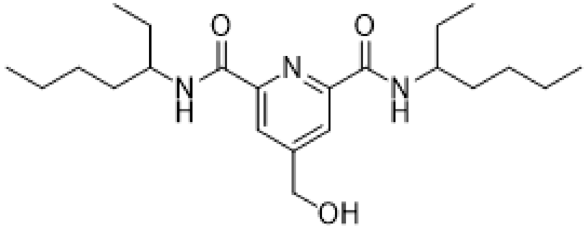

Chemical Formula: $C_{22}H_{37}N_3O_3$
Exact Mass: 391.2835
Molecular Weight: 391.5560

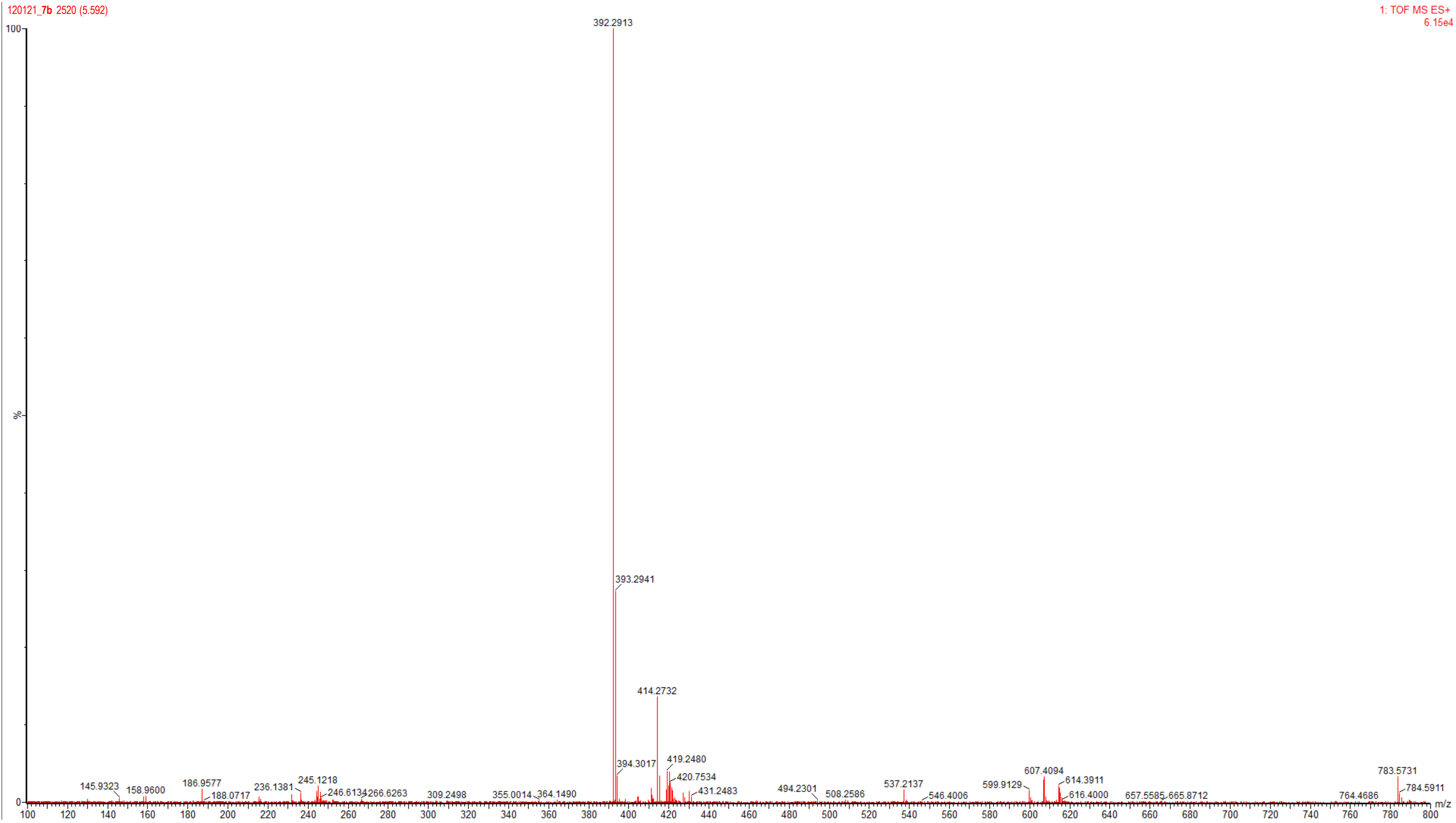

7b

[Back ↑]

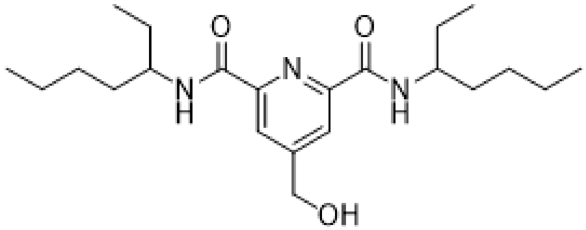

Chemical Formula: $C_{22}H_{37}N_3O_3$
Exact Mass: 391.2835
Molecular Weight: 391.5560

Single Mass Analysis
Tolerance = 50.0 mDa / DBE: min = -1.5, max = 50.0
Element prediction: Off
Number of isotope peaks used for i-FIT = 5
Monoisotopic Mass, Even Electron Ions
342 formula(e) evaluated with 24 results within limits (all results (up to 1000) for each mass)
Elements Used:

Mass	Calc. Mass	mDa	PPM	DBE	Formula	i-FIT	i-FIT Norm	Fit Conf %	C	H	N	O
392.2913	392.2913	0.0	0.0	5.5	C22 H38 N3 O3	241.6	3.362	3.47	22	38	3	3
	392.2953	-4.0	-10.2	9.5	C27 H38 N O	244.5	6.168	0.21	27	38	1	1
	392.2873	4.0	10.2	1.5	C17 H38 N5 O5	248.4	10.071	0.00	17	38	5	5
	392.2814	9.9	25.2	10.5	C24 H34 N5	240.3	2.002	13.51	24	34	5	
	392.3012	-9.9	-25.2	0.5	C20 H42 N O6	246.1	7.866	0.04	20	42	1	6
	392.2801	11.2	28.6	5.5	C23 H38 N O4	242.4	4.107	1.65	23	38	1	4
	392.3026	-11.3	-28.8	5.5	C21 H38 N5 O2	242.1	3.860	2.11	21	38	5	2
	392.2761	15.2	38.7	1.5	C18 H38 N3 O6	247.8	9.479	0.01	18	38	3	6

C22 H38 N3 O3

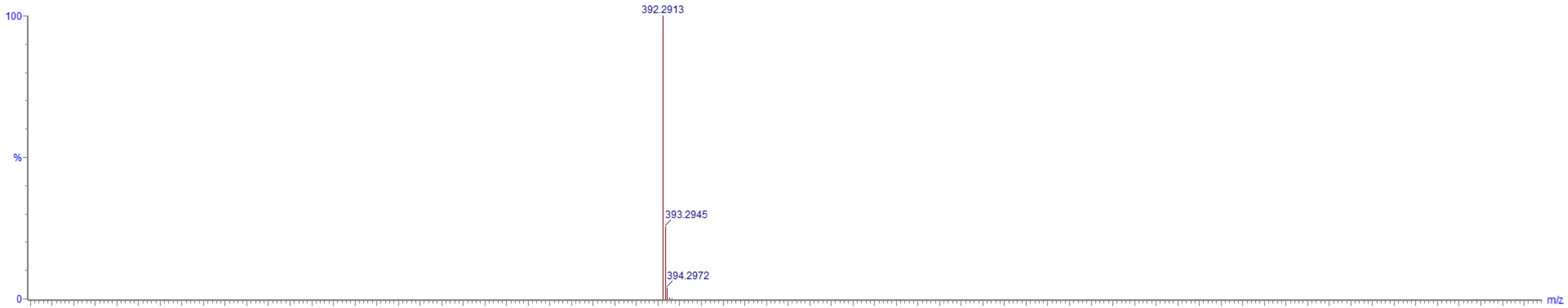

120121_7b 2520 (5.592)

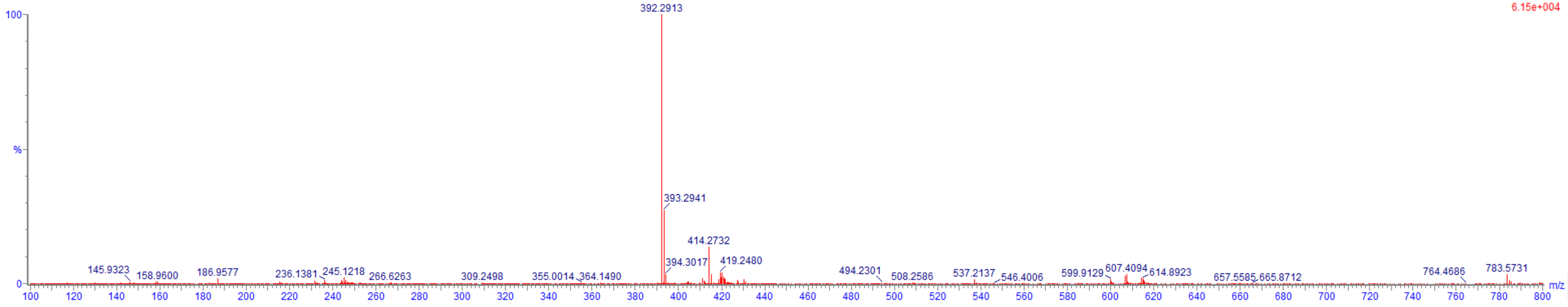

[Back ↑]

7c

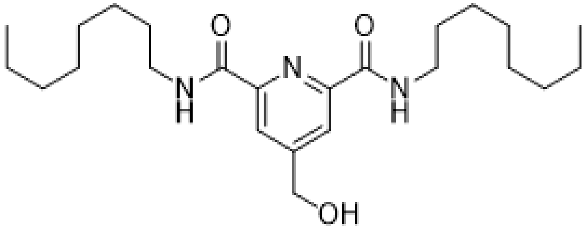

Chemical Formula: $C_{24}H_{41}N_3O_3$
Exact Mass: 419.3148
Molecular Weight: 419.6100

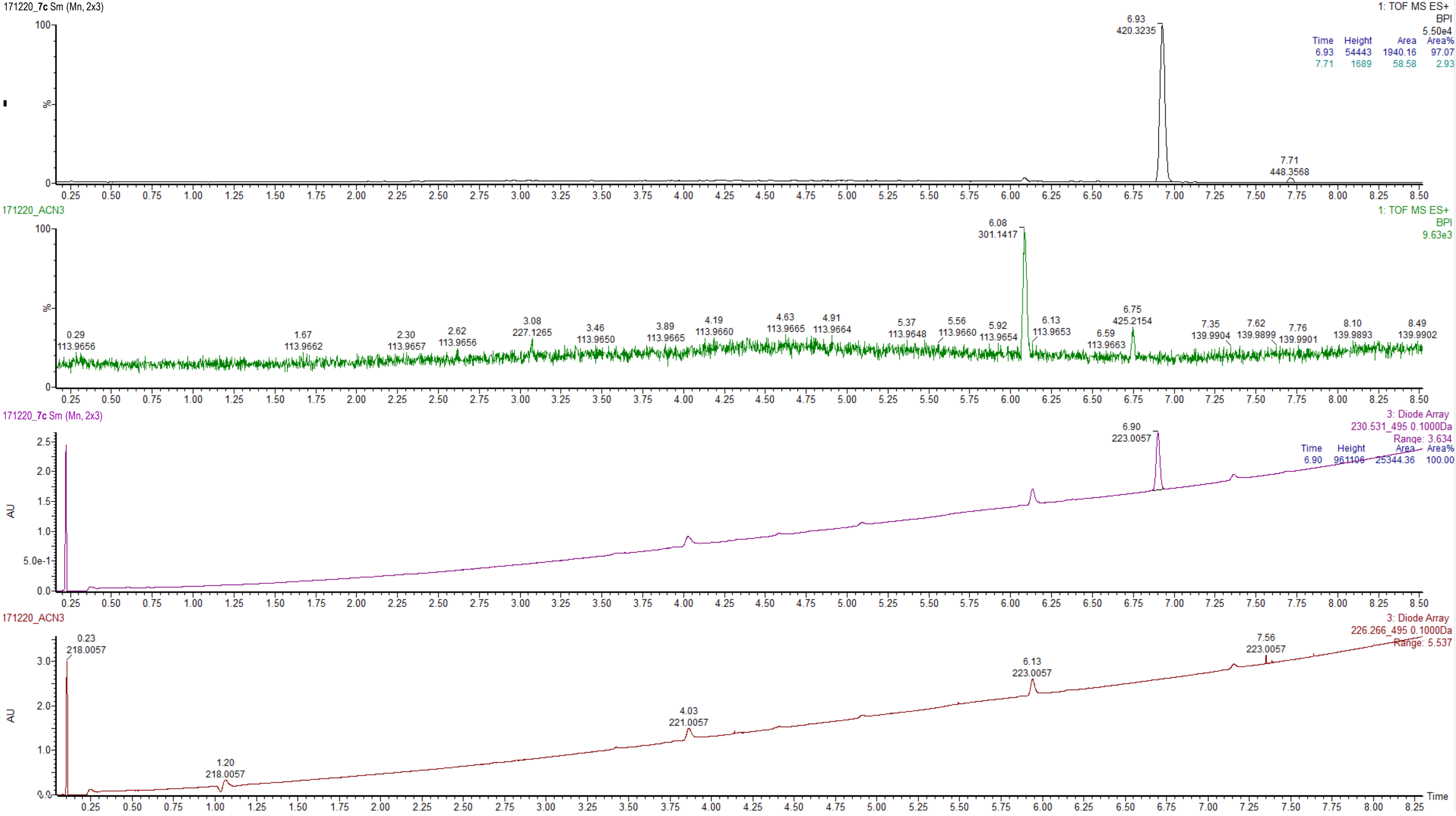

7c

[Back ↑]

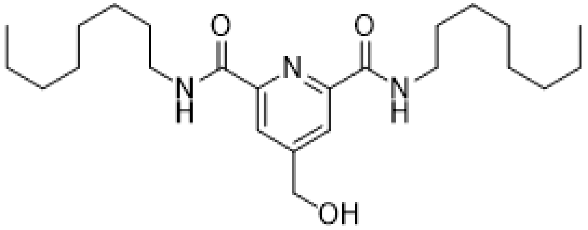

Chemical Formula: $C_{24}H_{41}N_3O_3$
Exact Mass: 419.3148
Molecular Weight: 419.6100

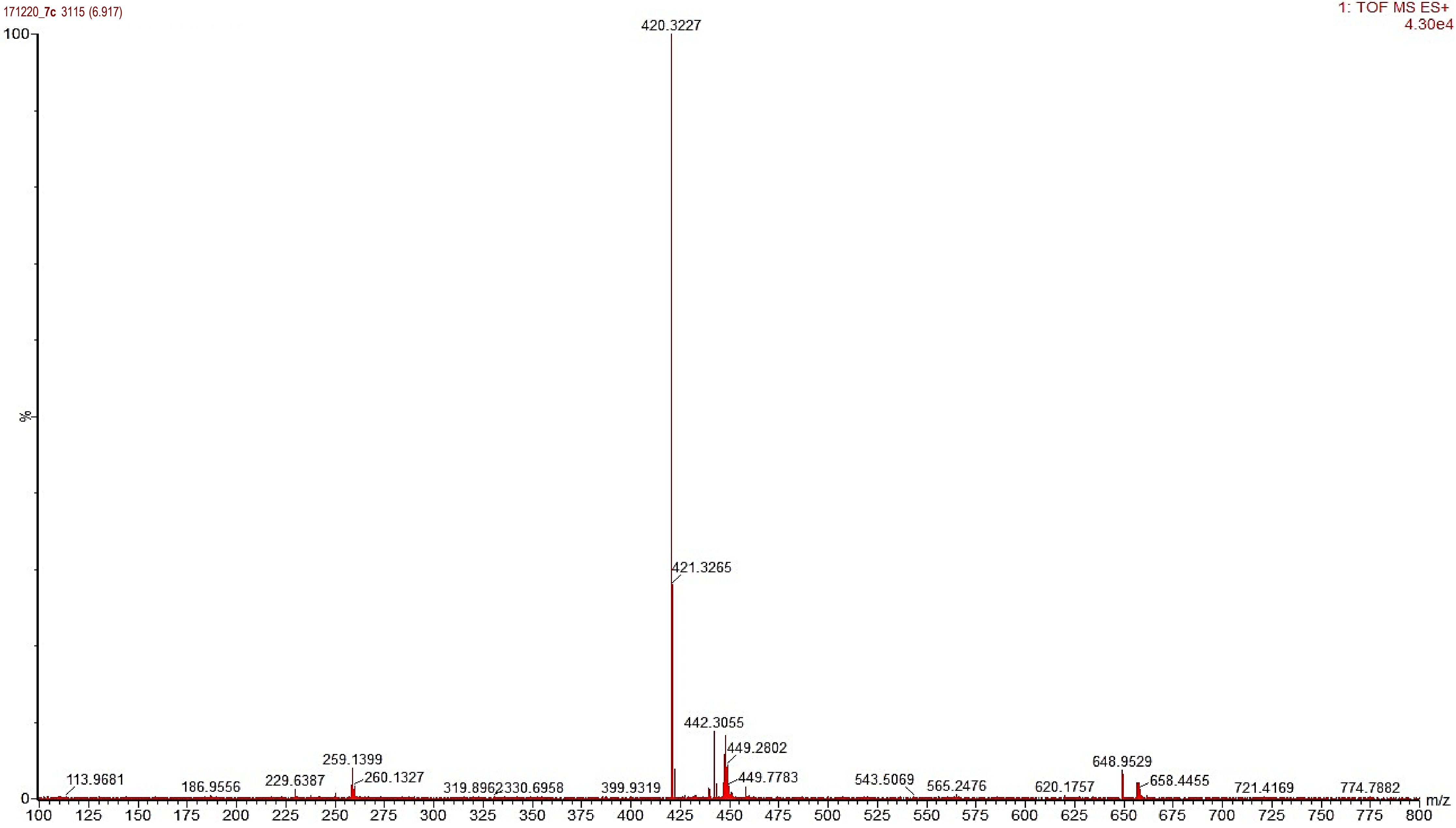

7c

[Back ↑]

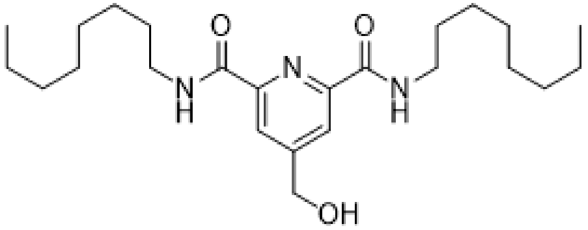

Chemical Formula: $C_{24}H_{41}N_3O_3$
Exact Mass: 419.3148
Molecular Weight: 419.6100

Single Mass Analysis
Tolerance = 50.0 mDa / DBE: min = -1.5, max = 50.0
Element prediction: Off
Number of isotope peaks used for i-FIT = 5
Monoisotopic Mass, Even Electron Ions
5870 formula(e) evaluated with 88 results within limits (all results (up to 1000) for each mass)
Elements Used:

Mass	Calc. Mass	mDa	PPM	DBE	Formula	i-FIT	i-FIT Norm	Fit Conf %	C	H	N	O	Cl	Br
420.3227	420.3226	0.1	0.2	5.5	C24 H42 N3 O3	312.2	4.272	1.40	24	42	3	3		
	420.3231	-0.4	-1.0	-1.5	C9 H38 N15 O4	319.3	11.350	0.00	9	38	15	4		
	420.3218	0.9	2.1	1.5	C19 H43 N7 O Cl	331.4	23.513	0.00	19	43	7	1	1	
	420.3244	-1.7	-4.0	0.5	C23 H47 N O3 Cl	331.5	23.556	0.00	23	47	1	3	1	
	420.3205	2.2	5.2	-1.5	C23 H51 N Br	332.4	24.498	0.00	23	51	1			1
	420.3199	2.8	6.7	6.5	C20 H38 N9 O	310.5	2.602	7.42	20	38	9	1		
	420.3266	-3.9	-9.3	9.5	C29 H42 N O	314.0	6.109	0.22	29	42	1	1		
	420.3186	4.1	9.8	1.5	C19 H42 N5 O5	313.6	5.619	0.36	19	42	5	5		

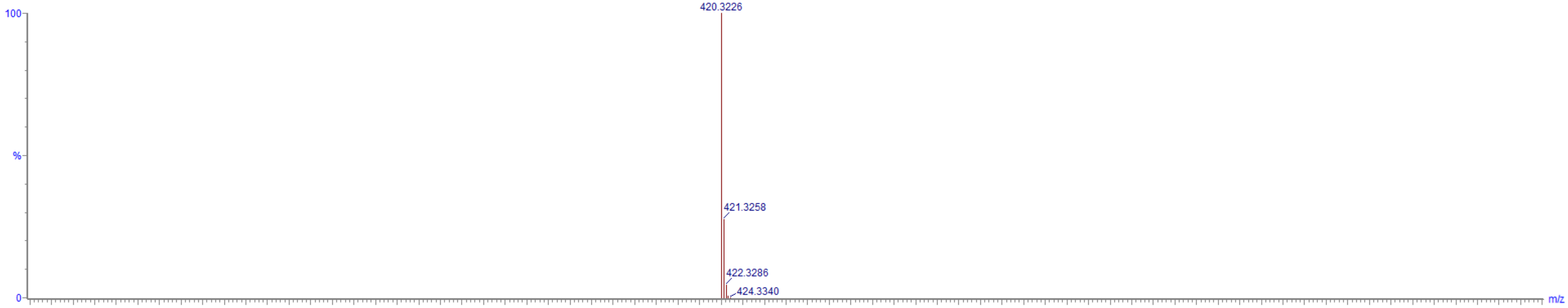

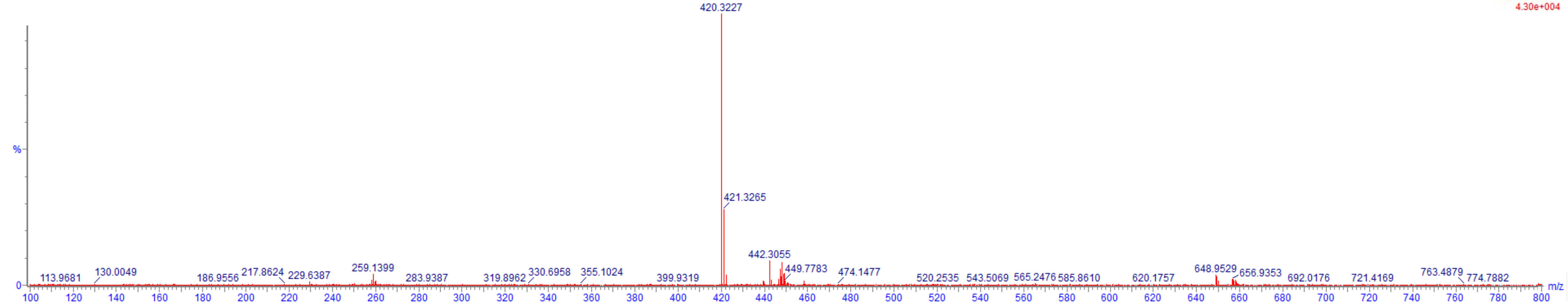

[Back ↑]

8a

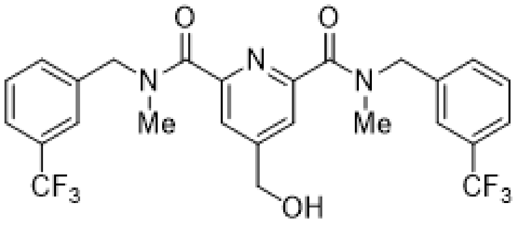

Chemical Formula: $C_{26}H_{23}F_6N_3O_3$
Exact Mass: 539.1644
Molecular Weight: 539.4784

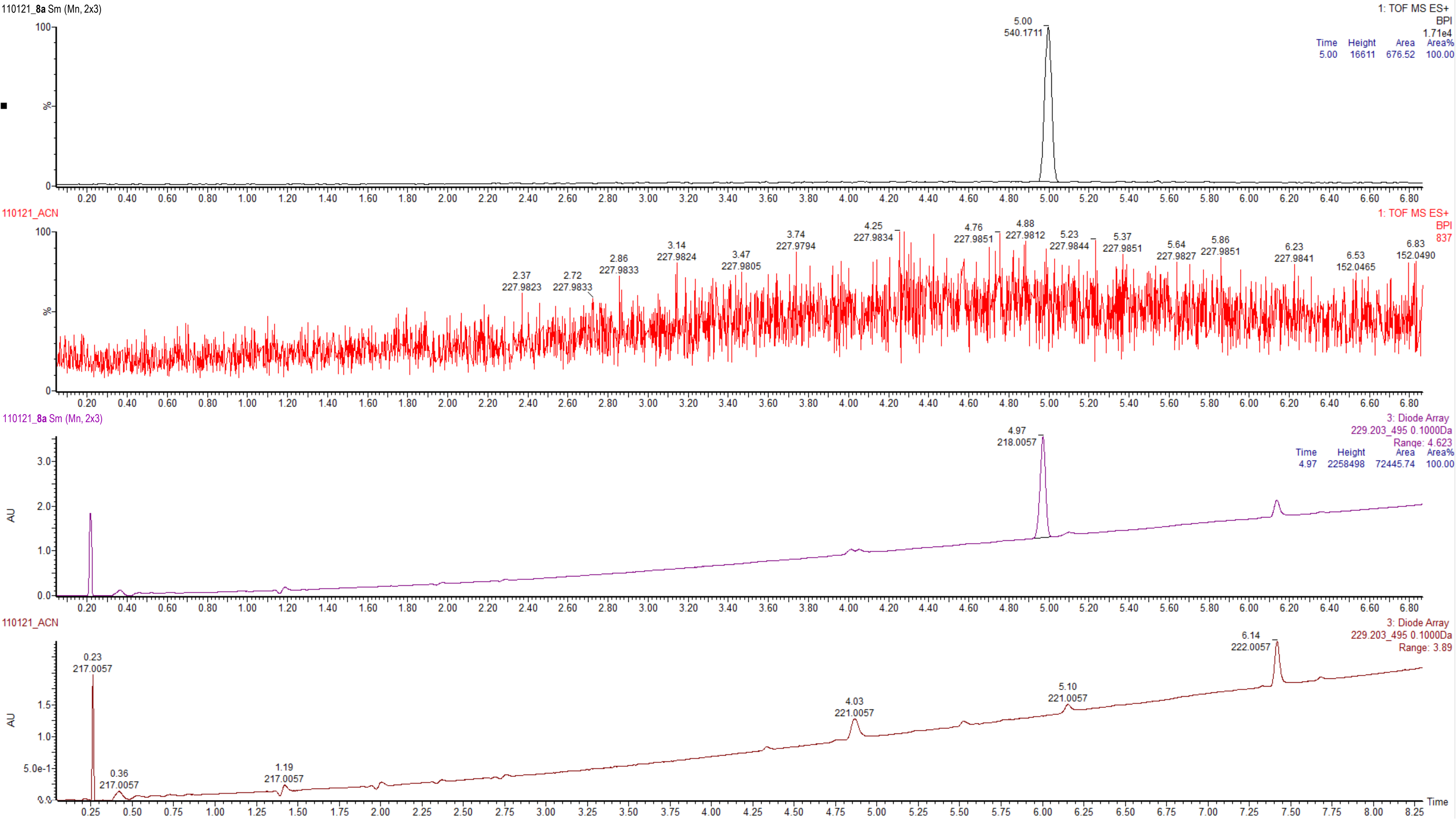

[Back ↑]

8a

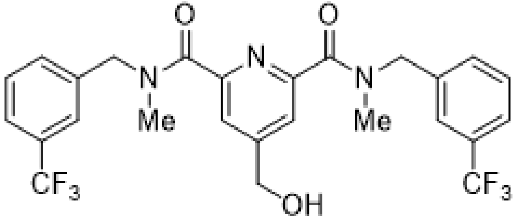

Chemical Formula: $C_{26}H_{23}F_6N_3O_3$
Exact Mass: 539.1644
Molecular Weight: 539.4784

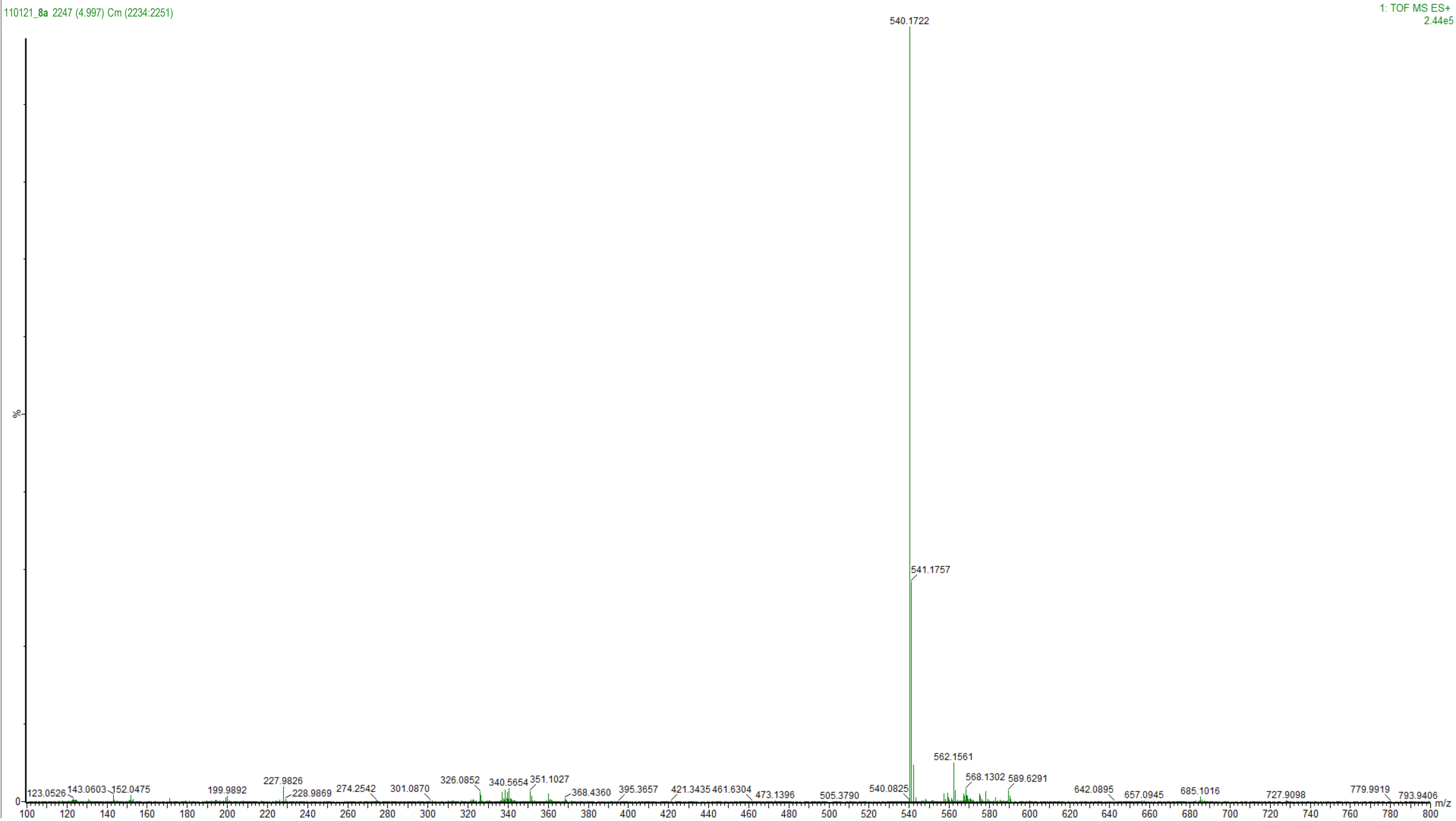

8a

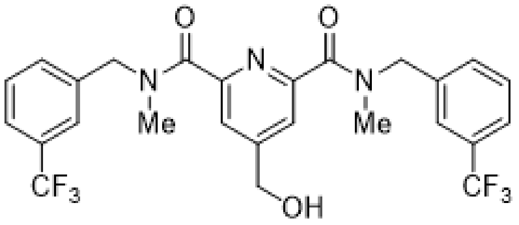

Chemical Formula: $C_{26}H_{23}F_6N_3O_3$
Exact Mass: 539.1644
Molecular Weight: 539.4784

Single Mass Analysis
Tolerance = 50.0 mDa / DBE: min = -1.5, max = 50.0
Element prediction: Off
Number of isotope peaks used for i-FIT = 5
Monoisotopic Mass, Even Electron Ions
3185 formula(e) evaluated with 280 results within limits (all results (up to 1000) for each mass)
Elements Used:

Mass	Calc. Mass	mDa	PPM	DBE	Formula	i-FIT	i-FIT Norm	Fit Conf %	C	H	N	O	F
540.1722	540.1722	0.0	0.0	13.5	C26 H24 N3 O3 F6	355.4	3.859	2.11	26	24	3	3	6
	540.1723	-0.1	-0.2	24.5	C34 H23 N3 O3 F	367.5	15.977	0.00	34	23	3	3	1
	540.1718	0.4	0.7	8.5	C20 H26 N5 O8 F4	361.7	10.111	0.00	20	26	5	8	4
	540.1729	-0.7	-1.3	4.5	C17 H27 N5 O9 F5	364.8	13.265	0.00	17	27	5	9	5
	540.1731	-0.9	-1.7	15.5	C25 H26 N5 O9	363.0	11.422	0.00	25	26	5	9	
	540.1712	1.0	1.9	28.5	C37 H22 N3 O2	368.9	17.346	0.00	37	22	3	2	
	540.1733	-1.1	-2.0	9.5	C23 H25 N3 O4 F7	357.8	6.195	0.20	23	25	3	4	7
	540.1710	1.2	2.2	17.5	C29 H23 N3 O2 F5	362.2	10.639	0.00	29	23	3	2	5

C26 H24 N3 O3 F6

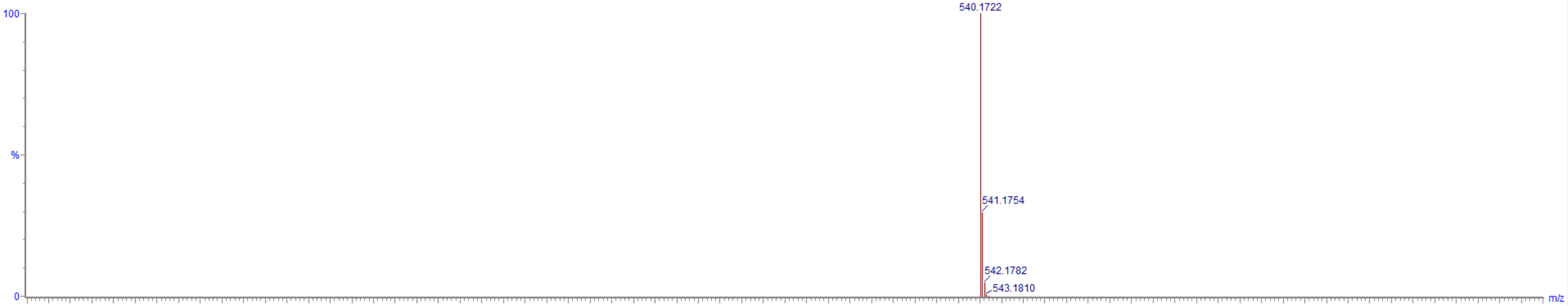

110121_8a 2247 (4.997) Cm (2234:2251)
1: TOF MS ES+
2.44e+005

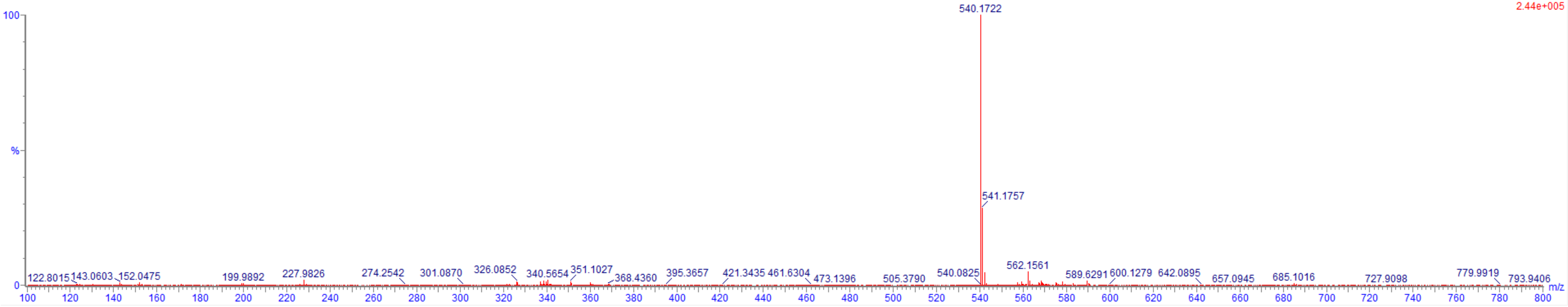

[Back ↑]

8b

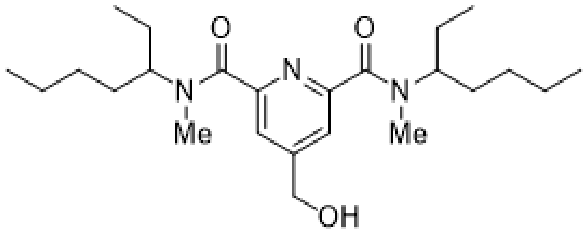

Chemical Formula: $C_{24}H_{41}N_3O_3$
Exact Mass: 419.3148
Molecular Weight: 419.6100

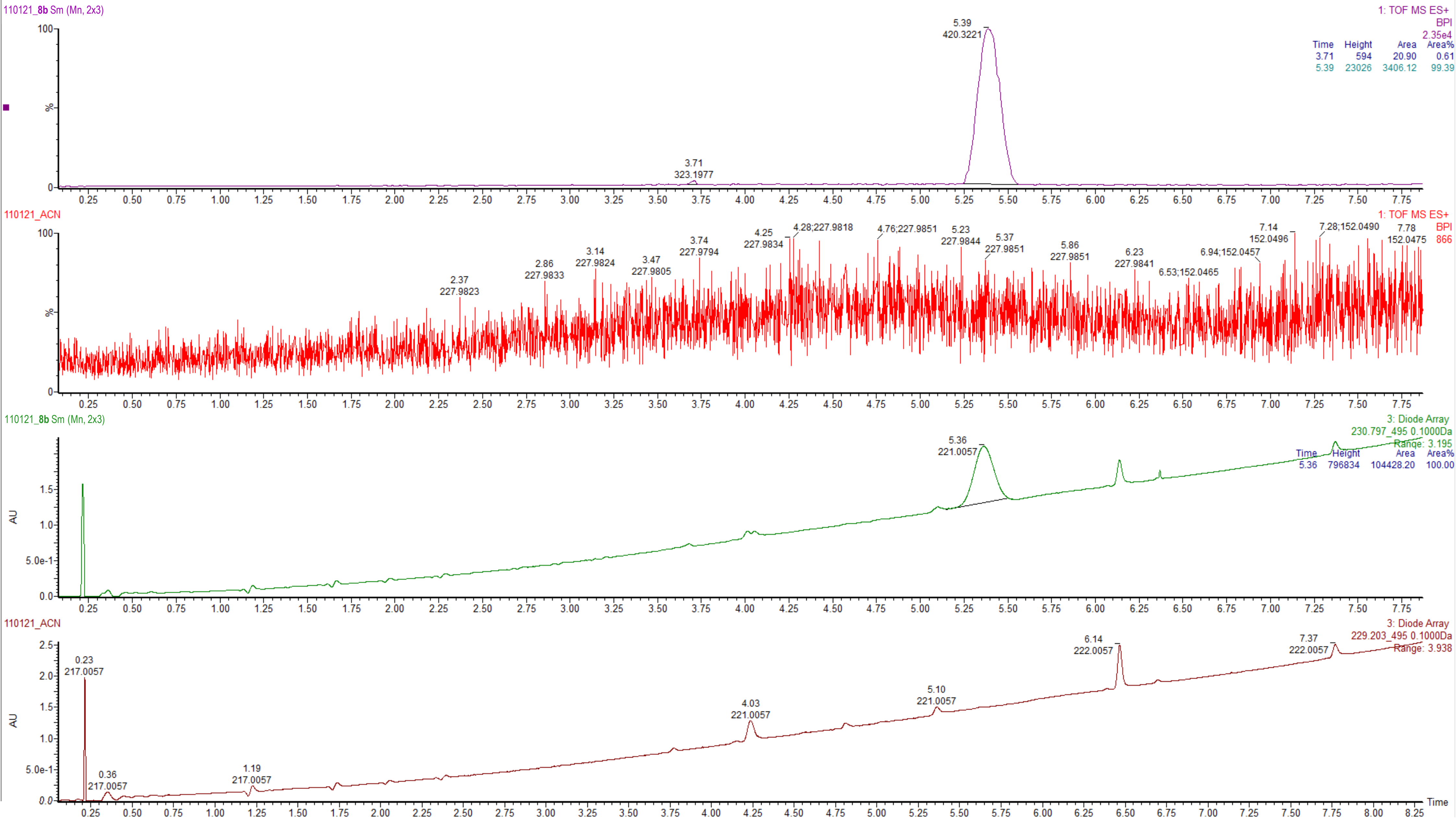

[Back ↑]

8b

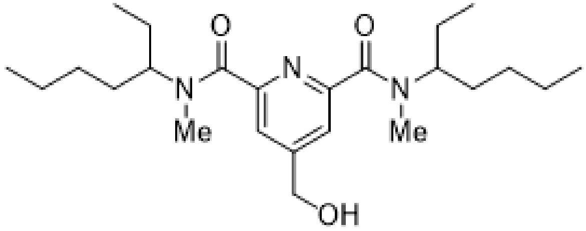

Chemical Formula: $C_{24}H_{41}N_3O_3$
Exact Mass: 419.3148
Molecular Weight: 419.6100

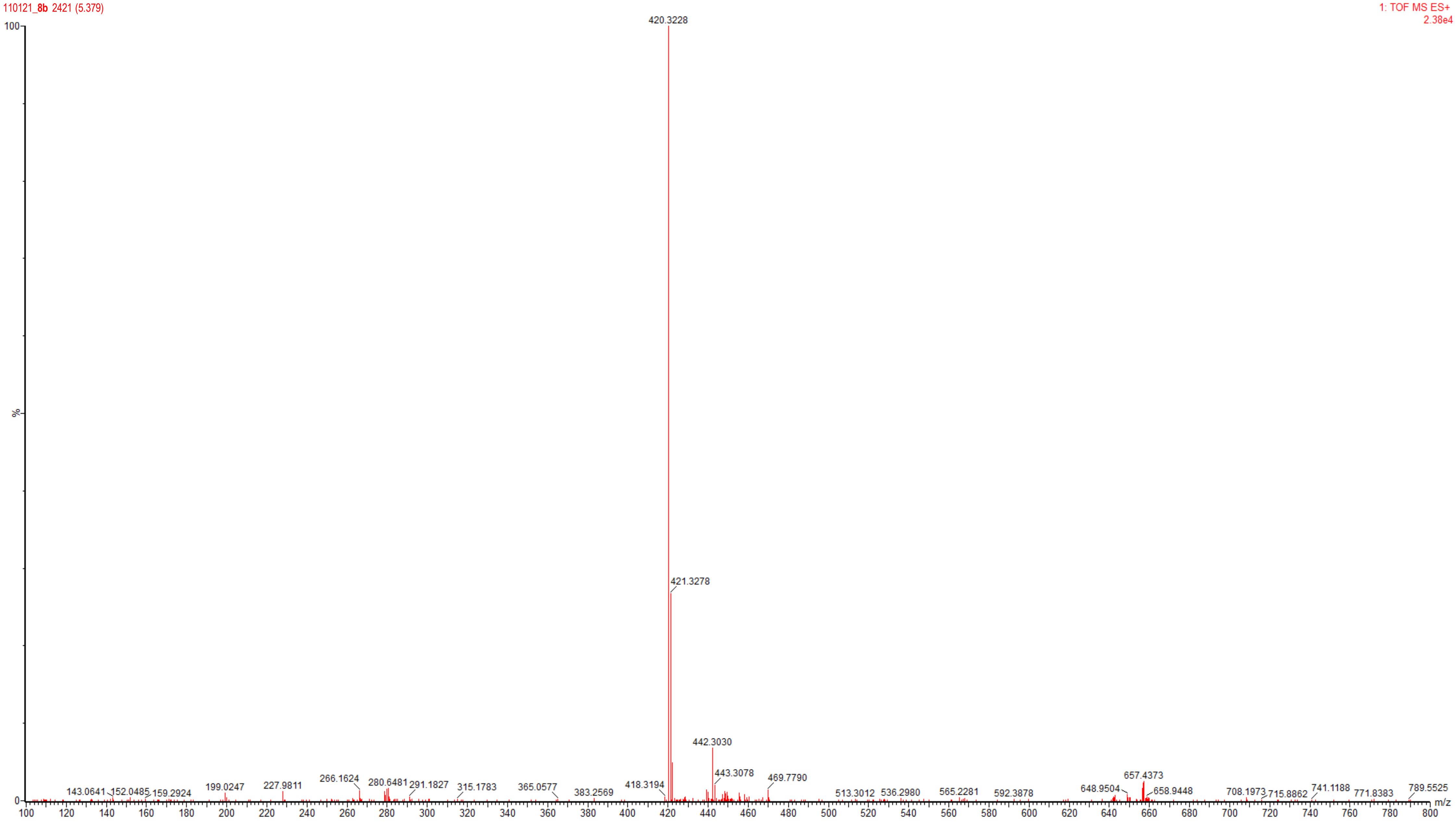

8b

[Back ↑]

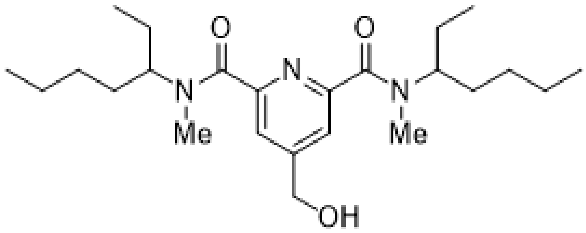

Chemical Formula: $C_{24}H_{41}N_3O_3$
Exact Mass: 419.3148
Molecular Weight: 419.6100

Single Mass Analysis
Tolerance = 50.0 mDa / DBE: min = -1.5, max = 50.0
Element prediction: Off
Number of isotope peaks used for i-FIT = 5
Monoisotopic Mass, Even Electron Ions
1643 formula(e) evaluated with 46 results within limits (all results (up to 1000) for each mass)
Elements Used:

Mass	Calc. Mass	mDa	PPM	DBE	Formula	i-FIT	i-FIT Norm	Fit Conf %	C	H	N	O	Na	Br
420.3228	420.3226	0.2	0.5	5.5	C24 H42 N3 O3	58.1	2.097	12.28	24	42	3	3		
	420.3242	-1.4	-3.3	6.5	C27 H43 N O Na	59.8	3.843	2.14	27	43	1	1	1	
	420.3205	2.3	5.5	-1.5	C23 H51 N Br	86.1	30.059	0.00	23	51	1			1
	420.3202	2.6	6.2	2.5	C22 H43 N3 O3 Na	60.0	4.001	1.83	22	43	3	3	1	
	420.3266	-3.8	-9.0	9.5	C29 H42 N O	62.3	6.291	0.19	29	42	1	1		
	420.3186	4.2	10.0	1.5	C19 H42 N5 O5	63.3	7.275	0.07	19	42	5	5		
	420.3162	6.6	15.7	-1.5	C17 H43 N5 O5 Na	65.7	9.735	0.01	17	43	5	5	1	
	420.3314	-8.6	-20.5	2.5	C21 H43 N5 O2 Na	61.8	5.801	0.30	21	43	5	2	1	

C24 H42 N3 O3

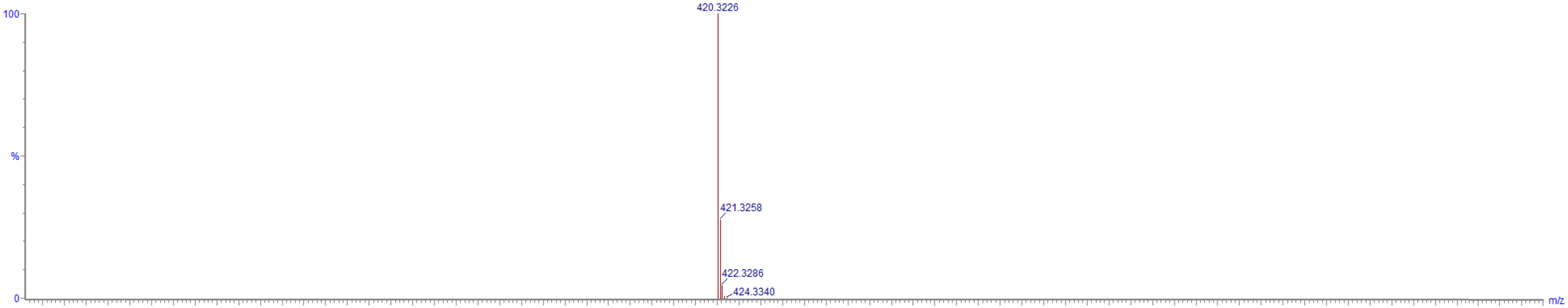

110121_8b 2421 (5.379)
1: TOF MS ES+
2.38e+004

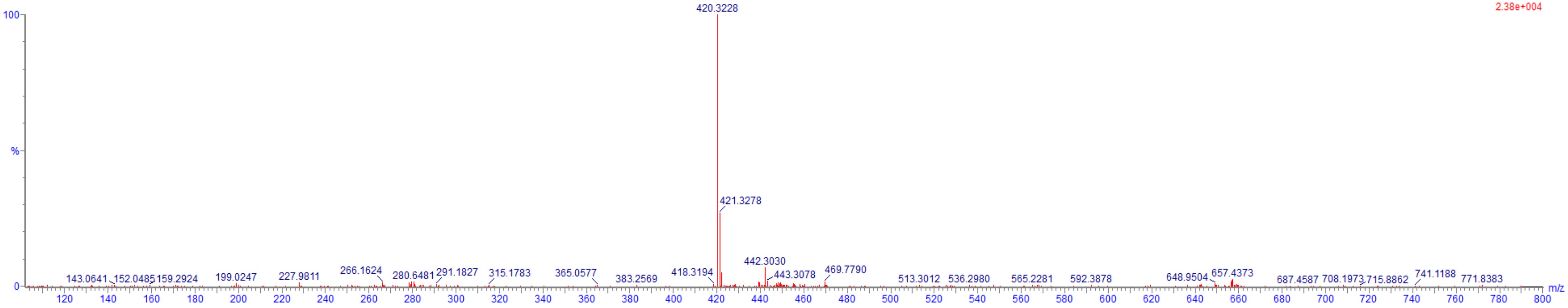

Supplement: Supplementary file 1 — jm2c01448_si_001.pdf [file jm2c01448_si_001.pdf]
